# Second-Strand Synthesis-Based Massively Parallel scRNA-Seq Reveals Cellular States and Molecular Features of Human Inflammatory Skin Pathologies

**DOI:** 10.1016/j.immuni.2020.09.015

**Published:** 2020-10-13

**Authors:** Travis K. Hughes, Marc H. Wadsworth, Todd M. Gierahn, Tran Do, David Weiss, Priscila R. Andrade, Feiyang Ma, Bruno J. de Andrade Silva, Shuai Shao, Lam C. Tsoi, Jose Ordovas-Montanes, Johann E. Gudjonsson, Robert L. Modlin, J. Christopher Love, Alex K. Shalek

**Affiliations:** 1 Institute for Medical Engineering & Science (IMES), MIT, Cambridge, Massachusetts, USA; 2 Department of Immunology, Harvard Medical School, Boston, MA, USA; 3 Broad Institute of MIT and Harvard, Cambridge, MA, USA; 4 Ragon Institute of MGH, MIT and Harvard, Cambridge, MA, USA; 5 Koch Institute for Integrative Cancer Research, MIT, Cambridge, MA, USA; 6 Department of Chemical Engineering, MIT, Cambridge, MA, USA; 7 Division of Dermatology, David Geffen School of Medicine at UCLA, Los Angeles, CA, USA; 8 Department of Dermatology, University of Michigan, Ann Arbor, MI, USA; 9 Division of Gastroenterology, Boston Children’s Hospital, Boston, MA, USA; 10 Department of Pediatrics, Harvard Medical School, Boston, MA, USA; 11 Harvard Stem Cell Institute, Cambridge, MA, USA; 12 Department of Chemistry, MIT, Cambridge, Massachusetts, USA; 13 Department of Microbiology, Immunology and Molecular Biology, David Geffen School of Medicine at UCLA, Los Angeles, CA, USA

**Keywords:** single-cell RNA sequencing, scRNA-seq, Seq-Well, skin inflammation, acne, alopecia areata, granuloma annulare, leprosy, psoriasis

## Abstract

High-throughput single-cell RNA-sequencing (scRNA-seq) methodologies enable characterization of complex biological samples by increasing the number of cells that can be profiled contemporaneously. Nevertheless, these approaches recover less information per cell than low-throughput strategies. To accurately report the expression of key phenotypic features of cells, scRNA-seq platforms are needed that are both high fidelity and high throughput. To address this need, we created Seq-Well S^3^ (“Second-Strand Synthesis”), a massively parallel scRNA-seq protocol that uses a randomly primed second-strand synthesis to recover complementary DNA (cDNA) molecules that were successfully reverse transcribed but to which a second oligonucleotide handle, necessary for subsequent whole transcriptome amplification, was not appended due to inefficient template switching. Seq-Well S^3^ increased the efficiency of transcript capture and gene detection compared with that of previous iterations by up to 10- and 5-fold, respectively. We used Seq-Well S^3^ to chart the transcriptional landscape of five human inflammatory skin diseases, thus providing a resource for the further study of human skin inflammation.

## Highlights

• Seq-Well S^3^ uses second-strand synthesis to improve transcript capture
• Seq-Well S^3^ was benchmarked against a best-in-class commercial platform
• Seq-Well S^3^ was applied to profile inflammatory cell states in skin diseases
• Analysis of skin inflammation uncovered unique and conserved cellular phenotypes

## Graphical Abstract

**Figure:**
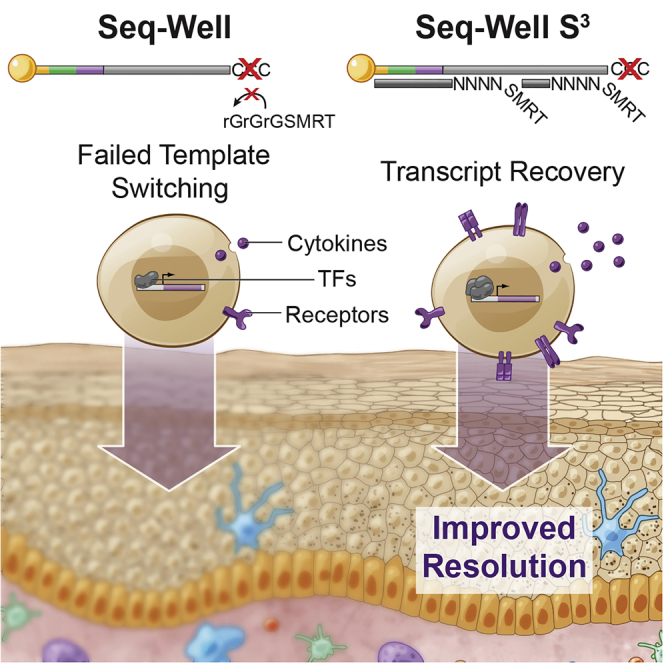

## Teaser

Hughes et al. report the development of a technique for high-throughput single-cell RNA-sequencing, “Seq-Well S^3^,” that enables increased sensitivity and improved detection of genes including transcription factors, cytokines, and cytokine receptors. Using Seq-Well S^3^, the authors define inflammatory cell states across multiple skin diseases.

## Introduction

Single-cell RNA-sequencing (scRNA-seq) is a powerful approach to define the cellular composition of healthy and diseased tissues (Klein et al., 2015; Macosko et al., 2015; Montoro et al., 2018; Ordovas-Montanes et al., 2018; Smillie et al., 2019; Vento-Tormo et al., 2018). The development of high-throughput methodologies has enabled the characterization of increasingly complex cellular samples. However, current scRNA-seq platforms typically demonstrate an inverse relationship between the number of cells that can be profiled at once and the amount of biological information that can be recovered from each cell. Thus, one must choose between quantity and quality, or alternatively employ two distinct approaches in parallel (Consortium et al., 2018). Indeed, inefficiencies in transcript capture among massively parallel methods limit our ability to resolve the distinct cell states that comprise broad cell types (Vieira Braga et al., 2019) and their essential but often lowly expressed molecular features, such as transcription factors, affinity receptors, and signaling molecules.

Improving the fidelity of scRNA-seq is particularly important for resolving differences within heterogeneous populations of immune cells (Dutertre et al., 2019; Villani et al., 2017). Here, subtle differences in surface receptor, transcription factor, and/or cytokine expression can profoundly affect cellular function, particularly in the setting of human pathology (Puel et al., 1998). Enhancing data quality in high-throughput scRNA-seq would facilitate a greater appreciation of the underlying molecular features that describe such cellular variation. Similarly, it would ease integration with legacy datasets that often utilize lowly expressed biomarkers, such as transcription factors and cytokines that are false-negative prone, when discriminating subsets of cells.

Most high-throughput scRNA-seq methods rely on early barcoding of cellular contents to achieve scale. Typically, these techniques recover single-cell transcriptomes for thousands of cells at once by leveraging reverse-emulsion droplets or picowells to isolate individual cells with uniquely barcoded poly-dT oligonucleotides, which can then capture and tag cellular messenger RNAs (mRNAs) during reverse transcription (Prakadan et al., 2017). Afterward, an additional oligonucleotide priming site is typically added to the 3′ end of the synthesized cDNA to enable PCR-based amplification of all transcripts (whole transcriptome amplification [WTA]). A number of techniques have been described to add this second priming site (Sasagawa et al., 2013; Shishkin et al., 2015). A common approach uses the terminal transferase activity of certain reverse transcriptase enzymes to facilitate a “template-switch” from the original mRNA to a second defined oligonucleotide (Picelli et al., 2013). Although simple to implement, this process has the potential to be highly inefficient, leading to the loss of molecules that have been converted to cDNA but not successfully tagged with a secondary PCR priming site (Islam et al., 2012; Kapteyn et al., 2010; Zajac et al., 2013).

To overcome these limitations, we have developed a massively parallel scRNA-seq protocol we call Seq-Well S^3^ (for “Second-Strand Synthesis”), which incorporates the use of a randomly primed second-strand synthesis after reverse transcription to append a second oligonucleotide handle for WTA. In cell lines and peripheral blood mononuclear cells (PBMCs), we demonstrated that Seq-Well S^3^ enables significant improvements in transcript and gene capture. To illustrate the utility of S^3^, we applied it to examine the cellular composition of normal skin and uncover alterations in cellular abundance and phenotype across multiple inflammatory skin conditions, including acne, alopecia areata, granuloma annulare (GA), leprosy, and psoriasis. Overall, our work provides a key methodological advance and a valuable resource for understanding how diverse inflammatory responses can affect a single tissue, as well as the range of cellular phenotypes that are possible upon perturbation.

## Results

### Second-Strand Synthesis (S^3^) Leads to Improved Transcript Capture and Gene Detection

**Figure 1:**
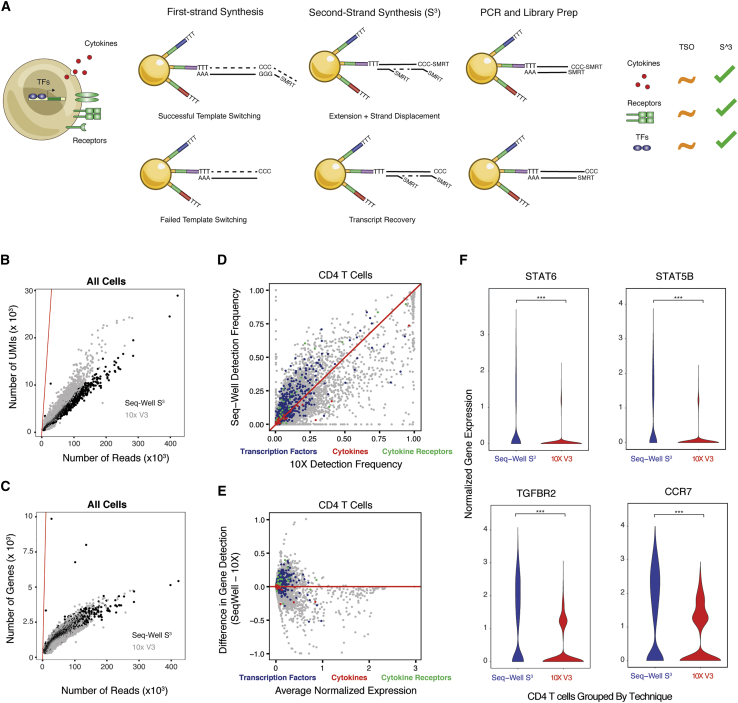
Overview of Second-Strand Synthesis (S^3^) (A) Conceptual illustration of the molecular features that define immune phenotypes as well as the Seq-Well second-strand synthesis method (Seq-Well S^3^). (B) Scatterplot showing differences in per-cell transcript capture (y-axis) as a function of aligned reads per cell (x axis) between 10x Genomics v3 (10x v3, grey) and Seq-Well S^3^ (black) in human PBMCs. Red line indicates where transcripts per cell and aligned reads would be equivalent. (C) Scatterplot shows the differences in per-cell gene detection (y axis) as a function of aligned reads per cell (x axis) between 10x v3 (grey) and Seq-Well S^3^ (black) in human PBMCs. Red line indicates where genes per cell and aligned reads would be equivalent. (D) Scatterplot comparing gene detection rates in CD4^+^ T cells between 10x v3 (x axis) and Seq-Well S^3^ (y axis). Red line indicates point of equivalence in gene detection frequency between methods. Colors correspond to classes of genes including transcription factors (blue), cytokines (red), and receptors (green). See also Table S1. (E) Scatterplot comparing gene detection frequency (y axis) between Seq-Well S^3^ (positive values) and 10x v3 (negative values) as a function of the average expression amounts (log(scaled UMI + 1)) of an individual gene (x axis). Red line indicates point of equivalence in gene detection frequency between methods. Colors correspond to classes of genes including transcription factors (blue), cytokines (red), and receptors (green). See also Table S1. (F) Violin plots of the distribution of normalized expression values (log(scaled UMI + 1)) for select transcription factors and cytokine receptors between Seq-Well S^3^ and 10x v3. ^∗∗∗^p < 1.0 × 10^−10^.

We hypothesized that the use of “template-switching” to append a second PCR handle during reverse transcription might limit the recovery of unique transcripts and genes from individual cells in some massively parallel scRNA-seq methods such as Seq-Well and Drop-Seq (Gierahn et al., 2017; Macosko et al., 2015). Thus, we incorporated a randomly primed second-strand synthesis following first-strand cDNA construction (Figures 1A and S1A). Briefly, after reverse transcription, we washed barcoded mRNA capture beads with 0.1 molar sodium hydroxide to remove attached RNA template strands, and then we performed a randomly primed second-strand synthesis to generate double-stranded cDNA that was labeled on one end with the SMART sequence and its reverse complement on the other (Figures 1A and S1A; STAR Methods) (Picelli et al., 2013; Picelli et al., 2014).

To examine the effectiveness of Seq-Well S^3^, we tested a number of conditions by using cell lines and human PBMCs (Figure S1B; STAR Methods). Here, we observed that S^3^ led to marked improvements in library complexity (i.e., the number of unique transcripts detected per aligned read) at matched sequencing depth below saturation (note: higher library complexity implies a greater amount of information remains to be detected through further sequencing). Seq-Well S^3^ was further able to function in the absence of a template-switching oligo (TSO), whereas Seq-Well v1 failed to generate appreciable product without a TSO (Figures S1B–S1E). In species-mixing experiments using HEK293 (human) and NIH-3T3 (mouse) cell lines, we achieved significant increases in the numbers of unique transcripts and genes detected per cell by using Seq-Well S^3^ compared with Seq-Well v1 (p < 0.05, Mann-Whitney U Test) (Figure S1D; STAR Methods), but comparable single-cell resolution (i.e., transcript purity) (Figures S1F and S1G).

To understand how Seq-Well S^3^ would perform on primary cells, we applied it to human PBMCs (Figures S1C, S1E, S2, and S3; STAR Methods), benchmarking against Seq-Well v1 and multiple versions of a commercial technology (abbreviations for such are as follows: 10x Genomics, v2 3′ chemistry: 10x v2; 10 Genomics, v3 3′ chemistry: 10x v3). Here, we down-sampled all resulting data to equivalent numbers of aligned reads per cell to account for differences in sequencing depth (for comparisons between Seq-Well S^3^ and 10x v2: 38,000 reads per cell; between Seq-Well S^3^ v10x v3: 47,000 reads per cell) (Table S1; STAR Methods).

Critically, when we compared the complexity of sequencing libraries generated by using Seq-Well S^3^ in relation to both Seq-Well v1 and 10x v2, we found that Seq-Well S^3^ significantly increased the number of transcripts and genes detected at matched read depth (p < 0.05, Mann-Whitney U Test & Linear Regression) (Figures S1C, S1E, and S2A; STAR Methods). Both Seq-Well S^3^ and 10x v2 displayed increased sensitivity compared with that of Seq-Well v1 (Seq-Well S^3^: 6-fold gene detection, 10-fold unique molecular identifier [UMI] detection), but Seq-Well S^3^ detected genes and transcripts for each cell type more efficiently than 10x v2 (defined as genes recovered at matched read depth) (Figure S2). Further, comparing Seq-Well S^3^ to 10x v3 across PBMC cell types in aggregate (average read depth: 47,000 reads per cell), we observed that Seq-Well S^3^ detected more genes per cell (Seq-Well S^3^: 1,402 ± 739 genes per cell; 10x v3: 1,225 ± 496 genes per cell), whereas 10x v3 detected more transcripts per cell at comparable sequencing depth (Seq-Well S^3^: 3,247 ± 2,418 UMIs per cell; 10x v3: 4,268 ± 2,109 UMIs per cell) (Figures 1B and 1C; Table S1).

We examined each cell type separately to confirm that these improvements were not driven by changes in the relative frequencies of different cell types (Figures S2B, S2C, S3A, and S3B). Among CD4^+^ T cells, for example, we observed significant increases in the numbers of transcripts captured and genes detected by using Seq-Well S^3^ in pairwise comparisons against 10x v2 (p < 0.05, Mann-Whitney U Test; CD4^+^ T cells, Seq-Well v1: 1,044 ± 62.3 UMIs per cell; 10x v2: 7,671 ± 103.9 UMIs per cell; Seq-Well S^3^: 13,390 ± 253.4 UMIs per cell; mean ± standard error of the median [SEM]) (Figure S2D; Table S1; STAR Methods). Meanwhile, in comparison with 10x v3, we observed that Seq-Well S^3^ detected more genes per cell (Seq-Well S^3^: 1,226 ± 604 genes per cell; 10x v3: 1,083 ± 246 genes per cell), whereas 10x v3 detected more transcripts per cell at comparable sequencing depth (Seq-Well S^3^: 2,739 ± 1,861 UMIs per cell; 10x v3: 4,047 ± 1,165 UMIs per cell) (Figure S3C; Table S1).

We sought to understand whether these improvements resulted in enhanced detection of biologically relevant genes typically under-represented in high-throughput scRNA-seq libraries (Consortium et al., 2018). Importantly, genes that were differentially detected (i.e., higher in S^3^) within each cell type include numerous transcription factors, cytokines, and cell surface receptors (Figures 1D, 1E, S2E, S2F, S3D, and S3E; Table S1). For example, among CD4^+^ T cells, compared with 10x v3, we observed significantly increased detection of transcription factors (e.g., *STAT6* and *STAT5B*) and cytokine receptors (e.g., *TGFBR2* and *CCR7*) (S^3^ vs. 10x v3, p < 0.05, Chi-Square Test) (Figures 1F and S3; Table S1) in Seq-Well S^3^.

We performed an additional comparison of enriched human CD4^+^ T cells profiled by using Seq-Well S^3^, 10x v2, and Smart-Seq2 (SS2), a commonly implemented microtiter plate-based scRNA-seq approach (Figures S2G–S2I; STAR Methods) (Picelli et al., 2013). Integrated analysis revealed that Seq-Well S^3^ detected more genes per cell than 10x v2 and nearly as many as SS2 in pairwise comparison of the techniques (10x v2: 2,057 ± 18.7 genes per cell; Seq-Well S^3^: 3,514 ± 36.2 genes per cell; SS2: 3,975 ± 74.0 genes per cell; mean ± SEM; p < 0.05, Mann-Whitney U Test) (Figure S2H; STAR Methods). Furthermore, comparing the frequency of gene detection between methods revealed crucial differences for transcription factors, cytokines, and cytokine receptors (STAR Methods). Surprisingly, we observed similar rates of gene detection between Seq-Well S^3^ and SS2 for a large number of biologically informative genes (Figure S2G). Critically, although comparable numbers of genes were detected across methods, Seq-Well S^3^ detected more genes per aligned read than either 10x v2 or SS2 (p < 0.05, Mann-Whitney U Test) (Figure S2I; STAR Methods).

### Diverse Cellular States across Healthy and Inflamed Skin

**Figure 2:**
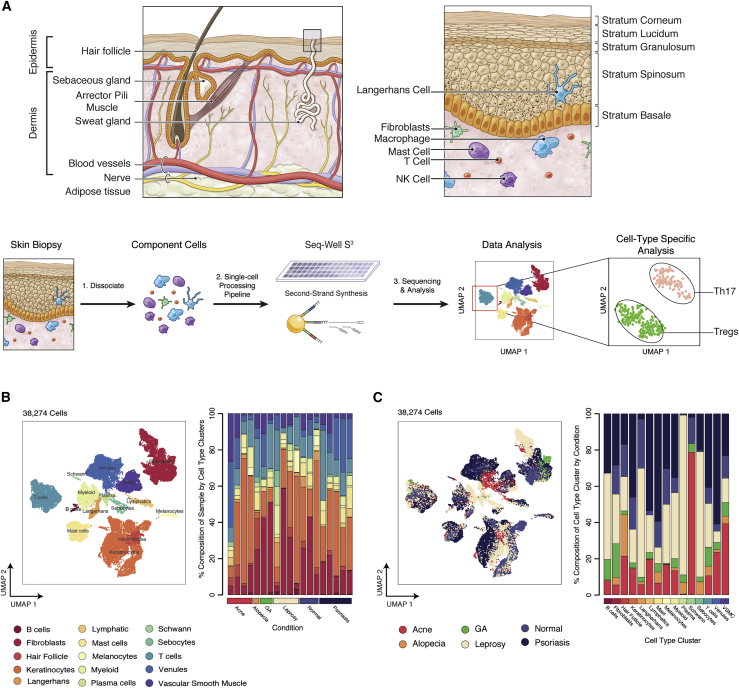
Cell Types Recovered across Inflammatory Skin Conditions (A) (Top, left) Illustration of the anatomic organization and major features of human skin. Shown at the top, right is the cell type composition of the epidermis and dermis. Shown on the bottom is a sample processing pipeline for skin samples (Table S2). (B) (Left) UMAP plot for 38,274 cells colored by cell type cluster. Shown on the right is a stacked barplot depicting the cell type composition for each of the 19 skin biopsies. (C) (Left) UMAP plot for 38,274 cells colored by inflammatory skin condition. Shown on the right is a stacked barplot depicting the proportion of cells from each skin condition within phenotypic clusters.

To demonstrate the utility of Seq-Well S^3^ to profile cellular states in human pathology, we applied it to characterize normal human skin and multiple inflammatory skin conditions, including acne, alopecia areata, GA, leprosy, and psoriasis. In total, we processed 19 skin biopsies (acne, n = 4; alopecia, n = 1; GA, n = 2; leprosy, n = 4; psoriasis, n = 5; normal skin, n = 3) and, after data quality filtering, retained 38,274 high-quality single-cell transcriptomes spanning 35 clusters identified through Louvain clustering in Scanpy (Wolf et al., 2018) (Figures 2A–2C and S4A–S4C; STAR Methods). To collapse clusters to cell types, we performed enrichment analyses to identify cluster-defining genes and manually assigned cell type identities on the basis of the expression of known lineage markers and hierarchical clustering (Figures 2B, S4A, S4C, and S4D; Table S3; STAR Methods). We further classified cells by using SingleR (Aran et al., 2019) and observed close concordance between manually identified cell types and automated classification where appropriate reference signatures existed (Figure S4B and Table S2; STAR Methods). Ultimately, we recovered 15 primary cell types, including the following: B cells (marked by expression of *MS4A1* and *CD79A*), fibroblasts (*DCN* and *COL6A2*), hair follicles (*SOX9*), keratinocytes (KCs) (*KRT5* and *KRT1*), Langerhans cells (LCs) (*CD207*), lymphatic endothelial cells (*LYVE1*), mast cells (*CPA3* and *IL1RL1*), melanocytes (*MLANA*), myeloid cells (*CD68* and *CTSS*), plasma cells (*IGHG1*), Schwann cells (*SCN7A*), sebocytes (*DCD*), T cells (*CD3D* and *TRBC2*), venular endothelial cells (ECs) (*SELE* and CD93), and vascular smooth muscle cells (VSMCs) (*TAGLN*) (Figures 2 and S4A–S4E; Table S3). As a final quality measure, we examined the distribution of reads, transcripts, and genes within each major cell population and observed consistent coverage (Figure S4F; Table S2).

### Seq-Well S^3^ Describes T Cell States across Inflammatory Skin Conditions

**Figure 3:**
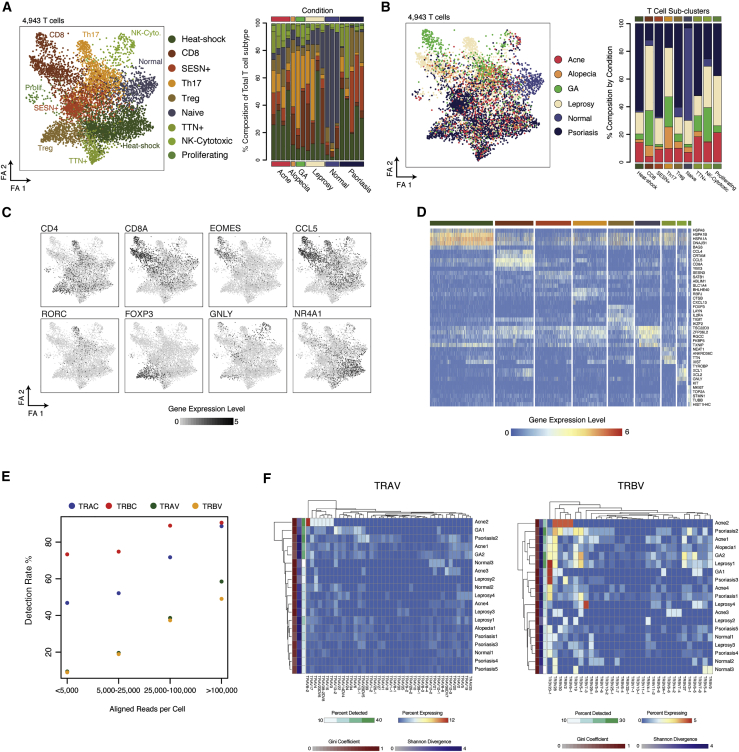
Identification of Inflammatory T cell States by using Seq-Well S^3^ (A) (Left) Force-directed graph of 4,943 T cells colored by phenotypic sub-cluster. Shown on the right is a stacked barplot depicting the distribution of T cell sub-clusters within each biopsy. (B) (Left) Force-directed graph of 4,943 T cells colored by inflammatory skin condition. Shown on the right is a stacked barplot depicting the contribution of each inflammatory skin condition to the T cell sub-clusters. (C) T cell force-directed graphs displaying normalized expression (log(scaled UMI + 1)) of a curated group of sub-cluster-defining gene. Higher expression values are shown in black. (D) Heatmap showing normalized gene expression values (log(scaled UMI + 1)) for a curated list of sub-cluster-defining genes across nine T cell sub-clusters. See also Table S3. (E) Plot showing rates of detection of TCR genes from human skin T cells across a range of sequencing depths. (F) Heatmaps showing the distribution of TRAV (left) and TRBV (right) gene expression among T cells within each sample. Within each sample (rows), the color represents the percent of T cells expressing a given TRAV or TRBV gene (columns). The sidebar shows the gini coefficient (red), the Shannon Divergence (blue), and the percent of T cells (green) within each sample with non-zero expression of either TRAV or TRBV genes.

To determine the biological features that could be captured by using Seq-Well S^3^, we first examined T cells because each inflammatory skin condition is known to significantly skew T cell phenotypes (Figure 3) (Diani et al., 2015; Lowes et al., 2014). We performed dimensionality reduction and sub-clustering across T cells alone (Figures 3A and 3B; STAR Methods). This revealed nine sub-clusters that closely correspond to natural killer (NK) cells and CD8^+^ T cells, as well as several known CD4^+^ T helper (Th) cell subsets. As before, we used the enhanced sensitivity of Seq-Well S^3^ for lineage defining transcripts to help annotate the identity of each sub-cluster; for example, in regulatory T cells and Th-17 cells, we detected distinct expression of canonical transcription factors (e.g., *FOXP3* and *RORC*, respectively) and immune receptors (e.g. *TIGIT*, *CTLA4*, *IL2RA* and *CXCR6*, respectively) (Figures 3C–3D and S5; Table S3). Additionally, we cross-referenced each sub-cluster’s marker genes against a series of curated signatures in the SaVant database (Lopez et al., 2017) to confirm our assignments, which highlighted similarity to previously characterized T cell and NK cell subsets (Best et al., 2013; Bezman et al., 2012) (Figure S5A; STAR Methods).

We next explored variability in T cell subset composition by skin pathology (Figures 3A and 3B). This revealed potentially varied T cell contributions to different classes of cutaneous inflammation. For example, in two out of four leprosy biopsies, we observed a population enriched for expression of canonical Th-17 genes including *RORC*, which encodes the Th-17-lineage-defining transcription factor RORγt (Ivanov et al., 2006) (Figures 3A and 3B). We further found that a sub-cluster of T cells, which express *NR4A1*, a transcription factor indicative of dysfunctional T cells (Liu et al., 2019), and are enriched for genes involved in nuclear organization (*NEAT* and *ANKRD36*), was over-represented in psoriasis samples (Figures 3B and 3C). We also observed an expansion of regulatory T cells in three of five patients with psoriasis, and an additional population of T cells characterized by expression of *SESN3,* a marker of T cell senescence (Lanna et al., 2017), *SATB1*, and *FURIN* (Figures 3A–3D).

Directed analysis within CD8^+^ T cells revealed a sub-grouping of activated CD8^+^ T cells expressing elevated amounts of several inflammatory cytokines (*TNF*, *CCL4*, and *XCL1*), specific affinity receptors (*FASLG* and *TNFRSF9*), and transcription factors (*KLF9* and *EGR2*); this phenotypic skewing was observed primarily in a patient with GA (Figure S5B, top; Table S3; STAR Methods). We also uncovered considerable variation within the cluster containing cytotoxic T cells and NK cells (cytotoxic), where we found the highest degree of cytotoxic gene expression (*GNLY*, *GZMB*, and *PRF1*) (Table S3). Indeed, sub-clustering analysis of this cytotoxic cluster revealed 3 distinct sub-groups (Figure S5B): (1) a sub-group of NK cells (cytotoxic-1) enriched for expression of *c-KIT*, *RANKL* (TNFSF11), and *GITR* (TNFSFR18); (2) a sub-group of *CD16*^+^ cells (cytotoxic-2) expressing cytotoxic effector molecules (*GNLY*, *PRF1*, and *GZMB*) and NK surface receptors, consistent with either NK cell or tri-cytotoxic cytotoxic T lymphocytes (CTLs) (Balin et al., 2018); and (3) a sub-group of CD8^+^ T cells (cytotoxic-3; marked by *TNFSF8*, *SLAMF1*, *CLEC2D*, and *CD5*) that express both TCR αβ and γδ constant genes (Figure S5B, bottom; Table S3) (Söderström et al., 2010).

Profiling of TCR expression is critical to understand T cell antigen specificity (Zhang et al., 2018). Among *CD4*^+^ T cells obtained from peripheral blood, we recovered TCR-V and TCR-J genes at a higher frequency by using Seq-Well S^3^ than by using 10x v2 (p < 0.05, Chi-square Test) (Figure S5C; STAR Methods), and observed paired detection of *TRAC* and *TRBC* in Seq-Well S^3^ in 1,293 out of 1,485 CD4^+^ T cells (87.1% Paired Detection Rate) (Figure S5C). In the setting of skin inflammation, we detected *TRAC* in 53.5% of T cells, *TRBC* in 76.7% (Figure 3E), and paired detection in 45.1%. Among T cells with at least 25,000 aligned reads, we recovered paired α and β chains in 68.6%. Among cytotoxic cells, we observed expression of γ and δ constant genes (*TRGC* and *TRDC*), whereas the remaining T cell clusters exclusively expressed α and β TCR constant genes (Table S3). These data further suggested that the cytotoxic cluster represents a diverse population of γδ, NK, and cytotoxic CD8^+^ T cells that share common gene expression features and, potentially, roles in inflammation.

Finally, we examined the distribution of TCR V gene expression across inflammatory skin biopsies to identify clonally expanded T cells (Figure 3F; STAR Methods). We found biased distributions of TRAV and TRBV genes (e.g., elevated Gini coefficients and low Shannon Divergence) (Oakes et al., 2017) within multiple biopsies including those from leprosy and acne (Leprosy 2 and Acne 2, TRAV and TRBV Gini Coefficient > 0.85) (Figure 3F).

### Spectrum of Myeloid Cell States in Skin Inflammation

**Figure 4:**
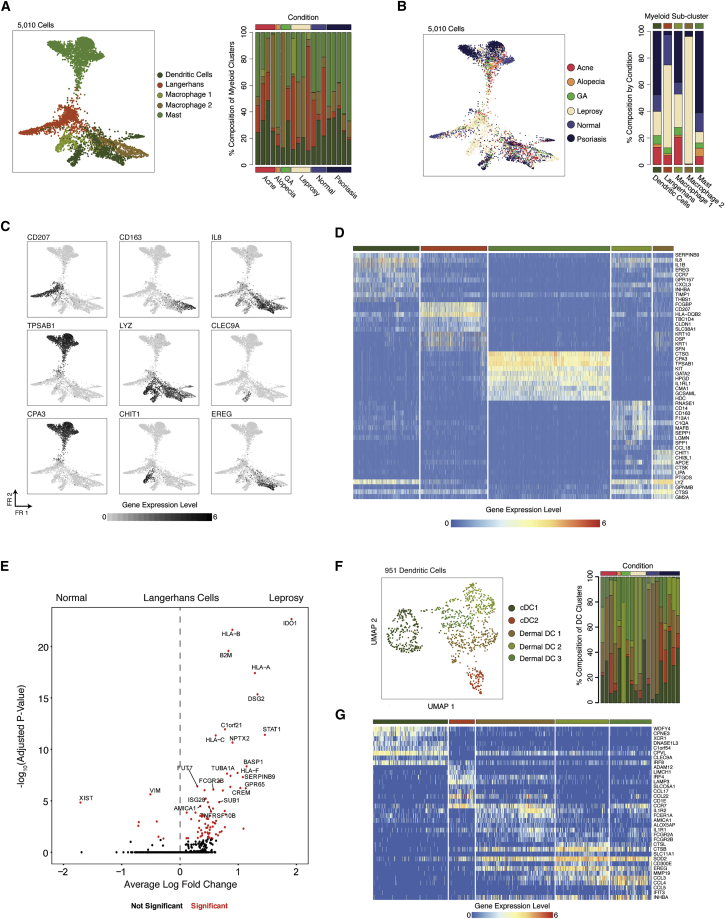
Diverse Myeloid Cell States Uncovered by using Seq-Well S^3^ (A) (Left) Force-directed graph of 5,010 myeloid cells colored by phenotypic sub-cluster (NB, LCs were enriched from leprosy and normal skin). Shown on the right is a stacked barplot showing the distribution of myeloid sub-clusters within each biopsy. See also Table S3. (B) (Left) Force-directed graph of 5,010 myeloid cells colored by inflammatory skin condition. Shown on the right is a stacked barplot showing the contribution of each inflammatory skin condition to each myeloid sub-cluster. (C) Force-directed graphs of 5,010 myeloid cells highlighting expression of a curated group of sub-cluster defining genes (log(scaled UMI + 1)). Higher expression values are shown in black. See also Table S3. (D) Heatmap showing the normalized expression (log(scaled UMI + 1)) of a curated list of myeloid cell type cluster-defining genes. (E) Volcano plot showing genes differentially expressed in LCs between leprosy (n_cells_ = 67) and normal skin (n_cells_ = 171). Log10-fold change values are shown on the x axis and −log10 adjusted p values are shown on the y axis. See also Table S5. (F) (Left) UMAP plot for 951 DCs from human skin colored by inflammatory skin condition. Shown on the right is a stacked barplot showing the distribution DC sub-grouping within 19 skin biopsies. (G) Heatmap showing the distribution of normalized gene expression amounts (log(scaled UMI + 1)) for cluster-defining genes across dermal DC subpopulations. See also Table S3.

In the setting of cutaneous inflammation, myeloid cells play a key role in maintaining tissue homeostasis, wound healing, and response to pathogens (Malissen et al., 2014). We identified numerous myeloid cell subpopulations defined by combinations of surface markers, cytokines, and lineage-defining transcription factors. Specifically, we independently analyzed 5,010 myeloid cells and uncovered 10 sub-clusters representing 4 primary myeloid cell types based on expression of canonical lineage markers and comparison to cell type signatures in the SaVant database: dendritic cells (DCs) (*CLEC9A* and *CLEC10A*), LCs (*CD207* and *CD1A*), macrophages (*CD68* and *CD163*), and mast cells (*CPA3* and *TPSAB1*) (Figures 4A, S5D, and S5E; Table S3; STAR Methods) (Lopez et al., 2017).

Among the macrophages, our data revealed two distinct sub-clusters (Figures 4A and 4B). One spanned normal skin as well as multiple types of skin inflammation and was characterized by elevated expression of previously characterized markers of dermal macrophages (*CD163*, *STAB1*, and *CEPP*) (Fuentes-Duculan et al., 2010). The other, meanwhile, was observed primarily in a single leprosy patient and was defined by genes involved in extracellular proteolysis (*LYZ*, *CHIT1*, and *CHI3L1*) (Di Rosa et al., 2013).

We initially identified LCs cells on the basis of expression of canonical markers (*CD207* and *CD1A*) (Figures 4C and 4D; Table S3) (Romani et al., 2003). When we directly compared LCs between a single leprosy biopsy and a single normal skin biopsy from which we performed bead-based LC enrichment (STAR Methods), we detected elevated expression of *IDO1*, *STAT1*, *HCAR3*, and MHC class I molecules (*HLA-A*, *HLA-B*, and *HLA-F*) in LCs in leprosy infection (Figure 4E; Table S5) (Hunger et al., 2004; Pinheiro et al., 2018). We further performed gene-ontology analysis among genes up-regulated in LCs from leprosy and observed enrichment of genes related to IFN-γ response (Table S5).

Sub-analysis of the DC cluster revealed multiple sub-groups, including conventional and dermal DCs (Figure 4F). Consistent with previous observations from peripheral blood, we detected a sub-group that corresponds to cDC1 (*CLEC9A*, *IRF8*, and *WDFY4*) (Villani et al., 2017) (Dutertre et al., 2019) (p < 0.05, permutation test) (Figure S5F and S5G; STAR Methods). We further identified another representing cDC2 cells (*IRF4*, *SOCS2*, *SLCO5A1*, *CD1B*, and *CD1E*) (Figures 4F, S5H and Table S3; STAR Methods) (Guilliams et al., 2016). Importantly, we detected expression of *IL12B*, a subunit of the IL-23 cytokine, which has previously been shown to promote mucosal type 17 inflammation via secretion of IL-23 (Schlitzer et al., 2013), within these *IRF4*^+^ cDC2 cells (Figure S5I). This sub-grouping of cDC2 cells also expressed high amounts of *CCL17* and *CCL22*, chemokines involved in T cell chemotaxis (Figure 4G) (Stutte et al., 2010).

Among the dermal DCs, we identified 3 subgroups that were broadly distinguished from the conventional DC clusters by expression of *CLEC10A* (Figure S5I)*,* which has been shown to influence T cell cytokine responses in skin (Kashem et al., 2015; Kumamoto et al., 2013). Cells from dermal DC sub-group 1 showed elevated expression of *IL1R1*, *IL1R2*, and *CCR7* and Fc-receptors including *FCER1A*, *FCGR2A*, and *FCGR2B*, which are important for interfacing with humoral immunity (Figure S5I; Table S3) (Guilliams et al., 2014). We observed a second population of dermal DCs (dermal DC sub-group 2), and there was elevated expression of cathepsins (*CTSL* and *CTSB*) and surface receptors (*CD300E* and *SLC11A1*), which collectively represent markers of DC activation (Figure S5I) (Brckalo et al., 2010). Finally, a third sub-grouping of dermal DCs (dermal DC sub-group 3) was distinguished elevated expression of pro-inflammatory chemokines up-regulated during DC maturation (*CCL3*, *CCL4*, and *CCL5*) (Jin et al., 2010) and soluble mediators (*EREG* and *INHBA*).

### Detection of Endothelial Heterogeneity and Vascular Addressin Expression

**Figure 5:**
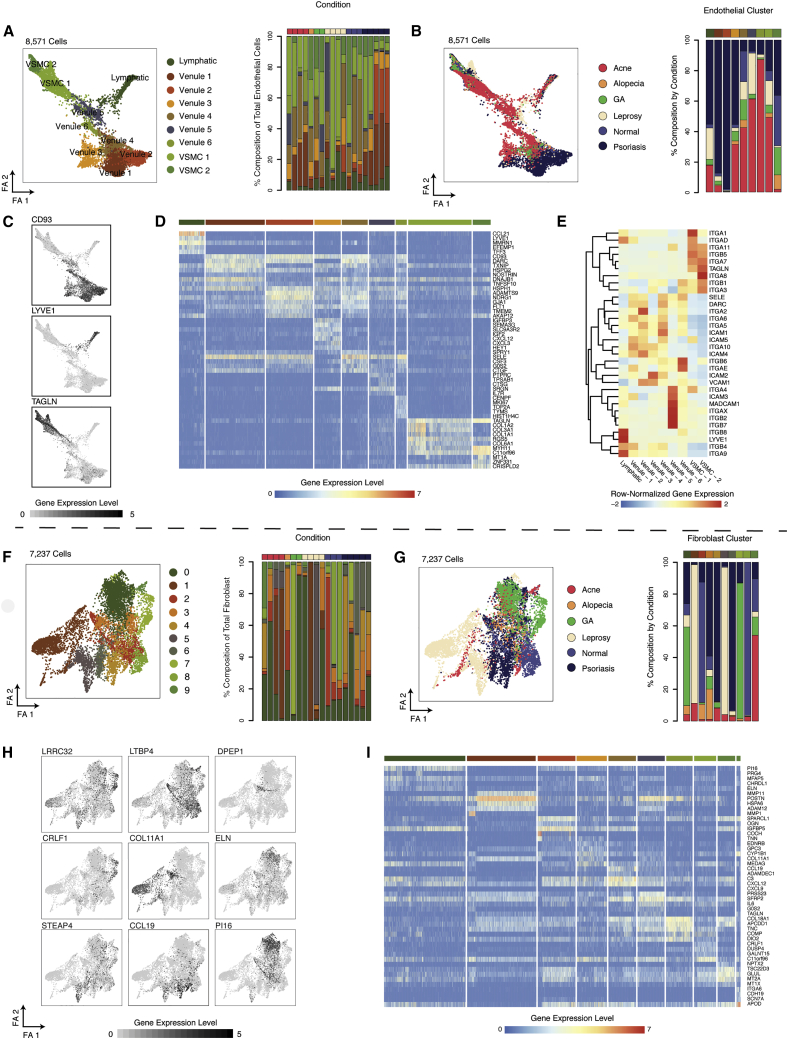
Stromal Cell Diversity (A) Force-directed plots for 8,571 endothelial cells colored by phenotypic sub-cluster (left) and stacked barplot showing the distribution of endothelial phenotypic sub-clusters across samples (right) (Table S3). (B) Force-directed plots for 8,571 endothelial colored by inflammatory skin condition (left) and stacked barplot showing the contribution of each inflammatory skin condition to endothelial phenotypic sub-clusters (right). (C) Force-directed plot colored by normalized expression amounts of genes that mark endothelial cell types: (Left) *CD93*, venules, (Middle) *TAGLN*, arterioles, (Right) *LYVE1*, lymphatics. (D) Heatmap showing patterns of normalized gene expression amounts (log(scaled UMI + 1)) across nine clusters of endothelial cells (Table S3). (E) Heatmap showing row-normalized expression amounts of vascular addressins across phenotypic sub-clusters of endothelial cells. (F) Force-directed plots for 7,237 fibroblasts colored by phenotypic sub-cluster (left) and stacked barplot showing the distribution of fibroblast phenotypic sub-clusters across samples (right) (Table S3). (G) Force-directed plots for 7,237 fibroblasts colored by inflammatory skin condition (left) and stacked barplot showing the contribution of each inflammatory skin condition to fibroblast phenotypic sub-clusters (right). (H) Force-directed graphs highlighting fibroblast cluster-defining genes. (I) Heatmap showing the normalized gene expression levels (log(scaled UMI + 1)) of fibroblast cluster-defining genes (Table S3).

Multiple types of ECs exist within the dermis of the skin. Importantly, DARC^+^ post-capillary venules are the primary site of egress of immune cells from circulation into tissues, which is guided by addressin expression (Schön et al., 2003). Using the improved sensitivity of Seq-Well S^3^, we sought to understand the spectrum of EC diversity and vascular addressin expression across multiple instances of skin inflammation (von Andrian and Mempel, 2003). We identified three primary sub-clusters of dermal ECs defined by distinct expression patterns: VSMCs (*TAGLN*), ECs (*CD93*), and lymphatic ECs (*LYVE1*) (Figures 5A–5C; STAR Methods). Importantly, we found multiple sub-clusters of CD93^+^ ECs across normal and inflamed skin biopsies (Figures 5A–5B; Table S3). For example, we observed a cluster of *DARC*^−^, *CD93*^+^ ECs (venule sub-cluster 3) that displays elevated expression of *SLC9A3R2*, which is involved in endothelial homeostasis (Bhattacharya et al., 2012), and another that is proliferating (venule sub-cluster 6) (Figure 5D). Notably, across sub-populations of CD93^+^ ECs (venule sub-clusters 1–6), we observed varied expression of vascular addressins (Thiriot et al., 2017) (Figure 5E). Among post-capillary venules, we measured broadly elevated expression of *ITGA5*, *ITGA6*, *ICAM2*, and *ITGA2*, whereas VSMCs expressed higher amounts of *ITGA7*, *ITGA8*, and *ITGB5*. Further, we observed the highest expression of *ITGB4*, *ITGB8*, and *ITGA9*, among lymphatic ECs (Figure 5E).

### Altered Dermal Fibroblast Identities in Skin Inflammation

Dermal fibroblasts provide structural support and are the primary source of extracellular matrix components within the skin. Previous studies have reported significant variation among dermal fibroblasts on the basis of their relationship to anatomic features of the skin (Driskell et al., 2013; Driskell and Watt, 2015). In comparison to inflamed biopsies, fibroblasts from normal skin display enrichments in *LTBP4*, *IGFBP5*, and *TCF4* (fibroblast clusters 2 and 8) (Table S3). Consistent with previous single-cell studies of dermal fibroblasts, we observed a sub-population of fibroblasts (fibroblast cluster 3) that expressed *COL11A1*, *DPEP1*, and *RBP4* and is suggested to have a role in connective tissue differentiation (Figure 5H; Table S3) (Tabib et al., 2018)*.*

In GA, we observed two distinct fibroblast populations. Fibroblasts from GA patient 1 (sub-cluster 0) displayed elevated expression of protease inhibitor 16 (*PI16*), which inhibits the function of MMP2 (Hazell et al., 2016), and *ITIH5*, a protease inhibitor important for maintenance of dermal hyaluronic acid that is overexpressed in skin inflammation (Figures 5H–5I; Table S3) (Huth et al., 2015). Fibroblasts from GA patient 2 (sub-cluster 7), meanwhile, expressed elevated amounts of *SPOCK1* (Avg-Log FC: 0.99), *CRLF1* (Avg-Log FC: 1.38), and *COMP* (Avg-Log FC: 1.35), a cartilage protein that is upregulated in matrix-producing fibroblasts after myocardial infarction (Fu et al., 2018).

We also observed distinct fibroblast phenotypes in leprosy infection. Specifically, we found a population of fibroblasts (fibroblast cluster 1) marked by combined expression of *POSTN* (Periostin) and *MMP11*, a marker of fibroblasts in basal cell carcinoma (Micke et al., 2007) (Figure 5I; Table S3). In another leprosy biopsy, we observed a population of pro-inflammatory fibroblasts (fibroblast cluster 5) that expresses elevated amounts of *SFRP2*, *PRSS23*, and *IL6*. Finally, among all 5 psoriasis biopsies, we observed a population of pro-inflammatory fibroblasts (fibroblast cluster 4) marked by elevated expression of *CCL19*, *TNFSF13B* (BAFF), and *CXCL12* (Figures 5H–5I; Table S3).

### Keratinocyte Differentiation Trajectories

**Figure 6:**
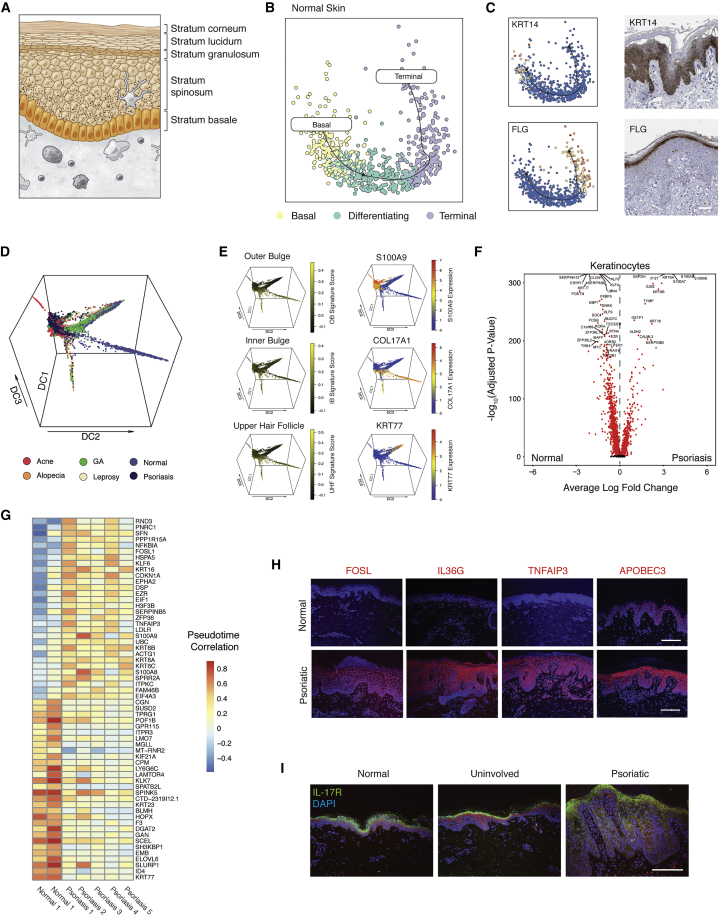
Keratinocyte Differentiation Trajectories (A) Diagram showing the layers of the epidermis and morphologic changes associated with keratinocyte differentiation. (B) t-SNE plot showing differentiation trajectory of keratinocytes from normal skin from basal cells (yellow) through differentiating cells (aqua) and terminal keratinocytes (purple). (C) (Top, left) t-SNE plot of normal keratinocytes colored by *KRT14* expression. Shown at the top right is KRT14 staining from the human protein atlas (Uhlén et al., 2015). Shown on the bottom left is a t-SNE plot of normal keratinocytes colored by *FLG* expression. Shown on the bottom right is FLG staining from the human protein atlas (Uhlén et al., 2015). Scale bars, 50 μm. (D) Diffusion map of 10,777 keratinocytes colored by inflammatory skin condition. Axes correspond to diffusion components 1, 2, and 3. (E) Diffusion map of keratinocytes colored by signatures of hair-follicle-specific gene expression (Joost et al., 2016) (Left: outer bulge, inner bulge, and upper hair follicle) and genes that distinguish basal (*COL17A1*), normal (*KRT77*), and inflamed (*S100A9*) keratinocytes. (F) Volcano plot of genes differentially expressed between psoriatic and normal keratinocytes. Log10-fold change values are shown on the x axis and −log10 adjusted p values are shown on the y axis. (G) Heatmap showing gene-specific Pearson correlation values between diffusion pseudotime and gene expression for two normal skin biopsies and five psoriatic biopsies. (H) (Top) Immunofluorescence staining in normal (above) and psoriatic (below) for FOSL, IL36G, TNFAIP3, and APOBEC3. All images stained for nuclei (DAPI) and gene of interest (red fluorescence). Scale bar, 100 μm. (I). Immunofluorescence staining for IL-17R expression (green) in normal (left), uninvolved (middle), and psoriatic skin (right). Scale bar, 100 μm.

Within the epidermis, KCs undergo a stereotyped differentiation process in which cells acquire altered morphologies and phenotypes as they mature (Figure 6A) (Fuchs, 1990). Using KCs from normal skin, we performed pseudo-temporal analysis to reconstruct the differentiation process of normal epidermal KCs (Figure 6B; STAR Methods) (Saelens et al., 2019). In normal skin, we first identified a population of KCs enriched for expression of *KRT14*, a marker of basal KCs (Figure 5C) (Pellegrini et al., 2001). We then used known patterns of cytokeratin expression to infer localization of KCs along a supervised differentiation trajectory (Figures 6C and S6A) (Ordovas-Montanes et al., 2018). Our trajectory analysis revealed patterns of transcription factor and cytokeratin expression that closely correspond to previously established signatures of KC maturation in both normal skin samples where we recovered enough KCs to perform trajectory analysis (STAR Methods; Figures S6A and S6B) (Cheng et al., 2018). Consistent with immunohistochemical staining from the Human Protein Atlas (Figure 6C) (Uhlén et al., 2015), we found enriched expression of filaggrin (*FLG*), a protein in the outer layers of the epidermis (Sandilands et al., 2009), among keratinocytes that lie at the terminal points in the pseudo-temporal ordering (Figures 6C and S6B).

We next examined patterns of KC differentiation across pathologic conditions and discovered marked deviation in the differentiation trajectory of psoriatic KCs (Figure 6D). We further identified distinct lineages for basal KCs (*COL17A1*) and cells of the hair follicle, where we detected enrichment of published hair follicle signatures (Figure 6E) (Joost et al., 2016). Consistent with previous reports, differential expression analysis revealed significant up-regulation of antimicrobial peptides (*S100A7*, *S100A8*, and *S100A9*) and pro-inflammatory cytokines (*IL36G* and *IL36RN*) in psoriatic KCs (Figures 6E–6F; Table S6) (Li et al., 2014).

Based on increased sensitivity of Seq-Well S^3^ to detect transcription factors observed in peripheral lymphocytes, we hypothesized that our data might enable identification of transcriptional regulators of psoriatic KCs. To identify potential drivers of the psoriatic disease process within the epidermis, we performed differential pseudo-time correlation analysis between psoriatic and normal KCs (STAR Methods). We separately constructed pseudo-time trajectories for normal (n = 2) and psoriatic KCs (n = 5), calculated correlation values between diffusion pseudo-time and gene expression amounts, and examined the difference in correlation values between psoriatic and normal KCs (Figures 6G, S6A, and S6B; Table S6). This uncovered positive correlation of *FOSL1*, an AP-1 transcription factor, with diffusion pseudo-time in psoriatic KCs, implying that *FOSL1* might be aberrantly expressed along the differentiation trajectory of psoriatic KCs. To validate this observation, we performed immunofluorescence staining for FOSL1 protein, and measured increased amounts of FOSL1 in psoriatic skin (Figure 6H; STAR Methods). We validated the distribution of additional genes overexpressed or differentially correlated with diffusion pseudo-time in psoriatic KCs (including *TNFAIP3*, *IL36G*, and *APOBEC3*) at the protein level (Figures 6H and S6A; Table S6; STAR Methods). Finally, we examined the relationship between differential expression and difference in pseudo-time correlation (Figure S6C). Here, we observed no overall relationship between differential expression and differential pseudotime, suggesting a more complicated picture of dysregulated gene expression in psoriatic KCs.

To further define differences in gene expression between normal and psoriatic KCs, we scored the expression amounts of known cytokine response signatures by using a series of reference signature gene lists derived from population RNA-seq of cultured keratinocytes exposed to multiple cytokines including IL-17A, IL-4, IL-13, TNF-α, IFN-α, and IFN-γ (Figure S6D; Table S6; STAR Methods) (Tsoi et al., 2019). Although IL-17 has been previously implicated in the pathogenesis of psoriasis, here we inferred the identity of cells that dominate the IL-17 response, localizing the expression of IL-17 responsive genes to spinous KCs (Chiricozzi et al., 2014). To validate this observation, we performed immunofluorescent staining for IL-17R protein and measured the highest staining within spinous KCs exclusively within psoriatic skin (Figure 6I; STAR Methods).

## Discussion

Here, we present Seq-Well S^3^, a high-throughput and high-fidelity scRNA-seq platform. Through use of a templated second-strand synthesis, S^3^ reclaims cDNA molecules that were successfully reverse transcribed but not labeled with a second oligonucleotide handle through template switching and thus would normally have been lost in common bead-based high-throughput scRNA-seq protocol such as Seq-Well or Drop-Seq. Using Seq-Well S^3^, in relation to Seq-Well v1 (Gierahn et al., 2017), we obtained a 5- to 10-fold increase in the number of unique genes and transcripts captured per cell at similar sequencing depth. Beyond aggregate increases in the number of genes and transcripts recovered per cell, Seq-Well S^3^ facilitated enhanced detection of lineage-defining factors in immune and parenchymal cells—such as transcription factors, cytokines, and cytokine receptors that are often transiently or lowly expressed among lymphocytes. Critically, the Seq-Well S^3^ protocol is easy to integrate into current bead-based RNA-seq platforms, such as Drop-Seq (Macosko et al., 2015) and spatial RNA-seq platforms like Slide-Seq (Rodriques et al., 2019), making it broadly useful for the single-cell community.

Increases in the sensitivity of gene and transcript detection are increasingly important as single-cell atlasing efforts shift from defining large differences between cell types within normal tissues to characterizing subtle alterations to cell states in disease. Although a number of high-throughput methods have been developed, each fills a specific role. For example, the methods that rely on split-pool barcoding of cells or nuclei, such as sci-RNA-seq (Cao et al., 2017) or SPLiT-seq (Rosenberg et al., 2018), can examine tens to hundreds of thousands of cells at once, enabling characterization of model organisms or complex chemical screens, but work best with certain cell types and are associated with substantial cell and transcript loss (Ding et al., 2020), limiting their applicability to precious clinical samples. Commercial reverse-emulsion droplet-based methods, like 10x, overcome these inefficiencies and provide streamlined workflows, but add substantial cost, both with respect to consumables and instrumentation, and constrain where and how samples can be run. Although the state-of-the-art continues to evolve rapidly, Seq-Well S^3^ provides a competitive alternative that is uniquely suited for clinical studies because of its efficiency, simplicity, compatibility with fragile cells, limited peripherals, flexible stopping points (post-reverse transcription), ability to be parallelized (up to 20 samples in a one-day experiment), high degree of technical reproducibility, and open molecular biology (which enables targeted enrichment of molecules of interest) (Tu et al., 2019; van Galen et al., 2019).

Cost is a key factor in the selection of scRNA-seq methods. Prior to sequencing, the cost of Seq-Well S^3^ is significantly less than that of commercial scRNA-seq platforms. Ignoring differences in instrumentation requirements and their associated costs (over an order of magnitude less for Seq-Well S^3^), Seq-Well S^3^ and 10x v3 required approximately the same amount of money to obtain 50,000 aligned reads for the PBMCs presented here (note: alignment rates and exact figures will change as a function sample type and pre-processing). Although this price exceeds that for the cell-based split-pool methods, at present, the fidelity is higher. Moreover, the throughput and price of processing for both can be further reduced through sample multiplexing, driving sequencing, rather than sample preparation (especially for Seq-Well S^3^), to dominate costs (McGinnis et al., 2019; Stoeckius et al., 2018).

The increased sensitivity of gene detection and transcript capture afforded by Seq-Well S^3^ enhances the strength of the inferences and hypotheses that can be generated when examining the cellular and molecular features of disease pathophysiology by using scRNA-seq. To date, single-cell analyses of healthy and diseased human skin have revealed heterogeneity among immune and parenchymal cell types (Cheng et al., 2018; He et al., 2020; Kim et al., 2020; Tabib et al., 2018). However, these studies have largely focused on a single cell type or disease. Here, we examined the cellular composition of normal skin as well as alterations in cellular phenotypes associated with multiple inflammatory skin conditions, including acne, alopecia areata, GA, leprosy, and psoriasis. Our results provide a draft atlas of human skin inflammation, creating a compendium of cell types and states for the broader research community (Angelo et al., 2014) while providing insights into putative mechanisms and the cellular localization of previously appreciated and unknown responses to specific inflammatory mediators in immunologic skin conditions.

We detected numerous T cell phenotypes and sub-phenotypes across inflammatory skin conditions by using Seq-Well S^3^. Among multiple psoriasis biopsies, we observed over-representation of Tregs, dysfunctional *NR4A1*-expressing T cells, and senescent *SESN3*^+^ T cells, which could reflect a role for broader T cell dysfunction in disease pathology (Šahmatova et al., 2017). Meanwhile, in leprosy, we identified a population of T cells enriched for expression of *ROR-γT*, consistent with a suggested role for Th-17 cells in bacterial control (Saini et al., 2013; Saini et al., 2016). However, this population was only detected in two of four biopsies, including one patient undergoing a reversal reaction, which implies a variable role for Th-17 cells across patients and forms of leprosy. Further, using Seq-Well S^3^, we observed improved TCR recovery in PBMCs. By examining V region sharing, we found the highest enrichment of TCR sequences in leprosy and acne biopsies, which suggests an important role for antigen-specific T cell responses in these diseases consistent with recent data from acne (Shao et al., 2020). However, further experimentation is needed to more fully understand the relationship between TCR clonality and T cell phenotype in skin inflammation.

In psoriasis, T cells are thought to be a primary driver of inflammation, and DCs play a central role in the recruitment and polarization of T cells that contribute to the hyperproliferation of KCs in the disease (Lowes et al., 2014). Across five patients with psoriasis, we reported a sub-cluster of DCs (*IRF4*^+^ cDC2) that displays elevated expression of *CCL17*, *CCL22*, and *IL12B*, markers of cDC2s that have recently been shown to drive psoriatic inflammation in mice and humans through the recruitment of inflammatory T cells (Kim et al., 2018; Zaba et al., 2010). We further observed a population of fibroblasts in psoriasis that express *CCL19*, *TNFSF13B* (BAFF), and *CXCL12*. Notably, expression of *CCL19* and *BAFF* by synovial fibroblasts has been implicated in the progression of rheumatoid arthritis (Pickens et al., 2011; Reyes et al., 2008), but their relevance to psoriasis has yet to be described and will require further exploration.

Among ECs, we identified two clusters marked by expression of *SLC9A3R2*, a marker of endothelial homeostasis, and a signature of proliferation (venule clusters 3 and 4) (Bhattacharya et al., 2012). These proliferating EC clusters were enriched in acne, which is thought to arise in response to infection with *P. acnes*, resulting in the formation of lesions that resemble a wound after eruption of the hair follicle into the dermis (Beylot et al., 2014). Our findings suggest a prominent role for proliferative angiogenesis in the wound healing response that is seen in acne (Holland et al., 2004).

Differentiated KCs have been suggested to be the primary responders to IL-17A in psoriasis on the basis of previous studies by using *in vitro* KC systems, given larger effect sizes in differentiated compared with monolayer KCs (Chiricozzi et al., 2014). By cross-analyzing the data generated here against an IL-17 response signature in KCs, we have shown that IL-17 responses are observed in KCs from all layers of the epidermis, but that these responses are stronger in KCs derived from more differentiated layers of the psoriatic epidermis. By more precisely localizing IL-17 responses in psoriasis, our data might help to inform improved treatment strategies.

Beyond describing what can be gleaned about the cellular and molecular deviations associated with any one disease, we distinguished expression patterns associated with multiple diseases by looking across different inflammatory skin conditions to reveal common and unique response features. For example, our profiling uncovered a diverse group of cytotoxic cells that contains NK cells, γδ T cells, and a sub-cluster of immature cytotoxic T cells that are derived primarily from leprosy and GA samples, which suggests common T cell programming between two forms of granulomatous inflammation. In GA, we observed multiple, distinct fibroblast populations that segregate between patients: one expressing cartilage associated proteins (e.g., *COMP*) and another expressing protease inhibitors and matrix metalloproteinases, respectively, which might reflect different forms of inflammation in distinct types of granuloma annulare (Piette and Rosenbach, 2016). In leprosy, meanwhile, we also detected a unique macrophage population defined by expression of extracellular proteases, as well as elevated expression of IFN-γ associated transcriptional programs in LCs, which might reflect their role in response to infection (Pinheiro et al., 2018). Identifying how common cellular phenotypes affect disease pathophysiology in distinct cellular ecosystems is a critical avenue for future inquiry.

By charting the spectrum of skin inflammation at single-cell resolution, we have generated a resource that will serve as a reference for future inquiry into cutaneous biology. Among many of the cell types and states we identified, we found expression features that are shared across diseases, suggesting potentially common targetable biology; in others, our work revealed unique features, potentially associated with disease trajectory, for further inquiry. In future studies, Seq-Well S^3^ will enable enhanced characterization of immune and parenchymal phenotypes in various types of inflammation across tissue compartments and how their interactions influence the development of human disease to reveal actionable therapeutic and prophylactic axes.

## Limitations of Study

Although Seq-Well S^3^ results in improved capture of transcripts from each cell, there are important limitations associated with the method and the results presented here. First, in Seq-Well S^3^, the size of the cDNAs after second-strand synthesis was shorter than that obtained in Seq-Well or Drop-Seq. This decreases the utility of Seq-Well S^3^ for certain downstream applications that seek information from full-length transcripts or from their 5′ ends. Meanwhile, although we uncover multiple cellular phenotypes across inflammatory skin conditions, we were limited in our ability to distinguish inter-individual variation from disease-specific biology because of low numbers of samples per condition. Here, future studies with larger cohort sizes and/or matched unaffected skin samples from the same individual will be needed to resolve disease- from individual-specific features. Further, many of our findings are based on mRNA expression and correlative. Follow up experiments using protein detection and perturbation strategies will be necessary to corroborate significance. Nevertheless, the increased sensitivity of gene detection and transcript capture afforded by S^3^ enhances the strength of the inferences that can be drawn from these types of single-cell data, as evidenced by the range of immune, stromal, and parenchymal cell states we describe across a spectrum of human skin inflammation.

## STAR★Methods

### Key Resources Table

**Table:** 

REAGENT or RESOURCE	SOURCE	IDENTIFIER
**Antibodies**		

IL-17RA	Lifespan Bioscience	Cat#LS-C359381
IL-17RC	Lifespan Bioscience	Cat#LS-400522
FOSL	Boster	Cat# A03927
IL-36G	Santa Cruz Biotechnology	Cat#Sc-80056; RRID: AB_2124893
TNFAIP3	Abcam	Cat#Ab74037; RRID: AB_1524499
APOBEC3A	Lifespan Bioscience	Cat#LS-C98892-400

**Biological Samples**		

Skin Biopsies	UCLA	N/A
Skin Biopsies	University of Michigan	N/A
PBMCs	MGH	N/A

**Chemicals, Peptides, and Recombinant Proteins**		

Maxima H-RT and Buffer	ThermoFisher Scientific	Cat#EP0751
dNTPs	New England Biolabs	Cat#N0447L
Polyethylene Glycol 8000	Fisher Scientific	Cat#BP233-1
SUPERase^∗^In RNase inhibitor	ThermoFisher Scientific	Cat#AM2696
Exonuclease I and Buffer	New England Biolabs	Cat#M0293S
1M Tris-HCl, pH 8.0	ThermoFisher Scientific	Cat#15568025
Klenow Fragment (3’®5’ exo-)	New England Biolabs	Cat#M0212L
KAPA 2x HiFi HotStart PCR mix	Kapa Biosystems	Cat#KK2602
Nextera XT Kit	Illumina, Inc	Cat#FC-131-1096
UltraPure DNase/Rnase-Free Distilled Water	ThermoFisher Scientific	Cat#10977015
TWEEN 20	Fisher Scientific	Cat#BP337-100
Sodium Dodecyl Sulfate (SDS) Solution	Sigma	Cat#71736-100mL
TE Buffer	ThermoFisher Scientific	Cat#12090015

**Critical Commercial Assays**		

mRNA Capture Beads	Chemgenes Corp.	Cat#MACOSKO-2011-10B
KAPA 2x HiFi HotStart PCR mix	Kapa Biosystems	Cat#KK2602
NextSeq500 (75 cycles)	Illumina	Cat#20024906
Nova-Seq S2 (100 cycles)	Illumina	Cat#20028316

**Deposited Data**		

Raw and Processed data	GEO	GEO: GSE150672

**Experimental Models: Cell Lines**		

HEK293	ATCC	CRL-1573
NIH/3T3s	ATCC	CRL-1658

**Oligonucleotides**		

Template-Switching Oligo: AAGCAGTGGTATCAACGCAG AG TGAATrGrGrG	This Paper	N/A
SMART PCR Primer: AAGCAGTGGTATCAACGCAGAGT	This Paper	N/A
S^3^ Randomer:AAGCAGTGGTAT CAACGCAGAGTGANNNGGNNNB	This Paper	N/A
P5-SMART Hybrid Oligo: AATGATACGGCGACCACCG AGATCTACACGCCTGTCCGC G-GAAGCAG TGGTA TCAACGCAGAGT^∗^A^∗^C	This Paper	N/A
Custom Read 1 Primer: GCCTGTCCGCGGAAGC AGTGGTATCAACGCAGAGTAC	This Paper	N/A

**Software and Algorithms**		

Seurat	Satija et al. 2015	http://satijalab.org/seurat/
SCANPY	Wolf et al. 2018	http://github.com/theislab/Scanpy

### Resource Availability

#### Lead Contact

Additional information and requests for resources and reagents should be directed to the Lead Contact: Alex K. Shalek (shalek@mit.edu).

#### Materials Availability

All unique reagents generated are listed in the key resources table along with the supplemental protocol. Additional requests for resources and reagents can be directed to the Lead Contact.

#### Data and Code Availability

Raw and processed data are available on the gene expression omnibus (GEO) in GSE150672. Processed data are further available in an interactive format as part of the Alexandria Project (https://singlecell.broadinstitute.org/single_cell?scpbr=the-alexandria-project). Additional code is available upon request from the Lead Contact.

### Experimental Model and Subject Details

#### Cell Lines

HEK293 and NIH-3T3 cell lines used in species mixing experiments were obtained from ATCC. Cell lines were cultured in DMEM supplemented with 10% FBS at 37C with 5% CO2.

#### PBMCs

Peripheral blood mononuclear cells (PBMCs) used in optimization and comparison experiments were obtained from Massachusetts General Hospital. Aliquots of 1.0x10^6^ PBMCs were frozen in 90% FBS with 10% DMSO and thawed prior to use in experiments.

#### Human Skin Samples

Skin biopsies were obtained from a total of 16 patients at the University of California, Los Angeles and University of Southern California Hansen’s Clinic, while an additional 3 samples were obtained from the University of Michigan. Informed written consent was obtained from human subjects under a protocol approved by the institutional review boards of the University of Michigan and University of California Los Angeles (UCLA). This study was conducted according to the Declaration of Helsinki Principles.

### Method Details

#### Skin Biopsy Processing

For each sample, a 4-mm punch biopsy was obtained following local anesthesia and was placed immediately into 10 mL of RPMI on ice. Initially, skin biopsies were incubated in 5 mL of a 0.4% Dispase II solution (Roche Inc.) at 37°C for 1 hour with vigorous shaking. The dermis and epidermis were then carefully separated using forceps and transferred to separate tubes for additional processing. Epidermal samples were placed in 3 mL of 0.25% Trypsin and 10 U/mL DNAse for 30 minutes at 37°C. Trypsin was neutralized with 3 mL of fetal calf serum (FCS), and the tissue was passed through a 70-micron nylon cell strainer which was washed with 5 mL of RPMI. Epidermal cells were then pelleted at 300xg for 10 minutes and counted. Dermal samples were minced with a scalpel and incubated in a solution of 0.4% collagenase 2 and 10 U/mL DNAse for 2 hours at 37°C with agitation. The cell suspension was passed through a 70-micron cell strainer and washed with 5 mL of RPMI. Cells were pelleted at 300 xg for 10 minutes, resuspended in 1 mL of RPMI and counted. MACS enrichment for CD1A^+^ cells was performed for epidermal fractions from biopsies from normal skin 1 and leprosy 1.

#### Single-Cell Processing Pipeline

We utilized Seq-Well, a massively parallel, low-input scRNA-seq platform for clinical samples, to capture the transcriptome of single cells. A complete, updated protocol for Seq-Well S^3^ is included as a Supplementary Protocol and is hosted on the Shalek Lab website (www.shaleklab.com). Briefly, 10-15,000 cells were loaded onto a functionalized-polydimethylsiloxane (PDMS) array preloaded with uniquely barcoded mRNA capture beads (Chemgenes; MACOSKO-2011-10). After cells had settled into wells, the array was then sealed with a hydroxylated polycarbonate membrane with a pore size of 10 nm, facilitating buffer exchange while confining biological molecules within each well. Following membrane-sealing, subsequent buffer exchange permits cell lysis, mRNA transcript hybridization to beads, and bead removal before proceeding with reverse transcription. The obtained bead-bound cDNA product then underwent Exonuclease I treatment (New England Biolabs; M0293M) to remove excess primer before proceeding with second-strand synthesis.

#### Templated Second-Strand Synthesis

Following Exonuclease I treatment, beads were washed once with 500 μL of a TE-SDS (0.5% SDS) solution, and twice in 500 μL of a TE-Tween (0.01% Tween) solution. After the second TE-TW wash the beads were solvated with 500 μL of 0.1 M NaOH and mixed for 5 minutes at room temperature using an end-over-end rotator with intermittent agitation to denature the mRNA-cDNA hybrid product on the bead. Following denaturing, the NaOH was removed and beads were washed once with 1 M TE, and then combined with a mastermix consisting of 40 μL 5x maxima RT buffer, 80 μL 30% PEG8000 solution, 20 μL 10mM dNTPs, 2 μL 1 mM dn-SMART oligo, 5 μL Klenow Exo-, and 53 μl of DI ultrapure water. Second-strand synthesis was carried out by incubating the beads for 1 hour at 37°C with end-over-end rotation and intermittent agitation. Following incubation, beads were sequentially washed twice with 0.5 mL of TE buffer with 0.01% Tween 20, and once with 0.5 mL of TE. Immediately prior to PCR amplification, beads were washed once with 0.5 mL of water and resuspended in 0.5 mL of water.

#### PCR Amplification

After second-strand synthesis, PCR amplification was performed using KAPA HiFi PCR Mix (Kapa Biosystems KK2602). Specifically, a 40 μL PCR Mastermix consisting of 25 μL of KAPA 5X Mastermix, 0.4 μL of 100 μM ISPCR oligo, and 14.6 μL of nuclease-free water was combined with 2,000 beads per reaction. For each sample, the total number of PCR reactions performed varied based on the number of beads recovered following second-strand synthesis. PCR amplification was performed using the following cycling conditions: an initial denaturation at 95°C for 3 minutes, then 4 cycles of 98°C for 20 seconds, 65°C for 45 seconds, and 72°C for 3 minutes, followed by 9-12 cycles of 98°C or 20 seconds, 67°C or 20 second, and 72°C for 3 minutes, and then a final extension of 72°C for 5 minutes. Following PCR amplification, WTA products were isolated through two rounds of SPRI purification using Ampure Spri beads (Beckman Coulter, Inc.) at both 0.6x and 0.8x volumetric ratio and quantified using a Qubit.

#### Optimization of Second-Strand Synthesis

A series of experiments was performed to validate the performance of the second-strand synthesis protocol relative other techniques. Species-mixing and PBMC profiling experiments were performedfor the comparison of the Seq-Well protocol with and without second-strand synthesis. For species-mixing experiments, we applied a mixture of 5,000 HEK293 and 5,000 NIH-3T3 cells to a loaded Seq-Well device, while for PBMC comparisons, a total of 10,000 PBMCs were applied to Seq-Well devices. In optimization experiments, PBMCs were thawed and immediately loaded directly onto Seq-Well devices without stimulation. Following bead removal, beads were split into separate reverse transcription reactions with and without the template-switching oligo. After reverse transcription and ExoI treatment, beads for each comparison were processed separately with and without the second-strand synthesis protocol.

A series of optimization experiments were performed to validate the effectiveness of Seq-Well Sˆ3. Here, a series of control experiments were performed using beads from a single Seq-Well array loaded with 10,000 PBMCs. For each array the beads were split into six equal fractions and performed the following controls: (1) PCR amplification without the use of second-strand synthesis, (2) random second-strand synthesis followed by PCR amplification, (3) no template switching oligo without the use of second-strand synthesis, (4) no template switching oligo with subsequent random second-strand synthesis, (5) heat inactivation of the reverse transcription reagent without the use of second-strand synthesis, (6) heat inactivation of the reverse transcription reagent followed by random second-strand synthesis. Following PCR amplification, products were obtained from all conditions with the exception of Condition 3 (Seq-Well V1/ No TSO), which did not yield appreciable WTA product.

#### CD4^+^ T Cell Comparisons of 10x Genomics, Seq-Well S^3^, and Smart-Seq2

Human PBMC were thawed and rested overnight. Cells were stimulated for 18 hours by adding aCD3 (UCHT1) and aCD28 (CD28.2) antibodies to the bulk PBMC culture at a concentration of 1 mg/mL and 5 mg/mL, respectively, and CD4^+^ T cells were enriched following stimulation using magnetic negative selection (Stemcell Technologies). Following isolation, T cells were stained with calcein violet live stain (Thermo), Sytox dead stain (Thermo), and aCD45-AF647 (HI30) antibody at 4°C for 30 minutes. After two washes, aliquots of the cells were placed on ice and sorted directly into RLT buffer using a Sony SH800S Sorter for Smart-Seq2 processing and another unstained sample for 10x Chromium analysis. Once the cells were delivered, a third aliquot was loaded onto a Seq-Well array. Single-cell libraries were generated using the Smart-Seq 2, 10x v2, and Seq-Well S^3^ protocols. For comparison experiments between 10x v3 and Seq-Well S^3^ human PBMC were thawed and rested overnight. Aliquots of cells were washed, counted and placed on ice prior to processing with 10x v3 and Seq-Well S^3^ protocols.

#### Sequencing Library Preparation

A total of 1 ng of WTA product at a concentration of 0.2 ng/μL was combined with 10 μL of Buffer TD and 5 μL of Buffer ATM and incubated at 55°C for 5 minutes. Following tagmentation, 5 μL of Buffer NT was added and incubated at room temperature for 5 minutes to neutralize the reaction. A total of 8 μL of nuclease-free water, 15 μL of buffer NPM, 1 μL of Custom P5 hybrid Oligo, and 1 μL of N700 Index oligo were combined and PCR amplification was performed using the following cycling conditions: an initial denaturation of 95°C for 30 seconds, then 12 cycles of 95°C for 10 seconds, 55°C for 30 seconds, and 72°C for 30 seconds, followed by a final extension of 72°C for 5 minutes. PCR products were isolated through two rounds of SPRI purification (0.6x and 0.8x volumetric ratios) and quantified using a Qubit. Library size distributions were determined using an Agilent Bioanalyzer D1000 High Sensitivity Screen tape.

#### DNA Sequencing and Alignment of PBMC Optimization samples

PBMC optimization experiments were all sequenced on NextSeq500 75 cycle kits. Sequencing read alignment was performed using version 1 of the Drop-seq pipeline (Macosko et al., 2015). NextSeq runs were loaded at a final concentration of 2.2 pM along with the custom read 1 primer using NextSeq 550 v2 sequencing kits at the Ragon Institute. Briefly, for each sequencing run, raw sequencing reads were converted from bcl files to FASTQs using bcl2fastq and demultiplexed using Nextera N700 indices that corresponded to individual samples. Demultiplexed FASTQs were then aligned using an implementation of DropSeqTools v1.0 maintained by the Broad Institute for data analysis, and aligned to the Hg19 genome using standard parameters. Individual reads were tagged with a 12-bp barcode and 8-bp unique molecular identifier (UMI) contained in Read 1 of each sequencing fragment. Following alignment, aligned read 2 sequences were grouped by the 12-bp cell barcodes and subsequently collapsed by the 8-bp UMI for digital gene expression (DGE) matrix extraction and generation.

#### Tissue Immunofluorescence Staining

Formalin fixed, paraffin-embedded tissue slides obtained from psoriasis patients and normal controls were heated for 30 min at 60°C, rehydrated, and epitope retrieved with Tris-EDTA, pH 6. Slides were blocked, incubated with primary antibody APOBEC3 (LS-C98892-400; Lifespan bioscience), FOSL (A03927; Boster), IL-36G (sc-80056; Santa Cruz Biotechnology), TNFAIP3 (ab74037, Abcam), IL-17RC (LS-C400522, Lifespan bioscience), and IL-17RA (LS-C359381, Lifespan bioscience) overnight at 4 °C. Slides were then washed and incubated with Donkey anti-Rabbit IgG 594, Donkey anti-Mouse IgG 488, or Donkey anti-Rat IgG 594 (all from Invitrogen) for 1 h at room temperature. Slides were washed and prepared in mounting medium with 4',6-diamidino-2-phenylindole (DAPI) (VECTASHIELD Antifade Mounting Medium with DAPI, H-1200, VECTOR). Images were acquired using Zeiss Axioskop 2 microscope and analyzed by SPOT software 5.1. Images presented are representative of at least three experiments from separate donors.

### Quantification and Statistical Analysis

#### PBMC Comparison Experiments

Unaligned sequencing reads from 10x genomics and Seq-Well S^3^ were downsampled to an average sequencing depth of 38,000 reads per cell. Specifically, downsampling was performed on Seq-Well S^3^ to match the sequencing depth of 10x Genomics v2. For each data set, variable gene identification was separately performed (Seq-Well S^3^, 856 variable genes and 10x Genomics v2, 516 genes). Principal component analysis was performed, and the first 20 principal components were use for t-SNE dimensionality reduction and cluster identification and discovered clusters. The proportion of cell types recovered between Seq-Well S^3^ and 10x v2 was compared using a Chi-Square test.

Differences in aggregate gene detection and transcript capture were separately examined within each cell type between Seq-Well S^3^ and 10x v2 using a Mann-Whitney U Test. A Lilliefors test was used to assess normality of the distribution of genes and UMIs for each technique. The linear relationship between the number of UMIs captured and aligned sequencing reads was calculated as a measure of library complexity. Specifically, the slope of the regression line between the number of UMIs against the number of aligned reads was calculated for each PBMC cell type for each technique. Library complexity was compared using a multivariable linear regression model in which the number of transcripts per cell was modeled as follows: nUMI ∼ Intercept + B1^∗^nReads + B2^∗^Technique + B3^∗^nReads^∗^Technique. Statistical significance of the difference in slope (i.e. library complexity) was determined based on p values for the interaction term B3^∗^nReads^∗^Technique, the magnitude and significance of which correspond to a difference in slope (i.e. library complexity or the number of UMIs per aligned read). For example, in a library of low-complexity, application of additional sequencing reads might result in detection of a new transcript in every 20th aligned read (i.e. slope = 0.05). Conversely, a library of high complexity might result in detection of a new transcript with every 4 aligned reads (i.e. slope = 0.25). These comparisons were performed on libraries that have been sequenced or down-sampled to similar depths as over-sequencing can alter the relative perception of differences in library complexity. Here, libraries that have been “over-sequenced” will appear to have lower complexity because unique molecular identifiers will eventually accumulate additional reads upon saturation.

Additional comparisons were performed between Seq-Well S^3^ and 10x v3 on a per cell type basis. Downsampling was performed within each cell type, for both Seq-Well S^3^ and 10x v3, to the same number of aligned reads. The tagged aligned BAMs were first split by cell types, and Samtools (Li et al., 2009) was used to sort and down-sample each cell-type-specific BAM to the appropriate read depth. Picard-Tools and Drop-seq tools were used to extract down-sampled barcodes and generate expression matrices for both aligned reads and unique molecular identifier counts after separate down-sampling for each cell type within each technique.

#### Comparison of Gene Detection Rates

For each cell type cluster, the rate of detection for each gene was calculated as the proportion of cells with a non-zero expression value. Gene detection rates were separately calculated across CD4^+^ T cells, B cells, CD8^+^/NK cells, and monocytes for both Seq-Well S^3^ and 10x Genomics v2. For comparisons between Seq-Well S^3^ and 10x Genomics v3, gene detection rates were separately calculated within 8 cell types. For comparisons of relationship between gene-detection rates and overall expression levels, the expression level of individual genes was calculated as the average normalized expression value within each cell type for all cells identified in both Seq-Well S^3^ and 10x v2 data. Statistical significance of differences in gene detection frequencies was assessed using a chi-square test using the number of cells in which a given gene had a non-zero expression values for each technique.

#### Sequencing and Alignment of Skin Samples

Sequencing read alignment was performed using version 2 of the Drop-seq pipeline previously described in Macosko et al. Briefly, for each Nova-Seq sequencing run, raw sequencing reads were converted from bcl files to FASTQs using bcl2fastq based on Nextera N700 indices that corresponded to individual samples. Demultiplexed FASTQs were then aligned to the Hg19 genome using STAR and the DropSeq Pipeline on a cloud-computing platform maintained by the Broad Institute. Individual reads were tagged with a 12-bp barcode and 8-bp unique molecular identifier (UMI) contained in Read 1 of each sequencing fragment. Following alignment, reads were grouped by the 12-bp cell barcodes and subsequently collapsed by the 8-bp UMI for digital gene expression (DGE) matrix extraction and generation.

#### Cell Quality Filtering

Cells were initially filtered on the basis of gene detection (> 500 genes per cell) and transcript detection (> 700 UMIs per cell) for inclusion in downstream analysis. Cells with fractional representation of mitochondrial genes greater than 40% were excluded. To account for potential transcript spreading, any duplicated or hamming=1 barcodes among samples sequenced on the same Nova-Seq runs were removed. For each sample, variable gene identification was separately performed and 30 principal components were calculated. Within each sample, jackstraw simulations were used to identify significant principal components that were then used to perform t-SNE dimensionality reduction and clustering for each sample using only significant principal components. Within each sample, clusters defined exclusively by mitochondrial gene expression, indicative of low-quality cells, were removed from downstream analysis.

#### Removal of Ambient RNA Contamination

Correction for ambient RNA contamination was performed within each sample using SoupX (Young and Behjati, 2018). Appropriate UMI thresholds for background contamination were determined using EmptyDrops (Lun et al., 2019) by calculating the likelihood that barcodes selected at UMI thresholds between 30 and 100 UMIs per barcode represent cells and selected the UMI threshold in which the distribution of likelihood most closely approximated a uniform distribution. An array-specific ‘soup’ profile was generated among barcodes below the UMI threshold. To calculate estimated per-cell contamination fractions, we manually selected genes observed to be bimodally expressed across cells, which suggests that these genes are predominantly expressed in a single cell type, but are observed at low-levels in other cell types for which endogenous expression would not be expected. For each array, individual transcripts were sequentially removed from each single-cell transcriptome until the probability of subsequent transcripts being soup-derived was less than 0.5 to generate a background-corrected UMI matrix for each Seq-Well S^3^ array.

#### Doublet Removal

Doublet removal was performed for each array individually using DoubletFinder (McGinnis et al., 2019). For each array, the expected doublet rate was estimated based on the cell loading density. A total of 20,000 cells were loaded to a loaded Seq-Well device containing 85,000 wells, which resulted in an expected doublet rate of 2.37%. For each array, pseudo-doublets were generated using the following parameter values in DoubletFinder: proportion.artificial = 0.25 and proportion.NN = 0.01. Cells were identified as doublets based on their rank order in the distribution of the proportion of artificial nearest neighbors (pANN) by selecting the pANN value for the cell at the expected doublet percentile and used the corresponding pANN value as a threshold to remove additional cells with pANN greater than or equal to this value.

#### Analysis of Combined Skin Dataset

Variable gene identification and dimensionality reduction to identify 38 cell type clusters across 49,373 cells using Louvain clustering (resolution = 1.75). Cluster-defining genes were identified within each cluster by performing a Wilcox test in Seurat (Satija et al., 2015) and used to identify cell types. An initial round of dimensionality reduction and cluster identification was performed among cell types used in subsequent analysis (i.e. T cells, myeloid cells, B and plasma cells, endothelial cells, fibroblasts, and keratinocytes). Based on the initial sub-clustering results for each cell type, sub-clusters defined by residual contamination not corrected for by SoupX background correction and doublet filtering were removed. In total, 11,099 cells from sub-clusters defined by residual contamination were removed: 1,471 from the T cell sub-analysis, 497 from the myeloid sub-analysis, 2,444 from the endothelial sub-analysis, 2,512 from the fibroblast sub-analysis, and 4,175 from the keratinocyte sub-analysis.

After this stringent quality control filtering step, a total of 38,274 cells were included in downstream analysis of the atlas of skin inflammation. Variable gene identification and identified 5,897 genes as variably expressed. UMAP dimensionality reduction was performed among 5,897 variably expressed and a total of 35 cell type clusters were identified using Louvain clustering (Resolution = 1.5) in Scanpy (Wolf et al., 2018). Hierarchical clustering was performed across 35 cell type clusters using a gene set composed of the top 25 cluster-defining genes from each cluster. Average gene expression values within each across the 522 unique cluster-defining genes was used to perform hierarchical clustering. A dendrogram was generated to display the similarity of clusters, and the observed relationships were used to inform rational combination of related cell type clusters for combined analysis. Cell type assignments were assigned through a combination of literature-based assessment of expression signatures and manual curation. Validation of cell type manual identification based on the combination of literature and manual curation was performed by automated cell type classification using SingleR (Aran et al., 2019). Here, 38,274 cells were classified using the blueprint encode reference dataset, and ell types assigned by SingleR were compared to the manually assigned cell type classifications.

#### Identification of T cell Sub-Clusters

Among the 4,943 T cells identified in the total dataset, 5,574 variable genes were identified and used to construct a force-directed graph and to perform Louvain clustering (resolution = 0.8). Cell type identities for nine T cell sub-clusters were established by examining the expression of known marker genes corresponding to CD4^+^ T helper and CD8^+^ T cell subsets. Further, T cell signatures were compared to previously identified signatures in SaVant (Lopez et al., 2017). To further define variation, additional sub-analyses were performed among both CD8^+^ T cells and NK-Cytotoxic cells to generate separate UMAP dimensionality reduction using a total of 5 principal components calculated across variable genes using Seurat. For CD8^+^ T cells, sub-clustering was performed using a resolution of 0.3, while a resolution of 0.6 was used for sub-clustering analysis for NK-Cytotoxic T cells.

#### T cell Receptor Detection and Clonal Expansion

The detection rates for TCR α and β (Constant, V and J genes) were examined among CD4^+^ T cells from experiments performed on PBMCs using the Seq-Well v1, Seq-Well S^3^ protocol and 10x v2. Specifically, detection of constant genes (e.g. *TRAC* and *TRBC2*) and variable genes (e.g. TRAV/TRBV) was determined by non-zero values for either genes for α and β constant genes or any TRAV/TRBV gene, respectively. Detection rates in PBMCs were calculated across multiple sequencing depths: <5,000, 5,000-25,000, 25-000-100,000, and > 100,000 aligned reads per cell.

The rate of TCR detection was identified across 2,908 T cells obtained from human skin biopsies. Conservation of V gene sequence was used as a proxy for clonal expansion among skin T cells. V gene usage for each T cell was established by identified the V gene (TRAV or TRBV) with the highest expression level. The distribution of TRAV and TRBV genes within each sample to identify potential clonal expansions. The gini coefficient and the Shannon divergence were calculated for TRAV and TRBV sequences within each sample to identify over-represented TRAV/TRBV sequences.

#### Identification of Myeloid Heterogeneity

Sub-analysis was performed among myeloid populations (i.e. dendritic cells, macrophages, mast cells, and Langerhans cells) identified in global analysis of 38,274 total cells. Using a combined dataset of 5,010 myeloid cells, variable gene identification and dimensionality reduction was performed in Scanpy. A force-directed graph was constructed across 6,599 variable genes and Louvain clustering (resolution = 0.80) was performed, and we obtained 10 sub-clusters of myeloid cells.

To understand differences in Langerhans cells in normal skin, differential expression analysis was performed within each cluster of Langerhans cells. Differential expression was performed between Langerhans cells from Myeloid cluster 8 between normal and leprosy skin biopsies. Gene-set enrichment analysis was performed among genes upregulated in Langerhan’s cells from leprosy samples in comparison to signatures contained in the MSigDb database.

UMAP dimensionality reduction and Louvain clustering (resolution = 0.45) were performed among 951 dendritic cells. Cluster-defining genes were identified within each of 5 sub-groupings of dendritic cells by performing a Wilcox test in Seurat. Comparisons to published signatures of dendritic cell phenotypes were performed to understand how dendritic cells related to previous findings (Dutertre et al., 2019; Villani et al., 2017). Specifically, expression scores were generated using the top 10 genes using the AddModuleScore function in Seurat. Significance of cluster enrichment was determined by performing 1,000 permutations in which cell and signature score identifiers were randomly re-assigned.

#### Identification of Endothelial Heterogeneity

Sub-analysis was performed across 8,571 endothelial cells identified in the global analysis to generate a force-directed graph and Louvain clustering (resolution = 0.6) using 5,082 variable genes. Genes enriched in each of 9 endothelial sub-cluster were identified using a Wilcox test in Seurat. For each addressin gene examined, the distribution of cells with non-zero expression was examined in each of the endothelial cells.

#### Identification of Fibroblast Heterogeneity

Sub-analysis was performed across 7,237 fibroblasts identified in global analysis. UMAP dimensionality reduction using 4,825 variable genes and Louvain clustering (resolution = 0.6) were performed. Enrichment analysis was performed using a Wilcox test to identify cluster-defining genes for each of 10 fibroblasts sub-clusters. For each fibroblast sub-cluster, the fractional composition of cells from each sample and condition was examined.

#### Enrichment of Immune and Stromal Populations by Condition

To understand enrichment of specific cell types in a given condition, the proportional composition of a given cell subset was calculated with each skin biopsy. For example, the proportion of T cell subsets obtained from each of 19 skin biopsies was determined as the number of cells of a given sub-type divided by the total number of T cells obtained from that biopsy. To assess significance of enrichment within a given inflammatory skin condition, comparisons were performed in two ways: (1) a Mann-Whitney U test was used to examine differences in the proportion of individual cell subsets between biopsies of a given condition and all other samples; and (2) a Mann-Whitney U test was performed between proportions from biopsies of a given condition and those obtained from normal skin. Specifically, we calculated enrichments for those conditions for which at least 2 biopsies were obtained, i.e. acne (n-4), leprosy (n=4), granuloma annulare (n=2), and psoriasis (n=5). No comparisons of enrichment were performed for the single alopecia areata biopsy. We performed tests of enrichment within each of the following subsets: T cells, myeloid cells, dendritic cells, fibroblasts and endothelial cells.

#### Pseudo-temporal Reconstruction of Epidermal Keratinocytes

Diffusion analysis was performed across all keratinocytes and hair follicle cells using the Diffmap function in Scanpy (Wolf et al., 2018), which implements a method for diffusion pseudotime reconstruction (Haghverdi et al., 2015). Pseudo-temporal analysis was performed within normal keratinocyte separately, using the basal keratinocyte population as the origin of the pseudo-temporal ordering. Differential expression analysis between normal and psoriatic keratinocytes was performed in Seurat using a Wilcox rank-sum test across all keratinocytes and among basal, differentiating, and terminal keratinocytes.

Differentiation trajectories were constructed for keratinocytes from normal and psoriatic skin biopsies using Scorpius as implemented in dyno (Saelens et al., 2019). Correlation of gene expression patterns with pseudo-temporal order were examined separately for keratinocyte populations from each sample. Here, linear regression was performed between pseudo-time values and gene expression values for normal and psoriatic keratinocytes. Differential pseudo-time correlation analysis was performed between normal and psoriatic keratinocytes for 2 normal skin samples and 5 psoriasis samples. The difference in average pseudo-time correlation between psoriatic and normal keratinocytes was calculated to identify genes that are uniquely involved in the development of psoriatic keratinocytes. Reference immuohistochemical staining for KRT14 and FLG was obtained from the Human Protein Atlas (KRT14: https://www.proteinatlas.org/ENSG00000186847-KRT14/tissue/skin+1#img and https://www.proteinatlas.org/ENSG00000143631-FLG/tissue/skin+2#img).

#### Keratinocyte Cytokine-Response Profiles

Among both normal and psoriatic keratinocytes, cytokine response scores were generated using a series of reference datasets that were previously generated from *in vitro* experiments in which cultured keratinocytes were stimulated with cytokines, individually or in combination (IL-17A, IL17-A + TNF-α, TNF-α, IFN-α, IL-4, IL-13, and IFN-γ). Expression signature were generated relative an unstimulated control population of keratinocytes. For each cytokine condition, the top 100 differentially expressed genes were used to generate a cytokine response score across both psoriatic and normal keratinocytes in Seurat. The distribution of cytokine response scores were examined across basal, differentiating, and terminal keratinocytes between normal and psoriatic keratinocytes.

### Additional Resources

An extensive protocol is included as part of the supplementary materials along with an itemized cost-model for Seq-Well S^3^. These are also available on Shalek lab website: http://shaleklab.com/resource/seq-well/.

## Supplemental Information

Document S1. Figures S1–S6 and Seqwell S^3^ Master Protocol

Table S1. Gene Detection Frequencies for Transcription Factors, Cytokines and Cytokine Receptors, Related to Figure 1 Results from gene-level comparisons between Seq-Well S3, 10x v2, and 10x v3.

Table S2. Patient Information and Cell-Level Metadata, Related to Figure 2 Cell-level metadata including manual and automated cell-type annotations.

Table S3. Gene Signatures, Related to Figures 2, 3, 4, and 5 Gene signatures for cell-type clusters at multiple levels: generic cell-types, T cell subsets, myeloid cells, dendritic cells, fibroblasts, and endothelial cells.

Table S4. Enrichment of Sub-phenotypes across Inflammatory Skin Conditions, related to Figures 3, 4, and 5 Statistical significance of proportional differences in immune and stromal sub-phenotypes in inflammatory skin conditions.

Table S5. Differential Expression and Gene-Set Enrichment Analysis between Langerhan’s Cells from Leprosy and Normal Skin, Related to Figure 4 Differential expression results between LC cells in Leprosy and normal skin and gene-set enrichment analysis of genes overexpressed in Langerhan’s cells from Leprosy.

Table S6. Genes Differentially Expressed and Differentially Correlated between Psoriatic and Normal Keratinocytes and Keratinocyte Cytokine Response Signatures, Related to Figure 6 Differential Expression Results between psoriatic and normal keratinocytes. Per-cell pseudo-time correlation values for normal and psoriatic keratinocytes. Gene expression signatures generated after cytokine exposure of keratinocyte *in vitro*.

Document S2. Article plus Supplemental Information
