# Supplementary material for: Second-Strand Synthesis-Based Massively Parallel scRNA-Seq Reveals Cellular States and Molecular Features of Human Inflammatory Skin Pathologies
Source: Immunity. 2020 Oct 13;53(4):878–894.e7. doi: 10.1016/j.immuni.2020.09.015 (PMC7562821; doi:10.1016/j.immuni.2020.09.015)
Supplement: Document S1. Figures S1–S6 and Seqwell S3 Master Protocol [file mmc1.pdf]

Immunity, Volume *53*

Supplemental Information

Second-Strand Synthesis-Based Massively Parallel scRNA-Seq Reveals Cellular States and Molecular Features of Human Inflammatory Skin Pathologies

Travis K. Hughes, Marc H. Wadsworth II, Todd M. Gierahn, Tran Do, David Weiss, Priscila R. Andrade, Feiyang Ma, Bruno J. de Andrade Silva, Shuai Shao, Lam C. Tsoi, Jose Ordovas-Montanes, Johann E. Gudjonsson, Robert L. Modlin, J. Christopher Love, and Alex K. Shalek

Figure S1.

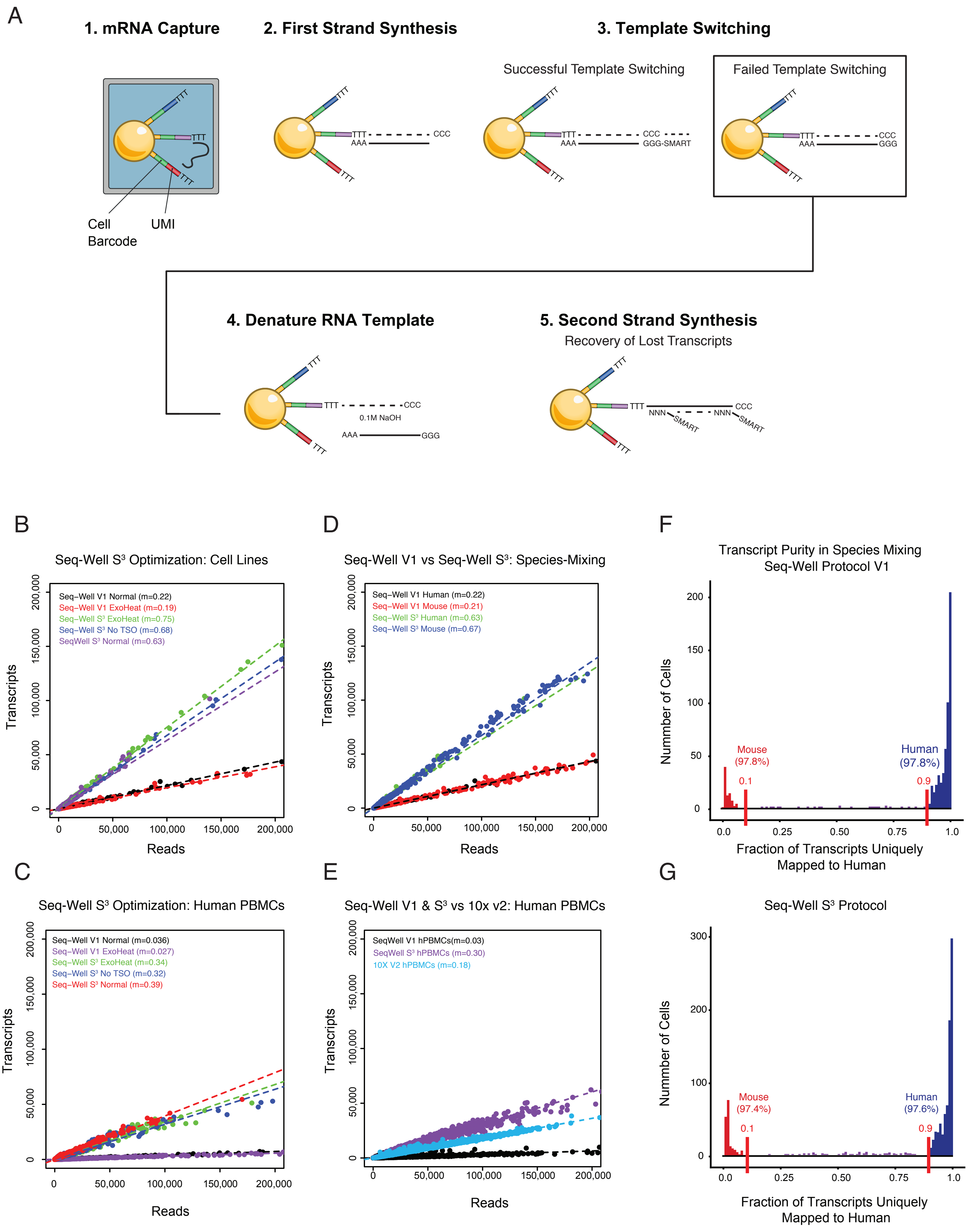
A
1. mRNA Capture
2. First Strand Synthesis
3. Template Switching
Successful Template Switching
Failed Template Switching
Cell Barcode
UMI
TTT
AAA
CCC
GGG-SMART
GGG
4. Denature RNA Template
0.1M NaOH
5. Second Strand Synthesis
Recovery of Lost Transcripts
NNN
SMART
B
Seq-Well S^3 Optimization: Cell Lines
Seq–Well V1 Normal (m=0.22)
Seq–Well V1 ExoHeat (m=0.19)
Seq–Well S^3 ExoHeat (m=0.75)
Seq–Well S^3 No TSO (m=0.68)
SeqWell S^3 Normal (m=0.63)
Transcripts
Reads
D
Seq-Well V1 vs Seq-Well S^3: Species-Mixing
Seq–Well V1 Human (m=0.22)
Seq–Well V1 Mouse (m=0.21)
Seq–Well S^3 Human (m=0.63)
Seq–Well S^3 Mouse (m=0.67)
Transcripts
Reads
F
Transcript Purity in Species Mixing
Seq-Well Protocol V1
Nummber of Cells
Mouse (97.8%)
Human (97.8%)
0.1
0.9
Fraction of Transcripts Uniquely Mapped to Human
C
Seq-Well S^3 Optimization: Human PBMCs
Seq–Well V1 Normal (m=0.036)
Seq–Well V1 ExoHeat (m=0.027)
Seq–Well S^3 ExoHeat (m=0.34)
Seq–Well S^3 No TSO (m=0.32)
Seq–Well S^3 Normal (m=0.39)
Transcripts
Reads
E
Seq-Well V1 & S^3 vs 10x v2: Human PBMCs
SeqWell V1 hPBMCs(m=0.03)
SeqWell S^3 hPBMCs (m=0.30)
10X V2 hPBMCs (m=0.18)
Transcripts
Reads
G
Seq-Well S^3 Protocol
Nummber of Cells
Mouse (97.4%)
Human (97.6%)
0.1
0.9
Fraction of Transcripts Uniquely Mapped to Human

Figure S1. Second-Strand Synthesis Overview, related to Figure 1

A. Illustration of the second strand synthesis procedure: (1) mRNA is captured via poly-T priming of poly-adenylated mRNA; (2) First strand synthesis is performed to generate single-stranded cDNA template on bead-bound sequences; (3) Successful template switching: The use of enzymes with terminal transferase activity generates a 3' overhang of 3 cytosines. Template switching utilizes this overhang to append the SMART sequence to both ends of the cDNA molecule during first strand synthesis. Failed Template Switching: If template switching fails, this results in loss of previously primed and reverse transcribed mRNA molecules; (4) mRNA template is chemically denatured using 0.1M NaOH; (5) Second strand synthesis is performed using a random-octamer with the SMART sequence in the 5' orientation; and, (6) Following second strand synthesis, PCR amplification, library preparation and sequencing are performed to generate data.

B. Scatterplots show the relationship between transcript detection (y-axis) and number of aligned reads per cell (x-axis) for a series of optimization experiments using HEK293 and NIH-3T3 cell lines.

C. Scatterplots show the relationship between transcript detection (y-axis) and the number of aligned reads per cell (x-axis) for a series of optimization experiments using PBMCs.

D. Scatterplots that illustrate the relationship between number of transcripts detected (y-axis) and number of aligned reads per cell (x-axis) between Seq-Well V1 and Seq-Well S^3 in species mixing experiments using HEK293 and NIH-3T3 cells.

E. Scatterplots that illustrate the relationship between number of transcripts detected (y-axis) and number of aligned reads per cell (x-axis) between Seq-Well V1 and Seq-Well S^3 in a series of optimization experiment using human PBMCs.

F. Histograms that show the fraction of transcripts uniquely mapped to the human genome for each cell for Seq-Well V1. Colors indicate species classification for cells with at least 90% purity of human (blue) or mouse (red) mapping.

G. Histograms that show the fraction of transcripts uniquely mapped to the human genome for each cell for Seq-Well S^3. Colors indicate species classification for cells with at least 90% purity of human (blue) or mouse (red) mapping.

Figure S2.

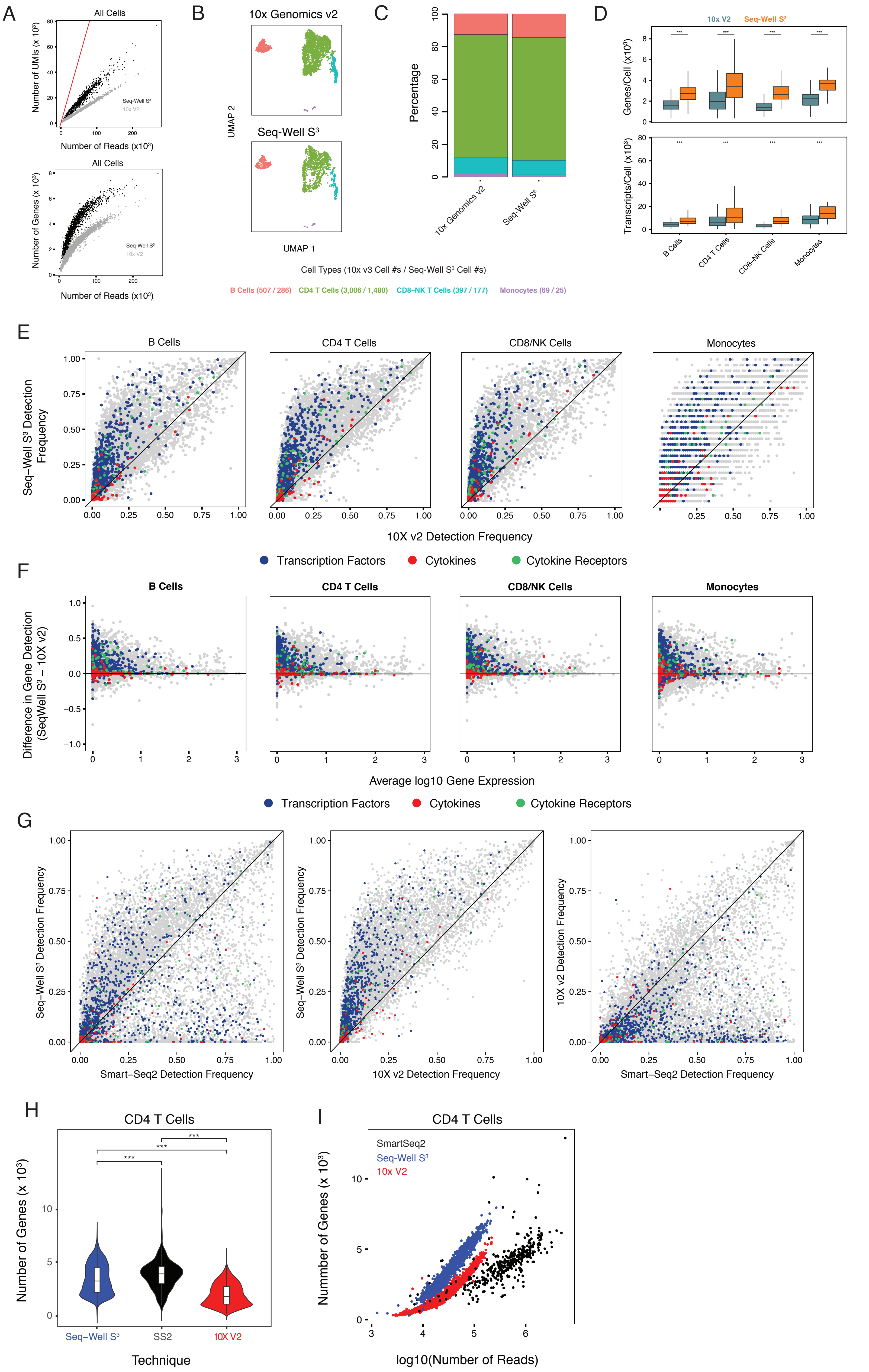

A
All Cells
Number of UMIs (x 10³)
Number of Reads (x10³)
Seq-Well S³
10x V2
All Cells
Number of Genes (x 10³)
Number of Reads (x10³)
B
10x Genomics v2
Seq-Well S³
UMAP 2
UMAP 1
C
Percentage
10x Genomics v2
Seq-Well S³
D
10x V2
Seq-Well S³
Genes/Cell (x10³)
Transcripts/Cell (x10³)
B Cells
CD4 T Cells
CD8-NK Cells
Monocytes
Cell Types (10x v3 Cell #s / Seq-Well S³ Cell #s)
B Cells (507 / 286)
CD4 T Cells (3,006 / 1,480)
CD8–NK T Cells (397 / 177)
Monocytes (69 / 25)
E
B Cells
CD4 T Cells
CD8/NK Cells
Monocytes
Seq–Well S³ Detection Frequency
10X v2 Detection Frequency
Transcription Factors
Cytokines
Cytokine Receptors
F
B Cells
CD4 T Cells
CD8/NK Cells
Monocytes
Difference in Gene Detection (SeqWell S³ – 10X v2)
Average log10 Gene Expression
Transcription Factors
Cytokines
Cytokine Receptors
G
Seq–Well S³ Detection Frequency
Smart–Seq2 Detection Frequency
Seq–Well S³ Detection Frequency
10X v2 Detection Frequency
10X v2 Detection Frequency
Smart–Seq2 Detection Frequency
H
CD4 T Cells
Number of Genes (x 10³)
Seq–Well S³
SS2
10X V2
Technique
I
CD4 T Cells
SmartSeq2
Seq-Well S³
10x V2
Nummber of Genes (x 10³)
log10(Number of Reads)

Figure S2. PBMC Methods Comparisons, related to Figure 1

A. Top: Scatterplot showing differences in per-cell transcript capture (y-axis) as a function of aligned reads per cell (x-axis) between 10x Genomics v2 (10x v3, grey) and Seq-Well S^3 (black). Red line indicates uniform line where transcripts per cell and aligned reads would be equivalent. Bottom: Scatterplot shows the differences in per-cell gene detection (y-axis) as a function of aligned reads per cell (x-axis) between 10x v2 (grey) and Seq-Well S^3 (black).

B. UMAP plots showing detected cell-types among PBMCs using 10X v2 (left) and Seq-Well S^3 (right).

C. Stacked barplots show the proportion of cell types recovered using Seq-Well S^3 (left) and 10X v2 (right).

D. Top: Boxplots (median +- quartiles) showing the distribution of per cell gene detection from 10X v2 (left) and Seq-Well S^3 (right). Bottom: Boxplots (median +/- quartiles) showing the distribution of per cell- transcript capture from 10X v2 (left) and Seq-Well S^3 (right).

E. Scatterplots showing a comparison of gene detection frequencies between Seq-Well S^3 (y-axis) and 10x v2 (x-axis) for each cell type.

F. Scatterplots showing the difference in gene detection between Seq-Well S^3 and 10X v2 (y-axis) as a function of average normalized expression (log(scaled UMI + 1)) (x-axis).

G. Scatterplots showing a comparison of gene detection frequencies among sorted $CD4^+$ T cells between **(Left)** Seq-well S^3 (y-axis) and 10x v2 (x-axis), (**Middle)** Seq-Well S^3 (y-axis) and Smart-Seq2 (x-axis), and **(Right)** 10x v2 (y-axis) and Smart-Seq2 (x-axis).

H. Violin plots (boxplots median +- quartiles) showing the distribution of per-cell transcript capture for Seq-well S^3 (blue; n = 1,485), 10x v2 (red; n = 2995), and Smart-Seq2 (black, n = 382). *** P-values < 1.0 $x10^{-10}$.

I. Scatterplot showing the relationship between aligned reads and genes detected per cell between Seq-Well S^3 (blue), 10x v2 (red) and Smart-Seq2 (black) in sorted PBMC $CD4^+$ T cells.

Figure S3.

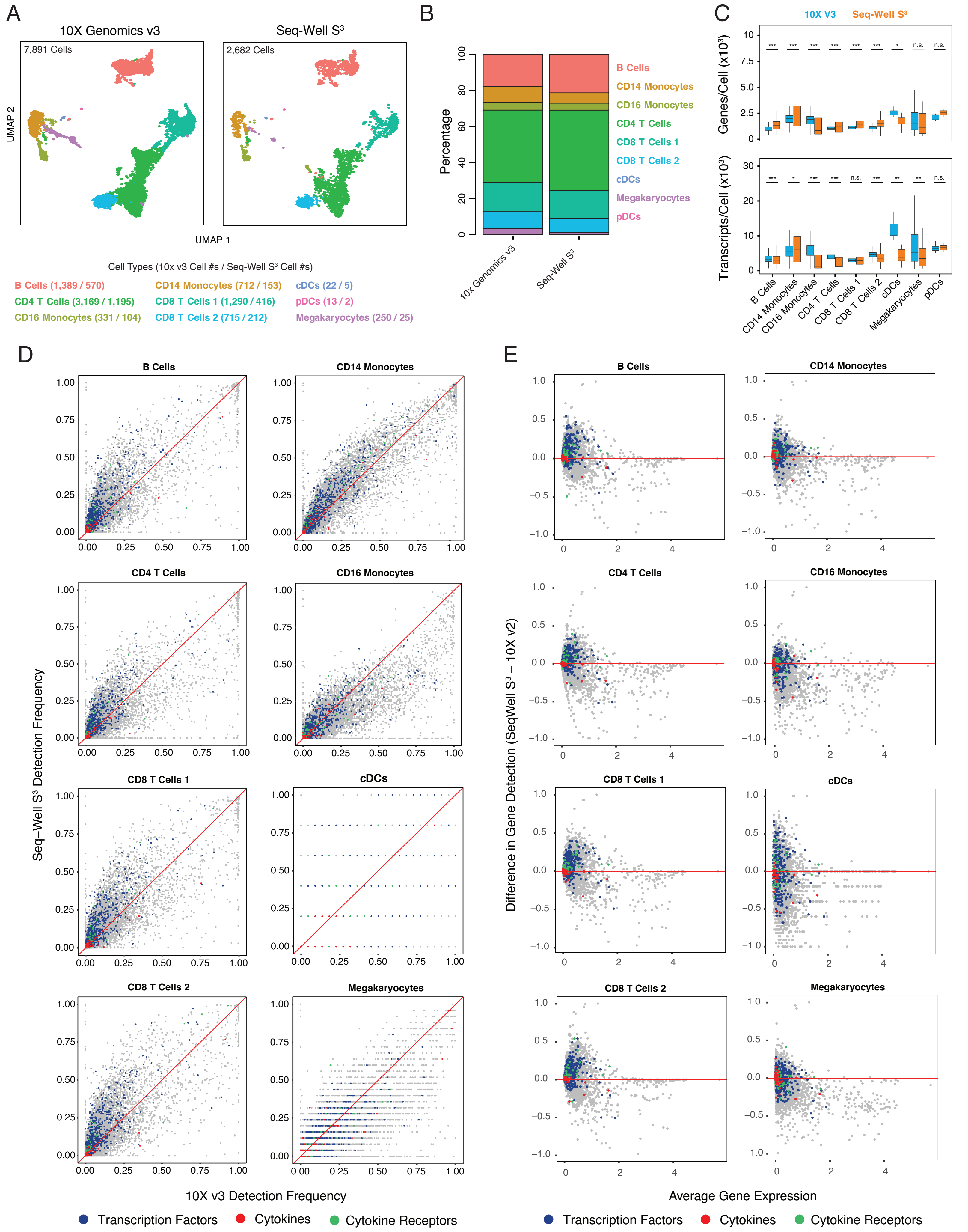
A
10X Genomics v3
Seq-Well S³
7,891 Cells
2,682 Cells
UMAP 2
UMAP 1
Cell Types (10x v3 Cell #s / Seq-Well S³ Cell #s)
B Cells (1,389 / 570)
CD4 T Cells (3,169 / 1,195)
CD16 Monocytes (331 / 104)
CD14 Monocytes (712 / 153)
CD8 T Cells 1 (1,290 / 416)
CD8 T Cells 2 (715 / 212)
cDCs (22 / 5)
pDCs (13 / 2)
Megakaryocytes (250 / 25)
B
Percentage
10x Genomics v3
Seq-Well S³
B Cells
CD14 Monocytes
CD16 Monocytes
CD4 T Cells
CD8 T Cells 1
CD8 T Cells 2
cDCs
Megakaryocytes
pDCs
C
10X V3
Seq-Well S³
Genes/Cell (x10³)
Transcripts/Cell (x10³)
D
B Cells
CD14 Monocytes
CD4 T Cells
CD16 Monocytes
CD8 T Cells 1
cDCs
CD8 T Cells 2
Megakaryocytes
Seq-Well S³ Detection Frequency
10X v3 Detection Frequency
E
Difference in Gene Detection (SeqWell S³ – 10X v2)
Average Gene Expression
Transcription Factors
Cytokines
Cytokine Receptors

Figure S3. Seq-Well S^3 vs 10x v3 3' PBMC Methods Comparisons, related to Figure 1

A. UMAP plots for 7,891 PBMCs from 10x Genomics v3 (left) and 2,682 PBMCs from Seq-Well S^3 (right).

B. Stacked barplots showing the distribution of recovered cell types between 10x Genomics v3 and Seq-Well S^3.

C. Top: Boxplots (median +- quartiles) showing the distribution of per cell gene detection from 10X v3 (left) and Seq-Well S^3 (right). Bottom: Boxplots (median +/- quartiles) showing the distribution of per cell-gene detection from 10X v3 (left) and Seq-Well S^3 (right).

D. Scatterplots showing a comparison of gene detection frequencies between Seq-Well S^3 (y-axis) and 10x v3 (x-axis) for each cell type.

E. Scatterplots showing the difference in gene detection between Seq-Well S^3 and 10X v3 (y-axis) as a function of average expression (counts) (x-axis).

Figure S4.

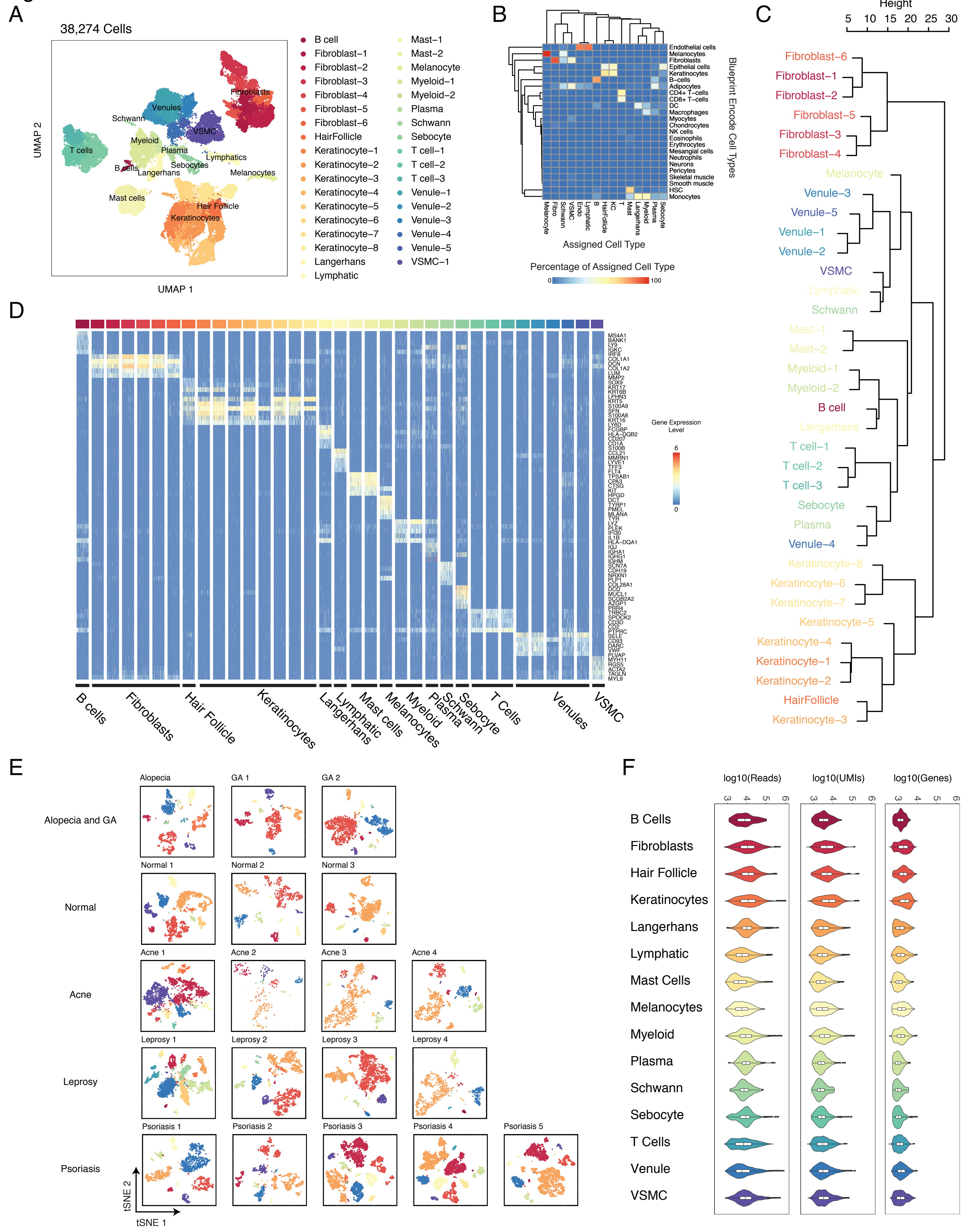
A
38,274 Cells
UMAP 2
UMAP 1
Fibroblasts
Venules
Schwann
VSMC
Myeloid
T cells
Plasma
Lymphatics
Sebocytes
B cells
Langerhans
Melanocytes
Mast cells
Hair Follicle
Keratinocytes
B cell
Fibroblast−1
Fibroblast−2
Fibroblast−3
Fibroblast−4
Fibroblast−5
Fibroblast−6
HairFollicle
Keratinocyte−1
Keratinocyte−2
Keratinocyte−3
Keratinocyte−4
Keratinocyte−5
Keratinocyte−6
Keratinocyte−7
Keratinocyte−8
Langerhans
Lymphatic
Mast−1
Mast−2
Melanocyte
Myeloid−1
Myeloid−2
Plasma
Schwann
Sebocyte
T cell−1
T cell−2
T cell−3
Venule−1
Venule−2
Venule−3
Venule−4
Venule−5
VSMC−1
B
Blueprint Encode Cell Types
Endothelial cells
Melanocytes
Fibroblasts
Epithelial cells
Keratinocytes
B−cells
Adipocytes
CD4+ T−cells
CD8+ T−cells
DC
Macrophages
Myocytes
Chondrocytes
NK cells
Eosinophils
Erythrocytes
Mesangial cells
Neutrophils
Neurons
Pericytes
Skeletal muscle
Smooth muscle
HSC
Monocytes
Melanocyte
Fibro
Schwann
VSMC
Endo
Lymphatic
B
HairFollicle
KC
T
Mast
Langerhans
Myeloid
Plasma
Sebocyte
Assigned Cell Type
Percentage of Assigned Cell Type
0
100
C
Height
5 10 15 20 25 30
Fibroblast−6
Fibroblast−1
Fibroblast−2
Fibroblast−5
Fibroblast−3
Fibroblast−4
Melanocyte
Venule−3
Venule−5
Venule−1
Venule−2
VSMC
Lymphatic
Schwann
Mast−1
Mast−2
Myeloid−1
Myeloid−2
B cell
Langerhans
T cell−1
T cell−2
T cell−3
Sebocyte
Plasma
Venule−4
Keratinocyte−8
Keratinocyte−6
Keratinocyte−7
Keratinocyte−5
Keratinocyte−4
Keratinocyte−1
Keratinocyte−2
HairFollicle
Keratinocyte−3
D
MS4A1
BANK1
LY9
IGKC
IRF8
COL1A1
DCN
COL1A2
LUM
MMP2
SOX9
KRT17
KRT6B
LPHN3
KRT5
S100A9
SFN
S100A8
KRT16
LY6D
FCGBP
HLA−DQB2
CD207
CD1A
S100B
CCL21
MMRN1
LYVE1
TFF3
FLT4
TPSAB1
CPA3
CTSG
KIT
HPGD
DCT
TYRP1
PMEL
MLANA
TYR
LYZ
PLEK
IFI30
IL1B
HLA−DQA1
IGJ
IGHA1
IGHG1
IGHM
SCN7A
CDH19
NRXN1
PLP1
COL28A1
DCD
MUCL1
SCGB2A2
AZGP1
PRR4
TRBC2
SPOCK2
CD3D
CD2
PTPRC
SELE
CD93
DARC
VWF
PLVAP
MYH11
RGS5
ACTA2
TAGLN
MYL9
Gene Expression Level
6
0
B cells
Fibroblasts
Hair Follicle
Keratinocytes
Langerhans
Lymphatic
Mast cells
Melanocytes
Myeloid
Plasma
Schwann
Sebocyte
T Cells
Venules
VSMC
E
Alopecia and GA
Alopecia
GA 1
GA 2
Normal
Normal 1
Normal 2
Normal 3
Acne
Acne 1
Acne 2
Acne 3
Acne 4
Leprosy
Leprosy 1
Leprosy 2
Leprosy 3
Leprosy 4
Psoriasis
Psoriasis 1
Psoriasis 2
Psoriasis 3
Psoriasis 4
Psoriasis 5
tSNE 2
tSNE 1
F
log10(Reads)
log10(UMIs)
log10(Genes)
3 4 5 6
B Cells
Fibroblasts
Hair Follicle
Keratinocytes
Langerhans
Lymphatic
Mast Cells
Melanocytes
Myeloid
Plasma
Schwann
Sebocyte
T Cells
Venule
VSMC

Figure S4. Overview of Samples, related to Figure 2

A. UMAP plot for 38,274 cells colored by 35 cell type clusters.

B. Comparison of cell-type classification to results obtained using SingleR. Color corresponds to the percentage of manually classified cells types (columns) assigned to cell-type references contained in the Blueprint Encode dataset (rows).

C. Dendrogram of hierarchical clustering shows similarity of cell type clusters among top 25 cluster-defining genes.

D. Heatmap showing the relative expression of cell-type defining gene signatures across 38,274 cells (**Table S4**).

E. t-SNE plots for each of the nineteen skin biopsies colored by generic cell type.

F. Violin plots show the distribution of per-cell quality metrics displayed in UMAP embedding of 38,274 cells colored by colored generic cell-type classification.

Figure S5.

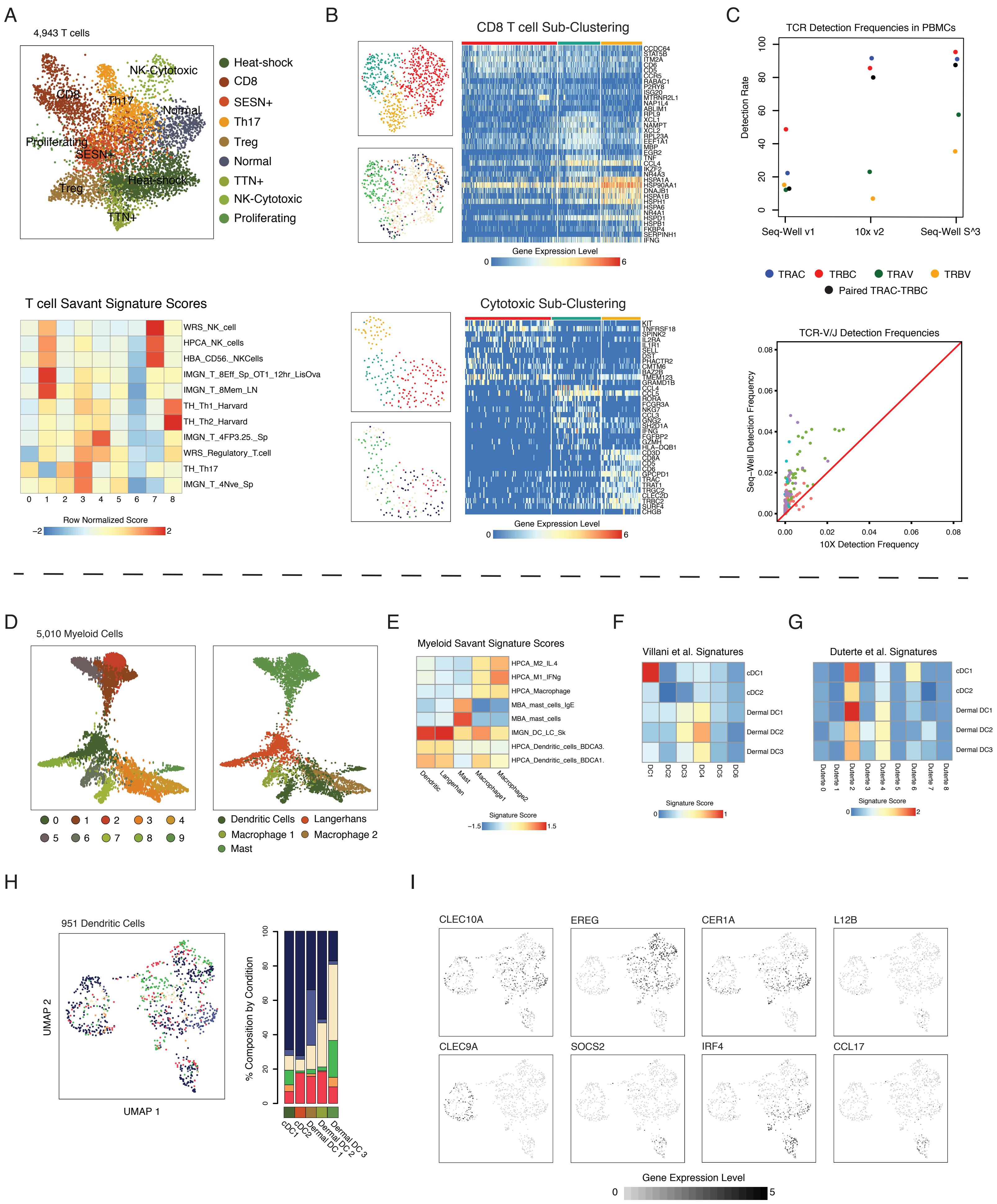
A
4,943 T cells
Heat-shock
CD8
SESN+
Th17
Treg
Normal
TTN+
NK-Cytotoxic
Proliferating
T cell Savant Signature Scores
WRS_NK_cell
HPCA_NK_cells
HBA_CD56._NKCells
IMGN_T_8Eff_Sp_OT1_12hr_LisOva
IMGN_T_8Mem_LN
TH_Th1_Harvard
TH_Th2_Harvard
IMGN_T_4FP3.25._Sp
WRS_Regulatory_T.cell
TH_Th17
IMGN_T_4Nve_Sp
Row Normalized Score
B
CD8 T cell Sub-Clustering
Gene Expression Level
Cytotoxic Sub-Clustering
C
TCR Detection Frequencies in PBMCs
Detection Rate
Seq-Well v1
10x v2
Seq-Well S^3
TRAC
TRBC
TRAV
TRBV
Paired TRAC-TRBC
TCR-V/J Detection Frequencies
Seq−Well Detection Frequency
10X Detection Frequency
D
5,010 Myeloid Cells
Dendritic Cells
Langerhans
Macrophage 1
Macrophage 2
Mast
E
Myeloid Savant Signature Scores
HPCA_M2_IL.4
HPCA_M1_IFNg
HPCA_Macrophage
MBA_mast_cells_IgE
MBA_mast_cells
IMGN_DC_LC_Sk
HPCA_Dendritic_cells_BDCA3.
HPCA_Dendritic_cells_BDCA1.
Signature Score
F
Villani et al. Signatures
cDC1
cDC2
Dermal DC1
Dermal DC2
Dermal DC3
Signature Score
G
Duterte et al. Signatures
Signature Score
H
951 Dendritic Cells
UMAP 2
UMAP 1
% Composition by Condition
cDC1
cDC2
Dermal DC 1
Dermal DC 2
Dermal DC 3
I
CLEC10A
EREG
CER1A
L12B
CLEC9A
SOCS2
IRF4
CCL17
Gene Expression Level

Figure S5. Immune Cell Heterogeneity, related to Figures 3 and 4

A. (Top) Force-directed graph of 4,943 T cells colored by T cell sub-cluster. (Bottom) Heatmap of gene-set enrichment scores based on comparison of T cell phenotypic sub-clusters to a curated list of reference signatures in the Savant database.

B. Sub-grouping results for CD8 T cells (top) (**Table S7**) and cytotoxic cells (bottom) (**Table S8**). For each analysis, t-SNE plots colored by inflammatory skin condition (top-left) and sub-cluster (bottom-left) are shown. For each clusters, heatmaps show gene expression patterns across T and NK cells sub-types (right).

C. (Top) Detection rates for TCR genes for PBMCs in Seq-Well v1, 10x v2. and Seq-Well S^3. (Bottom) Detection frequency of TCR V-J (e.g. TRAV/J and TRBV/J) genes in $CD4^+$ T cells from peripheral blood between Seq-Well S^3 (y-axis) and 10x v2 (x-axis). Colors correspond to TRAJ (red), TRAV (green), TRBJ (blue), and TRBV (purple) genes.

D. (Left) Force-directed graph of 5,010 myeloid cells colored by myeloid sub-clusters (Louvain resolution = 0.6). (Right) Force-directed graph of 5,010 myeloid cells colored by myeloid phenotypes.

E. Heatmap of gene-set enrichment scores based on comparison of myeloid phenotypic sub-clusters to a curated list of reference signatures in the Savant database.

F. Heatmap showing average signature score across 5 dermal DC populations based on dendritic cell signatures from *Villani et al. Science 2017*.

G. Heatmap showing average signature score across 5 dermal DC populations based on signatures from et al. Immunity 2019.

H. (Left) UMAP plot for 951 dendritic cells from human skin colored by inflammatory skin condition. (Right) Stacked barplot showing composition of dendritic cells within each of nine skin biopsies by DC sub-cluster.

I. UMAP plots colored by normalized expression levels for DC sub-grouping-defining genes.

Figure S6.

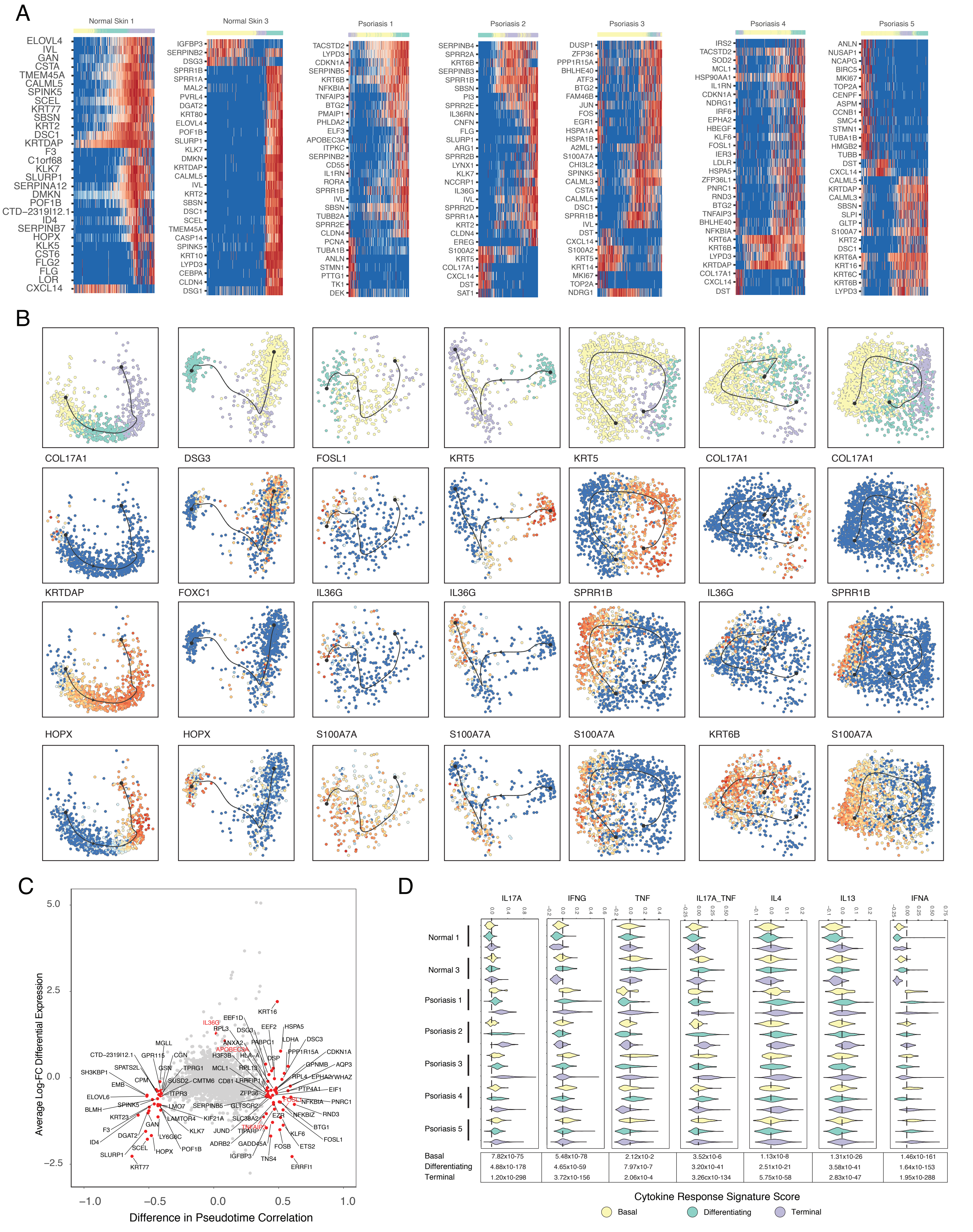
A
Normal Skin 1
Normal Skin 3
Psoriasis 1
Psoriasis 2
Psoriasis 3
Psoriasis 4
Psoriasis 5
B
COL17A1
DSG3
FOSL1
KRT5
KRT5
COL17A1
COL17A1
KRTDAP
FOXC1
IL36G
IL36G
SPRR1B
IL36G
SPRR1B
HOPX
HOPX
S100A7A
S100A7A
S100A7A
KRT6B
S100A7A
C
Average Log-FC Differential Expression
Difference in Pseudotime Correlation
D
IL17A
IFNG
TNF
IL17A_TNF
IL4
IL13
IFNA
Normal 1
Normal 3
Psoriasis 1
Psoriasis 2
Psoriasis 3
Psoriasis 4
Psoriasis 5
Basal
Differentiating
Terminal
7.82x10-75
4.88x10-178
1.20x10-298
5.48x10-78
4.65x10-59
3.72x10-156
2.12x10-2
7.97x10-7
2.06x10-4
3.52x10-6
3.20x10-41
3.26cx10-134
1.13x10-8
2.51x10-21
5.75x10-58
1.31x10-26
3.58x10-41
2.83x10-47
1.46x10-161
1.64x10-153
1.95x10-288
Cytokine Response Signature Score
Basal
Differentiating
Terminal

Figure S6. Keratinocyte Differentiation Trajectories, related to Figure 6

A. Heatmaps showing enrichment of genes along pseudo-temporal trajectories for keratinocytes from 2 normal skin biopsies and 5 psoriatic keratinocyte biopsies.

B. Plots showing the expression of trajectory-defining genes for 2 normal and 5 psoriatic biopsies.

C. Scatterplot showing the relationship between differential expression between psoriatic and normal keratinocyte (y-axis) and differential pseudotime correlation (x-axis). Genes highlighted in red have differential correlation values greater than 0.4 or less than -0.4. Gene names highlighted in red are genes examined through *in situ* staining shown in **Figure 5H**.

D. Violin plots showing localization of cytokine response signatures in basal, differentiating and terminal keratinocytes across 2 normal and 5 psoriatic biopsies. P-values from T-tests showing differences in cytokine signature scores for basal, differentiating and terminal keratinocytes between normal and psoriatic keratinocytes.

Seq-Well S^3 Master Protocol

As outlined in:

Highly Efficient, Massively-Parallel Single-Cell RNA-Seq Reveals Cellular States and Molecular Features of Human Skin Pathology

Travis K Hughes[1,2,3,4,5,12], Marc H Wadsworth II[1,3,4,5,12], Todd M Gierahn[5,6,12], Tran Do[7], David Weiss[7], Priscila R. Andrade[7], Feiyang Ma[7], Bruno J. de Andrade Silva[7], Shuai Shao[8], Lam C Tsoi[8], Jose Ordovas-Montanes [3,9,10,11], Johann E Gudjonsson[8], Robert L Modlin[9], J Christopher Love[3,4,5,6,13], and Alex K Shalek[1,2,3,4,5,13, 14]

[1] Institute for Medical Engineering & Science (IMES) and Department of Chemistry, MIT, Cambridge, Massachusetts, USA

[2] Department of Immunology, Harvard Medical School, Boston, MA 02115, USA

[3] Broad Institute of MIT and Harvard, Cambridge, Massachusetts, USA

[4] Ragon Institute of MGH, MIT and Harvard, Cambridge, Massachusetts, USA

[5] Koch Institute for Integrative Cancer Research, MIT, Cambridge, Massachusetts, USA

[6] Department of Chemical Engineering, MIT, Cambridge, Massachusetts, USA

[7] Division of Dermatology and Department of Microbiology, Immunology and Molecular Biology, David Geffen School of Medicine at UCLA, Los Angeles, California, USA

[8] Department of Dermatology, University of Michigan, Ann Arbor, Michigan, USA

[9] Division of Gastroenterology, Boston Children's Hospital, Boston, MA, USA.

[10] Department of Pediatrics, Harvard Medical School, Boston, MA, USA.

[11] Harvard Stem Cell Institute, Cambridge, MA, USA.

[12] These authors contributed equally to this work

[13] These senior authors contributed equally to this work

[14] Lead Contacts: Alex K. Shalek (AKS), shalek@mit.edu; J. Christopher Love, clove@mit.edu

Table of Contents

In-Depth Protocol

3 Membrane Preparation
7 Bead Loading
11 Cell Loading
12 Membrane Sealing
16 Cell Lysis and Hybridization
17 Bead Removal Method 1
18 Reverse Transcription & Exonuclease Digestion
19 Second Strand Synthesis & PCR
22 Library Preparation
24 Sequencing

Appendices

25 A: Array Synthesis
29 B: Synthesis Protocol Checklist
31 C: Mounting the Master
32 D: Buffer Guide
35 E: Bead Removal Method 2
36 F: Imaging in Array
37 G: Shopping List

Shalek & Love Labs, MIT

Membrane Preparation

1. Carefully place a pre-cut (22 x 66 mm) polycarbonate membrane onto a glass slide using a gloved finger and tweezers to separate the membrane and paper.
   Note 1: Make certain the shiny side of the polycarbonate membrane is facing up to be in contact with the oxygen plasma and eventually the surface of the array.
   Note 2: Discard any membranes that have creases or other large-scale imperfections.

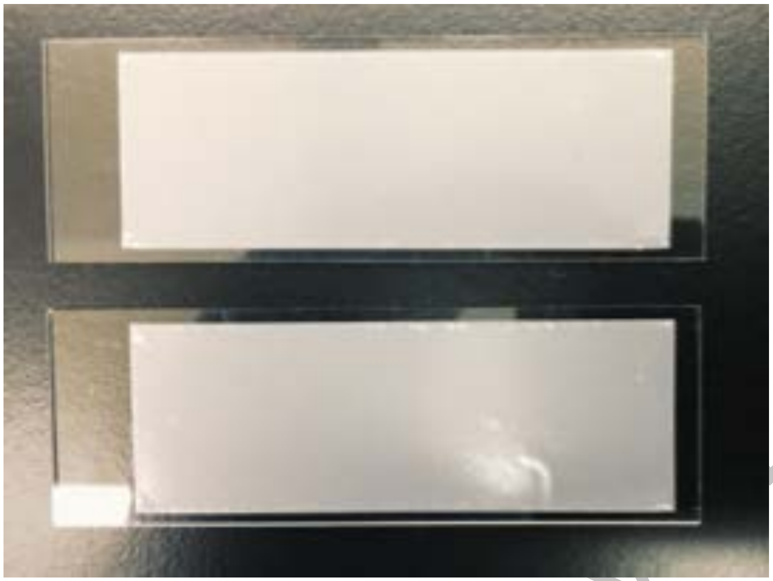

2. Place membranes onto a shelf in the plasma cleaner.
   Note 1: Shelves are not provided, but any piece of glass will do.
   Note 2 (optional): If you have two shelves, place membranes on the bottom shelf to reduce risk of them flying after vacuum is removed.

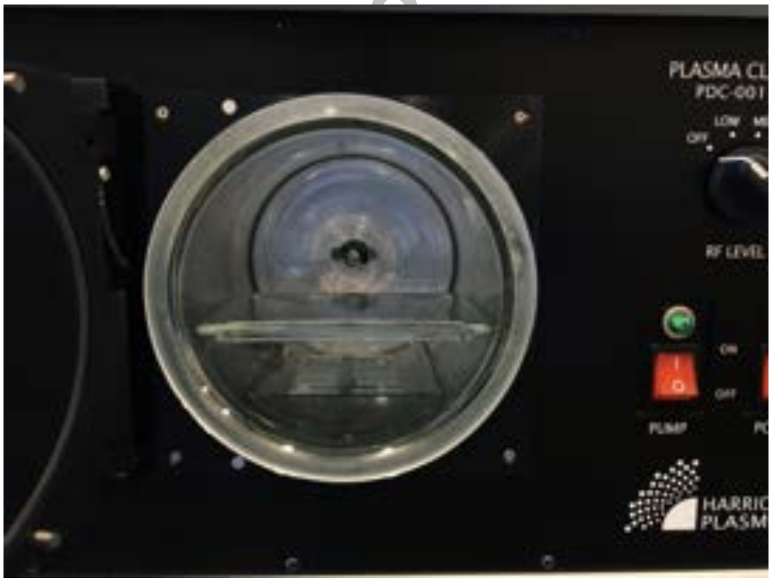

3. Close the plasma cleaner door, then turn on the main power and pump switch. To

Labs, MIT

form a vacuum, ensure that the 3-way valve lever is at the 9:00 position as shown below and that the door is completely shut.

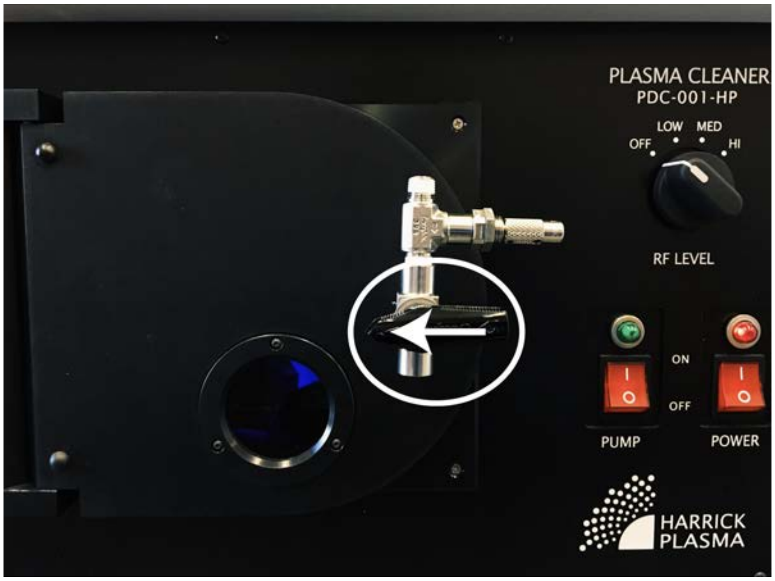

4. Allow vacuum to form for 2-3 minutes. Once the vacuum has formed, simultaneously turn the valve to 12:00 while turning the power to the Hi setting (shown below).
Note: The plasma should be a bright pink. If not, adjust the air valve to increase or decrease the amount of oxygen entering the chamber.

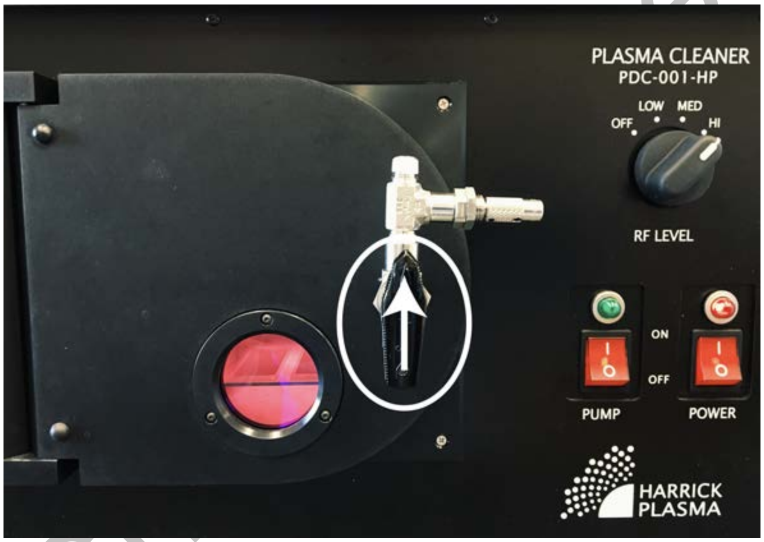

Labs, MIT

5. Treat membranes with plasma for 5-7 minutes.
   Note: We treat membranes for 7 minutes, but treatment times can vary.

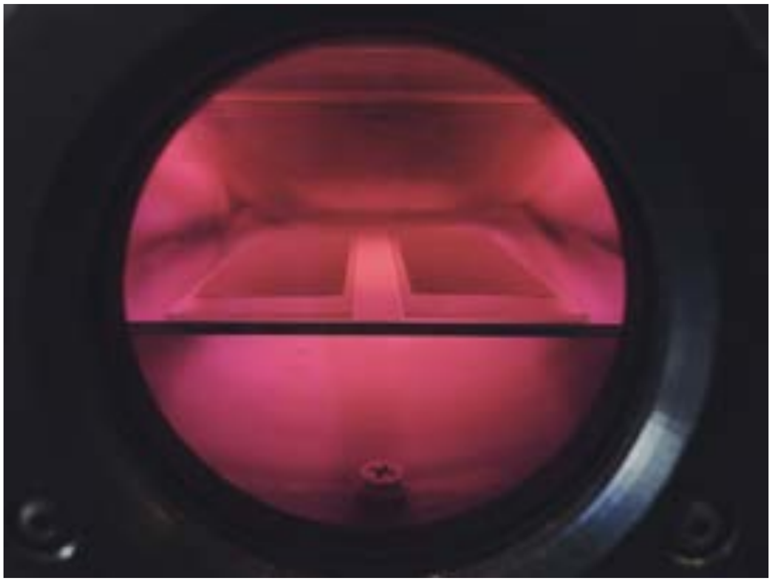

6. **Critical** – After treatment, in the following order: **(1)** turn the RF level valve from HIGH to OFF, **(2)** turn the air valve from the 12:00 position to the 9:00 position, and **(3)** then turn off the power followed by turning off the vacuum. Then *slowly open* the valve until air can be heard entering the chamber (approximate valve position shown below). Leave until door opens (~5 min).

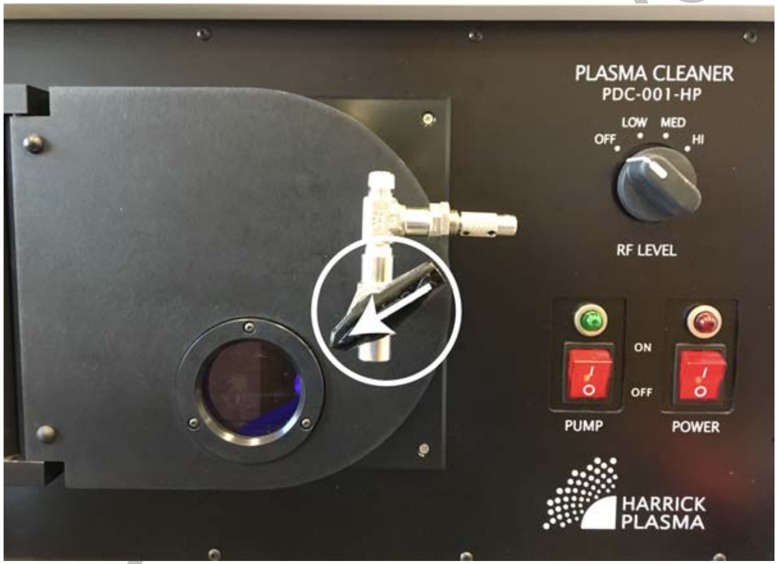

7. Remove slides (with membranes) from the plasma clean and transfer to a 4-well dish.
   Note 1: If membranes have slightly folded over, slowly flip the membrane back using needle nose tweezers.
   Note 2: If membranes have blown off the slide entirely, repeat above procedure to ensure you know which side was exposed to plasma.

e Labs, MIT

8. Using a P1000 pipette, gently hydrate one end of the membrane with a single drop of 1xPBS so that it adheres to the slide before dispensing the entire volume. Once the membrane is hydrated, continuing add 1xPBS until you reach 5 mL (use either a serological pipette or P1000 pipette to complete hydration).

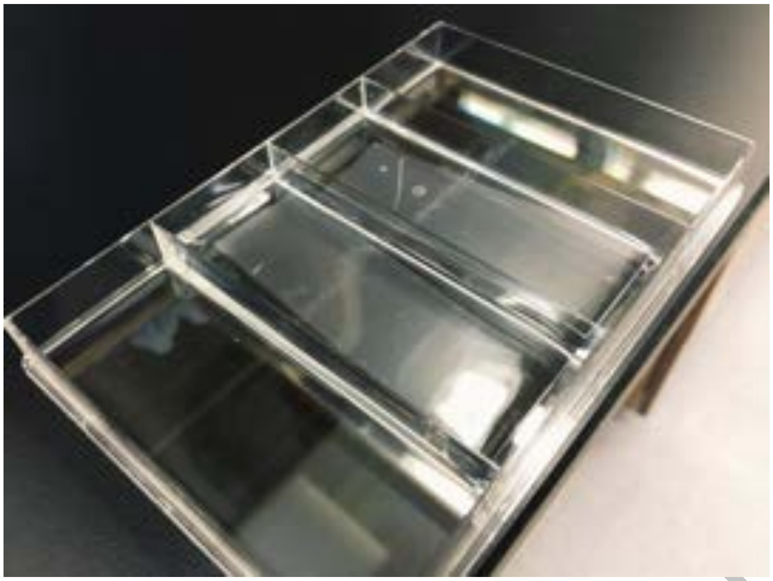

9. Remove any air bubbles underneath the membrane using wafer forceps or a pipette tip.

10. Membranes are now functionalized and ready for use.
Note 1: Membranes solvated with 1xPBS should be used within **48 hours.**
Note 2: If transporting solvated membranes (e.g. between buildings), remove all but ~1 mL of 1xPBS to prevent membranes from flipping within the dish.
Note 3: Alternatively, membranes initially solvated in 1xPBS can be dried and stored for 4 weeks at room temperature. To dry them out, carefully remove membranes, keeping them on their glass slides, from the 1xPBS solution, transfer the membranes to the benchtop, cover them with a tip box, and let them dry for 15-20 minutes. As the membranes dry they'll become opaque which is normal.
Note 4: Before use the membranes should be rehydrated with 5 mL of 1xPBS. Drying out membranes is helpful when traveling or when running seq-well in a laboratory without access to a plasma cleaner.

EXPERIMENTAL NOTES

Shalek & Love Labs, MIT

Bead Loading

1. Aspirate storage solution and solvate each array with 5 mL of bead loading buffer (BLB; **See Appendix D: Buffers Guide**).

2. Place array(s) under vacuum with rotation (50 RPM) for 10 minutes to remove air bubbles in wells. **Note:** Rotation is optional

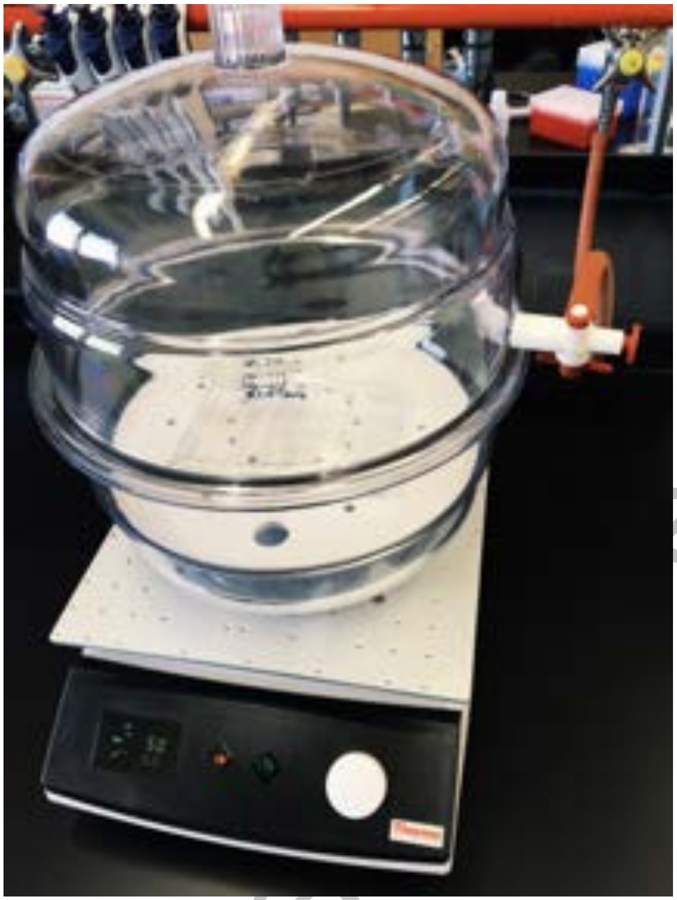

3. Aliquot ~110,000 beads from stock into a 1.5 mL tube and spin on a tabletop centrifuge for 15 seconds to form a pellet.

4. Aspirate storage buffer and wash beads twice in 500 uL of BLB.

5. Pellet beads, aspirate BLB, and resuspend beads in 200 uL of BLB.
Note: For each array, it's recommended to load ~110,000 beads.

6. Before loading beads, thoroughly aspirate BLB from the dish containing the array(s), being careful not to aspirate or dry the PDMS surface of the array(s).

Shalek Labs, MIT

7. Using a P200 pipette, apply 200 uL containing 110,000 beads, in a drop-wise fashion, to the surface of each array (see image below and Bead Loading Diagram on page 10).

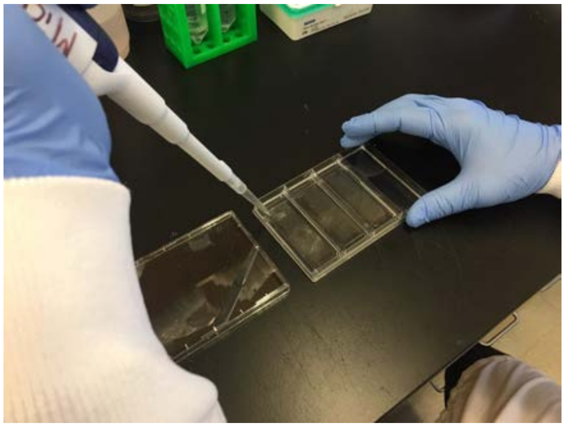

8. Allow the arrays to sit for 5 minutes, rocking them intermittently in the x & y direction. **Pro-Tip:** This step can be extended to 10 minutes to allow the beads more time to settle. However, make sure to monitor the surface of the array so that it doesn't dry out.

9. Thoroughly wash array(s) to remove excess beads from the surface. For each wash:
    1. Position each array so that it sits in the center of the 4-well dish.
    2. Dispense 500 uL of BLB in the upper right corner of each array and 500 uL in the bottom right corner of each array. Be careful not to directly pipette onto the microwells, as it can dislodge beads.
    3. Using wafer forceps or a pipette tip, push each array against the left side of the 4-well dish to create a capillary flow; this will help remove beads from the surface.
    4. Aspirate the liquid, reposition each array, and repeat on the other side.

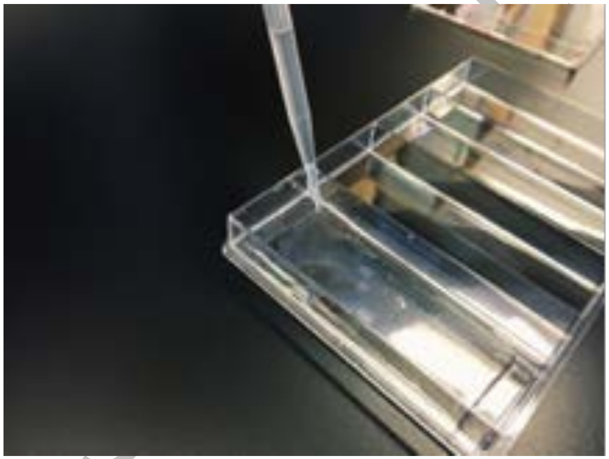

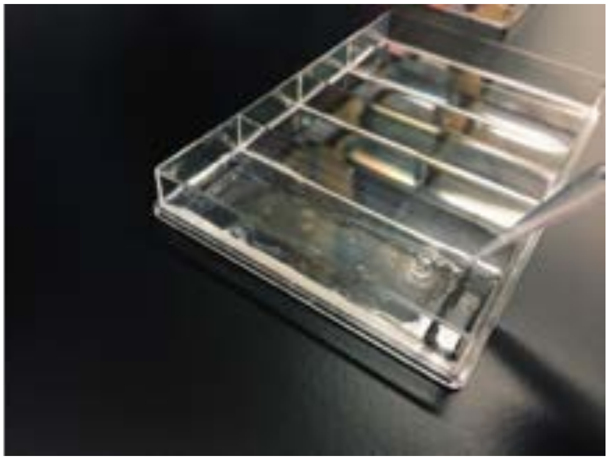

10. Repeat step 9 as necessary. Periodically examine the array(s) under microscope to confirm that no loose beads are present on the surface, as this will interfere with membrane attachment. Usually it takes 4 washes/side to thoroughly remove excess beads (this depends on your original loading density).

ove Labs, MIT

11. Once excess beads have been removed from the surface, solvate each array with 5 mL of BLB and proceed to cell loading.

Notes:

1. If continuing to cell loading immediately (i.e., within 6 hours), loaded arrays should be stored in 5 mL of BLB.
2. If you are not going to use the arrays on the day they're loaded, remove the BLB buffer, rinse the arrays once with 5 mL of 1xPBS, and then solvate the arrays with 5 mL of quenching buffer. Arrays can be stored in quenching buffer for 10 days (**See Appendix D: Buffers Guide**).

EXPERIMENTAL NOTES

Shalek & Love Labs, MIT

Bead Loading Diagrams

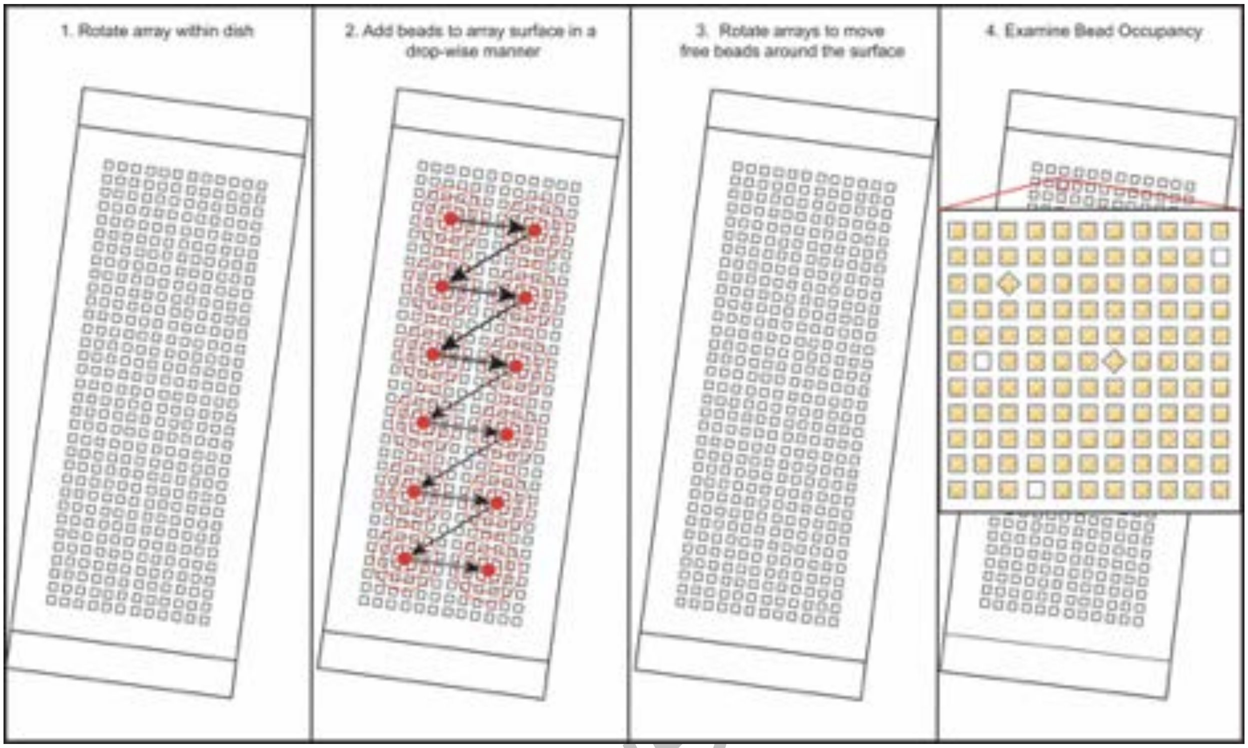

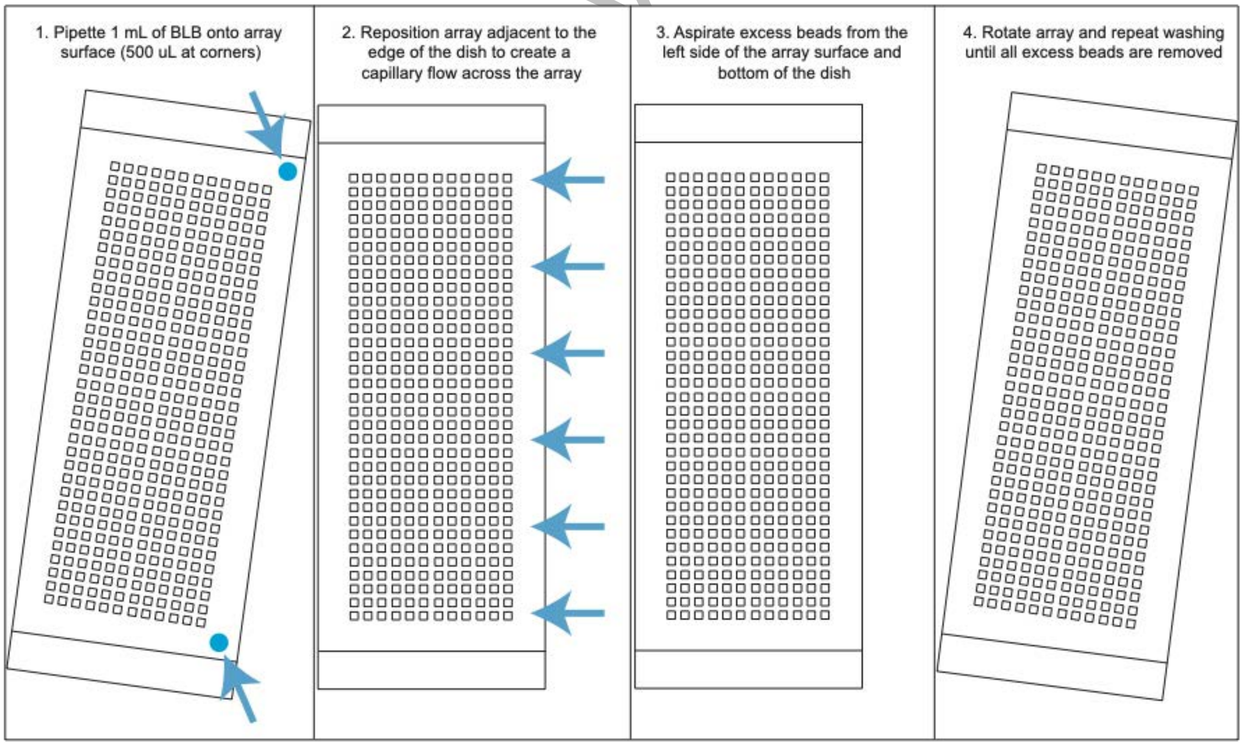

Cell Loading (Without Imaging)

If you want to image cells in the array, please refer to Appendix F

1. At this point, your array should be loaded with beads and sitting in 5 mL of BLB.

2. Obtain the cell or tissue sample and prepare a single cell suspension using an optimized protocol for tissue dissociation.

3. While preparing your single-cell suspension, aspirate the BLB from each array (or quenching buffer) and rinse the array twice in 5 mL of 1xPBS to bring the solution in the four-well dish to physiological pH.

4. After the second wash, aspirate the 1xPBS and soak the loaded array in 5 mL of RPMI + 10% (RP-10) FBS for 5 minutes.
   Note 1: This step is performed to mitigate non-specific adhesion of cells to primary amines on the top surface of the array.
   Note 2: Any supplemented media can be used in place of RP-10.

5. After obtaining a single-cell suspension, count cells using a hemocytometer and make a new solution of 10,000-15,000 cells in 200 uL of RP-10.
   Note 1: You can use your preferred media for prepping the cell loading solution.
   Note 2: Be sure to not use automated cell counters, particularly following tissue dissociation. This can provide an inaccurate cell count, compromising the experiment.

6. Thoroughly aspirate the RP-10/supplemented media (to ensure the array will not move during cell loading).
7. Center your array in the well and then apply the cell loading solution onto the surface in a dropwise fashion (similar to how beads were applied in the previous section).

8. Allow cells to settle for 10 minutes, intermittently rock the array in the x & y direction.

9. Wash array 4x with 5 mL of 1xPBS to remove the serum. For each wash, gently rock the array in the x & y direction, and then aspirate the 1xPBS. Once you have aspirated the 1xPBS out of the dish, gently tilt the 4-well dish toward you and aspirate directly off the bottom border of the array; this will help to completely remove the excess serum on the surface of the array
   Note: These washes are critical to remove excess serum which can interfere with successful membrane attachment.

10. Aspirate the final 1xPBS wash and replace with 5 mL of RPMI media ***without*** FBS.
    Note: You can use any media here as long as it **does not** contain serum.

EXPERIMENTAL NOTES

Shalek & Love Labs, MIT

Membrane Sealing

1. Gather the following materials before sealing the array(s):

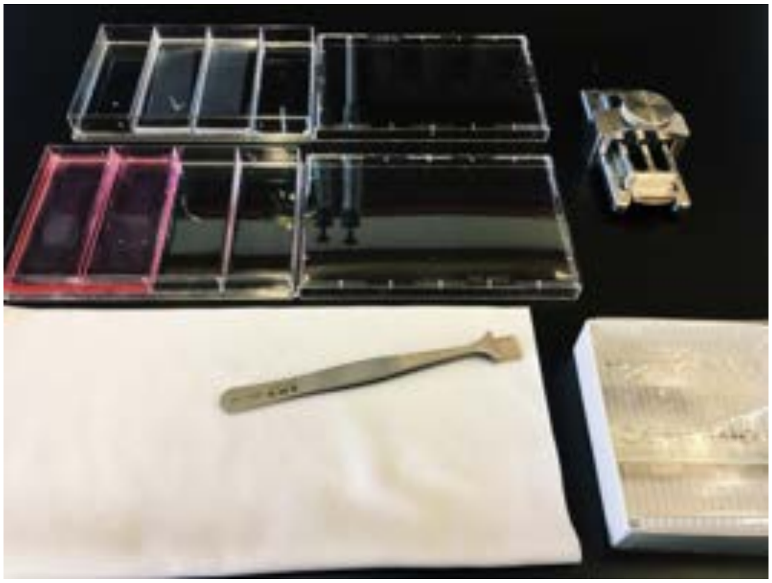

- Array loaded with beads and cells (See Bead/Cell Loading)
- Pre-treated membrane (See Membrane Preparation)
- Wafer forceps (or P1000 pipette tip)
- Paper towels
- Agilent clamp
- Clean microscope slides

2. Use the wafer forceps to transfer the array from media to the lid of a 4-well dish, being careful to ensure that the array is not tilted.

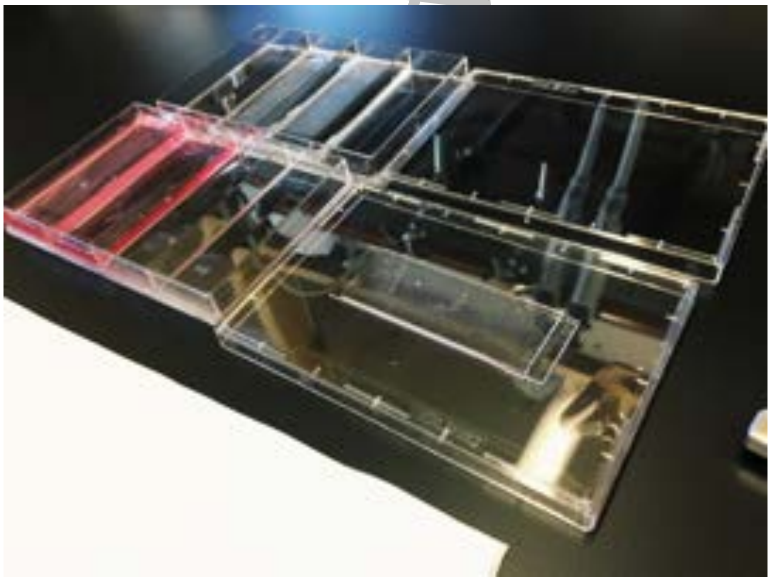

Love Labs, MIT

3. Once the array is positioned on the lid of a 4-well dish, carefully aspirate excess liquid from around the edge of the array and the exposed surface of the glass slide. (Note: Be careful not to aspirate directly from the PDMS surface).

4. Using wafer forceps or a pipette tip, remove a pre-treated membrane from the 4-well dish.

5. Gently dab away moisture from the glass slide on the paper towel until the membrane does not spontaneously change position on the glass slide.

6. Carefully position the membrane on the center of the microscope slide, leaving a small membrane overhang (2-3 mm) beyond the edge of slide.

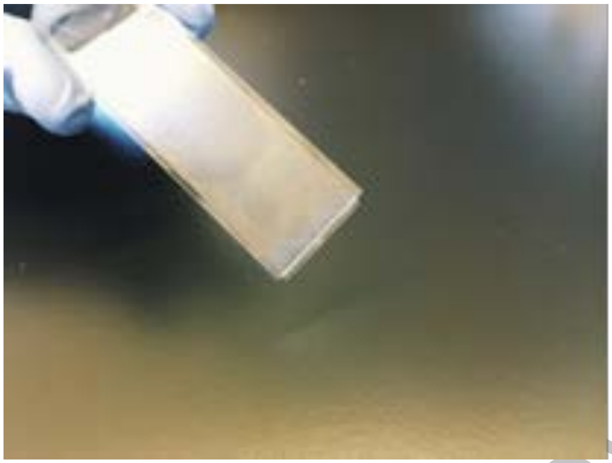
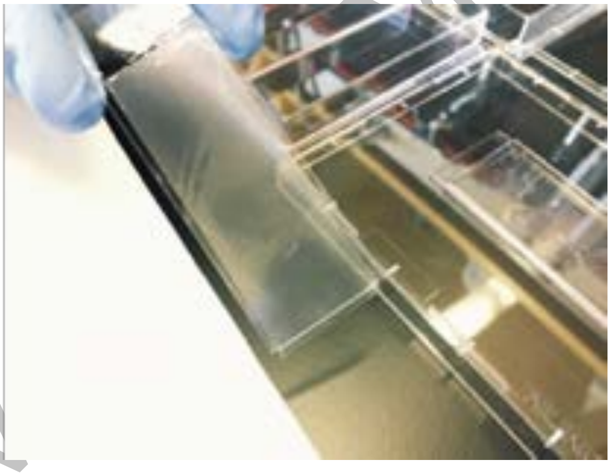

7. Holding the membrane in your left hand, invert the microscope slide so that the treated surface of the membrane is facing down.

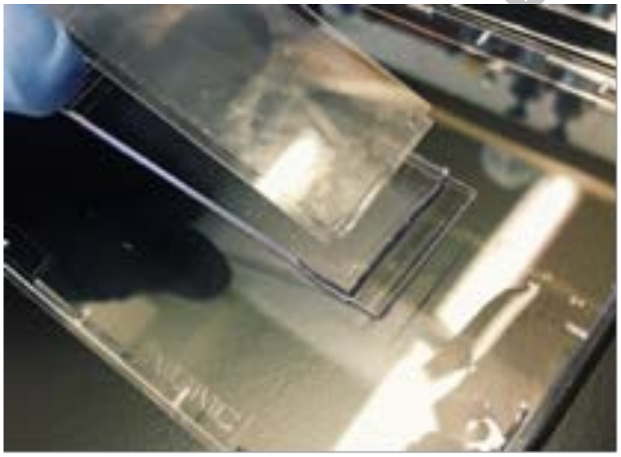
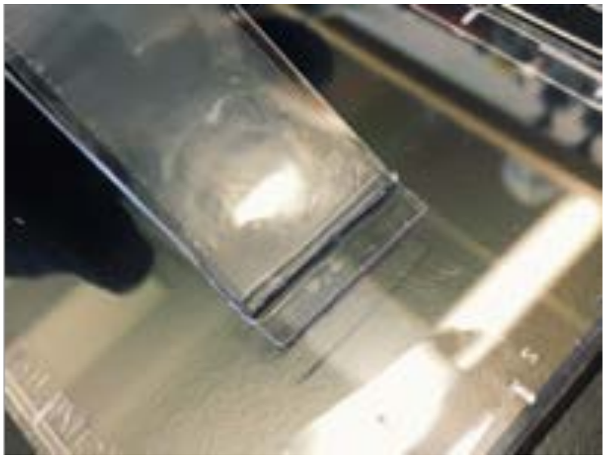

EXPERIMETNAL NOTES

8. Place the overhang of the membrane in contact with the PDMS surface of the array just beyond the boundary of the microwells.

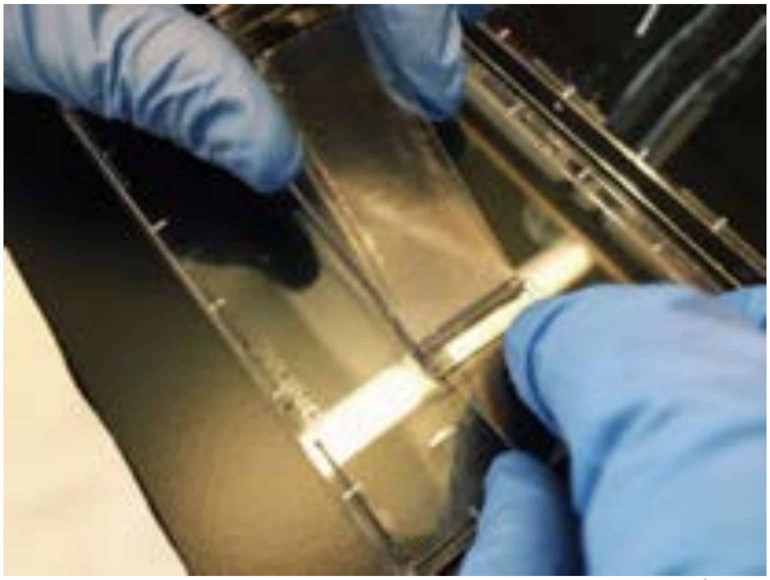

9. Using a clean slide held in your right hand, firmly hold down the overhang of the membrane against the PDMS surface of the array.

10. **Critical Step:** While maintaining pressure with your right hand to hold the membrane in place, gently apply the membrane.
Note 1: For optimal results, use only the weight of the slide to apply the membrane with the left hand.
Note 2: Attempts to manually seal the microwell device using excess pressure result in a ‘squeegee’ effect, effectively removing moisture from the membrane while fixing membrane creases in place.
Note 3: As you apply the membrane you should see a fluid interface form and expand as direct, uniform contact between the slide and the array will naturally remove some of the media as the membrane is applied.
Note 4: You can use either your left or right hand for membrane-sealing (most people use their dominant hand to apply the membrane). Please practice this step before the actual experiment to figure out which hand you’re most comfortable with.

11. After applying the membrane, carefully pry the array and membrane from the surface of the lid and transfer to an Agilent clamp.

12. After transferring the sealed array to the clamp, place a glass slide on top of the sealed array.

Shalek & Love Labs, MIT

13. Close the clamp and tighten to the point of resistance, then place it in a 37C incubator for 30-40 minutes.
    Note: This time is flexible and depends on the incubator. If you want to decrease this incubation time, please optimize on cell lines before proceeding with precious samples.

14. Repeat membrane-sealing protocol procedure if running multiple arrays.

EXPERIMETNAL NOTES

Shalek & Love Labs, MIT

Cell Lysis & Hybridization

1. Remove the clamp from the incubator, and then remove the array from the Agilent clamp. (Note: At this point, the glass slide will be attached to the array and membrane).

2. Submerge the array, with top slide still attached, in 5 mL of complete lysis buffer (**See Appendix D: Buffers Guide**).

3. Gently rock the array in lysis buffer until the top glass slide spontaneously detaches.
   Note 1: Do not pry the top slide off as this can reverse membrane sealing. The time necessary for detachment of the top slide varies (10 seconds – 10 minutes).
   Note 2: If the top slide does not release after 10 minutes, gently pry the top slide off using wafer forceps or a pipette tip. Just be careful.

4. Once the top slide has detached, place the arrays on a horizontal rotator for 20 minutes at 50-60 rpm.

5. After 20 minutes, remove the lysis buffer and wash each array with 5 mL of hybridization Buffer (**See Appendix C: Buffers Guide**).
   Note 1: Use a separate waste container for lysis buffer because guanidine thiocyanate can react with bleach in TC traps to create cyanide gas.
   Note 2: The hybridization buffer used to wash the array post-lysis may contain trace amounts of guanidine thiocyanate and should, therefore, be disposed of in the lysis buffer waste container.

6. Aspirate hybridization buffer and add another 5 mL of hybridization buffer to each array and rotate for 40 minutes at 50-60 rpm.

7. While the arrays are rocking in hybridization buffer, prepare RT master mix. (See **Reverse Transcription & Exonuclease Digestion**)

EXPERIMENTAL NOTES

Shalek & Love Labs, MIT

Bead Removal Method 1

1. After the arrays have rocked in hybridization buffer for 40 minutes, carefully peel back each membrane using fine-tipped tweezers.

2. Place array into a 50 mL conical containing 30-40 mL of Wash 1 solution.

3. Holding the array above the 50mL conical (shown below), repeatedly dispense approximately 1 mL of Wash 1 solution from the conical across the surface of the array to dislodge beads (**See Appendix D: Buffers Guide**).
   Note: Vigorously dispense Wash 1 buffer to remove beads.

4. Repeat these 10 times, periodically checking to see if beads are dislodging.

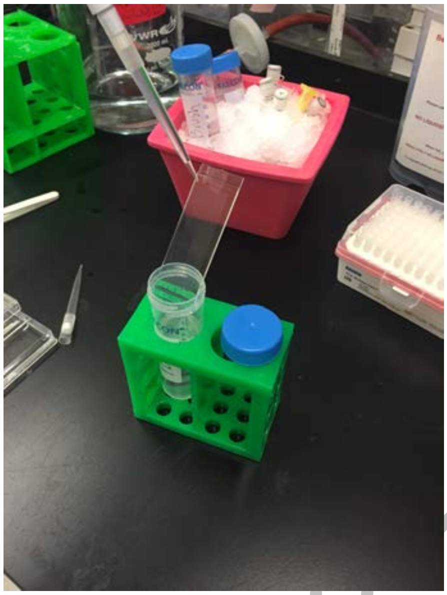

5. After repeatedly rinsing the array from top to bottom, use a clean glass slide to ***gently*** scrape the array to remove any beads that remain in the array.
   Note: At this point it is possible to visually inspect the array to assess bead removal.

6. Once you are satisfied with bead removal, place the empty array back in the 4-well disk, cap the 50 mL conical, and pellet beads for 5 minutes at 1000xg.
   Note 1: You can visually inspect the success of your bead removal by looking at the arrays under a light microscope.
   Note 2: Where possible, use a swinging bucket centrifuge to collect beads. The use of a fixed-rotor centrifuge can lead to the formation of a bead pellet on the elbow rather than the bottom of the conical tube, which can lead to inefficient recovery.

7. After centrifugation, aspirate all but ~1 mL of excess Wash Buffer, collect the beads using a P1000 pipette, and transfer beads suspended in wash buffer to a separate 1.5 mL eppendorf tube for each array.

Shalek & Love Labs, MIT

Reverse Transcription & Exonuclease Digestion

Reverse Transcription (RT)

1. Prepare the following RT mastermix during the hybridization step:

40 uL	H_2O
40 uL	Maxima 5X RT Buffer
80 uL	30% PEG8K
20 uL	10 mM dNTPs (Clontech)
5 uL	RNase Inhibitor (Lucigen)
5 uL	100 uM Template Switch Oligo
10 uL	Maxima H-RT

Note: Add the Maxima H-RT enzyme to the mastermix immediately before adding to beads.

2. Centrifuge eppendorf tubes containing collected beads for 1 minute at 1000xg.

3. Remove supernatant and resuspend in 250 uL of 1X Maxima RT Buffer and centrifuge beads for 1 minute at 1000xg.

4. Aspirate 1X Maxima RT Buffer and resuspend beads in 200 uL of the RT mastermix.

5. Incubate at room temperature for 30 minutes with end-over-end rotation. After 30 minutes, incubate at 52C for 90 minutes with end-over-end rotation.
   Note: The reverse transcription reaction can proceed overnight, if necessary.

7. Following the RT reaction, wash beads once with 500 uL of TE-SDS, and twice with 500 uL of TE-Tween (TE-TW). **Following Reverse Transcription, beads can be stored at 4C in TE-TW.**

Exonuclease I Treatment

1. Prepare the following Exonuclease I Mix:

20 uL	10x ExoI Buffer
170 uL	H_2O
10 uL	ExoI

2. Centrifuge beads for 1 minute at 1000xg and aspirate the TE-TW solution.

3. Resuspend in 500 uL of 10 mM Tris-HCl pH 8.0.

4. Centrifuge beads again, remove supernatant and resuspend beads in 200 uL of exonuclease I mix.

5. Incubate at 37C for 50 minutes with end-over-end rotation.

6. Wash the beads once with 500 uL of TE-SDS, twice with 500 uL TE-TW.
   Beads can be stored at 4C in TE-TW.

Shalek & Love Labs, MIT

Second Strand Synthesis & PCR

Second Strand Synthesis (*Beginning after 2nd wash of TE-TW after Exo treatment)*

1. Prepare the following 2nd strand synthesis mix:

40 uL	Maxima 5X RT Buffer
80 uL	30% PEG8000
20 uL	10 mM dNTPs (Clontech)
2 uL	1 mM dN-SMRT oligo
5 uL	Klenow Enzyme
53 uL	H_2O

Note: Add the Klenow enzyme immediately before adding to beads.

2. After aspiration of 2nd TE-TW wash, resuspend beads in 500 uL 0.1 M NaOH.
Note: Make the 0.1 M NaOH solution fresh each time you perform second strand synthesis.

3. Rotate tube for 5 min at room temp, then spin (800xg for 1 minute) and aspirate supernatant.

4. Wash once with 500 uL of TE-TW, and once with 500 uL 1xTE

5. Resuspend beads in 200 uL 2nd strand synthesis reaction and rotate end-over-end at 37C for 1 hr.

6. Wash beads twice with 500 uL TE-Tween and once with 500 uL TE

7. Proceed directly with the PCR protocol.

PCR (Whole Transcriptome Amplification (WTA))

1. Prepare the following PCR mastermix:

25 uL	2X KAPA HiFi Hotstart Readymix
14.6 uL	H_2O
0.4 uL	100 uM SMART PCR Primer
40 uL	per reaction

2. Wash beads once with 500 uL of water, pellet beads, remove supernatant and resuspend in 500 uL of water.
Note 1: If you do not want to count the beads then after the 500 uL water wash in step 2, resuspend the beads in 240 uL of water and proceed to step 6.
Note 2: If you choose this path, prepare mastermix for 24 PCR reactions for each array being processed.

Shalek & Love Labs, MIT

3. Mix well (do not vortex) to evenly resuspend beads and transfer 20 uL of beads to a separate 1.5 mL tube to count the beads.
Note: Don’t vortex beads as this can result in bead fragmentation.

4. Pellet the small aliquot of beads, aspirate the supernatant, and resuspend in 20 uL of bead counting solution (10% PEG, 2.5 M NaCl).
Note: The bead counting solution aids in even dispersion of beads across a hemocytometer.

5. Count the beads using a hemocytometer.

6. Add 40 uL of PCR mastermix per reaction to 96-well plate.

7. Add 1,500 – 2,000 beads per reaction in 10 uL of water for a total volume of 50 uL per PCR reaction, making certain to PCR the entire array.

8. Use the following cycling conditions to perform whole-transcriptome amplification:

**Start:**	
95C	3 minutes
**4 Cycles:**	
98C	20 seconds
65C	45 seconds
72C	3 minutes
**9-12 Cycles:**	
98C	20 seconds
67C	20 seconds
72C	3 minutes
**Final Extension:**	
72C	5 minutes
4 C	Infinite hold

Note: The total number of PCR cycles necessary for amplification depends on the cell type used.
- 13 cycles are optimal for cell lines or larger cells (e.g. macrophages)
- 16 cycles are optimal for primary cells

EXPERIMENTAL NOTES

Shalek & Love Labs, MIT

Purification of PCR products and analysis on the BioAnalyzer or Agilent TapeStation

1. Pool PCR products from between 6 and 8 PCR reactions in a 1.5 mL microcentrifuge tube so that you have 10-12,000 beads/1.5 mL microcentrifuge tube.

2. Purify PCR products using Ampure SPRI beads and the following protocol:
   Note: Please refer to the Ampure SPRI bead official protocol for more details.
   A. Spri at 0.6x volumetric ratio.
   B. Allow the tubes to sit on the tube-rack off the magnet for 5 minutes, and then place the rack on the magnet for 5 minutes.
   C. Perform 3 washes with 80% ethanol.
   Note: At each wash step rotate each tube 180 degrees 6 times to allow beads to pass through the ethanol solution to the opposite side of the tube.
   D. After the third wash, remove the 80% ethanol wash solution. Further, use a P200 with fresh tips to remove any residual ethanol and allow beads to dry for 10-15 minutes. (**Note**: Beads will have a cracked appearance once dry). Remove the rack from the magnet, elute dried beads in 100 uL, place the rack on the magnet and then transfer the 100 uL supernatant which contains eluted DNA to a new 1.5 mL microcentrifuge tube or 96-well plate.
   E. Spri the 100 uL at 1.0x volumetric ratio and repeat steps b and c
   F. After the third wash, allow the beads to dry for 15 minutes, remove the rack from the magnetic, elute the beads in 15 uL, place the rack back on the magnet and then transfer the 15 uL to a new 1.5 mL microcentrifuge tube or 96-well plate.

3. Run a BioAnalyzer High Sensitivity Chip or Agilent D5000 High Sensitivity Screentape according to the manufacturer's instructions.
   Use 1 uL of the purified cDNA sample as input.
   - Your WTA library should be fairly smooth, with an average bp size of 0.7-2 kbps.

4. Proceed to library preparation or store the WTA product at 4C (short-term) or -20C (long-term).

EXPERIMENTAL NOTES

Shalek & Love Labs, MIT

Library Preparation

Tagmentation of cDNA with Nextera XT

1. Ensure your thermocyclers are setup for Tagmentation (step 5) & PCR (step 9).

2. For each sample, combine 1000 pg of purified cDNA with water in a total volume of 5 uL. It's ideal to dilute your PCR product in a separate tube/plate so that you can add 5 uL of that for tagmentation.
   Example: For 1000 pg reactions, dilute PCR product, in a new plate, to 200 pg/uL, then you can add 5 uL of this to a reaction tube for a 1000 pg reaction.
   Note 1: We typically perform Nextera reactions in duplicate for WTA product from each pool of 6-8 PCR reactions. For example, if you recover 3 pools/array, you would run a total of 6 nextera reactions.
   Note 2: These volumes can be reduced by half to reduce reagent costs, if desired.

3. To each tube, add 11 uL of Nextera TD buffer, then 4 uL of ATM buffer (the total volume of the reaction is now 20 uL).

4. Mix by pipetting ~5 times. Centrifuge plate at 1000x g for 10-15 seconds.

5. Incubate at 55C for 5 minutes.

6. Add 5 uL of Neutralization Buffer. Mix by pipetting ~5 times. **Note:** Bubbles are normal.

7. Incubate at room temperature for 5 minutes.

8. Add to each PCR tube:

15 uL	Nextera PCR mix
8 uL	H_2O
1 uL	10 uM New-P5-SMART PCR hybrid oligo
1 uL	10uM Nextera N7XX oligo

9. After sealing and centrifuging (1 minute at 1000xg) the PCR plate, run the following PCR program:

**Start:**	
72C	3 minutes
95C	30 seconds
**12 cycles:**	
95C	10 seconds
55C	30 seconds
72C	30 seconds
**Final Extension:**	
72C	5 minutes
4C	Infinite hold

Shalek & Love Labs, MIT

Purification of PCR products and analysis on the BioAnalyzer or Agilent TapeStation

1. Pool PCR products from between 6 and 8 PCR reactions in a 1.5 mL microcentrifuge tube so that you have 10-12,000 beads/1.5 mL microcentrifuge tube.

2. Purify PCR products using Ampure SPRI beads and the following protocol:
   Note: Please refer to the Ampure SPRI bead official protocol for more details.
   A. Spri at 0.6x volumetric ratio.
   B. Allow the tubes to sit on the tube-rack off the magnet for 5 minutes, and then place the rack on the magnet for 5 minutes.
   C. Perform 3 washes with 80% ethanol.
      Note: At each wash step rotate each tube 180 degrees 6 times to allow beads to pass through the ethanol solution to the opposite side of the tube.
   D. After the third wash, remove the 80% ethanol wash solution. Further, use a P200 with fresh tips to remove any residual ethanol and allow beads to dry for 10-15 minutes. (**Note**: Beads will have a cracked appearance once dry). Remove the rack from the magnet, elute dried beads in 100 uL, place the rack on the magnet and then transfer the 100 uL supernatant which contains eluted DNA to a new 1.5 mL microcentrifuge tube or 96-well plate.
   E. Spri the 100 uL at 1.0x volumetric ratio and repeat steps b and c
   F. After the third wash, allow the beads to dry for 15 minutes, remove the rack from the magnetic, elute the beads in 15 uL, place the rack back on the magnet and then transfer the 15 uL to a new 1.5 mL microcentrifuge tube or 96-well plate.

3. Run a BioAnalyzer High Sensitivity Chip or Agilent D1000 High Sensitivity Screentape according to the manufacturer's instructions.
   - Use 1 uL of the purified cDNA sample as input.
   - Your tagmented library should be fairly smooth, with an average bp size of 400-800 bp.
   - Smaller-sized libraries might have more polyA reads
   - Larger libraries may have lower sequence cluster density and cluster quality.

Note: We have successfully sequenced libraries from 400-800bp.

5. Proceed to sequencing.

Shalek & Love Labs, MIT

Sequencing

Once your sequencing library has passed the proper quality controls, you're ready to proceed to sequencing. For a detailed loading protocol, please consult the Illumina website for a step-by-step manual. (https://support.illumina.com/downloads.html)

NextSeq500 – Shalek Lab protocol

1. Make a 5 uL library pool at 4 nM as input for denaturation.
2. To this 5 uL library, add 5 uL of 0.2 N NaOH (make this solution fresh).
3. Flick to mix, then spin down and let tube sit for 5 minutes at room temperature.
4. After 5 minutes, add 5 uL of 0.2 M Tris-HCl pH 7.5.
5. Add 985 uL of HT1 Buffer to make a 1 mL, 20 pM library (solution 1).
6. In a new tube (solution 2), add 165 uL of solution 1 and dilute to 1.5 mL with HT1 buffer to make a 2.2 pM solution – this is the recommended loading concentration.
   Note: Optimal loading concentration is 1.8-2.5 pM
7. Follow Illumina's guide for loading a NextSeq500 Kit

Sequencing specifications for the MiSeq or NextSeq:

Read 1: 20 bp *

Read 2: 50 bp

Read 1 Index: 8 bp ← *only necessary if you are multiplexing samples*

Custom Read 1 primer

Sequencing specifications for the Nova-Seq:

Read 1: 20 bp *

Read 2: 50-80 bp

Read 1 Index: 8 bp

Read 2 Index: 8 bp (optional, but recommended)

Custom Read 1 primer

Note 1: If you're loading on a Nova-Seq you'll want to use dual-indexing to mitigate index switching.

Note 2: Read 1 can sometimes be 21 base pairs; this depends on the company and bead lot you are ordering from. Please consult with your bead provider to determine which read length to use.

NextSeq 500:
(http://support.illumina.com/content/dam/illumina-support/documents/documentation/system_documentation/nextseq/nextseq-custom-primers-guide-15057456-01.pdf)
(Follow Illumina's guide for custom primers)
MiSeq:
(http://support.illumina.com/content/dam/illumina-support/documents/documentation/system_documentation/miseq/miseq-system-custom-primers-guide-15041638-01.pdf)

Shalek & Love Labs, MIT

Appendix A: Array Synthesis

Day 0: Pouring PDMS Arrays

Note: If you need to mount your master, please refer to **appendix F**

1. Combine Sylgard crosslinker with Sylgard base at a 1:10 ratio and mix vigorously for 5 minutes to create a PDMS master mix.

2. Once mixing is complete, put your PDMS master mix under vacuum for 20 minutes to remove any air bubbles.

3. Use a 10 mL syringe to inject 6-10 mL of PDMS master mix into molds with mounted PDMS masters.

4. Incubate at 70C for 2.5 hours.

Day 1: Array Functionalization Part 1

Note: For this section, make all solutions fresh!

1. Remove excess PDMS from edges of the glass slide.

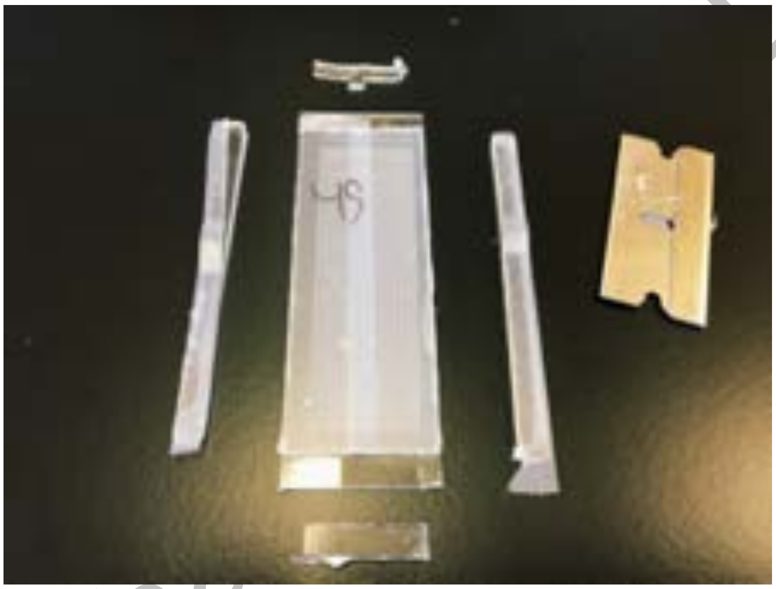

2. Use scotch tape to remove excess PDMS from the surface of the array and the glass slide.

Sha[illegible] Labs, MIT

3. Place clean arrays into a metal slide basket

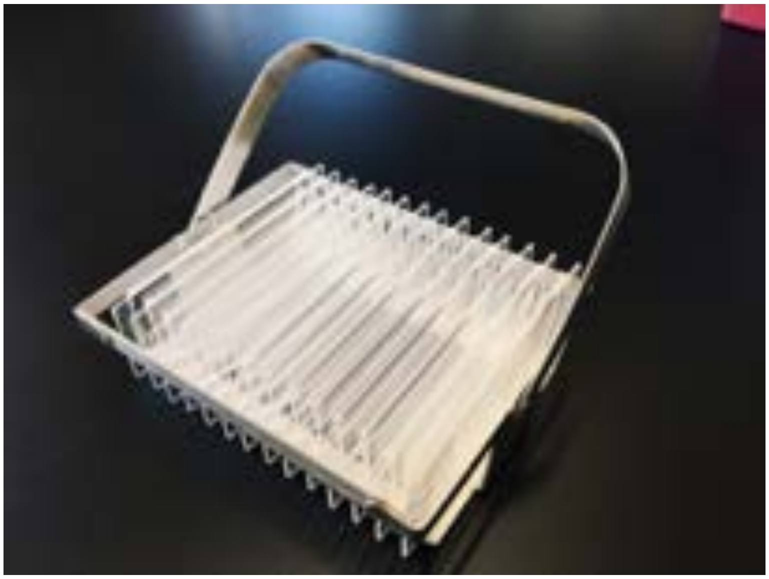

4. Rinse arrays in 100% ethanol for 5 minutes, then let dry at room temperature (RT) for 15 minutes.

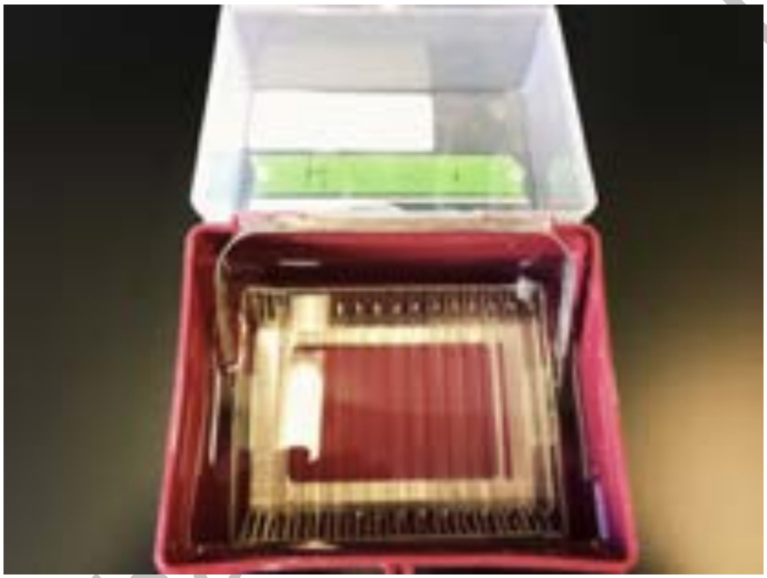

5. Plasma treat arrays on high for 5-7 minutes.
Note 1: Adjust the air valve so that the plasma is pink.

6. Following plasma treatment, immediately submerge arrays in 350 mL of 0.05% APTES in 95% ethanol for 15 minutes.

7. Spin dry arrays **(500 RPM** for 1 minute).
Note: Our rotor model is TX-10000 75003017 (Thermo) with a rotor radius of 209 mm. 500 RPM on this instrument is ~ 60xg.

Sh[illegible] Labs, MIT

8. Incubate at room temperature for 10 minutes.

9. Submerge in 300 mL of acetone and rock until all bubbles are out of the wells; this typically takes approximately 5 minutes.

10. Place in 350 mL of 0.2% PDITC/10% pyridine/90% DMF solution in a glass chamber (or polypropylene tip box) for 2 hours at room temperature.
Note: While this is rocking, prepare your chitosan solution (See **Appendix D**)

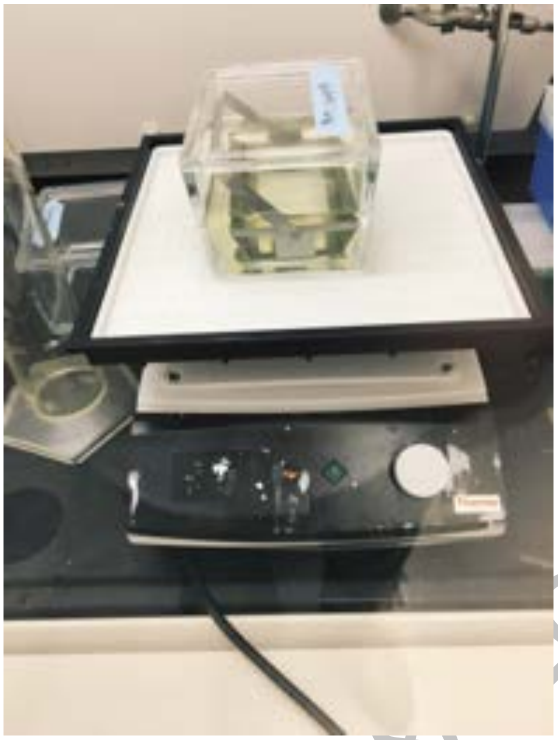

11. After the PDITC soak, wash arrays briefly in two boxes of 300 mL DMF.
Note: For each brief wash, simply dunk the arrays in the solution 5-10 times and then transfer to the new solution.

12. Dunk and wash the arrays in 300 mL of acetone.

13. Move to a fresh 350 mL of acetone and rock for 20 minutes.

14. Spin dry arrays (**500 RPM** for 1 minute).

15. Place arrays at 70C for 2 hours.

16. Remove from oven and let sit at room temperature for 20 minutes.

17. Submerge arrays in 350 mL of 0.2% chitosan solution (pH 6.0-6.1; See Appendix D) and incubate at 37C for 1.5 hours.

Shalek & Love Labs, MIT

18. Wash arrays 4x in separate 300 mL distilled water baths.

19. Submerge in 350 mL of 20 ug/mL aspartic acid, 2 M NaCl, and 100 mM sodium carbonate solution (pH 10.0)

20. Place in vacuum chamber and apply house vacuum.
Note: You should see bubbles form indicating the solvation of wells.

21. Place vacuum chamber (still connected to house vacuum) on a rocker and rock (50-70 RPM) overnight at room temperature.

Day 2: Array Functionalization – Part 2

1. The following morning, remove arrays from vacuum and rotate at 50-60 RPM for 3 hours at room temperature.

2. Place arrays at 4C and soak 24 hours before use.
Note: Arrays can be stored in the aspartic acid solution for 3 months at 4C.

EXPERIMENTAL NOTES

Shalek & Love Labs, MIT

Appendix B: Synthesis Protocol Checklist

Date:

Synthesizer:

Number of Arrays:

Start time:

End time:

Before you start:

1. Pull the PDITC from the fridge (this takes ~1hr to come to room temperature)
2. Make certain you have enough boxes for the various incubations
3. Clean a 1L bottle, add stir bar, and dissolve 1 gram of chitosan in 500mL of DI water

Step 1: Plasma treatment of the arrays

1. Soak the arrays in 300mL of 95% ethanol for 5 minutes (50 rpm)
2. Dry the arrays for 5 minutes @ 500 rpm (60xg)
3. Plasma treat **two** trays at a time
   A. Form seal for **3 minutes**
   B. Plasma treat for **5-7 minutes**.
   C. What color was the plasma? (circle one):
      No Color Light purple Light pink
   D. While the arrays are being treated, prep the APTES solution
      APTES Solution: 180uL of APTES stock in 350mL of 95% ethanol
4. Proceed with protocol

Step 2: PDITC Soak

***Autoclave the chitosan solution after starting the PDITC incubation

1. Volume of PDITC solution you're prepping:________________liters (Standard: 350 mL)
2. Mass of PDITC added:________________________________grams (Standard: 0.72 grams)
3. Volume of pyridine added:________________________________liters (Standard: 35 mL)
4. Length of incubation:___________________________________hours (Standard: 2 hours)
5. Number of DMF washes:___________________________________(Standard: 2 washes)
6. Number of acetone washes:___________________(Standard: 1 wash, and then transfer to new acetone box for a **20-minute** soak)
7. Proceed with protocol

Shalek & Love Labs, MIT

Step 3: Oven incubation and chitosan preparation

1. After **20-minute soak**, remove arrays from acetone and spin down (1 min. @ 500 rpm)
2. Length of 70C incubation:________________________________hrs (Standard: 2 hrs)
3. Chitosan protocol checklist: Did you… (Y / N responses)
   A. Autoclave chitosan (40-minute sterilization, 20-minute drying):_______________
   B. Let solution come to room temperature (can also do this @ 4C):______________
   C. Calibrate the pH Meter with appropriate buffers:_________________________
   D. Add 4 mL of glacial acetic acid (solution should be on stir plate):_____________
   E. Let solution stand for **5 minutes**:___________________________________
   F. Add 50 mL of 5 M NaCl:_______________________________________
   G. Titrate with 5 M NaOH:_______________________________________
   H. Achieve pH of 6.0 – 6.1:_______________________________________
   I. Remember, it is **critical** to make certain the you achieve a pH of 6.0 – 6.1 and that it holds. Parts A-H should be completed **before** the completion of the 2 hr 70C incubation.
4. Length of room temperature incubation:_______________minutes (Standard: 20 minutes)
   A. Second check of chitosan pH:
5. Length of chitosan incubation:______________________________(Standard: 1.5 hrs)
   A. Temperature = 37C, Rotation = 70 rpm

Step 4: In-well functionalization

1. Number of DI water rinses:_____________________________________(Standard: 4)
2. pH of aspartic acid solution:_________________________________(Standard: pH 10)
3. Length of overnight incubation:____________________________(Standard: 12-16 hrs)
4. Day 2: length of room temperature incubation @ 50 rpm:___________(Standard: 4 hrs)

Array Lot: <Your initials>_<Synthesis Date>_<Box Number>

Shalek & Love Labs, MIT

Appendix C: Master Mounting Protocol

1. Mix and degas PDMS in normal 1:10 ratio

2. While PDMS degases, use sandpaper to gently score back of silicon master and base plate to improve adhesion. Careful – silicon masters are brittle.

3. Wash back of master and base plate with 95% ethanol until no more dust is removed when wiping surface clean with paper towel.

4. Use gloved finger to spread vacuum grease on bottom of BasePlate2 around square holes where the nanowell arrays will be cast. You want a relatively thick layer, even on skinny parts between array holes, to make sure there is a seal between master and plate.

5. Carefully lower BasePlate2 onto the array side of the master making sure to not touch the array area with any of the greased surface. 4 array masters should fit into the 4 square holes. Gently slide plate against master to center the arrays.

6. Place Base Plate 1 on paper towels to catch PDMS running off plate.

7. Pour ~30 mL of mixed PDMS in center of Base Plate 1.

8. Place master/BasePlate2 sandwich on top of the PDMS.

9. Gently apply pressure in the center of the master while making circular motions to push PDMS out from between layers. You want to see PDMS coming out of all sides to ensure a complete coat.

10. Screw 6/32 screws into respective holes on base plate very gently. Too much pressure too fast may crack master. Do not fully tighten. Do your best to make screws even – look at width of crack between base plates on all sides and make equal.

11. Place both top plates on top.

12. Screw 10/24 screws into their holes just enough such that they catch. Again, do not fully tighten.

13. Place in 90C oven for 3 hours.

14. May need to do one dummy round of arrays to remove any PDMS or grease that got onto the nanowell features.

Shalek & Love Labs, MIT

Appendix D: Buffers Guide

CellCover10

Reagents

- CellCover (Anacyte Art. No. 800-125)
- FBS (Thermo Fisher Scientific Cat. No. 10437028)
- Sodium Carbonate (Sigma Cat. No. 223530-500G)

Working Concentrations

- 10% FBS
- 100 mM Sodium Carbonate

Bead Loading Buffer

Reagents

- Sodium Carbonate (Sigma Cat No. 223530-500G)
- BSA (Sigma Cat No. A9418-100G)
- Water (Thermo Fisher Scientific Cat No. 10977023)

Quick Preparation Guide (50 mL)

1. 2.5 mL 2 M Sodium Carbonate
2. 42.5 mL H_2O
3. Add 5 mL BSA (100 mg/mL)
4. Titrate with glacial acetic acid to achieve a pH of 10.0

Working Concentrations

- 100 mM Sodium Carbonate
- 10% BSA

Complete Lysis Buffer

Reagents

- Pre-lysis buffer
- 10% Sarkosyl (Sigma Cat No. L7414)
- 100% 2-Mercaptoethanol (Sigma Cat No. M3148-25ML)

Quick Preparation Guide (50 mL)

1. 47.25 mL Pre-Lysis Buffer
2. 250 uL 10% Sarkosyl
3. 500 uL BME

Working Concentrations

- 5 M Guanidine Thiocyanate
- 1 mM EDTA
- 0.50% Sarkosyl
- 1.0% BME

Shalek & Love Labs, MIT

Hybridization Buffer

Reagents
- 5 M NaCl (Thermo Fisher Scientific Cat No. 24740011)
- 1x PBS (Thermo Fisher Scientific Cat No. 10010023)
- 8% (v/v) PEG8000 (Sigma Cat No. 83271-500ML-F)

Quick Preparation Guide (50 mL)
1. 20 mL 5 M NaCl
2. 26 mL of PBS
3. 4 mL PEG8000

Working Concentrations
- 2 M NaCl

Wash Buffer

Reagents
- 5 M NaCl (Thermo Fisher Scientific Cat No. 24740011)
- 1 M $MgCl_2$ (Sigma Cat No.63069-100ML)
- 1 M Tris-HCl pH 8.0 (Thermo Fisher Scientific Cat No. 15568025)
- Water (Thermo Fisher Scientific Cat No. 10977023)
- 8% (v/v) PEG8000 (Sigma Cat No. 83271-500ML-F)

Quick Preparation Guide (50 mL)
1. 20 mL 5 M NaCl
2. 150 uL 1 M $MgCl_2$
3. 1 mL 1 M Tris-HCl pH 8.0
4. 24.85 mL H_2O
5. 4 mL PEG8000

Working Concentrations
- 2 M NaCl
- 3 mM $MgCl_2$
- 20 mM Tris-HCl pH 8.0

Array Quenching Buffers

Reagents
- Sodium Carbonate (Sigma Cat No. 223530-500G)
- 1 M Tris-HCl pH 8.0 (Thermo Fisher Scientific Cat No. 15568025)
- Water (Thermo Fisher Scientific Cat No. 10977023)

Quick Preparation Guide (50 mL)
1. 2.5 mL 2 M Sodium Carbonate
2. 500 uL 1 M Tris-HCl pH 8.0
3. 47 mL H_2O

Working Concentrations
- 100 mM Sodium Carbonate
- 10 mM Tris-HCl pH 8.0

Shalek & Love Labs, MIT

0.2% Chitosan Solution

Reagents

- Chitosan (Sigma Cat No. C3646-100G)
- Water (Thermo Fisher Scientific Cat No. 10977023)

Quick Preparation Guide

1. Add 1 gram of chitosan to 500 mL of DI water
2. Autoclave solution (40 minutes sterilization, 20 minutes dry)
3. Allow chitosan solution to come to room temperature, and then add 2-3 mL of glacial acetic acid.

Note: The chitosan will not start dissolving until the pH is acidic, and even then it will not fully dissolve. This is ok.

4. Add 50 mL 5 M NaCl, then titrate the chitosan solution with NaOH to bring the pH to 6.2.

TE - Tween Storage Solution

- 10 mM Tris pH 8.0 + 1 mM EDTA
- 0.01% Tween-20

Quick Preparation Guide (50 mL)

1. 49.95 mL H_2O
2. 5 uL Tween-20

TE - SDS Solution

- 10 mM Tris pH 8.0 + 1 mM EDTA
- 0.5% SDS

Quick Preparation Guide (50 mL)

1. 49.75 mL H_2O
2. 250 uL SDS

Shalek & Love Labs, MIT

Appendix E: Bead Removal Method 2 (“Spin-Out”)

1. Remove membrane and place array into an empty 50 mL conical tube.

2. Ensure that the array is angled within the tube as shown below.
Note: The array might move around at this point, which isn’t something to worry about.

3. Add 48-50 mL of Wash 1 solution (See Buffers Guide)

4. Place the insert so the array is secured angled as shown in the image below.

5. Secure the lid and seal with parafilm, if necessary.

6. Put the sealed conical in a centrifuge, making certain the PDMS surface of the array is facing away from the rotor arm (See Diagram Below).

7. Centrifuge at 2000 x g for 5 minutes to remove the beads.

8. At this point you should see a small, but visible, pellet of beads at the bottom of the tube.

9. Aspirate 5 - 10 mL of Wash 1 solution to enable easier removal of the array.

10. Remove the array and carefully position it over the top of the 50 mL tube.

11. Repeatedly wash any remaining beads from the surface of the array over the surface of the 50 mL falcon tube using 1 mL of Wash 1 remaining in the tube.

12. Spin again at 2000 x g for 5 minutes to pellet beads.

13. Aspirate all wash 1 solution except for ~ 1mL.
Note: Be careful to not disturb the pellet of beads.

14. Transfer beads to a 1.5 mL centrifuge tube and proceed to reverse transcription.

Shalek & Love Labs, MIT

Appendix F: Imaging in Array

1. When pre-imaging cells, cells should be loaded first as beads will obstruct view of many cells and beads autofluorescence can interfere with the signal.

2. Obtain a cell or tissue sample and prepare a single cell suspension using your preferred protocol.

3. Count cells using a hemocytometer and resuspend 10,000 cells in 200 uL of cold CellCover (Anacyte).

4. Incubate cells in at 4C for 1 hour.

5. After the cells have been fixed, perform antibody staining at 4C.
Note: Some epitopes may no longer be available as a result of the fixation process.

6. Wash cells twice with 1x PBS, resuspend in 200 uL of CellCover10 buffer (pH 10 + 10% FBS; **See Appendix D: Buffers Guide**), and place on ice.
Note: CellCover != CellCover10.

7. Obtain empty functionalized array(s), aspirate storage solution and soak each array in 5 mL of CellCover10 buffer (**See Appendix D: Buffers Guide**).

8. Aspirate media and load your fixed cells onto each array in a dropwise format.

9. Gently rock the array(s) in the x & y direction for 5 minutes.

10. Wash each array twice with 5 mL of CellCover10 (pH 10 + 10% FBS), then solvate each them in 5 mL of CellCover (No FBS).

11. Place a lift slip on each array, then image with a microscope.

12. After imaging, wash each array in 5 mL of CellCover10 media.

13. Immediately load beads using the bead loading protocol provided above.
Note: In the protocol provided above, beads are washed and loaded in BLB. When loading cells first, you will replace BLB with CellCover10 for all steps. After beads are loaded and sufficiently washed, you will wash the array 4x with CellCover10 without FBS and solvate arrays in CellCover.

14. Proceed with membrane sealing.

Shalek & Love Labs, MIT

Appendix G: Shopping List

Device Manufacturing

Equipment

- Dow Corning Sylgard 184 Silicone Encapsulant Clear 0.5 kg kit (Part No. 184 SIL ELAST KIT 0.5 PG)
- Protolabs Custom Array Molding Plates (Please refer to www.shaleklab.com/seq-well
  - Make out of aluminum and make sure to tap holes only on base plateBasePlate1 v3.1 (Bottom plate you mount the wafer to)
- BasePlate2 v3.1 (Divider for arrays)
- TopPlate1 v3.1 (Plate that holds the glass slides)
- TopPlate2 v3.1 (Top plate)
- 45 micron Silicon Master Wafer Size (Please refer to www.shaleklab.com/seq-well)
- Master, pre-silanized – (FlowJem, Inc. Toronto, Canada)
- Corning 72x25 Microscope Slides (Corning Life Sciences Cat. No. 2947)
- 6/32 ¼" Hex Screws
- 5/8" Hex 10/24 Screws
- Hex Screwdriver
- Vacuum grease
- 80 grit sandpaper
- 95% ethanol in spray bottle

Array Functionalization

Equipment

- Plasma Oven (Harrick Plasma PDC-001-HP)
- 2x 30-slide rack slotted (VWR Cat No. 25461-014)
- 16x20 cm staining dish (VWR Cat No. 25461-018)
- Vacuum Desiccator (VWR Cat No. 24988-164)
- Sterile 4-well dishes (Thermo Fisher Scientific Cat No. 267061)

Reagents

- 200 proof ethanol (VWR Cat No. 89125-188)
- (3-Aminopropyl)triethoxysilane (Sigma Cat No. A3648)
- Acetone (Avantor Product No. 2440-10)
- p-Phenylene Diisothiocyanate (PDITC) (Sigma Cat No. 258555-5G)
- Pyridine (Sigma 270970-1L)
- Dimethylformamide (DMF) (Sigma Cat No. 227056-1L)
- Chitosan (Sigma Cat No. C3646-100G)
- Poly(L-glutamic) acid sodium solution (Sigma Cat No. P4761-100MG)
- 5M NaCl (Sigma Cat No. S6546-1L)
- Sodium Carbonate (Sigma Cat No. S2127-500G)

Shalek & Love Labs, MIT

Buffer Reagents

Bead Loading Buffer

- Sodium Carbonate (Sigma Cat No. 223530-500G)
- BSA (Sigma Cat No. A9418-100G)
- Water (Thermo Fisher Scientific Cat No. 10977023)

Complete Lysis

- Guanidine Thiocyanate, (Sigma Cat No. AM9422)
- 0.5 M EDTA (Thermo Fisher Scientific Cat No. 15575020)
- Water (Thermo Fisher Scientific Cat No. 10977023)
- 10% Sarkosyl (Sigma Cat No. L7414)
- 100% 2-Mercaptoethanol (Sigma Cat No. M3148-25ML)

Hybridization Buffer

- 5 M NaCl (Thermo Fisher Scientific Cat No. 24740011)
- 1x PBS (Thermo Fisher Scientific Cat No. 10010023)
- PEG-8K (50%) (Fisher Scientific Cat No. BP337-100ML)

Wash Buffer

- 5 M NaCl (Thermo Fisher Scientific Cat No. 24740011)
- 1 M $MgCl_2$ (Sigma Cat No.63069-100ML)
- 1 M Tris-HCl pH 8.0 (Thermo Fisher Scientific Cat No. 15568025)
- Water (Thermo Fisher Scientific Cat No. 10977023)

Array Quenching Buffer

- Sodium Carbonate (Sigma Cat No. 223530-500G)
- 1 M Tris-HCl pH 8.0 (Thermo Fisher Scientific Cat No. 15568025)
- Water (Thermo Fisher Scientific Cat No. 10977023)

RT Reagents

- UltraPure Distilled Water (Thermo Fisher Scientific Cat No. 10977023)
- Maxima 5x RT Buffer/Maxima H-RT (Thermo Fisher Scientific Cat No. EPO0753)
- 20% Ficoll PM-400 (Sigma Cat No. F5415-50mL)
- 10 mM dNTPs (New England BioLabs Cat No. N0447L)
- RNAse Inhibitor (Thermo Fisher Scientific Cat No. AM2696)
- Template Switching Oligo (Order from IDT)

Exonuclease Reagents

- Exonuclease I (*E. coli*) (New England Biolabs Cat No. M0293S)

Second Strand Synthesis Reagents

- Maxima 5x RT Buffer/Maxima H-RT (Thermo Fisher Scientific Cat No. EPO0753)
- 10 mM dNTPs (New England BioLabs Cat No. N0447L)
- dN-SMART Oligo (Order from IDT)
- UltraPure Distilled Water (Thermo Fisher Scientific Cat No. 10977023)
- Klenow Exo- (New England BioLabs Cat No. M0212S)
- 30% PEG8000 (Sigma-Aldrich 89510-1KG-F)

Shalek & Love Labs, MIT

PCR Reagents

- IS PCR Primer (Order from IDT)
- KAPA HiFi Hotstart Readymix PCR Kit (Kapa Biosystems Cat No. KK-2602)

Nextera Reagents

- Nextera XT DNA Library Preparation Kit (96 samples) (Illumina FC-131-1096)
- New-P5-SMART PCR Hybrid Oligo (Order from IDT)
- Nextera N70X Oligo (Order from Illumina)

Operating Equipment

- Polycarbonate (PCTE) 0.01 micron 62x22 mm precut membranes, 100 count (Sterlitech Custom Order)
- mRNA Capture Beads (Chemgenes Cat No. MACOSKO-2011-10)
- Lifter Slips, 25x60mm (Electron Microscopy Science Cat No. 72186-60)
- Agilent Clamps (Agilent Technologies Cat No. G2534A)

Sequences

Barcoded Bead SeqB:
5'–Bead–Linker--TTTTTTTAAGCAGTGGTATCAACGCAGAGTAC-
JJJJJJJJJJJJNNNNNNNNTTTTTTTTTTTTTTTTTTTTTTTTTTTTTT--3'

Template Switching Oligo (TSO):
AAGCAGTGGTATCAACGCAGAGTGAATrGrGrG

dN-Smart Randomer (dN-SMRT):
AAGCAGTGGTATCAACGCAGAGTGANNNGGNNNB

Smart PCR Primer (TSO_PCR):
AAGCAGTGGTATCAACGCAGAGT

New-P5-SMART PCR Hybrid Oligo (P5-TSO_Hybrid):
AATGATACGGCGACCACCGAGATCTACACGCCTGTC-
CGCGGAAGCAGTGGTATCAACGCAGAGT*A*C

Custom Read 1 Primer (Read_1_Custom_SeqB):
GCCTGTCCGCGGAAGCAGTGGTATCAACGCAGAGTAC

Shalek & Love Labs, MIT

Seq-Well Cost Object Breakdown (Non-discounted prices were used in this cost model)

Array Synthesis	Cost (USD)	Per Array (USD)	Part Number
PDMS	109.23	1.37	184 SIL
ProtoLabs Mounts	1100.84	11.01	Protolabs Quote
Silicon Wafer	1070	10.70	FlowJem Quote
Corning Microscope slides	320	0.22	Cat no. 2947
Chitosan	156	0.05	C3646-100G
APTES (100mL)	73.7	0.00	A3648-100ML
Acetone	180.06	1.80	2440-10
PDITC	116	1.29	258555-5G
Pyridine	190	0.45	270970-1L
DMF	321	2.82	319937-4L
L-Aspartic Acid	29	0.03	A9256-100G
5M NaCl	51.2	0.49	S6546-1L
Sodium Carbonate	57.2	0.00	S2127-500G
	**Total (per array)**	30.22	

Bead Synthesis	Cost (USD)	Per Array (USD)	Part Number
**Bead Synthesis**	4500	45	Macosko-Chemgenes
	**Total (per array)**	45.00	

Loading Buffers	Cost (USD)	Per Array (USD)	Part Number
Sodium Carbonate	54.1	0.01	S2127-500G
BSA	641	0.01	A9418-100G
Water	185	0.37	10977023
Guanidine Thiocyanate	284	0.01	AM9422
0.5M EDTA	81.25	0.01	15575020
10% sarkosyl	117	0.01	L7414-10ML
100% BME	80.6	0.01	M3148-250ML
5M NaCL	51.2	0.01	S6546-1L
1xPBS	196	0.98	10010049
1M Mg2Cl	119	0.01	63069-100ML
1M Tris-HCl pH 8.0	69.8	0.1	15568025
	**Total (per array)**	46.53	

Downstream Processing	Cost (USD)	Per Array (USD)	Part Number
**RT**			
Maxima RT Buffer (5X)	680	17.00	EPO0753
PEG8K	105	0.01	F5415-50ML

10 mM dNTP	250	1.25	N0447L
RNAse Inhibitor	448	4.48	AM2696
TSO	IDT Quote	0.01	IDT quote
**Second Strand**			
Klenow	244	6.10	M0212M
10 mM dNTP	250	1.25	N0447L
**PCR**			
Exonuclease digestion	281	0.75	M0293L
ISPCR	IDT Quote	0.01	IDT quote
KAPA HIFI	640.5	61.00	KK-2602
**Nextera**		0.00	
Nextera XT library prep kit	3270	96.18	FC-131-1096
P5 hybrid olgio	IDT Quote	0.01	IDT quote
Nextera N7XX primer	IDT Quote	0.01	IDT quote
**Sequencing QC**			
Tape Station - D5000 screentapes	527	25.00	5067-5592
Tape Station - D5000 Reagents	179	8.50	5067-5593
	**Total (per array)**	221.56	
**Sequencing**			
Nextseq 500 kit	1570		Illumina quote
QC librarys (WTA and NTA)			
*Assume we load 2 arrays/run			

**Seq-Well S^3 Protocol Steps**	**Costs per array**	**10x V3 3' Protocol Steps (BMC)**	**Costs per array**
Array Synthesis	30.22	Setup Costs	1235
Bead Synthesis	45.00	Price per sample	2100
Loading Buffers	46.53	Sequencing	3010
Downstream Processing	221.56		
Sequencing	785.00		
**Seq-Well Pipeline Costs**	**Costs per array**	**10x V3 3' Pipeline Costs**	**Costs per array**
Pre-Seq Costs (w/ synthesis)	343.31	Pre-Seq Costs (w/ setup)	3335
Pre-Seq Costs (w/o synthesis)	313.09	Pre-Seq Costs (w/o setup)	2100
Total Costs (w/ synthesis)	1128.31	Total Costs (w/ setup)	6345
Total Costs (w/o synthesis)	1098.09	Total Costs (w/o setup)	5110
