# Supplementary material for: Second-Strand Synthesis-Based Massively Parallel scRNA-Seq Reveals Cellular States and Molecular Features of Human Inflammatory Skin Pathologies
Source: Immunity. 2020 Oct 13;53(4):878–894.e7. doi: 10.1016/j.immuni.2020.09.015 (PMC7562821; doi:10.1016/j.immuni.2020.09.015)
Supplement: Document S2. Article plus Supplemental Information [file mmc8.pdf]

Resource
Immunity

Second-Strand Synthesis-Based Massively Parallel scRNA-Seq Reveals Cellular States and Molecular Features of Human Inflammatory Skin Pathologies

Graphical Abstract

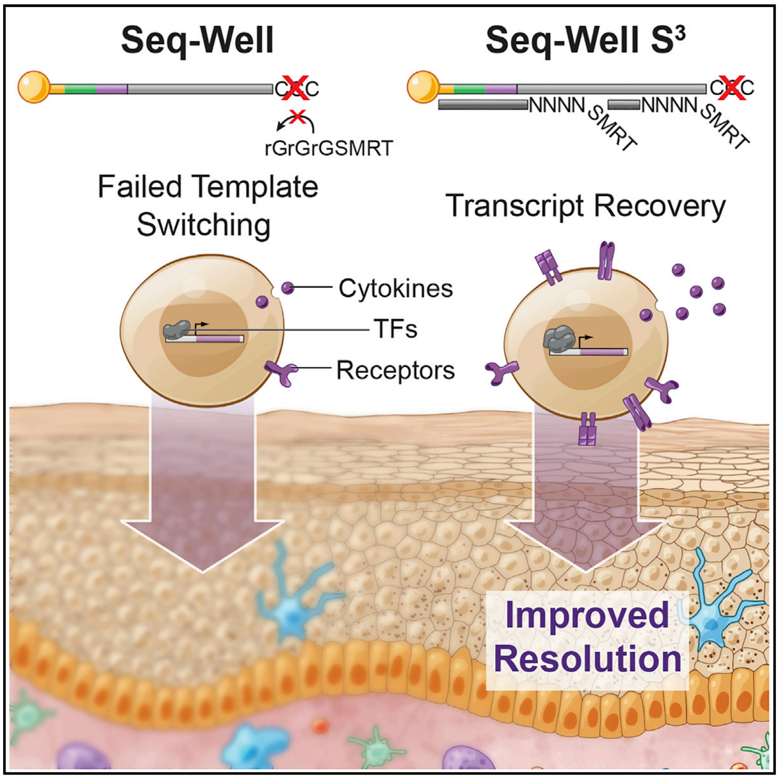

Authors

Travis K. Hughes, Marc H. Wadsworth II, Todd M. Gierahn, ..., Robert L. Modlin, J. Christopher Love, Alex K. Shalek

Correspondence

clove@mit.edu (J.C.L.), shalek@mit.edu (A.K.S.)

In Brief

Hughes et al. report the development of a technique for high-throughput single-cell RNA-sequencing, "Seq-Well S^3," that enables increased sensitivity and improved detection of genes including transcription factors, cytokines, and cytokine receptors. Using Seq-Well S^3, the authors define inflammatory cell states across multiple skin diseases.

Highlights

- Seq-Well S^3 uses second-strand synthesis to improve transcript capture
- Seq-Well S^3 was benchmarked against a best-in-class commercial platform
- Seq-Well S^3 was applied to profile inflammatory cell states in skin diseases
- Analysis of skin inflammation uncovered unique and conserved cellular phenotypes

Hughes et al., 2020, Immunity 53, 878–894
October 13, 2020 © 2020 The Authors. Published by Elsevier Inc.
https://doi.org/10.1016/j.immuni.2020.09.015

CellPress
OPEN ACCESS

Immunity

Resource

Second-Strand Synthesis-Based Massively Parallel scRNA-Seq Reveals Cellular States and Molecular Features of Human Inflammatory Skin Pathologies

Travis K. Hughes,[1,2,3,4,5,12,14] Marc H. Wadsworth II,[1,3,4,5,12,14] Todd M. Gierahn,[5,6,14] Tran Do,[7,13] David Weiss,[7,13] Priscila R. Andrade,[7,13] Feiyang Ma,[7,13] Bruno J. de Andrade Silva,[7,13] Shuai Shao,[8] Lam C. Tsoi,[8] Jose Ordovas-Montanes,[3,9,10,11] Johann E. Gudjonsson,[8] Robert L. Modlin,[9] J. Christopher Love,[3,4,5,6,15,*] and Alex K. Shalek[1,2,3,4,5,11,15,16,*]

[1]Institute for Medical Engineering & Science (IMES), MIT, Cambridge, Massachusetts, USA
[2]Department of Immunology, Harvard Medical School, Boston, MA, USA
[3]Broad Institute of MIT and Harvard, Cambridge, MA, USA
[4]Ragon Institute of MGH, MIT and Harvard, Cambridge, MA, USA
[5]Koch Institute for Integrative Cancer Research, MIT, Cambridge, MA, USA
[6]Department of Chemical Engineering, MIT, Cambridge, MA, USA
[7]Division of Dermatology, David Geffen School of Medicine at UCLA, Los Angeles, CA, USA
[8]Department of Dermatology, University of Michigan, Ann Arbor, MI, USA
[9]Division of Gastroenterology, Boston Children's Hospital, Boston, MA, USA
[10]Department of Pediatrics, Harvard Medical School, Boston, MA, USA
[11]Harvard Stem Cell Institute, Cambridge, MA, USA
[12]Department of Chemistry, MIT, Cambridge, Massachusetts, USA
[13]Department of Microbiology, Immunology and Molecular Biology, David Geffen School of Medicine at UCLA, Los Angeles, CA, USA
[14]These authors contributed equally
[15]Senior authors
[16]Lead Contact
*Correspondence: clove@mit.edu (J.C.L.), shalek@mit.edu (A.K.S.)
https://doi.org/10.1016/j.immuni.2020.09.015

SUMMARY

High-throughput single-cell RNA-sequencing (scRNA-seq) methodologies enable characterization of complex biological samples by increasing the number of cells that can be profiled contemporaneously. Nevertheless, these approaches recover less information per cell than low-throughput strategies. To accurately report the expression of key phenotypic features of cells, scRNA-seq platforms are needed that are both high fidelity and high throughput. To address this need, we created Seq-Well S^3 ("Second-Strand Synthesis"), a massively parallel scRNA-seq protocol that uses a randomly primed second-strand synthesis to recover complementary DNA (cDNA) molecules that were successfully reverse transcribed but to which a second oligonucleotide handle, necessary for subsequent whole transcriptome amplification, was not appended due to inefficient template switching. Seq-Well S^3 increased the efficiency of transcript capture and gene detection compared with that of previous iterations by up to 10- and 5-fold, respectively. We used Seq-Well S^3 to chart the transcriptional landscape of five human inflammatory skin diseases, thus providing a resource for the further study of human skin inflammation.

INTRODUCTION

Single-cell RNA-sequencing (scRNA-seq) is a powerful approach to define the cellular composition of healthy and diseased tissues (Klein et al., 2015; Macosko et al., 2015; Montoro et al., 2018; Ordovas-Montanes et al., 2018; Smillie et al., 2019; Vento-Tormo et al., 2018). The development of high-throughput methodologies has enabled the characterization of increasingly complex cellular samples. However, current scRNA-seq platforms typically demonstrate an inverse relationship between the number of cells that can be profiled at once and the amount of biological information that can be recovered from each cell. Thus, one must choose between quantity and quality, or alternatively employ two distinct approaches in parallel (Consortium et al., 2018). Indeed, inefficiencies in transcript capture among massively parallel methods limit our ability to resolve the distinct cell states that comprise broad cell types (Vieira Braga et al., 2019) and their essential but often lowly expressed molecular features, such as transcription factors, affinity receptors, and signaling molecules.

This is an open access article under the CC BY-NC-ND license (http://creativecommons.org/licenses/by-nc-nd/4.0/).

Improving the fidelity of scRNA-seq is particularly important for resolving differences within heterogeneous populations of immune cells (Dutertre et al., 2019; Villani et al., 2017). Here, subtle differences in surface receptor, transcription factor, and/or cytokine expression can profoundly affect cellular function, particularly in the setting of human pathology (Puel et al., 1998). Enhancing data quality in high-throughput scRNA-seq would facilitate a greater appreciation of the underlying molecular features that describe such cellular variation. Similarly, it would ease integration with legacy datasets that often utilize lowly expressed biomarkers, such as transcription factors and cytokines that are false-negative prone, when discriminating subsets of cells.

Most high-throughput scRNA-seq methods rely on early barcoding of cellular contents to achieve scale. Typically, these techniques recover single-cell transcriptomes for thousands of cells at once by leveraging reverse-emulsion droplets or picowells to isolate individual cells with uniquely barcoded poly-dT oligonucleotides, which can then capture and tag cellular messenger RNAs (mRNAs) during reverse transcription (Prakadan et al., 2017). Afterward, an additional oligonucleotide priming site is typically added to the 3′ end of the synthesized cDNA to enable PCR-based amplification of all transcripts (whole transcriptome amplification [WTA]). A number of techniques have been described to add this second priming site (Sasagawa et al., 2013; Shishkin et al., 2015). A common approach uses the terminal transferase activity of certain reverse transcriptase enzymes to facilitate a "template-switch" from the original mRNA to a second defined oligonucleotide (Picelli et al., 2013). Although simple to implement, this process has the potential to be highly inefficient, leading to the loss of molecules that have been converted to cDNA but not successfully tagged with a secondary PCR priming site (Islam et al., 2012; Kapteyn et al., 2010; Zajac et al., 2013).

To overcome these limitations, we have developed a massively parallel scRNA-seq protocol we call Seq-Well S^3 (for "Second-Strand Synthesis"), which incorporates the use of a randomly primed second-strand synthesis after reverse transcription to append a second oligonucleotide handle for WTA. In cell lines and peripheral blood mononuclear cells (PBMCs), we demonstrated that Seq-Well S^3 enables significant improvements in transcript and gene capture. To illustrate the utility of S^3, we applied it to examine the cellular composition of normal skin and uncover alterations in cellular abundance and phenotype across multiple inflammatory skin conditions, including acne, alopecia areata, granuloma annulare (GA), leprosy, and psoriasis. Overall, our work provides a key methodological advance and a valuable resource for understanding how diverse inflammatory responses can affect a single tissue, as well as the range of cellular phenotypes that are possible upon perturbation.

RESULTS

Second-Strand Synthesis (S^3) Leads to Improved Transcript Capture and Gene Detection

We hypothesized that the use of "template-switching" to append a second PCR handle during reverse transcription might limit the recovery of unique transcripts and genes from individual cells in some massively parallel scRNA-seq methods such as Seq-Well and Drop-Seq (Gierahn et al., 2017; Macosko et al., 2015). Thus, we incorporated a randomly primed second-strand synthesis following first-strand cDNA construction (Figures 1A and S1A). Briefly, after reverse transcription, we washed barcoded mRNA capture beads with 0.1 molar sodium hydroxide to remove attached RNA template strands, and then we performed a randomly primed second-strand synthesis to generate double-stranded cDNA that was labeled on one end with the SMART sequence and its reverse complement on the other (Figures 1A and S1A; STAR Methods) (Picelli et al., 2013; Picelli et al., 2014).

To examine the effectiveness of Seq-Well S^3, we tested a number of conditions by using cell lines and human PBMCs (Figure S1B; STAR Methods). Here, we observed that S^3 led to marked improvements in library complexity (i.e., the number of unique transcripts detected per aligned read) at matched sequencing depth below saturation (note: higher library complexity implies a greater amount of information remains to be detected through further sequencing). Seq-Well S^3 was further able to function in the absence of a template-switching oligo (TSO), whereas Seq-Well v1 failed to generate appreciable product without a TSO (Figures S1B–S1E). In species-mixing experiments using HEK293 (human) and NIH-3T3 (mouse) cell lines, we achieved significant increases in the numbers of unique transcripts and genes detected per cell by using Seq-Well S^3 compared with Seq-Well v1 ($p < 0.05$, Mann-Whitney U Test) (Figure S1D; STAR Methods), but comparable single-cell resolution (i.e., transcript purity) (Figures S1F and S1G).

To understand how Seq-Well S^3 would perform on primary cells, we applied it to human PBMCs (Figures S1C, S1E, S2, and S3; STAR Methods), benchmarking against Seq-Well v1 and multiple versions of a commercial technology (abbreviations for such are as follows: 10x Genomics, v2 3′ chemistry: 10x v2; 10 Genomics, v3 3′ chemistry: 10x v3). Here, we down-sampled all resulting data to equivalent numbers of aligned reads per cell to account for differences in sequencing depth (for comparisons between Seq-Well S^3 and 10x v2: 38,000 reads per cell; between Seq-Well S^3 v10x v3: 47,000 reads per cell) (Table S1; STAR Methods).

Critically, when we compared the complexity of sequencing libraries generated by using Seq-Well S^3 in relation to both Seq-Well v1 and 10x v2, we found that Seq-Well S^3 significantly increased the number of transcripts and genes detected at matched read depth ($p < 0.05$, Mann-Whitney U Test & Linear Regression) (Figures S1C, S1E, and S2A; STAR Methods). Both Seq-Well S^3 and 10x v2 displayed increased sensitivity compared with that of Seq-Well v1 (Seq-Well S^3: 6-fold gene detection, 10-fold unique molecular identifier [UMI] detection), but Seq-Well S^3 detected genes and transcripts for each cell type more efficiently than 10x v2 (defined as genes recovered at matched read depth) (Figure S2). Further, comparing Seq-Well S^3 to 10x v3 across PBMC cell types in aggregate (average read depth: 47,000 reads per cell), we observed that Seq-Well S^3 detected more genes per cell (Seq-Well S^3: 1,402 ± 739 genes per cell; 10x v3: 1,225 ± 496 genes per cell), whereas 10x v3 detected more transcripts per cell at comparable sequencing depth (Seq-Well S^3: 3,247 ± 2,418 UMIs per cell; 10x v3: 4,268 ± 2,109 UMIs per cell) (Figures 1B and 1C; Table S1).

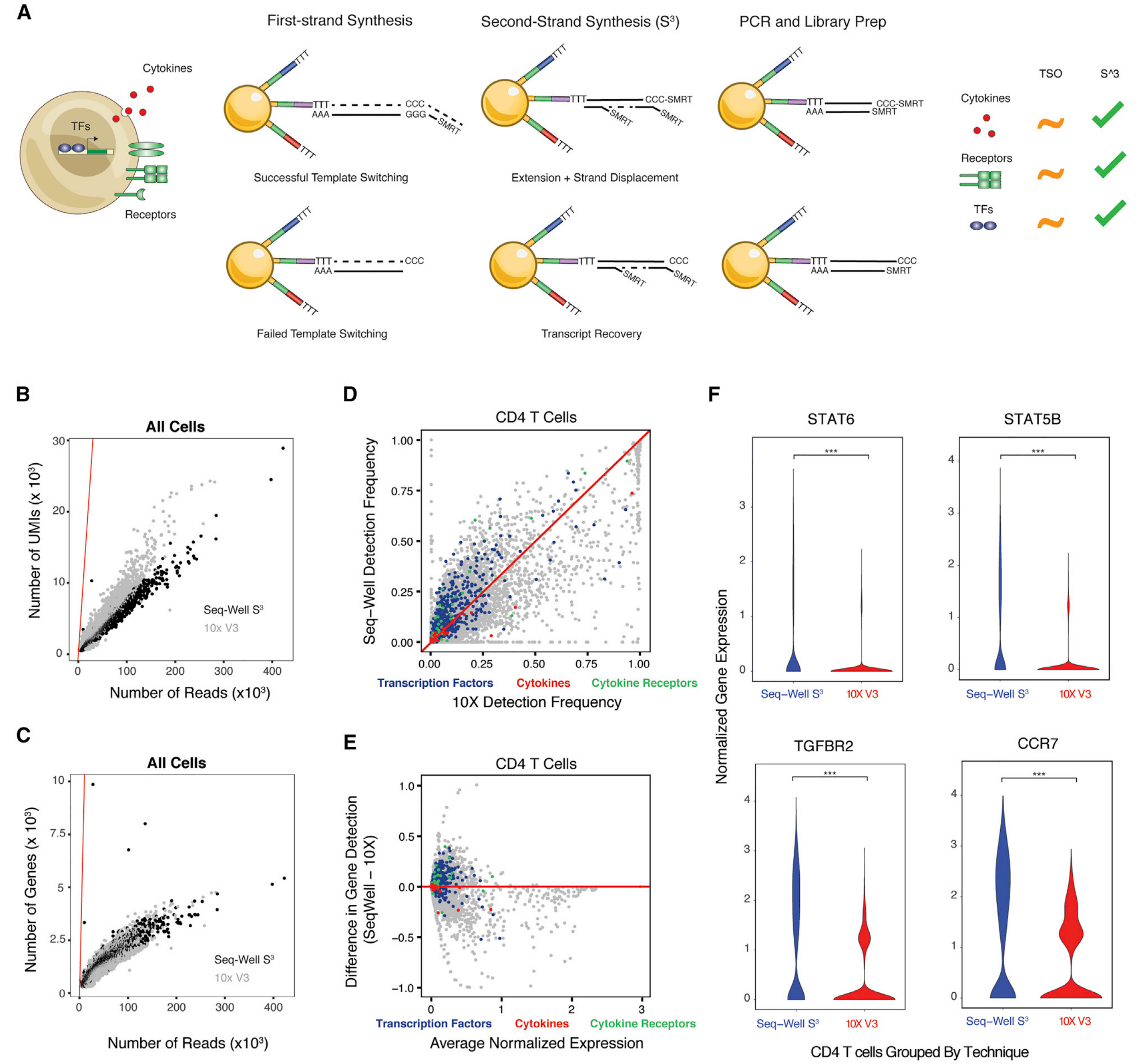

Figure 1. Overview of Second-Strand Synthesis (S^3)

(A) Conceptual illustration of the molecular features that define immune phenotypes as well as the Seq-Well second-strand synthesis method (Seq-Well S^3).

(B) Scatterplot showing differences in per-cell transcript capture (y-axis) as a function of aligned reads per cell (x axis) between 10x Genomics v3 (10x v3, grey) and Seq-Well S^3 (black) in human PBMCs. Red line indicates where transcripts per cell and aligned reads would be equivalent.

(C) Scatterplot shows the differences in per-cell gene detection (y axis) as a function of aligned reads per cell (x axis) between 10x v3 (grey) and Seq-Well S^3 (black) in human PBMCs. Red line indicates where genes per cell and aligned reads would be equivalent.

(D) Scatterplot comparing gene detection rates in $CD4^+$ T cells between 10x v3 (x axis) and Seq-Well S^3 (y axis). Red line indicates point of equivalence in gene detection frequency between methods. Colors correspond to classes of genes including transcription factors (blue), cytokines (red), and receptors (green). See also Table S1.

(E) Scatterplot comparing gene detection frequency (y axis) between Seq-Well S^3 (positive values) and 10x v3 (negative values) as a function of the average expression amounts (log(scaled UMI + 1)) of an individual gene (x axis). Red line indicates point of equivalence in gene detection frequency between methods. Colors correspond to classes of genes including transcription factors (blue), cytokines (red), and receptors (green). See also Table S1.

(F) Violin plots of the distribution of normalized expression values (log(scaled UMI + 1)) for select transcription factors and cytokine receptors between Seq-Well S^3 and 10x v3. ***$p < 1.0 \times 10^{-10}$.

We examined each cell type separately to confirm that these improvements were not driven by changes in the relative frequencies of different cell types (Figures S2B, S2C, S3A, and S3B). Among $CD4^+$ T cells, for example, we observed significant increases in the numbers of transcripts captured and genes detected by using Seq-Well S^3 in pairwise comparisons against 10x

v2 ($p < 0.05$, Mann-Whitney U Test; CD4$^+$ T cells, Seq-Well v1: 1,044 ± 62.3 UMIs per cell; 10x v2: 7,671 ± 103.9 UMIs per cell; Seq-Well S^3: 13,390 ± 253.4 UMIs per cell; mean ± standard error of the median [SEM]) (Figure S2D; Table S1; STAR Methods). Meanwhile, in comparison with 10x v3, we observed that Seq-Well S^3 detected more genes per cell (Seq-Well S^3: 1,226 ± 604 genes per cell; 10x v3: 1,083 ± 246 genes per cell), whereas 10x v3 detected more transcripts per cell at comparable sequencing depth (Seq-Well S^3: 2,739 ± 1,861 UMIs per cell; 10x v3: 4,047 ± 1,165 UMIs per cell) (Figure S3C; Table S1).

We sought to understand whether these improvements resulted in enhanced detection of biologically relevant genes typically under-represented in high-throughput scRNA-seq libraries (Consortium et al., 2018). Importantly, genes that were differentially detected (i.e., higher in S^3) within each cell type include numerous transcription factors, cytokines, and cell surface receptors (Figures 1D, 1E, S2E, S2F, S3D, and S3E; Table S1). For example, among CD4$^+$ T cells, compared with 10x v3, we observed significantly increased detection of transcription factors (e.g., *STAT6* and *STAT5B*) and cytokine receptors (e.g., *TGFBR2* and *CCR7*) (S^3 vs. 10x v3, $p < 0.05$, Chi-Square Test) (Figures 1F and S3; Table S1) in Seq-Well S^3.

We performed an additional comparison of enriched human CD4$^+$ T cells profiled by using Seq-Well S^3, 10x v2, and Smart-Seq2 (SS2), a commonly implemented microtiter plate-based scRNA-seq approach (Figures S2G–S2I; STAR Methods) (Picelli et al., 2013). Integrated analysis revealed that Seq-Well S^3 detected more genes per cell than 10x v2 and nearly as many as SS2 in pairwise comparison of the techniques (10x v2: 2,057 ± 18.7 genes per cell; Seq-Well S^3: 3,514 ± 36.2 genes per cell; SS2: 3,975 ± 74.0 genes per cell; mean ± SEM; $p < 0.05$, Mann-Whitney U Test) (Figure S2H; STAR Methods). Furthermore, comparing the frequency of gene detection between methods revealed crucial differences for transcription factors, cytokines, and cytokine receptors (STAR Methods). Surprisingly, we observed similar rates of gene detection between Seq-Well S^3 and SS2 for a large number of biologically informative genes (Figure S2G). Critically, although comparable numbers of genes were detected across methods, Seq-Well S^3 detected more genes per aligned read than either 10x v2 or SS2 ($p < 0.05$, Mann-Whitney U Test) (Figure S2I; STAR Methods).

Diverse Cellular States across Healthy and Inflamed Skin

To demonstrate the utility of Seq-Well S^3 to profile cellular states in human pathology, we applied it to characterize normal human skin and multiple inflammatory skin conditions, including acne, alopecia areata, GA, leprosy, and psoriasis. In total, we processed 19 skin biopsies (acne, n = 4; alopecia, n = 1; GA, n = 2; leprosy, n = 4; psoriasis, n = 5; normal skin, n = 3) and, after data quality filtering, retained 38,274 high-quality single-cell transcriptomes spanning 35 clusters identified through Louvain clustering in Scanpy (Wolf et al., 2018) (Figures 2A–2C and S4A–S4C; STAR Methods). To collapse clusters to cell types, we performed enrichment analyses to identify cluster-defining genes and manually assigned cell type identities on the basis of the expression of known lineage markers and hierarchical clustering (Figures 2B, S4A, S4C, and S4D; Table S3; STAR Methods). We further classified cells by using SingleR (Aran et al., 2019) and observed close concordance between manually identified cell types and automated classification where appropriate reference signatures existed (Figure S4B and Table S2; STAR Methods). Ultimately, we recovered 15 primary cell types, including the following: B cells (marked by expression of *MS4A1* and *CD79A*), fibroblasts (*DCN* and *COL6A2*), hair follicles (*SOX9*), keratinocytes (KCs) (*KRT5* and *KRT1*), Langerhans cells (LCs) (*CD207*), lymphatic endothelial cells (*LYVE1*), mast cells (*CPA3* and *IL1RL1*), melanocytes (*MLANA*), myeloid cells (*CD68* and *CTSS*), plasma cells (*IGHG1*), Schwann cells (*SCN7A*), sebocytes (*DCD*), T cells (*CD3D* and *TRBC2*), venular endothelial cells (ECs) (*SELE* and CD93), and vascular smooth muscle cells (VSMCs) (*TAGLN*) (Figures 2 and S4A–S4E; Table S3). As a final quality measure, we examined the distribution of reads, transcripts, and genes within each major cell population and observed consistent coverage (Figure S4F; Table S2).

Seq-Well S^3 Describes T Cell States across Inflammatory Skin Conditions

To determine the biological features that could be captured by using Seq-Well S^3, we first examined T cells because each inflammatory skin condition is known to significantly skew T cell phenotypes (Figure 3) (Diani et al., 2015; Lowes et al., 2014). We performed dimensionality reduction and sub-clustering across T cells alone (Figures 3A and 3B; STAR Methods). This revealed nine sub-clusters that closely correspond to natural killer (NK) cells and CD8$^+$ T cells, as well as several known CD4$^+$ T helper (Th) cell subsets. As before, we used the enhanced sensitivity of Seq-Well S^3 for lineage defining transcripts to help annotate the identity of each sub-cluster; for example, in regulatory T cells and Th-17 cells, we detected distinct expression of canonical transcription factors (e.g., *FOXP3* and *RORC*, respectively) and immune receptors (e.g. *TIGIT*, *CTLA4*, *IL2RA* and *CXCR6*, respectively) (Figures 3C–3D and S5; Table S3). Additionally, we cross-referenced each sub-cluster's marker genes against a series of curated signatures in the SaVant database (Lopez et al., 2017) to confirm our assignments, which highlighted similarity to previously characterized T cell and NK cell subsets (Best et al., 2013; Bezman et al., 2012) (Figure S5A; STAR Methods).

We next explored variability in T cell subset composition by skin pathology (Figures 3A and 3B). This revealed potentially varied T cell contributions to different classes of cutaneous inflammation. For example, in two out of four leprosy biopsies, we observed a population enriched for expression of canonical Th-17 genes including *RORC*, which encodes the Th-17-lineage-defining transcription factor RORγt (Ivanov et al., 2006) (Figures 3A and 3B). We further found that a sub-cluster of T cells, which express *NR4A1*, a transcription factor indicative of dysfunctional T cells (Liu et al., 2019), and are enriched for genes involved in nuclear organization (*NEAT* and *ANKRD36*), was over-represented in psoriasis samples (Figures 3B and 3C). We also observed an expansion of regulatory T cells in three of five patients with psoriasis, and an additional population of T cells characterized by expression of *SESN3,* a marker of T cell senescence (Lanna et al., 2017), *SATB1*, and *FURIN* (Figures 3A–3D).

Directed analysis within CD8$^+$ T cells revealed a sub-grouping of activated CD8$^+$ T cells expressing elevated amounts of several inflammatory cytokines (*TNF*, *CCL4*, and *XCL1*), specific affinity

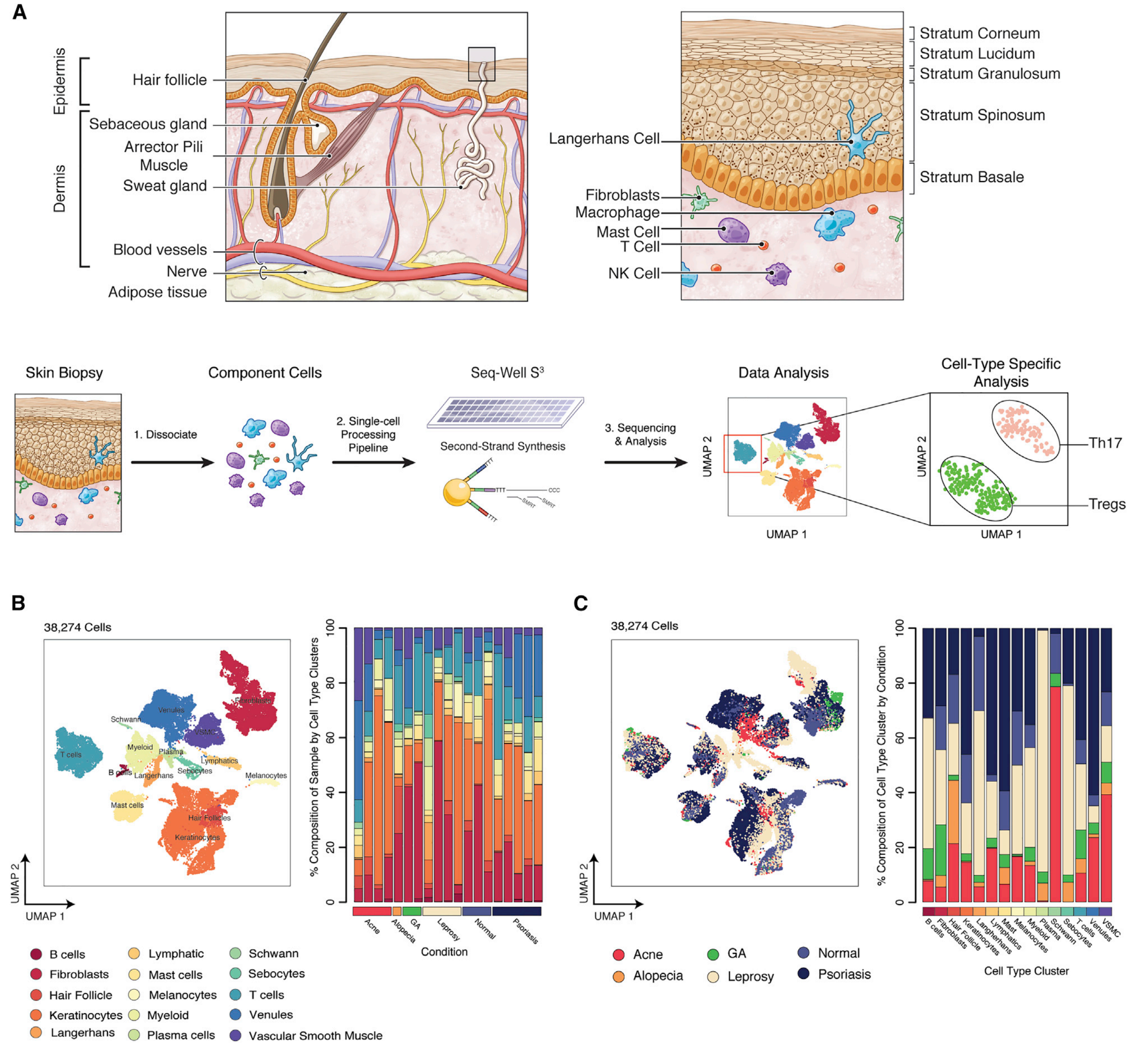

Figure 2. Cell Types Recovered across Inflammatory Skin Conditions

(A) (Top, left) Illustration of the anatomic organization and major features of human skin. Shown at the top, right is the cell type composition of the epidermis and dermis. Shown on the bottom is a sample processing pipeline for skin samples (Table S2).

(B) (Left) UMAP plot for 38,274 cells colored by cell type cluster. Shown on the right is a stacked barplot depicting the cell type composition for each of the 19 skin biopsies.

(C) (Left) UMAP plot for 38,274 cells colored by inflammatory skin condition. Shown on the right is a stacked barplot depicting the proportion of cells from each skin condition within phenotypic clusters.

receptors (*FASLG* and *TNFRSF9*), and transcription factors (*KLF9* and *EGR2*); this phenotypic skewing was observed primarily in a patient with GA (Figure S5B, top; Table S3; STAR Methods). We also uncovered considerable variation within the cluster containing cytotoxic T cells and NK cells (cytotoxic), where we found the highest degree of cytotoxic gene expression (*GNLY*, *GZMB*, and *PRF1*) (Table S3). Indeed, sub-clustering analysis of this cytotoxic cluster revealed 3 distinct sub-groups (Figure S5B): (1) a sub-group of NK cells (cytotoxic-1) enriched for expression of *c-KIT*, *RANKL* (TNFSF11), and *GITR* (TNFSFR18); (2) a sub-group of *CD16*$^+$ cells (cytotoxic-2) expressing cytotoxic effector molecules (*GNLY*, *PRF1*, and *GZMB*) and NK surface receptors, consistent with either NK cell or tri-cytotoxic cytotoxic T lymphocytes (CTLs) (Balin et al., 2018); and (3) a sub-group of CD8$^+$ T cells (cytotoxic-3; marked by *TNFSF8*, *SLAMF1*, *CLEC2D*, and *CD5*) that express both TCR $\alpha\beta$ and $\gamma\delta$ constant genes (Figure S5B, bottom; Table S3) (Söderström et al., 2010).

CellPress
OPEN ACCESS

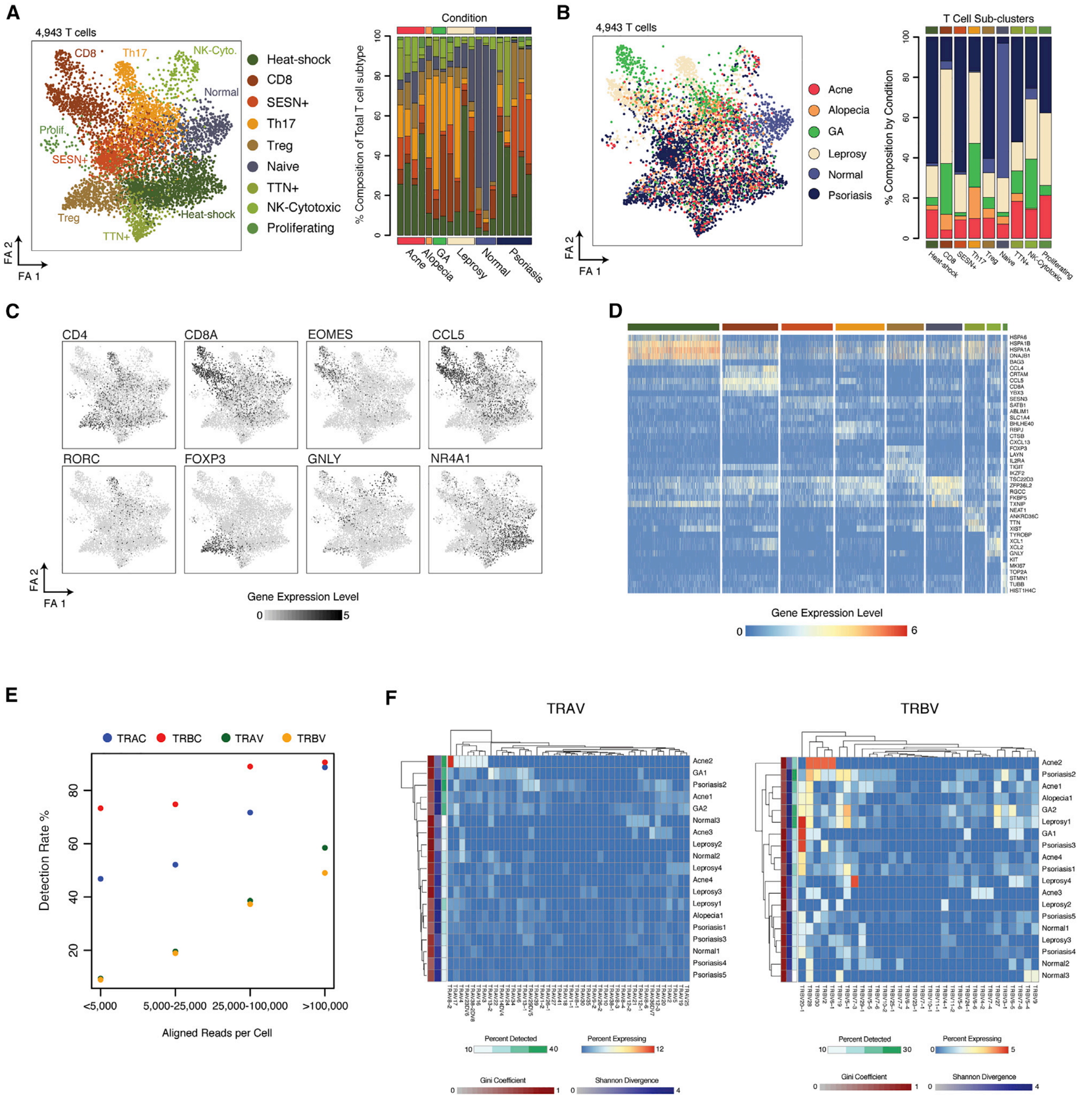

Figure 3. Identification of Inflammatory T cell States by using Seq-Well S[3]

(A) (Left) Force-directed graph of 4,943 T cells colored by phenotypic sub-cluster. Shown on the right is a stacked barplot depicting the distribution of T cell sub-clusters within each biopsy.

(B) (Left) Force-directed graph of 4,943 T cells colored by inflammatory skin condition. Shown on the right is a stacked barplot depicting the contribution of each inflammatory skin condition to the T cell sub-clusters.

(C) T cell force-directed graphs displaying normalized expression (log(scaled UMI + 1)) of a curated group of sub-cluster-defining gene. Higher expression values are shown in black.

(D) Heatmap showing normalized gene expression values (log(scaled UMI + 1)) for a curated list of sub-cluster-defining genes across nine T cell sub-clusters. See also Table S3.

(E) Plot showing rates of detection of TCR genes from human skin T cells across a range of sequencing depths.

(F) Heatmaps showing the distribution of TRAV (left) and TRBV (right) gene expression among T cells within each sample. Within each sample (rows), the color represents the percent of T cells expressing a given TRAV or TRBV gene (columns). The sidebar shows the gini coefficient (red), the Shannon Divergence (blue), and the percent of T cells (green) within each sample with non-zero expression of either TRAV or TRBV genes.

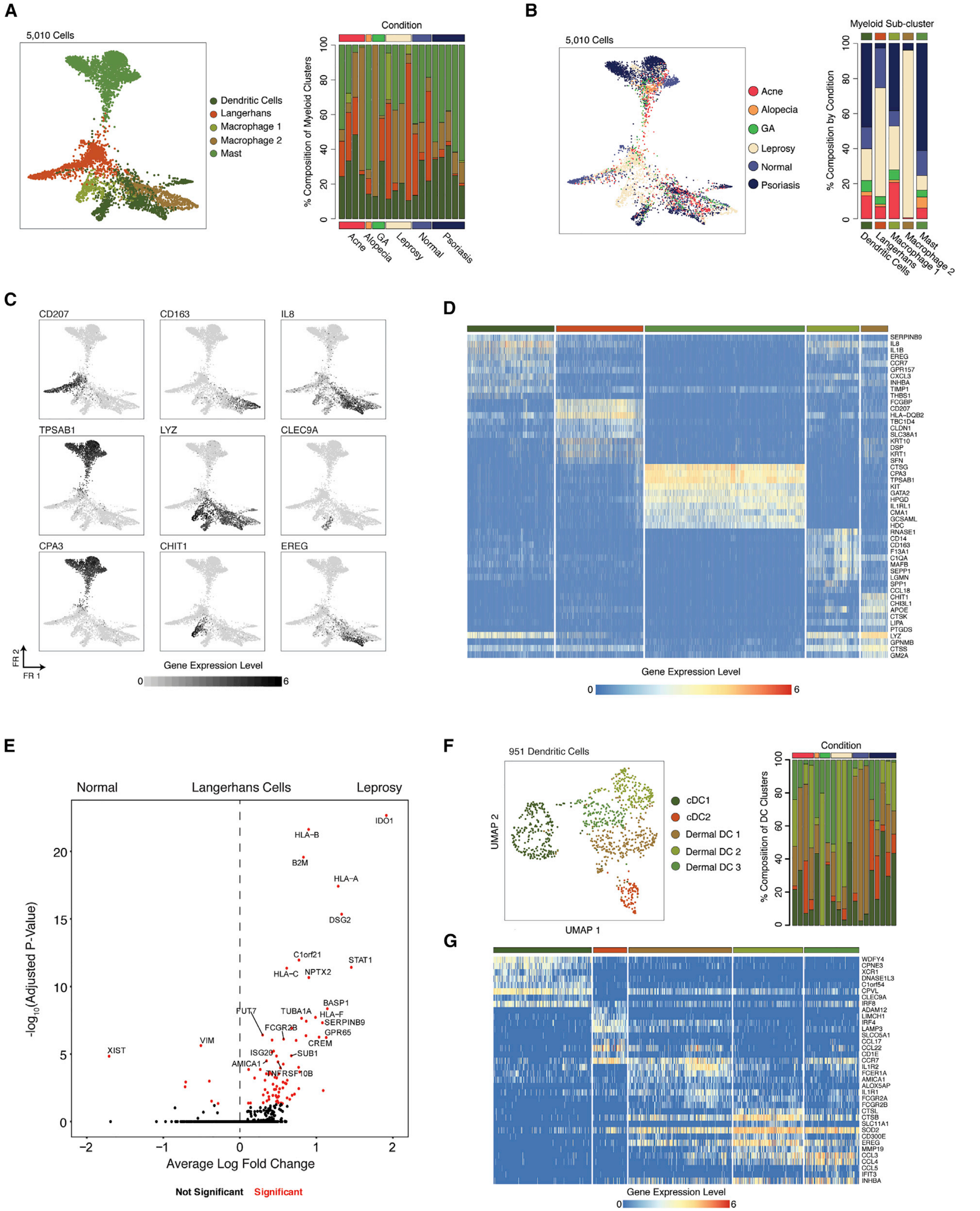

Figure 4. Diverse Myeloid Cell States Uncovered by using Seq-Well S^3

(A) (Left) Force-directed graph of 5,010 myeloid cells colored by phenotypic sub-cluster (NB, LCs were enriched from leprosy and normal skin). Shown on the right is a stacked barplot showing the distribution of myeloid sub-clusters within each biopsy. See also Table S3.

(legend continued on next page)

CellPress
OPEN ACCESS

Profiling of TCR expression is critical to understand T cell antigen specificity (Zhang et al., 2018). Among $CD4^+$ T cells obtained from peripheral blood, we recovered TCR-V and TCR-J genes at a higher frequency by using Seq-Well S^3 than by using 10x v2 ($p < 0.05$, Chi-square Test) (Figure S5C; STAR Methods), and observed paired detection of *TRAC* and *TRBC* in Seq-Well S^3 in 1,293 out of 1,485 $CD4^+$ T cells (87.1% Paired Detection Rate) (Figure S5C). In the setting of skin inflammation, we detected *TRAC* in 53.5% of T cells, *TRBC* in 76.7% (Figure 3E), and paired detection in 45.1%. Among T cells with at least 25,000 aligned reads, we recovered paired α and β chains in 68.6%. Among cytotoxic cells, we observed expression of γ and δ constant genes (*TRGC* and *TRDC*), whereas the remaining T cell clusters exclusively expressed α and β TCR constant genes (Table S3). These data further suggested that the cytotoxic cluster represents a diverse population of $\gamma\delta$, NK, and cytotoxic $CD8^+$ T cells that share common gene expression features and, potentially, roles in inflammation.

Finally, we examined the distribution of TCR V gene expression across inflammatory skin biopsies to identify clonally expanded T cells (Figure 3F; STAR Methods). We found biased distributions of TRAV and TRBV genes (e.g., elevated Gini coefficients and low Shannon Divergence) (Oakes et al., 2017) within multiple biopsies including those from leprosy and acne (Leprosy 2 and Acne 2, TRAV and TRBV Gini Coefficient > 0.85) (Figure 3F).

Spectrum of Myeloid Cell States in Skin Inflammation

In the setting of cutaneous inflammation, myeloid cells play a key role in maintaining tissue homeostasis, wound healing, and response to pathogens (Malissen et al., 2014). We identified numerous myeloid cell subpopulations defined by combinations of surface markers, cytokines, and lineage-defining transcription factors. Specifically, we independently analyzed 5,010 myeloid cells and uncovered 10 sub-clusters representing 4 primary myeloid cell types based on expression of canonical lineage markers and comparison to cell type signatures in the SaVant database: dendritic cells (DCs) (*CLEC9A* and *CLEC10A*), LCs (*CD207* and *CD1A*), macrophages (*CD68* and *CD163*), and mast cells (*CPA3* and *TPSAB1*) (Figures 4A, S5D, and S5E; Table S3; STAR Methods) (Lopez et al., 2017).

Among the macrophages, our data revealed two distinct subclusters (Figures 4A and 4B). One spanned normal skin as well as multiple types of skin inflammation and was characterized by elevated expression of previously characterized markers of dermal macrophages (*CD163*, *STAB1*, and *CEPP*) (Fuentes-Duculan et al., 2010). The other, meanwhile, was observed primarily in a single leprosy patient and was defined by genes involved in extracellular proteolysis (*LYZ*, *CHIT1*, and *CHI3L1*) (Di Rosa et al., 2013).

We initially identified LCs cells on the basis of expression of canonical markers (*CD207* and *CD1A*) (Figures 4C and 4D; Table S3) (Romani et al., 2003). When we directly compared LCs between a single leprosy biopsy and a single normal skin biopsy from which we performed bead-based LC enrichment (STAR Methods), we detected elevated expression of *IDO1*, *STAT1*, *HCAR3*, and MHC class I molecules (*HLA-A*, *HLA-B*, and *HLA-F*) in LCs in leprosy infection (Figure 4E; Table S5) (Hunger et al., 2004; Pinheiro et al., 2018). We further performed gene-ontology analysis among genes up-regulated in LCs from leprosy and observed enrichment of genes related to IFN-γ response (Table S5).

Sub-analysis of the DC cluster revealed multiple sub-groups, including conventional and dermal DCs (Figure 4F). Consistent with previous observations from peripheral blood, we detected a sub-group that corresponds to cDC1 (*CLEC9A*, *IRF8*, and *WDFY4*) (Villani et al., 2017) (Dutertre et al., 2019) ($p < 0.05$, permutation test) (Figure S5F and S5G; STAR Methods). We further identified another representing cDC2 cells (*IRF4*, *SOCS2*, *SLCO5A1*, *CD1B*, and *CD1E*) (Figures 4F, S5H and Table S3; STAR Methods) (Guilliams et al., 2016). Importantly, we detected expression of *IL12B*, a subunit of the IL-23 cytokine, which has previously been shown to promote mucosal type 17 inflammation via secretion of IL-23 (Schlitzer et al., 2013), within these $IRF4^+$ cDC2 cells (Figure S5I). This sub-grouping of cDC2 cells also expressed high amounts of *CCL17* and *CCL22*, chemokines involved in T cell chemotaxis (Figure 4G) (Stutte et al., 2010).

Among the dermal DCs, we identified 3 subgroups that were broadly distinguished from the conventional DC clusters by expression of *CLEC10A* (Figure S5I), which has been shown to influence T cell cytokine responses in skin (Kashem et al., 2015; Kumamoto et al., 2013). Cells from dermal DC sub-group 1 showed elevated expression of *IL1R1*, *IL1R2*, and *CCR7* and Fc-receptors including *FCER1A*, *FCGR2A*, and *FCGR2B*, which are important for interfacing with humoral immunity (Figure S5I; Table S3) (Guilliams et al., 2014). We observed a second population of dermal DCs (dermal DC sub-group 2), and there was elevated expression of cathepsins (*CTSL* and *CTSB*) and surface receptors (*CD300E* and *SLC11A1*), which collectively represent markers of DC activation (Figure S5I) (Brckalo et al., 2010). Finally, a third sub-grouping of dermal DCs (dermal DC sub-group 3) was distinguished elevated expression of pro-inflammatory chemokines up-regulated during DC maturation (*CCL3*, *CCL4*, and *CCL5*) (Jin et al., 2010) and soluble mediators (*EREG* and *INHBA*).

Detection of Endothelial Heterogeneity and Vascular Addressin Expression

Multiple types of ECs exist within the dermis of the skin. Importantly, $DARC^+$ post-capillary venules are the primary site of

(B) (Left) Force-directed graph of 5,010 myeloid cells colored by inflammatory skin condition. Shown on the right is a stacked barplot showing the contribution of each inflammatory skin condition to each myeloid sub-cluster.

(C) Force-directed graphs of 5,010 myeloid cells highlighting expression of a curated group of sub-cluster defining genes (log(scaled UMI + 1)). Higher expression values are shown in black. See also Table S3.

(D) Heatmap showing the normalized expression (log(scaled UMI + 1)) of a curated list of myeloid cell type cluster-defining genes.

(E) Volcano plot showing genes differentially expressed in LCs between leprosy ($n_{cells} = 67$) and normal skin ($n_{cells} = 171$). Log10-fold change values are shown on the x axis and −log10 adjusted p values are shown on the y axis. See also Table S5.

(F) (Left) UMAP plot for 951 DCs from human skin colored by inflammatory skin condition. Shown on the right is a stacked barplot showing the distribution DC sub-grouping within 19 skin biopsies.

(G) Heatmap showing the distribution of normalized gene expression amounts (log(scaled UMI + 1)) for cluster-defining genes across dermal DC subpopulations. See also Table S3.

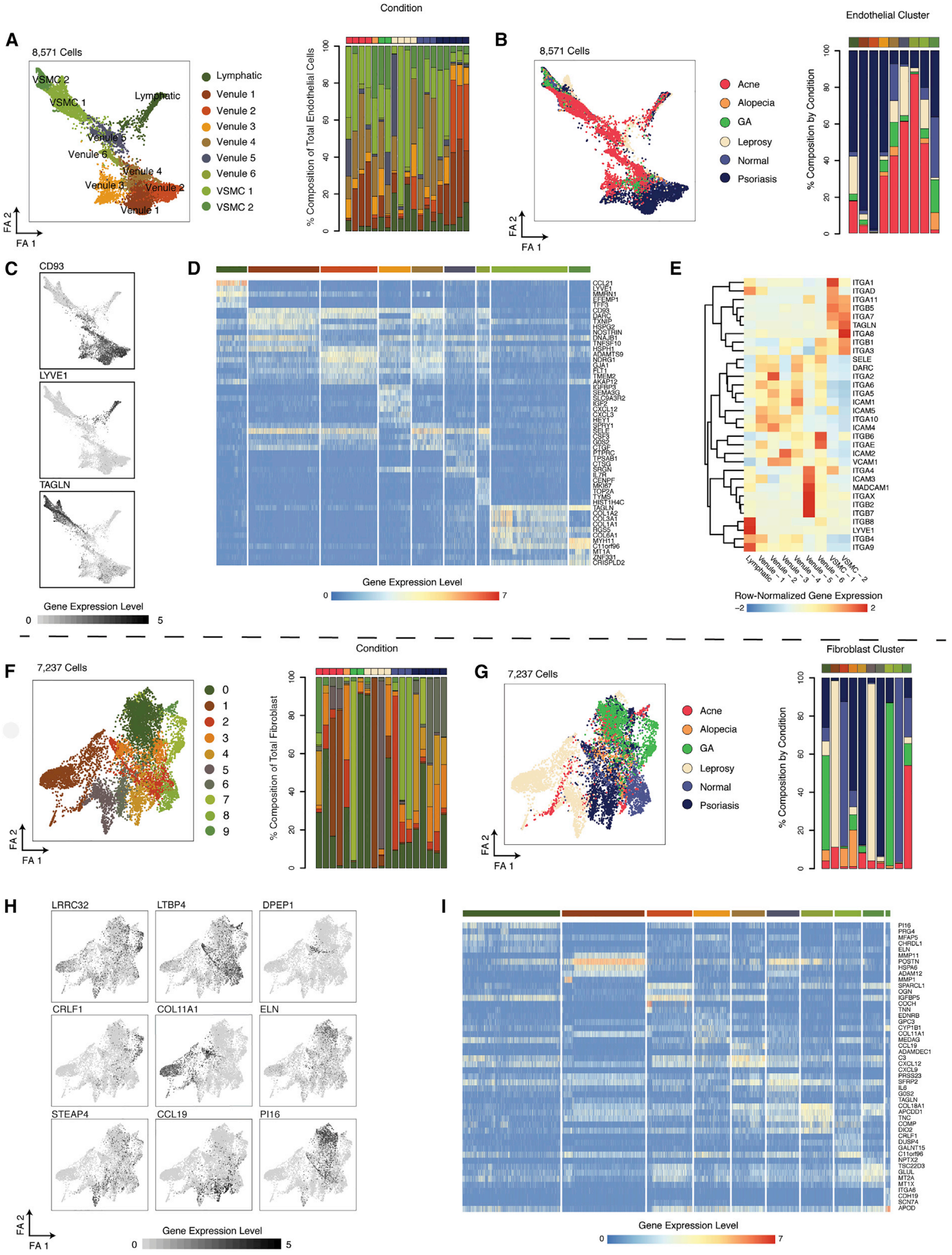

Figure 5. Stromal Cell Diversity

(A) Force-directed plots for 8,571 endothelial cells colored by phenotypic sub-cluster (left) and stacked barplot showing the distribution of endothelial phenotypic sub-clusters across samples (right) (Table S3).

(legend continued on next page)

egress of immune cells from circulation into tissues, which is guided by addressin expression (Schön et al., 2003). Using the improved sensitivity of Seq-Well S^3, we sought to understand the spectrum of EC diversity and vascular addressin expression across multiple instances of skin inflammation (von Andrian and Mempel, 2003). We identified three primary sub-clusters of dermal ECs defined by distinct expression patterns: VSMCs (*TAGLN*), ECs (*CD93*), and lymphatic ECs (*LYVE1*) (Figures 5A–5C; STAR Methods). Importantly, we found multiple sub-clusters of $CD93^+$ ECs across normal and inflamed skin biopsies (Figures 5A–5B; Table S3). For example, we observed a cluster of *DARC*$^-$, *CD93*$^+$ ECs (venule sub-cluster 3) that displays elevated expression of *SLC9A3R2*, which is involved in endothelial homeostasis (Bhattacharya et al., 2012), and another that is proliferating (venule sub-cluster 6) (Figure 5D). Notably, across sub-populations of $CD93^+$ ECs (venule sub-clusters 1–6), we observed varied expression of vascular addressins (Thiriot et al., 2017) (Figure 5E). Among post-capillary venules, we measured broadly elevated expression of *ITGA5*, *ITGA6*, *ICAM2*, and *ITGA2*, whereas VSMCs expressed higher amounts of *ITGA7*, *ITGA8*, and *ITGB5*. Further, we observed the highest expression of *ITGB4*, *ITGB8*, and *ITGA9*, among lymphatic ECs (Figure 5E).

Altered Dermal Fibroblast Identities in Skin Inflammation

Dermal fibroblasts provide structural support and are the primary source of extracellular matrix components within the skin. Previous studies have reported significant variation among dermal fibroblasts on the basis of their relationship to anatomic features of the skin (Driskell et al., 2013; Driskell and Watt, 2015). In comparison to inflamed biopsies, fibroblasts from normal skin display enrichments in *LTBP4*, *IGFBP5*, and *TCF4* (fibroblast clusters 2 and 8) (Table S3). Consistent with previous single-cell studies of dermal fibroblasts, we observed a sub-population of fibroblasts (fibroblast cluster 3) that expressed *COL11A1*, *DPEP1*, and *RBP4* and is suggested to have a role in connective tissue differentiation (Figure 5H; Table S3) (Tabib et al., 2018).

In GA, we observed two distinct fibroblast populations. Fibroblasts from GA patient 1 (sub-cluster 0) displayed elevated expression of protease inhibitor 16 (*PI16*), which inhibits the function of MMP2 (Hazell et al., 2016), and *ITIH5*, a protease inhibitor important for maintenance of dermal hyaluronic acid that is overexpressed in skin inflammation (Figures 5H–5I; Table S3) (Huth et al., 2015). Fibroblasts from GA patient 2 (sub-cluster 7), meanwhile, expressed elevated amounts of *SPOCK1* (Avg-Log FC: 0.99), *CRLF1* (Avg-Log FC: 1.38), and *COMP* (Avg-Log FC: 1.35), a cartilage protein that is upregulated in matrix-producing fibroblasts after myocardial infarction (Fu et al., 2018).

We also observed distinct fibroblast phenotypes in leprosy infection. Specifically, we found a population of fibroblasts (fibroblast cluster 1) marked by combined expression of *POSTN* (Periostin) and *MMP11*, a marker of fibroblasts in basal cell carcinoma (Micke et al., 2007) (Figure 5I; Table S3). In another leprosy biopsy, we observed a population of pro-inflammatory fibroblasts (fibroblast cluster 5) that expresses elevated amounts of *SFRP2*, *PRSS23*, and *IL6*. Finally, among all 5 psoriasis biopsies, we observed a population of pro-inflammatory fibroblasts (fibroblast cluster 4) marked by elevated expression of *CCL19*, *TNFSF13B* (BAFF), and *CXCL12* (Figures 5H–5I; Table S3).

Keratinocyte Differentiation Trajectories

Within the epidermis, KCs undergo a stereotyped differentiation process in which cells acquire altered morphologies and phenotypes as they mature (Figure 6A) (Fuchs, 1990). Using KCs from normal skin, we performed pseudo-temporal analysis to reconstruct the differentiation process of normal epidermal KCs (Figure 6B; STAR Methods) (Saelens et al., 2019). In normal skin, we first identified a population of KCs enriched for expression of *KRT14*, a marker of basal KCs (Figure 5C) (Pellegrini et al., 2001). We then used known patterns of cytokeratin expression to infer localization of KCs along a supervised differentiation trajectory (Figures 6C and S6A) (Ordovas-Montanes et al., 2018). Our trajectory analysis revealed patterns of transcription factor and cytokeratin expression that closely correspond to previously established signatures of KC maturation in both normal skin samples where we recovered enough KCs to perform trajectory analysis (STAR Methods; Figures S6A and S6B) (Cheng et al., 2018). Consistent with immunohistochemical staining from the Human Protein Atlas (Figure 6C) (Uhlén et al., 2015), we found enriched expression of filaggrin (*FLG*), a protein in the outer layers of the epidermis (Sandilands et al., 2009), among keratinocytes that lie at the terminal points in the pseudo-temporal ordering (Figures 6C and S6B).

We next examined patterns of KC differentiation across pathologic conditions and discovered marked deviation in the differentiation trajectory of psoriatic KCs (Figure 6D). We further identified distinct lineages for basal KCs (*COL17A1*) and cells of the hair follicle, where we detected enrichment of published hair follicle signatures (Figure 6E) (Joost et al., 2016). Consistent with previous reports, differential expression analysis revealed significant up-regulation of antimicrobial peptides (*S100A7*, *S100A8*, and *S100A9*) and pro-inflammatory cytokines (*IL36G* and

(B) Force-directed plots for 8,571 endothelial colored by inflammatory skin condition (left) and stacked barplot showing the contribution of each inflammatory skin condition to endothelial phenotypic sub-clusters (right).
(C) Force-directed plot colored by normalized expression amounts of genes that mark endothelial cell types: (Left) *CD93*, venules, (Middle) *TAGLN*, arterioles, (Right) *LYVE1*, lymphatics.
(D) Heatmap showing patterns of normalized gene expression amounts (log(scaled UMI + 1)) across nine clusters of endothelial cells (Table S3).
(E) Heatmap showing row-normalized expression amounts of vascular addressins across phenotypic sub-clusters of endothelial cells.
(F) Force-directed plots for 7,237 fibroblasts colored by phenotypic sub-cluster (left) and stacked barplot showing the distribution of fibroblast phenotypic sub-clusters across samples (right) (Table S3).
(G) Force-directed plots for 7,237 fibroblasts colored by inflammatory skin condition (left) and stacked barplot showing the contribution of each inflammatory skin condition to fibroblast phenotypic sub-clusters (right).
(H) Force-directed graphs highlighting fibroblast cluster-defining genes.
(I) Heatmap showing the normalized gene expression levels (log(scaled UMI + 1)) of fibroblast cluster-defining genes (Table S3).

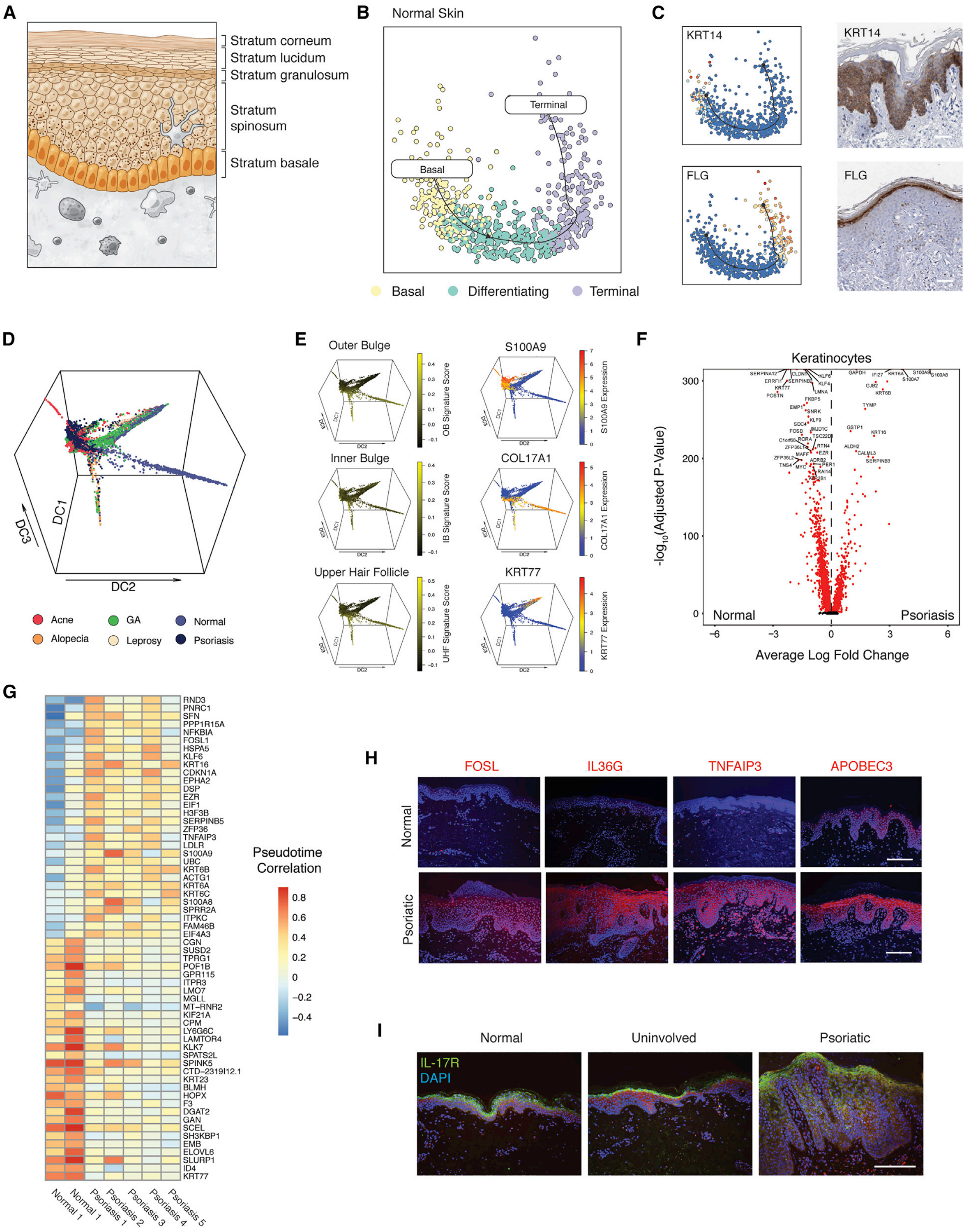

(legend on next page)

IL36RN) in psoriatic KCs (Figures 6E–6F; Table S6) (Li et al., 2014).

Based on increased sensitivity of Seq-Well S^3 to detect transcription factors observed in peripheral lymphocytes, we hypothesized that our data might enable identification of transcriptional regulators of psoriatic KCs. To identify potential drivers of the psoriatic disease process within the epidermis, we performed differential pseudo-time correlation analysis between psoriatic and normal KCs (STAR Methods). We separately constructed pseudo-time trajectories for normal (n = 2) and psoriatic KCs (n = 5), calculated correlation values between diffusion pseudo-time and gene expression amounts, and examined the difference in correlation values between psoriatic and normal KCs (Figures 6G, S6A, and S6B; Table S6). This uncovered positive correlation of *FOSL1*, an AP-1 transcription factor, with diffusion pseudo-time in psoriatic KCs, implying that *FOSL1* might be aberrantly expressed along the differentiation trajectory of psoriatic KCs. To validate this observation, we performed immunofluorescence staining for FOSL1 protein, and measured increased amounts of FOSL1 in psoriatic skin (Figure 6H; STAR Methods). We validated the distribution of additional genes overexpressed or differentially correlated with diffusion pseudo-time in psoriatic KCs (including *TNFAIP3*, *IL36G*, and *APOBEC3*) at the protein level (Figures 6H and S6A; Table S6; STAR Methods). Finally, we examined the relationship between differential expression and difference in pseudo-time correlation (Figure S6C). Here, we observed no overall relationship between differential expression and differential pseudotime, suggesting a more complicated picture of dysregulated gene expression in psoriatic KCs.

To further define differences in gene expression between normal and psoriatic KCs, we scored the expression amounts of known cytokine response signatures by using a series of reference signature gene lists derived from population RNA-seq of cultured keratinocytes exposed to multiple cytokines including IL-17A, IL-4, IL-13, TNF-α, IFN-α, and IFN-γ (Figure S6D; Table S6; STAR Methods) (Tsoi et al., 2019). Although IL-17 has been previously implicated in the pathogenesis of psoriasis, here we inferred the identity of cells that dominate the IL-17 response, localizing the expression of IL-17 responsive genes to spinous KCs (Chiricozzi et al., 2014). To validate this observation, we performed immunofluorescent staining for IL-17R protein and measured the highest staining within spinous KCs exclusively within psoriatic skin (Figure 6I; STAR Methods).

DISCUSSION

Here, we present Seq-Well S^3, a high-throughput and high-fidelity scRNA-seq platform. Through use of a templated second-strand synthesis, S^3 reclaims cDNA molecules that were successfully reverse transcribed but not labeled with a second oligonucleotide handle through template switching and thus would normally have been lost in common bead-based high-throughput scRNA-seq protocol such as Seq-Well or Drop-Seq. Using Seq-Well S^3, in relation to Seq-Well v1 (Gierahn et al., 2017), we obtained a 5- to 10-fold increase in the number of unique genes and transcripts captured per cell at similar sequencing depth. Beyond aggregate increases in the number of genes and transcripts recovered per cell, Seq-Well S^3 facilitated enhanced detection of lineage-defining factors in immune and parenchymal cells—such as transcription factors, cytokines, and cytokine receptors that are often transiently or lowly expressed among lymphocytes. Critically, the Seq-Well S^3 protocol is easy to integrate into current bead-based RNA-seq platforms, such as Drop-Seq (Macosko et al., 2015) and spatial RNA-seq platforms like Slide-Seq (Rodriques et al., 2019), making it broadly useful for the single-cell community.

Increases in the sensitivity of gene and transcript detection are increasingly important as single-cell atlasing efforts shift from defining large differences between cell types within normal tissues to characterizing subtle alterations to cell states in disease. Although a number of high-throughput methods have been developed, each fills a specific role. For example, the methods that rely on split-pool barcoding of cells or nuclei, such as sci-RNA-seq (Cao et al., 2017) or SPLiT-seq (Rosenberg et al., 2018), can examine tens to hundreds of thousands of cells at once, enabling characterization of model organisms or complex chemical screens, but work best with certain cell types and are associated with substantial cell and transcript loss (Ding et al., 2020), limiting their applicability to precious clinical samples. Commercial reverse-emulsion droplet-based methods, like 10x, overcome these inefficiencies and provide streamlined workflows, but add substantial cost, both with respect to consumables and instrumentation, and constrain where and how

Figure 6. Keratinocyte Differentiation Trajectories

(A) Diagram showing the layers of the epidermis and morphologic changes associated with keratinocyte differentiation.

(B) t-SNE plot showing differentiation trajectory of keratinocytes from normal skin from basal cells (yellow) through differentiating cells (aqua) and terminal keratinocytes (purple).

(C) (Top, left) t-SNE plot of normal keratinocytes colored by *KRT14* expression. Shown at the top right is KRT14 staining from the human protein atlas (Uhlén et al., 2015). Shown on the bottom left is a t-SNE plot of normal keratinocytes colored by *FLG* expression. Shown on the bottom right is FLG staining from the human protein atlas (Uhlén et al., 2015). Scale bars, 50 μm.

(D) Diffusion map of 10,777 keratinocytes colored by inflammatory skin condition. Axes correspond to diffusion components 1, 2, and 3.

(E) Diffusion map of keratinocytes colored by signatures of hair-follicle-specific gene expression (Joost et al., 2016) (Left: outer bulge, inner bulge, and upper hair follicle) and genes that distinguish basal (*COL17A1*), normal (*KRT77*), and inflamed (*S100A9*) keratinocytes.

(F) Volcano plot of genes differentially expressed between psoriatic and normal keratinocytes. Log10-fold change values are shown on the x axis and −log10 adjusted p values are shown on the y axis.

(G) Heatmap showing gene-specific Pearson correlation values between diffusion pseudotime and gene expression for two normal skin biopsies and five psoriatic biopsies.

(H) (Top) Immunofluorescence staining in normal (above) and psoriatic (below) for FOSL, IL36G, TNFAIP3, and APOBEC3. All images stained for nuclei (DAPI) and gene of interest (red fluorescence). Scale bar, 100 μm.

(I). Immunofluorescence staining for IL-17R expression (green) in normal (left), uninvolved (middle), and psoriatic skin (right). Scale bar, 100 μm.

samples can be run. Although the state-of-the-art continues to evolve rapidly, Seq-Well S^3 provides a competitive alternative that is uniquely suited for clinical studies because of its efficiency, simplicity, compatibility with fragile cells, limited peripherals, flexible stopping points (post-reverse transcription), ability to be parallelized (up to 20 samples in a one-day experiment), high degree of technical reproducibility, and open molecular biology (which enables targeted enrichment of molecules of interest) (Tu et al., 2019; van Galen et al., 2019).

Cost is a key factor in the selection of scRNA-seq methods. Prior to sequencing, the cost of Seq-Well S^3 is significantly less than that of commercial scRNA-seq platforms. Ignoring differences in instrumentation requirements and their associated costs (over an order of magnitude less for Seq-Well S^3), Seq-Well S^3 and 10x v3 required approximately the same amount of money to obtain 50,000 aligned reads for the PBMCs presented here (note: alignment rates and exact figures will change as a function sample type and pre-processing). Although this price exceeds that for the cell-based split-pool methods, at present, the fidelity is higher. Moreover, the throughput and price of processing for both can be further reduced through sample multiplexing, driving sequencing, rather than sample preparation (especially for Seq-Well S^3), to dominate costs (McGinnis et al., 2019; Stoeckius et al., 2018).

The increased sensitivity of gene detection and transcript capture afforded by Seq-Well S^3 enhances the strength of the inferences and hypotheses that can be generated when examining the cellular and molecular features of disease pathophysiology by using scRNA-seq. To date, single-cell analyses of healthy and diseased human skin have revealed heterogeneity among immune and parenchymal cell types (Cheng et al., 2018; He et al., 2020; Kim et al., 2020; Tabib et al., 2018). However, these studies have largely focused on a single cell type or disease. Here, we examined the cellular composition of normal skin as well as alterations in cellular phenotypes associated with multiple inflammatory skin conditions, including acne, alopecia areata, GA, leprosy, and psoriasis. Our results provide a draft atlas of human skin inflammation, creating a compendium of cell types and states for the broader research community (Angelo et al., 2014) while providing insights into putative mechanisms and the cellular localization of previously appreciated and unknown responses to specific inflammatory mediators in immunologic skin conditions.

We detected numerous T cell phenotypes and sub-phenotypes across inflammatory skin conditions by using Seq-Well S^3. Among multiple psoriasis biopsies, we observed over-representation of Tregs, dysfunctional *NR4A1*-expressing T cells, and senescent *SESN3*$^+$ T cells, which could reflect a role for broader T cell dysfunction in disease pathology (Šahmatova et al., 2017). Meanwhile, in leprosy, we identified a population of T cells enriched for expression of *ROR-*γ*T*, consistent with a suggested role for Th-17 cells in bacterial control (Saini et al., 2013; Saini et al., 2016). However, this population was only detected in two of four biopsies, including one patient undergoing a reversal reaction, which implies a variable role for Th-17 cells across patients and forms of leprosy. Further, using Seq-Well S^3, we observed improved TCR recovery in PBMCs. By examining V region sharing, we found the highest enrichment of TCR sequences in leprosy and acne biopsies, which suggests an important role for antigen-specific T cell responses in these diseases consistent with recent data from acne (Shao et al., 2020). However, further experimentation is needed to more fully understand the relationship between TCR clonality and T cell phenotype in skin inflammation.

In psoriasis, T cells are thought to be a primary driver of inflammation, and DCs play a central role in the recruitment and polarization of T cells that contribute to the hyperproliferation of KCs in the disease (Lowes et al., 2014). Across five patients with psoriasis, we reported a sub-cluster of DCs (*IRF4*$^+$ cDC2) that displays elevated expression of *CCL17*, *CCL22*, and *IL12B*, markers of cDC2s that have recently been shown to drive psoriatic inflammation in mice and humans through the recruitment of inflammatory T cells (Kim et al., 2018; Zaba et al., 2010). We further observed a population of fibroblasts in psoriasis that express *CCL19*, *TNFSF13B* (BAFF), and *CXCL12*. Notably, expression of *CCL19* and *BAFF* by synovial fibroblasts has been implicated in the progression of rheumatoid arthritis (Pickens et al., 2011; Reyes et al., 2008), but their relevance to psoriasis has yet to be described and will require further exploration.

Among ECs, we identified two clusters marked by expression of *SLC9A3R2*, a marker of endothelial homeostasis, and a signature of proliferation (venule clusters 3 and 4) (Bhattacharya et al., 2012). These proliferating EC clusters were enriched in acne, which is thought to arise in response to infection with *P. acnes*, resulting in the formation of lesions that resemble a wound after eruption of the hair follicle into the dermis (Beylot et al., 2014). Our findings suggest a prominent role for proliferative angiogenesis in the wound healing response that is seen in acne (Holland et al., 2004).

Differentiated KCs have been suggested to be the primary responders to IL-17A in psoriasis on the basis of previous studies by using *in vitro* KC systems, given larger effect sizes in differentiated compared with monolayer KCs (Chiricozzi et al., 2014). By cross-analyzing the data generated here against an IL-17 response signature in KCs, we have shown that IL-17 responses are observed in KCs from all layers of the epidermis, but that these responses are stronger in KCs derived from more differentiated layers of the psoriatic epidermis. By more precisely localizing IL-17 responses in psoriasis, our data might help to inform improved treatment strategies.

Beyond describing what can be gleaned about the cellular and molecular deviations associated with any one disease, we distinguished expression patterns associated with multiple diseases by looking across different inflammatory skin conditions to reveal common and unique response features. For example, our profiling uncovered a diverse group of cytotoxic cells that contains NK cells, $\gamma\delta$ T cells, and a sub-cluster of immature cytotoxic T cells that are derived primarily from leprosy and GA samples, which suggests common T cell programming between two forms of granulomatous inflammation. In GA, we observed multiple, distinct fibroblast populations that segregate between patients: one expressing cartilage associated proteins (e.g., *COMP*) and another expressing protease inhibitors and matrix metalloproteinases, respectively, which might reflect different forms of inflammation in distinct types of granuloma annulare (Piette and Rosenbach, 2016). In leprosy, meanwhile, we also detected a unique macrophage population defined by expression of extracellular proteases, as well as elevated expression of IFN-γ associated

transcriptional programs in LCs, which might reflect their role in response to infection (Pinheiro et al., 2018). Identifying how common cellular phenotypes affect disease pathophysiology in distinct cellular ecosystems is a critical avenue for future inquiry.

By charting the spectrum of skin inflammation at single-cell resolution, we have generated a resource that will serve as a reference for future inquiry into cutaneous biology. Among many of the cell types and states we identified, we found expression features that are shared across diseases, suggesting potentially common targetable biology; in others, our work revealed unique features, potentially associated with disease trajectory, for further inquiry. In future studies, Seq-Well S^3 will enable enhanced characterization of immune and parenchymal phenotypes in various types of inflammation across tissue compartments and how their interactions influence the development of human disease to reveal actionable therapeutic and prophylactic axes.

LIMITATIONS OF STUDY

Although Seq-Well S^3 results in improved capture of transcripts from each cell, there are important limitations associated with the method and the results presented here. First, in Seq-Well S^3, the size of the cDNAs after second-strand synthesis was shorter than that obtained in Seq-Well or Drop-Seq. This decreases the utility of Seq-Well S^3 for certain downstream applications that seek information from full-length transcripts or from their 5′ ends. Meanwhile, although we uncover multiple cellular phenotypes across inflammatory skin conditions, we were limited in our ability to distinguish inter-individual variation from disease-specific biology because of low numbers of samples per condition. Here, future studies with larger cohort sizes and/or matched unaffected skin samples from the same individual will be needed to resolve disease- from individual-specific features. Further, many of our findings are based on mRNA expression and correlative. Follow up experiments using protein detection and perturbation strategies will be necessary to corroborate significance. Nevertheless, the increased sensitivity of gene detection and transcript capture afforded by S^3 enhances the strength of the inferences that can be drawn from these types of single-cell data, as evidenced by the range of immune, stromal, and parenchymal cell states we describe across a spectrum of human skin inflammation.

STAR★METHODS

Detailed methods are provided in the online version of this paper and include the following:

- KEY RESOURCES TABLE
- RESOURCE AVAILABILITY
  - Lead Contact
  - Materials Availability
  - Data and Code Availability
- EXPERIMENTAL MODEL AND SUBJECT DETAILS
  - Cell Lines
  - PBMCs
  - Human Skin Samples
- METHOD DETAILS
  - Skin Biopsy Processing
  - Single-Cell Processing Pipeline
  - Templated Second-Strand Synthesis
  - PCR Amplification
  - Optimization of Second-Strand Synthesis
  - $CD4^+$ T Cell Comparisons of 10x Genomics, Seq-Well S^3, and Smart-Seq2
  - Sequencing Library Preparation
  - DNA Sequencing and Alignment of PBMC Optimization samples
  - Tissue Immunofluorescence Staining
- QUANTIFICATION AND STATISTICAL ANALYSIS
  - PBMC Comparison Experiments
  - Comparison of Gene Detection Rates
  - Sequencing and Alignment of Skin Samples
  - Cell Quality Filtering
  - Removal of Ambient RNA Contamination
  - Doublet Removal
  - Analysis of Combined Skin Dataset
  - Identification of T cell Sub-Clusters
  - T cell Receptor Detection and Clonal Expansion
  - Identification of Myeloid Heterogeneity
  - Identification of Endothelial Heterogeneity
  - Identification of Fibroblast Heterogeneity
  - Enrichment of Immune and Stromal Populations by Condition
  - Pseudo-temporal Reconstruction of Epidermal Keratinocytes
  - Keratinocyte Cytokine-Response Profiles
- ADDITIONAL RESOURCES

SUPPLEMENTAL INFORMATION

Supplemental Information can be found online at https://doi.org/10.1016/j.immuni.2020.09.015.

ACKNOWLEDGEMENTS

This work was supported in part by the NIH (P30CA14051, 5P01AI039671, 1DP2GM1194192, U19AI0899922, R01HL0957911, U54CA2173772, P01AI0396715, U24AI1186722, RM1HG0061931, R33CA2028201, R01AI1385461, R01HL1265541, R01DA0462771, U2CCA23319501, 5U19AI089992, R01AR060802, R01AI30025, P30AR075043, R01AI022553, R01AR040312, F30AI143160, and R01AR074302), the Bridge Project (a partnership between the Koch Institute for Integrative Cancer Research at MIT and the Dana-Farber/Harvard Cancer Center), and the Food Allergy Science Initiative at the Broad Institute. A.K.S. was supported by the Searle Scholars Program, the Beckman Young Investigator Program, the Pew-Stewart Scholars Program for Cancer Research, a Sloan Fellowship in Chemistry, and the Bill and Melinda Gates Foundation (OPP1139972, OPP1202327, OPP1137006, and OPP1202327). J.G was supported by the Taubman Medical Research Institute. J.O.-M. was supported by the Damon Runyon Cancer Research Foundation (Howard Hughes Medical Institute Fellow DRG-2274-16), and the Richard and Susan Smith Family Foundation.

AUTHOR CONTRIBUTIONS

T.K.H., M.W.H., T.M.G., R.L.M., J.C.L., and A.K.S. designed the study. T.K.H., M.H.W., T.D., D.W., P.A., and B.A. collected skin samples and performed single-cell sequencing experiments. S.S., L.C.T., and J.E.G. performed immunofluorescent staining. T.K.H., M.H.W., T.M.G., and F.M. analyzed data under the guidance of J.O.-M., R.L.M., J.C.L., and A.K.S. T.K.H., M.H.W., T.M.G., J.C.L., and A.K.S. wrote the manuscript with input from all authors.

DECLARATION OF INTERESTS

A.K.S. and J.C.L. have received compensation for consulting and SAB membership from Honeycomb Biotechnologies. A.K.S. has received compensation for consulting and SAB membership from Cellarity, Repertoire Immune Medicines, Orche Bio, and Dahlia Biosciences. T.M.G., T.K.H., M.H.W., A.K.S., and J.C.L. are co-inventors on a provisional patent application filed by MIT relating to the improved methodology described in this manuscript.

Received: July 1, 2019
Revised: May 21, 2020
Accepted: September 18, 2020
Published: October 13, 2020

REFERENCES

Angelo, M., Bendall, S.C., Finck, R., Hale, M.B., Hitzman, C., Borowsky, A.D., Levenson, R.M., Lowe, J.B., Liu, S.D., Zhao, S., et al. (2014). Multiplexed ion beam imaging of human breast tumors. Nat. Med. *20*, 436–442.

Aran, D., Looney, A.P., Liu, L., Wu, E., Fong, V., Hsu, A., Chak, S., Naikawadi, R.P., Wolters, P.J., Abate, A.R., et al. (2019). Reference-based analysis of lung single-cell sequencing reveals a transitional profibrotic macrophage. Nat. Immunol. *20*, 163–172.

Balin, S.J., Pellegrini, M., Klechevsky, E., Won, S.T., Weiss, D.I., Choi, A.W., Hakimian, J., Lu, J., Ochoa, M.T., and Bloom, B.R. (2018). Human antimicrobial cytotoxic T lymphocytes, defined by NK receptors and antimicrobial proteins, kill intracellular bacteria. Science immunology *3*, eaat7668.

Best, J.A., Blair, D.A., Knell, J., Yang, E., Mayya, V., Doedens, A., Dustin, M.L., Goldrath, A.W., Monach, P., and Shinton, S.A.; Immunological Genome Project Consortium (2013). Transcriptional insights into the CD8(+) T cell response to infection and memory T cell formation. Nat. Immunol. *14*, 404–412.

Beylot, C., Auffret, N., Poli, F., Claudel, J.P., Leccia, M.T., Del Giudice, P., and Dreno, B. (2014). Propionibacterium acnes: an update on its role in the pathogenesis of acne. J. Eur. Acad. Dermatol. Venereol. *28*, 271–278.

Bezman, N.A., Kim, C.C., Sun, J.C., Min-Oo, G., Hendricks, D.W., Kamimura, Y., Best, J.A., Goldrath, A.W., Lanier, L.L., and Gautier, E.L.; Immunological Genome Project Consortium (2012). Molecular definition of the identity and activation of natural killer cells. Nat. Immunol. *13*, 1000–1009.

Bhattacharya, R., Wang, E., Dutta, S.K., Vohra, P.K., e, G., Prakash, Y.S., and Mukhopadhyay, D. (2012). NHERF-2 maintains endothelial homeostasis. Blood *119*, 4798–4806.

Brckalo, T., Calzetti, F., Pérez-Cabezas, B., Borràs, F.E., Cassatella, M.A., and López-Botet, M. (2010). Functional analysis of the CD300e receptor in human monocytes and myeloid dendritic cells. Eur. J. Immunol. *40*, 722–732.

Cao, J., Packer, J.S., Ramani, V., Cusanovich, D.A., Huynh, C., Daza, R., Qiu, X., Lee, C., Furlan, S.N., Steemers, F.J., et al. (2017). Comprehensive single-cell transcriptional profiling of a multicellular organism. Science *357*, 661–667.

Cheng, J.B., Sedgewick, A.J., Finnegan, A.I., Harirchian, P., Lee, J., Kwon, S., Fassett, M.S., Golovato, J., Gray, M., Ghadially, R., et al. (2018). Transcriptional programming of normal and inflamed human epidermis at single-cell resolution. Cell Rep. *25*, 871–883.

Chiricozzi, A., Nograles, K.E., Johnson-Huang, L.M., Fuentes-Duculan, J., Cardinale, I., Bonifacio, K.M., Gulati, N., Mitsui, H., Guttman-Yassky, E., Suárez-Fariñas, M., and Krueger, J.G. (2014). IL-17 induces an expanded range of downstream genes in reconstituted human epidermis model. PLoS ONE *9*, e90284.

Consortium, T.M.; Tabula Muris Consortium; Overall coordination; Logistical coordination; Organ collection and processing; Library preparation and sequencing; Computational data analysis; Cell type annotation; Writing group; Supplemental text writing group; Principal investigators (2018). Single-cell transcriptomics of 20 mouse organs creates a Tabula Muris. Nature *562*, 367–372.

Di Rosa, M., Malaguarnera, G., De Gregorio, C., Drago, F., and Malaguarnera, L. (2013). Evaluation of CHI3L-1 and CHIT-1 expression in differentiated and polarized macrophages. Inflammation *36*, 482–492.

Diani, M., Altomare, G., and Reali, E. (2015). T cell responses in psoriasis and psoriatic arthritis. Autoimmun. Rev. *14*, 286–292.

Ding, J., Adiconis, X., Simmons, S.K., Kowalczyk, M.S., Hession, C.C., Marjanovic, N.D., Hughes, T.K., Wadsworth, M.H., Burks, T., and Nguyen, L.T. (2020). Systematic comparison of single-cell and single-nucleus RNA-sequencing methods. Nat. Biotechnol. 1–10, https://doi.org/10.1038/s41587-020-0465-8.

Driskell, R.R., and Watt, F.M. (2015). Understanding fibroblast heterogeneity in the skin. Trends Cell Biol. *25*, 92–99.

Driskell, R.R., Lichtenberger, B.M., Hoste, E., Kretzschmar, K., Simons, B.D., Charalambous, M., Ferron, S.R., Herault, Y., Pavlovic, G., Ferguson-Smith, A.C., and Watt, F.M. (2013). Distinct fibroblast lineages determine dermal architecture in skin development and repair. Nature *504*, 277–281.

Dutertre, C.-A., Becht, E., Irac, S.E., Khalilnezhad, A., Narang, V., Khalilnezhad, S., Ng, P.Y., van den Hoogen, L.L., Leong, J.Y., and Lee, B. (2019). Single-cell analysis of human mononuclear phagocytes reveals subset-defining markers and identifies circulating inflammatory dendritic cells. Immunity *51*, 573–589, e578.

Fu, X., Khalil, H., Kanisicak, O., Boyer, J.G., Vagnozzi, R.J., Maliken, B.D., Sargent, M.A., Prasad, V., Valiente-Alandi, I., Blaxall, B.C., and Molkentin, J.D. (2018). Specialized fibroblast differentiated states underlie scar formation in the infarcted mouse heart. J. Clin. Invest. *128*, 2127–2143.

Fuchs, E. (1990). Epidermal differentiation: the bare essentials. J. Cell Biol. *111*, 2807–2814.

Fuentes-Duculan, J., Suárez-Fariñas, M., Zaba, L.C., Nograles, K.E., Pierson, K.C., Mitsui, H., Pensabene, C.A., Kzhyshkowska, J., Krueger, J.G., and Lowes, M.A. (2010). A subpopulation of CD163-positive macrophages is classically activated in psoriasis. J. Invest. Dermatol. *130*, 2412–2422.

Gierahn, T.M., Wadsworth, M.H., 2nd, Hughes, T.K., Bryson, B.D., Butler, A., Satija, R., Fortune, S., Love, J.C., and Shalek, A.K. (2017). Seq-Well: portable, low-cost RNA sequencing of single cells at high throughput. Nat. Methods *14*, 395–398.

Guilliams, M., Bruhns, P., Saeys, Y., Hammad, H., and Lambrecht, B.N. (2014). The function of Fcγ receptors in dendritic cells and macrophages. Nat. Rev. Immunol. *14*, 94–108.

Guilliams, M., Dutertre, C.-A., Scott, C.L., McGovern, N., Sichien, D., Chakarov, S., Van Gassen, S., Chen, J., Poidinger, M., De Prijck, S., et al. (2016). Unsupervised high-dimensional analysis aligns dendritic cells across tissues and species. Immunity *45*, 669–684.

Haghverdi, L., Buettner, F., and Theis, F.J. (2015). Diffusion maps for high-dimensional single-cell analysis of differentiation data. Bioinformatics *31*, 2989–2998.

Hazell, G.G., Peachey, A.M., Teasdale, J.E., Sala-Newby, G.B., Angelini, G.D., Newby, A.C., and White, S.J. (2016). PI16 is a shear stress and inflammation-regulated inhibitor of MMP2. Sci. Rep. *6*, 39553.

He, H., Suryawanshi, H., Morozov, P., Gay-Mimbrera, J., Del Duca, E., Kim, H.J., Kameyama, N., Estrada, Y., Der, E., Krueger, J.G., et al. (2020). Single-cell transcriptome analysis of human skin identifies novel fibroblast subpopulation and enrichment of immune subsets in atopic dermatitis. J. Allergy Clin. Immunol. *145*, 1615–1628.

Holland, D.B., Jeremy, A.H., Roberts, S.G., Seukeran, D.C., Layton, A.M., and Cunliffe, W.J. (2004). Inflammation in acne scarring: a comparison of the responses in lesions from patients prone and not prone to scar. Br. J. Dermatol. *150*, 72–81.

Hunger, R.E., Sieling, P.A., Ochoa, M.T., Sugaya, M., Burdick, A.E., Rea, T.H., Brennan, P.J., Belisle, J.T., Blauvelt, A., Porcelli, S.A., and Modlin, R.L. (2004). Langerhans cells utilize CD1a and langerin to efficiently present nonpeptide antigens to T cells. J. Clin. Invest. *113*, 701–708.

Huth, S., Heise, R., Vetter-Kauczok, C.S., Skazik, C., Marquardt, Y., Czaja, K., Knüchel, R., Merk, H.F., Dahl, E., and Baron, J.M. (2015). Inter-α-trypsin inhibitor heavy chain 5 (ITIH5) is overexpressed in inflammatory skin diseases and affects epidermal morphology in constitutive knockout mice and murine 3D skin models. Exp. Dermatol. *24*, 663–668.

CellPress
OPEN ACCESS

Islam, S., Kjällquist, U., Moliner, A., Zajac, P., Fan, J.-B., Lönnerberg, P., and Linnarsson, S. (2012). Highly multiplexed and strand-specific single-cell RNA 5¢ end sequencing. Nat. Protoc. *7*, 813–828.

Ivanov, I.I., McKenzie, B.S., Zhou, L., Tadokoro, C.E., Lepelley, A., Lafaille, J.J., Cua, D.J., and Littman, D.R. (2006). The orphan nuclear receptor RORgammat directs the differentiation program of proinflammatory IL-17+ T helper cells. Cell *126*, 1121–1133.

Jin, P., Han, T.H., Ren, J., Saunders, S., Wang, E., Marincola, F.M., and Stroncek, D.F. (2010). Molecular signatures of maturing dendritic cells: implications for testing the quality of dendritic cell therapies. J. Transl. Med. *8*, 4.

Joost, S., Zeisel, A., Jacob, T., Sun, X., La Manno, G., Lönnerberg, P., Linnarsson, S., and Kasper, M. (2016). Single-cell transcriptomics reveals that differentiation and spatial signatures shape epidermal and hair follicle heterogeneity. Cell systems *3*, 221–237.

Kapteyn, J., He, R., McDowell, E.T., and Gang, D.R. (2010). Incorporation of non-natural nucleotides into template-switching oligonucleotides reduces background and improves cDNA synthesis from very small RNA samples. BMC Genomics *11*, 413.

Kashem, S.W., Riedl, M.S., Yao, C., Honda, C.N., Vulchanova, L., and Kaplan, D.H. (2015). Nociceptive sensory fibers drive interleukin-23 production from CD301b+ dermal dendritic cells and drive protective cutaneous immunity. Immunity *43*, 515–526.

Kim, T.-G., Kim, S.H., Park, J., Choi, W., Sohn, M., Na, H.Y., Lee, M., Lee, J.W., Kim, S.M., Kim, D.-Y., et al. (2018). Skin-Specific $CD301b^+$ Dermal Dendritic Cells Drive IL-17-Mediated Psoriasis-Like Immune Response in Mice. J. Invest. Dermatol. *138*, 844–853.

Kim, D., Kobayashi, T., Voisin, B., Jo, J.-H., Sakamoto, K., Jin, S.-P., Kelly, M., Pasieka, H.B., Naff, J.L., Meyerle, J.H., et al. (2020). Targeted therapy guided by single-cell transcriptomic analysis in drug-induced hypersensitivity syndrome: a case report. Nat. Med. *26*, 236–243.

Klein, A.M., Mazutis, L., Akartuna, I., Tallapragada, N., Veres, A., Li, V., Peshkin, L., Weitz, D.A., and Kirschner, M.W. (2015). Droplet barcoding for single-cell transcriptomics applied to embryonic stem cells. Cell *161*, 1187–1201.

Kumamoto, Y., Linehan, M., Weinstein, J.S., Laidlaw, B.J., Craft, J.E., and Iwasaki, A. (2013). $CD301b^+$ dermal dendritic cells drive T helper 2 cell-mediated immunity. Immunity *39*, 733–743.

Lanna, A., Gomes, D.C., Muller-Durovic, B., McDonnell, T., Escors, D., Gilroy, D.W., Lee, J.H., Karin, M., and Akbar, A.N. (2017). A sestrin-dependent Erk-Jnk-p38 MAPK activation complex inhibits immunity during aging. Nat. Immunol. *18*, 354–363.

Li, H., Handsaker, B., Wysoker, A., Fennell, T., Ruan, J., Homer, N., Marth, G., Abecasis, G., and Durbin, R.; 1000 Genome Project Data Processing Subgroup (2009). The sequence alignment/map format and SAMtools. Bioinformatics *25*, 2078–2079.

Li, B., Tsoi, L.C., Swindell, W.R., Gudjonsson, J.E., Tejasvi, T., Johnston, A., Ding, J., Stuart, P.E., Xing, X., Kochkodan, J.J., et al. (2014). Transcriptome analysis of psoriasis in a large case-control sample: RNA-seq provides insights into disease mechanisms. J. Invest. Dermatol. *134*, 1828–1838.

Liu, X., Wang, Y., Lu, H., Li, J., Yan, X., Xiao, M., Hao, J., Alekseev, A., Khong, H., Chen, T., et al. (2019). Genome-wide analysis identifies NR4A1 as a key mediator of T cell dysfunction. Nature *567*, 525–529.

Lopez, D., Montoya, D., Ambrose, M., Lam, L., Briscoe, L., Adams, C., Modlin, R.L., and Pellegrini, M. (2017). SaVanT: a web-based tool for the sample-level visualization of molecular signatures in gene expression profiles. BMC Genomics *18*, 824.

Lowes, M.A., Suárez-Fariñas, M., and Krueger, J.G. (2014). Immunology of psoriasis. Annu. Rev. Immunol. *32*, 227–255.

Lun, A.T.L., Riesenfeld, S., Andrews, T., Dao, T.P., Gomes, T., and Marioni, J.C.; participants in the 1st Human Cell Atlas Jamboree (2019). EmptyDrops: distinguishing cells from empty droplets in droplet-based single-cell RNA sequencing data. Genome Biol. *20*, 63.

Macosko, E.Z., Basu, A., Satija, R., Nemesh, J., Shekhar, K., Goldman, M., Tirosh, I., Bialas, A.R., Kamitaki, N., Martersteck, E.M., et al. (2015). Highly parallel genome-wide expression profiling of individual cells using nanoliter droplets. Cell *161*, 1202–1214.

Malissen, B., Tamoutounour, S., and Henri, S. (2014). The origins and functions of dendritic cells and macrophages in the skin. Nat. Rev. Immunol. *14*, 417–428.

McGinnis, C.S., Patterson, D.M., Winkler, J., Conrad, D.N., Hein, M.Y., Srivastava, V., Hu, J.L., Murrow, L.M., Weissman, J.S., Werb, Z., et al. (2019). MULTI-seq: sample multiplexing for single-cell RNA sequencing using lipid-tagged indices. Nat. Methods *16*, 619–626.

Micke, P., Kappert, K., Ohshima, M., Sundquist, C., Scheidl, S., Lindahl, P., Heldin, C.-H., Botling, J., Ponten, F., and Östman, A. (2007). In situ identification of genes regulated specifically in fibroblasts of human basal cell carcinoma. J. Invest. Dermatol. *127*, 1516–1523.

Montoro, D.T., Haber, A.L., Biton, M., Vinarsky, V., Lin, B., Birket, S.E., Yuan, F., Chen, S., Leung, H.M., Villoria, J., et al. (2018). A revised airway epithelial hierarchy includes CFTR-expressing ionocytes. Nature *560*, 319–324.

Oakes, T., Heather, J.M., Best, K., Byng-Maddick, R., Husovsky, C., Ismail, M., Joshi, K., Maxwell, G., Noursadeghi, M., Riddell, N., et al. (2017). Quantitative characterization of the T cell receptor repertoire of naive and memory subsets using an integrated experimental and computational pipeline which is robust, economical, and versatile. Front. Immunol. *8*, 1267.

Ordovas-Montanes, J., Dwyer, D.F., Nyquist, S.K., Buchheit, K.M., Vukovic, M., Deb, C., Wadsworth, M.H., 2nd, Hughes, T.K., Kazer, S.W., Yoshimoto, E., et al. (2018). Allergic inflammatory memory in human respiratory epithelial progenitor cells. Nature *560*, 649–654.

Pellegrini, G., Dellambra, E., Golisano, O., Martinelli, E., Fantozzi, I., Bondanza, S., Ponzin, D., McKeon, F., and De Luca, M. (2001). p63 identifies keratinocyte stem cells. Proc. Natl. Acad. Sci. USA *98*, 3156–3161.

Picelli, S., Björklund, Å.K., Faridani, O.R., Sagasser, S., Winberg, G., and Sandberg, R. (2013). Smart-seq2 for sensitive full-length transcriptome profiling in single cells. Nat. Methods *10*, 1096–1098.

Picelli, S., Faridani, O.R., Björklund, Å.K., Winberg, G., Sagasser, S., and Sandberg, R. (2014). Full-length RNA-seq from single cells using Smart-seq2. Nat. Protoc. *9*, 171–181.

Pickens, S.R., Chamberlain, N.D., Volin, M.V., Pope, R.M., Mandelin, A.M., 2nd, and Shahrara, S. (2011). Characterization of CCL19 and CCL21 in rheumatoid arthritis. Arthritis Rheum. *63*, 914–922.

Piette, E.W., and Rosenbach, M. (2016). Granuloma annulare: Clinical and histologic variants, epidemiology, and genetics. J. Am. Acad. Dermatol. *75*, 457–465.

Pinheiro, R.O., Schmitz, V., Silva, B.J.A., Dias, A.A., de Souza, B.J., de Mattos Barbosa, M.G., de Almeida Esquenazi, D., Pessolani, M.C.V., and Sarno, E.N. (2018). Innate immune responses in leprosy. Front. Immunol. *9*, 518.

Prakadan, S.M., Shalek, A.K., and Weitz, D.A. (2017). Scaling by shrinking: empowering single-cell 'omics' with microfluidic devices. Nat. Rev. Genet. *18*, 345–361.

Puel, A., Ziegler, S.F., Buckley, R.H., and Leonard, W.J. (1998). Defective IL7R expression in T(-)B(+)NK(+) severe combined immunodeficiency. Nat. Genet. *20*, 394–397.

Reyes, L.I., León, F., González, P., Rozas, M.F., Labarca, C., Segovia, A., Neira, O., and Naves, R. (2008). Dexamethasone inhibits BAFF expression in fibroblast-like synoviocytes from patients with rheumatoid arthritis. Cytokine *42*, 170–178.

Rodriques, S.G., Stickels, R.R., Goeva, A., Martin, C.A., Murray, E., Vanderburg, C.R., Welch, J., Chen, L.M., Chen, F., and Macosko, E.Z. (2019). Slide-seq: A scalable technology for measuring genome-wide expression at high spatial resolution. Science *363*, 1463–1467.

Romani, N., Holzmann, S., Tripp, C.H., Koch, F., and Stoitzner, P. (2003). Langerhans cells - dendritic cells of the epidermis. APMIS *111*, 725–740.

Rosenberg, A.B., Roco, C.M., Muscat, R.A., Kuchina, A., Sample, P., Yao, Z., Graybuck, L.T., Peeler, D.J., Mukherjee, S., Chen, W., et al. (2018). Single-cell profiling of the developing mouse brain and spinal cord with split-pool barcoding. Science *360*, 176–182.

Saelens, W., Cannoodt, R., Todorov, H., and Saeys, Y. (2019). A comparison of single-cell trajectory inference methods. Nat. Biotechnol. *37*, 547–554.

Šahmatova, L., Sügis, E., Šunina, M., Hermann, H., Prans, E., Pihlap, M., Abram, K., Rebane, A., Peterson, H., Peterson, P., et al. (2017). Signs of innate immune activation and premature immunosenescence in psoriasis patients. Sci. Rep. *7*, 7553.

Saini, C., Ramesh, V., and Nath, I. (2013). CD4+ Th17 cells discriminate clinical types and constitute a third subset of non Th1, Non Th2 T cells in human leprosy. PLoS Negl. Trop. Dis. *7*, e2338.

Saini, C., Siddiqui, A., Ramesh, V., and Nath, I. (2016). Leprosy reactions show increased Th17 cell activity and reduced FOXP3+ Tregs with concomitant decrease in TGF-β and increase in IL-6. PLoS Negl. Trop. Dis. *10*, e0004592.

Sandilands, A., Sutherland, C., Irvine, A.D., and McLean, W.H. (2009). Filaggrin in the frontline: role in skin barrier function and disease. J. Cell Sci. *122*, 1285–1294.

Sasagawa, Y., Nikaido, I., Hayashi, T., Danno, H., Uno, K.D., Imai, T., and Ueda, H.R. (2013). Quartz-Seq: a highly reproducible and sensitive single-cell RNA sequencing method, reveals non-genetic gene-expression heterogeneity. Genome Biol. *14*, R31.

Satija, R., Farrell, J.A., Gennert, D., Schier, A.F., and Regev, A. (2015). Spatial reconstruction of single-cell gene expression data. Nat. Biotechnol. *33*, 495–502.

Schlitzer, A., McGovern, N., Teo, P., Zelante, T., Atarashi, K., Low, D., Ho, A.W., See, P., Shin, A., Wasan, P.S., et al. (2013). IRF4 transcription factor-dependent CD11b+ dendritic cells in human and mouse control mucosal IL-17 cytokine responses. Immunity *38*, 970–983.

Schön, M.P., Zollner, T.M., and Boehncke, W.-H. (2003). The molecular basis of lymphocyte recruitment to the skin: clues for pathogenesis and selective therapies of inflammatory disorders. J. Invest. Dermatol. *121*, 951–962.

Shao, L., Liu, Y., Mei, J., Li, D., Chen, L., Pan, Q., Zhang, S., Dai, X., Liang, J., Sun, S., and Wang, J. (2020). High-throughput sequencing reveals the diversity of TCR β chain CDR3 repertoire in patients with severe acne. Mol. Immunol. *120*, 23–31.

Shishkin, A.A., Giannoukos, G., Kucukural, A., Ciulla, D., Busby, M., Surka, C., Chen, J., Bhattacharyya, R.P., Rudy, R.F., Patel, M.M., et al. (2015). Simultaneous generation of many RNA-seq libraries in a single reaction. Nat. Methods *12*, 323–325.

Smillie, C.S., Biton, M., Ordovas-Montanes, J., Sullivan, K.M., Burgin, G., Graham, D.B., Herbst, R.H., Rogel, N., Slyper, M., and Waldman, J. (2019). Intra-and inter-cellular rewiring of the human colon during ulcerative colitis. Cell *178*, 714–730.

Söderström, K., Stein, E., Colmenero, P., Purath, U., Müller-Ladner, U., de Matos, C.T., Tarner, I.H., Robinson, W.H., and Engleman, E.G. (2010). Natural killer cells trigger osteoclastogenesis and bone destruction in arthritis. Proc. Natl. Acad. Sci. USA *107*, 13028–13033.

Stoeckius, M., Zheng, S., Houck-Loomis, B., Hao, S., Yeung, B.Z., Mauck, W.M., 3rd, Smibert, P., and Satija, R. (2018). Cell Hashing with barcoded antibodies enables multiplexing and doublet detection for single cell genomics. Genome Biol. *19*, 224.

Stutte, S., Quast, T., Gerbitzki, N., Savinko, T., Novak, N., Reifenberger, J., Homey, B., Kolanus, W., Alenius, H., and Förster, I. (2010). Requirement of CCL17 for CCR7- and CXCR4-dependent migration of cutaneous dendritic cells. Proc. Natl. Acad. Sci. USA *107*, 8736–8741.

Tabib, T., Morse, C., Wang, T., Chen, W., and Lafyatis, R. (2018). SFRP2/DPP4 and FMO1/LSP1 define major fibroblast populations in human skin. J. Invest. Dermatol. *138*, 802–810.

Thiriot, A., Perdomo, C., Cheng, G., Novitzky-Basso, I., McArdle, S., Kishimoto, J.K., Barreiro, O., Mazo, I., Triboulet, R., Ley, K., et al. (2017). Differential DARC/ACKR1 expression distinguishes venular from non-venular endothelial cells in murine tissues. BMC Biol. *15*, 45.

Tsoi, L.C., Rodriguez, E., Degenhardt, F., Baurecht, H., Wehkamp, U., Volks, N., Szymczak, S., Swindell, W.R., Sarkar, M.K., Raja, K., et al. (2019). Atopic Dermatitis Is an IL-13-Dominant Disease with Greater Molecular Heterogeneity Compared to Psoriasis. J. Invest. Dermatol. *139*, 1480–1489.

Tu, A.A., Gierahn, T.M., Monian, B., Morgan, D.M., Mehta, N.K., Ruiter, B., Shreffler, W.G., Shalek, A.K., and Love, J.C. (2019). TCR sequencing paired with massively parallel 3¢ RNA-seq reveals clonotypic T cell signatures. Nat. Immunol. *20*, 1692–1699.

Uhlén, M., Fagerberg, L., Hallström, B.M., Lindskog, C., Oksvold, P., Mardinoglu, A., Sivertsson, Å., Kampf, C., Sjöstedt, E., Asplund, A., et al. (2015). Proteomics. Tissue-based map of the human proteome. Science *347*, 1260419.

van Galen, P., Hovestadt, V., Wadsworth, M.H., II, Hughes, T.K., Griffin, G.K., Battaglia, S., Verga, J.A., Stephansky, J., Pastika, T.J., and Story, J.L. (2019). Single-cell RNA-seq reveals AML hierarchies relevant to disease progression and immunity. Cell *176*, 1265–1281.

Vento-Tormo, R., Efremova, M., Botting, R.A., Turco, M.Y., Vento-Tormo, M., Meyer, K.B., Park, J.-E., Stephenson, E., Polański, K., Goncalves, A., et al. (2018). Single-cell reconstruction of the early maternal-fetal interface in humans. Nature *563*, 347–353.

Vieira Braga, F.A., Kar, G., Berg, M., Carpaij, O.A., Polanski, K., Simon, L.M., Brouwer, S., Gomes, T., Hesse, L., Jiang, J., et al. (2019). A cellular census of human lungs identifies novel cell states in health and in asthma. Nat. Med. *25*, 1153–1163.

Villani, A.-C., Satija, R., Reynolds, G., Sarkizova, S., Shekhar, K., Fletcher, J., Griesbeck, M., Butler, A., Zheng, S., and Lazo, S. (2017). Single-cell RNA-seq reveals new types of human blood dendritic cells, monocytes, and progenitors. Science *356*, eaah4573.

von Andrian, U.H., and Mempel, T.R. (2003). Homing and cellular traffic in lymph nodes. Nat. Rev. Immunol. *3*, 867–878.

Wolf, F.A., Angerer, P., and Theis, F.J. (2018). SCANPY: large-scale single-cell gene expression data analysis. Genome Biol. *19*, 15.

Young, M.D., and Behjati, S. (2018). SoupX removes ambient RNA contamination from droplet based single cell RNA sequencing data. bioRxiv, 303727.

Zaba, L.C., Fuentes-Duculan, J., Eungdamrong, N.J., Johnson-Huang, L.M., Nograles, K.E., White, T.R., Pierson, K.C., Lentini, T., Suárez-Fariñas, M., and Lowes, M.A. (2010). Identification of TNF-related apoptosis-inducing ligand and other molecules that distinguish inflammatory from resident dendritic cells in patients with psoriasis. Journal of Allergy and Clinical Immunology *125*, 1261–1268.

Zajac, P., Islam, S., Hochgerner, H., Lönnerberg, P., and Linnarsson, S. (2013). Base preferences in non-templated nucleotide incorporation by MMLV-derived reverse transcriptases. PLoS ONE *8*, e85270.

Zhang, L., Yu, X., Zheng, L., Zhang, Y., Li, Y., Fang, Q., Gao, R., Kang, B., Zhang, Q., Huang, J.Y., et al. (2018). Lineage tracking reveals dynamic relationships of T cells in colorectal cancer. Nature *564*, 268–272.

STAR★METHODS

KEY RESOURCES TABLE

REAGENT or RESOURCE	SOURCE	IDENTIFIER
Antibodies		
IL-17RA	Lifespan Bioscience	Cat#LS-C359381
IL-17RC	Lifespan Bioscience	Cat#LS-400522
FOSL	Boster	Cat# A03927
IL-36G	Santa Cruz Biotechnology	Cat#Sc-80056; RRID: AB_2124893
TNFAIP3	Abcam	Cat#Ab74037; RRID: AB_1524499
APOBEC3A	Lifespan Bioscience	Cat#LS-C98892-400
Biological Samples		
Skin Biopsies	UCLA	N/A
Skin Biopsies	University of Michigan	N/A
PBMCs	MGH	N/A
Chemicals, Peptides, and Recombinant Proteins		
Maxima H-RT and Buffer	ThermoFisher Scientific	Cat#EP0751
dNTPs	New England Biolabs	Cat#N0447L
Polyethylene Glycol 8000	Fisher Scientific	Cat#BP233-1
SUPERase*In RNase inhibitor	ThermoFisher Scientific	Cat#AM2696
Exonuclease I and Buffer	New England Biolabs	Cat#M0293S
1M Tris-HCl, pH 8.0	ThermoFisher Scientific	Cat#15568025
Klenow Fragment (3'®5' exo-)	New England Biolabs	Cat#M0212L
KAPA 2x HiFi HotStart PCR mix	Kapa Biosystems	Cat#KK2602
Nextera XT Kit	Illumina, Inc	Cat#FC-131-1096
UltraPure DNase/Rnase-Free Distilled Water	ThermoFisher Scientific	Cat#10977015
TWEEN 20	Fisher Scientific	Cat#BP337-100
Sodium Dodecyl Sulfate (SDS) Solution	Sigma	Cat#71736-100mL
TE Buffer	ThermoFisher Scientific	Cat#12090015
Critical Commercial Assays		
mRNA Capture Beads	Chemgenes Corp.	Cat#MACOSKO-2011-10B
KAPA 2x HiFi HotStart PCR mix	Kapa Biosystems	Cat#KK2602
NextSeq500 (75 cycles)	Illumina	Cat#20024906
Nova-Seq S2 (100 cycles)	Illumina	Cat#20028316
Deposited Data		
Raw and Processed data	GEO	GEO: GSE150672
Experimental Models: Cell Lines		
HEK293	ATCC	CRL-1573
NIH/3T3s	ATCC	CRL-1658
Oligonucleotides		
Template-Switching Oligo: AAGCAGTGGTATCAACGCAG AG TGAATrGrGrG	This Paper	N/A
SMART PCR Primer: AAGCAGTGGTATCAACGCAGAGT	This Paper	N/A
S^3 Randomer:AAGCAGTGGTAT CAACGCAGAGTGANNNGGNNNB	This Paper	N/A

(*Continued on next page*)

Continued		
REAGENT or RESOURCE	SOURCE	IDENTIFIER
P5-SMART Hybrid Oligo: AATGATACGGCGACCACCG AGATCTACACGCCTGTCCGC G-GAAGCAG TGGTA TCAACGCAGAGT*A*C	This Paper	N/A
Custom Read 1 Primer: GCCTGTCCGCGGAAGC AGTGGTATCAACGCAGAGTAC	This Paper	N/A
Software and Algorithms		
Seurat	Satija et al. 2015	http://satijalab.org/seurat/
SCANPY	Wolf et al. 2018	http://github.com/theislab/Scanpy

RESOURCE AVAILABILITY

Lead Contact

Additional information and requests for resources and reagents should be directed to the Lead Contact: Alex K. Shalek (shalek@mit.edu).

Materials Availability

All unique reagents generated are listed in the key resources table along with the supplemental protocol. Additional requests for resources and reagents can be directed to the Lead Contact.

Data and Code Availability

Raw and processed data are available on the gene expression omnibus (GEO) in GSE150672. Processed data are further available in an interactive format as part of the Alexandria Project (https://singlecell.broadinstitute.org/single_cell?scpbr=the-alexandria-project). Additional code is available upon request from the Lead Contact.

EXPERIMENTAL MODEL AND SUBJECT DETAILS

Cell Lines

HEK293 and NIH-3T3 cell lines used in species mixing experiments were obtained from ATCC. Cell lines were cultured in DMEM supplemented with 10% FBS at 37C with 5% CO2.

PBMCs

Peripheral blood mononuclear cells (PBMCs) used in optimization and comparison experiments were obtained from Massachusetts General Hospital. Aliquots of $1.0x10^6$ PBMCs were frozen in 90% FBS with 10% DMSO and thawed prior to use in experiments.

Human Skin Samples

Skin biopsies were obtained from a total of 16 patients at the University of California, Los Angeles and University of Southern California Hansen's Clinic, while an additional 3 samples were obtained from the University of Michigan. Informed written consent was obtained from human subjects under a protocol approved by the institutional review boards of the University of Michigan and University of California Los Angeles (UCLA). This study was conducted according to the Declaration of Helsinki Principles.

METHOD DETAILS

Skin Biopsy Processing

For each sample, a 4-mm punch biopsy was obtained following local anesthesia and was placed immediately into 10 mL of RPMI on ice. Initially, skin biopsies were incubated in 5 mL of a 0.4% Dispase II solution (Roche Inc.) at 37°C for 1 hour with vigorous shaking. The dermis and epidermis were then carefully separated using forceps and transferred to separate tubes for additional processing. Epidermal samples were placed in 3 mL of 0.25% Trypsin and 10 U/mL DNAse for 30 minutes at 37°C. Trypsin was neutralized with 3 mL of fetal calf serum (FCS), and the tissue was passed through a 70-micron nylon cell strainer which was washed with 5 mL of RPMI. Epidermal cells were then pelleted at 300xg for 10 minutes and counted. Dermal samples were minced with a scalpel and incubated in a solution of 0.4% collagenase 2 and 10 U/mL DNAse for 2 hours at 37°C with agitation. The cell suspension was passed through a 70-micron cell strainer and washed with 5 mL of RPMI. Cells were pelleted at 300 xg for 10 minutes, resuspended in 1 mL of RPMI and counted. MACS enrichment for $CD1A^+$ cells was performed for epidermal fractions from biopsies from normal skin 1 and leprosy 1.

Single-Cell Processing Pipeline

We utilized Seq-Well, a massively parallel, low-input scRNA-seq platform for clinical samples, to capture the transcriptome of single cells. A complete, updated protocol for Seq-Well S^3 is included as a Supplementary Protocol and is hosted on the Shalek Lab website (www.shaleklab.com). Briefly, 10-15,000 cells were loaded onto a functionalized-polydimethylsiloxane (PDMS) array preloaded with uniquely barcoded mRNA capture beads (Chemgenes; MACOSKO-2011-10). After cells had settled into wells, the array was then sealed with a hydroxylated polycarbonate membrane with a pore size of 10 nm, facilitating buffer exchange while confining biological molecules within each well. Following membrane-sealing, subsequent buffer exchange permits cell lysis, mRNA transcript hybridization to beads, and bead removal before proceeding with reverse transcription. The obtained bead-bound cDNA product then underwent Exonuclease I treatment (New England Biolabs; M0293M) to remove excess primer before proceeding with second-strand synthesis.

Templated Second-Strand Synthesis

Following Exonuclease I treatment, beads were washed once with 500 μL of a TE-SDS (0.5% SDS) solution, and twice in 500 μL of a TE-Tween (0.01% Tween) solution. After the second TE-TW wash the beads were solvated with 500 μL of 0.1 M NaOH and mixed for 5 minutes at room temperature using an end-over-end rotator with intermittent agitation to denature the mRNA-cDNA hybrid product on the bead. Following denaturing, the NaOH was removed and beads were washed once with 1 M TE, and then combined with a mastermix consisting of 40 μL 5x maxima RT buffer, 80 μL 30% PEG8000 solution, 20 μL 10mM dNTPs, 2 μL 1 mM dn-SMART oligo, 5 μL Klenow Exo-, and 53 μl of DI ultrapure water. Second-strand synthesis was carried out by incubating the beads for 1 hour at 37°C with end-over-end rotation and intermittent agitation. Following incubation, beads were sequentially washed twice with 0.5 mL of TE buffer with 0.01% Tween 20, and once with 0.5 mL of TE. Immediately prior to PCR amplification, beads were washed once with 0.5 mL of water and resuspended in 0.5 mL of water.

PCR Amplification

After second-strand synthesis, PCR amplification was performed using KAPA HiFi PCR Mix (Kapa Biosystems KK2602). Specifically, a 40 μL PCR Mastermix consisting of 25 μL of KAPA 5X Mastermix, 0.4 μL of 100 μM ISPCR oligo, and 14.6 μL of nuclease-free water was combined with 2,000 beads per reaction. For each sample, the total number of PCR reactions performed varied based on the number of beads recovered following second-strand synthesis. PCR amplification was performed using the following cycling conditions: an initial denaturation at 95°C for 3 minutes, then 4 cycles of 98°C for 20 seconds, 65°C for 45 seconds, and 72°C for 3 minutes, followed by 9-12 cycles of 98°C or 20 seconds, 67°C or 20 second, and 72°C for 3 minutes, and then a final extension of 72°C for 5 minutes. Following PCR amplification, WTA products were isolated through two rounds of SPRI purification using Ampure Spri beads (Beckman Coulter, Inc.) at both 0.6x and 0.8x volumetric ratio and quantified using a Qubit.

Optimization of Second-Strand Synthesis

A series of experiments was performed to validate the performance of the second-strand synthesis protocol relative other techniques. Species-mixing and PBMC profiling experiments were performedfor the comparison of the Seq-Well protocol with and without second-strand synthesis. For species-mixing experiments, we applied a mixture of 5,000 HEK293 and 5,000 NIH-3T3 cells to a loaded Seq-Well device, while for PBMC comparisons, a total of 10,000 PBMCs were applied to Seq-Well devices. In optimization experiments, PBMCs were thawed and immediately loaded directly onto Seq-Well devices without stimulation. Following bead removal, beads were split into separate reverse transcription reactions with and without the template-switching oligo. After reverse transcription and ExoI treatment, beads for each comparison were processed separately with and without the second-strand synthesis protocol.

A series of optimization experiments were performed to validate the effectiveness of Seq-Well S^3. Here, a series of control experiments were performed using beads from a single Seq-Well array loaded with 10,000 PBMCs. For each array the beads were split into six equal fractions and performed the following controls: (1) PCR amplification without the use of second-strand synthesis, (2) random second-strand synthesis followed by PCR amplification, (3) no template switching oligo without the use of second-strand synthesis, (4) no template switching oligo with subsequent random second-strand synthesis, (5) heat inactivation of the reverse transcription reagent without the use of second-strand synthesis, (6) heat inactivation of the reverse transcription reagent followed by random second-strand synthesis. Following PCR amplification, products were obtained from all conditions with the exception of Condition 3 (Seq-Well V1/ No TSO), which did not yield appreciable WTA product.

$CD4^+$ T Cell Comparisons of 10x Genomics, Seq-Well S^3, and Smart-Seq2

Human PBMC were thawed and rested overnight. Cells were stimulated for 18 hours by adding aCD3 (UCHT1) and aCD28 (CD28.2) antibodies to the bulk PBMC culture at a concentration of 1 mg/mL and 5 mg/mL, respectively, and $CD4^+$ T cells were enriched following stimulation using magnetic negative selection (Stemcell Technologies). Following isolation, T cells were stained with calcein violet live stain (Thermo), Sytox dead stain (Thermo), and aCD45-AF647 (HI30) antibody at 4°C for 30 minutes. After two washes, aliquots of the cells were placed on ice and sorted directly into RLT buffer using a Sony SH800S Sorter for Smart-Seq2 processing and another unstained sample for 10x Chromium analysis. Once the cells were delivered, a third aliquot was loaded onto a Seq-Well array. Single-cell libraries were generated using the Smart-Seq 2, 10x v2, and Seq-Well S^3 protocols. For comparison experiments

between 10x v3 and Seq-Well S^3 human PBMC were thawed and rested overnight. Aliquots of cells were washed, counted and placed on ice prior to processing with 10x v3 and Seq-Well S^3 protocols.

Sequencing Library Preparation

A total of 1 ng of WTA product at a concentration of 0.2 ng/μL was combined with 10 μL of Buffer TD and 5 μL of Buffer ATM and incubated at 55°C for 5 minutes. Following tagmentation, 5 μL of Buffer NT was added and incubated at room temperature for 5 minutes to neutralize the reaction. A total of 8 μL of nuclease-free water, 15 μL of buffer NPM, 1 μL of Custom P5 hybrid Oligo, and 1 μL of N700 Index oligo were combined and PCR amplification was performed using the following cycling conditions: an initial denaturation of 95°C for 30 seconds, then 12 cycles of 95°C for 10 seconds, 55°C for 30 seconds, and 72°C for 30 seconds, followed by a final extension of 72°C for 5 minutes. PCR products were isolated through two rounds of SPRI purification (0.6x and 0.8x volumetric ratios) and quantified using a Qubit. Library size distributions were determined using an Agilent Bioanalyzer D1000 High Sensitivity Screen tape.

DNA Sequencing and Alignment of PBMC Optimization samples

PBMC optimization experiments were all sequenced on NextSeq500 75 cycle kits. Sequencing read alignment was performed using version 1 of the Drop-seq pipeline (Macosko et al., 2015). NextSeq runs were loaded at a final concentration of 2.2 pM along with the custom read 1 primer using NextSeq 550 v2 sequencing kits at the Ragon Institute. Briefly, for each sequencing run, raw sequencing reads were converted from bcl files to FASTQs using bcl2fastq and demultiplexed using Nextera N700 indices that corresponded to individual samples. Demultiplexed FASTQs were then aligned using an implementation of DropSeqTools v1.0 maintained by the Broad Institute for data analysis, and aligned to the Hg19 genome using standard parameters. Individual reads were tagged with a 12-bp barcode and 8-bp unique molecular identifier (UMI) contained in Read 1 of each sequencing fragment. Following alignment, aligned read 2 sequences were grouped by the 12-bp cell barcodes and subsequently collapsed by the 8-bp UMI for digital gene expression (DGE) matrix extraction and generation.

Tissue Immunofluorescence Staining

Formalin fixed, paraffin-embedded tissue slides obtained from psoriasis patients and normal controls were heated for 30 min at 60°C, rehydrated, and epitope retrieved with Tris-EDTA, pH 6. Slides were blocked, incubated with primary antibody APOBEC3 (LS-C98892-400; Lifespan bioscience), FOSL (A03927; Boster), IL-36G (sc-80056; Santa Cruz Biotechnology), TNFAIP3 (ab74037, Abcam), IL-17RC (LS-C400522, Lifespan bioscience), and IL-17RA (LS-C359381, Lifespan bioscience) overnight at 4 °C. Slides were then washed and incubated with Donkey anti-Rabbit IgG 594, Donkey anti-Mouse IgG 488, or Donkey anti-Rat IgG 594 (all from Invitrogen) for 1 h at room temperature. Slides were washed and prepared in mounting medium with 4',6-diamidino-2-phenylindole (DAPI) (VECTASHIELD Antifade Mounting Medium with DAPI, H-1200, VECTOR). Images were acquired using Zeiss Axioskop 2 microscope and analyzed by SPOT software 5.1. Images presented are representative of at least three experiments from separate donors.

QUANTIFICATION AND STATISTICAL ANALYSIS

PBMC Comparison Experiments

Unaligned sequencing reads from 10x genomics and Seq-Well S^3 were downsampled to an average sequencing depth of 38,000 reads per cell. Specifically, downsampling was performed on Seq-Well S^3 to match the sequencing depth of 10x Genomics v2. For each data set, variable gene identification was separately performed (Seq-Well S^3, 856 variable genes and 10x Genomics v2, 516 genes). Principal component analysis was performed, and the first 20 principal components were use for t-SNE dimensionality reduction and cluster identification and discovered clusters. The proportion of cell types recovered between Seq-Well S^3 and 10x v2 was compared using a Chi-Square test.

Differences in aggregate gene detection and transcript capture were separately examined within each cell type between Seq-Well S^3 and 10x v2 using a Mann-Whitney U Test. A Lilliefors test was used to assess normality of the distribution of genes and UMIs for each technique. The linear relationship between the number of UMIs captured and aligned sequencing reads was calculated as a measure of library complexity. Specifically, the slope of the regression line between the number of UMIs against the number of aligned reads was calculated for each PBMC cell type for each technique. Library complexity was compared using a multivariable linear regression model in which the number of transcripts per cell was modeled as follows: nUMI ~ Intercept + B1*nReads + B2*Technique + B3*nReads*Technique. Statistical significance of the difference in slope (i.e. library complexity) was determined based on p values for the interaction term B3*nReads*Technique, the magnitude and significance of which correspond to a difference in slope (i.e. library complexity or the number of UMIs per aligned read). For example, in a library of low-complexity, application of additional sequencing reads might result in detection of a new transcript in every 20th aligned read (i.e. slope = 0.05). Conversely, a library of high complexity might result in detection of a new transcript with every 4 aligned reads (i.e. slope = 0.25). These comparisons were performed on libraries that have been sequenced or down-sampled to similar depths as over-sequencing can alter the relative perception of differences in library complexity. Here, libraries that have been "over-sequenced" will appear to have lower complexity because unique molecular identifiers will eventually accumulate additional reads upon saturation.

Additional comparisons were performed between Seq-Well S^3 and 10x v3 on a per cell type basis. Downsampling was performed within each cell type, for both Seq-Well S^3 and 10x v3, to the same number of aligned reads. The tagged aligned BAMs were first split by cell types, and Samtools (Li et al., 2009) was used to sort and down-sample each cell-type-specific BAM to the appropriate read depth. Picard-Tools and Drop-seq tools were used to extract down-sampled barcodes and generate expression matrices for both aligned reads and unique molecular identifier counts after separate down-sampling for each cell type within each technique.

Comparison of Gene Detection Rates

For each cell type cluster, the rate of detection for each gene was calculated as the proportion of cells with a non-zero expression value. Gene detection rates were separately calculated across $CD4^+$ T cells, B cells, $CD8^+$/NK cells, and monocytes for both Seq-Well S^3 and 10x Genomics v2. For comparisons between Seq-Well S^3 and 10x Genomics v3, gene detection rates were separately calculated within 8 cell types. For comparisons of relationship between gene-detection rates and overall expression levels, the expression level of individual genes was calculated as the average normalized expression value within each cell type for all cells identified in both Seq-Well S^3 and 10x v2 data. Statistical significance of differences in gene detection frequencies was assessed using a chi-square test using the number of cells in which a given gene had a non-zero expression values for each technique.

Sequencing and Alignment of Skin Samples

Sequencing read alignment was performed using version 2 of the Drop-seq pipeline previously described in Macosko et al. Briefly, for each Nova-Seq sequencing run, raw sequencing reads were converted from bcl files to FASTQs using bcl2fastq based on Nextera N700 indices that corresponded to individual samples. Demultiplexed FASTQs were then aligned to the Hg19 genome using STAR and the DropSeq Pipeline on a cloud-computing platform maintained by the Broad Institute. Individual reads were tagged with a 12-bp barcode and 8-bp unique molecular identifier (UMI) contained in Read 1 of each sequencing fragment. Following alignment, reads were grouped by the 12-bp cell barcodes and subsequently collapsed by the 8-bp UMI for digital gene expression (DGE) matrix extraction and generation.

Cell Quality Filtering

Cells were initially filtered on the basis of gene detection (> 500 genes per cell) and transcript detection (> 700 UMIs per cell) for inclusion in downstream analysis. Cells with fractional representation of mitochondrial genes greater than 40% were excluded. To account for potential transcript spreading, any duplicated or hamming=1 barcodes among samples sequenced on the same Nova-Seq runs were removed. For each sample, variable gene identification was separately performed and 30 principal components were calculated. Within each sample, jackstraw simulations were used to identify significant principal components that were then used to perform t-SNE dimensionality reduction and clustering for each sample using only significant principal components. Within each sample, clusters defined exclusively by mitochondrial gene expression, indicative of low-quality cells, were removed from downstream analysis.

Removal of Ambient RNA Contamination

Correction for ambient RNA contamination was performed within each sample using SoupX (Young and Behjati, 2018). Appropriate UMI thresholds for background contamination were determined using EmptyDrops (Lun et al., 2019) by calculating the likelihood that barcodes selected at UMI thresholds between 30 and 100 UMIs per barcode represent cells and selected the UMI threshold in which the distribution of likelihood most closely approximated a uniform distribution. An array-specific 'soup' profile was generated among barcodes below the UMI threshold. To calculate estimated per-cell contamination fractions, we manually selected genes observed to be bimodally expressed across cells, which suggests that these genes are predominantly expressed in a single cell type, but are observed at low-levels in other cell types for which endogenous expression would not be expected. For each array, individual transcripts were sequentially removed from each single-cell transcriptome until the probability of subsequent transcripts being soup-derived was less than 0.5 to generate a background-corrected UMI matrix for each Seq-Well S^3 array.

Doublet Removal

Doublet removal was performed for each array individually using DoubletFinder (McGinnis et al., 2019). For each array, the expected doublet rate was estimated based on the cell loading density. A total of 20,000 cells were loaded to a loaded Seq-Well device containing 85,000 wells, which resulted in an expected doublet rate of 2.37%. For each array, pseudo-doublets were generated using the following parameter values in DoubletFinder: proportion.artificial = 0.25 and proportion.NN = 0.01. Cells were identified as doublets based on their rank order in the distribution of the proportion of artificial nearest neighbors (pANN) by selecting the pANN value for the cell at the expected doublet percentile and used the corresponding pANN value as a threshold to remove additional cells with pANN greater than or equal to this value.

Analysis of Combined Skin Dataset

Variable gene identification and dimensionality reduction to identify 38 cell type clusters across 49,373 cells using Louvain clustering (resolution = 1.75). Cluster-defining genes were identified within each cluster by performing a Wilcox test in Seurat (Satija et al., 2015) and used to identify cell types. An initial round of dimensionality reduction and cluster identification was performed among cell types used in subsequent analysis (i.e. T cells, myeloid cells, B and plasma cells, endothelial cells, fibroblasts, and keratinocytes). Based on

the initial sub-clustering results for each cell type, sub-clusters defined by residual contamination not corrected for by SoupX background correction and doublet filtering were removed. In total, 11,099 cells from sub-clusters defined by residual contamination were removed: 1,471 from the T cell sub-analysis, 497 from the myeloid sub-analysis, 2,444 from the endothelial sub-analysis, 2,512 from the fibroblast sub-analysis, and 4,175 from the keratinocyte sub-analysis.

After this stringent quality control filtering step, a total of 38,274 cells were included in downstream analysis of the atlas of skin inflammation. Variable gene identification and identified 5,897 genes as variably expressed. UMAP dimensionality reduction was performed among 5,897 variably expressed and a total of 35 cell type clusters were identified using Louvain clustering (Resolution = 1.5) in Scanpy (Wolf et al., 2018). Hierarchical clustering was performed across 35 cell type clusters using a gene set composed of the top 25 cluster-defining genes from each cluster. Average gene expression values within each across the 522 unique cluster-defining genes was used to perform hierarchical clustering. A dendrogram was generated to display the similarity of clusters, and the observed relationships were used to inform rational combination of related cell type clusters for combined analysis. Cell type assignments were assigned through a combination of literature-based assessment of expression signatures and manual curation. Validation of cell type manual identification based on the combination of literature and manual curation was performed by automated cell type classification using SingleR (Aran et al., 2019). Here, 38,274 cells were classified using the blueprint encode reference dataset, and ell types assigned by SingleR were compared to the manually assigned cell type classifications.

Identification of T cell Sub-Clusters

Among the 4,943 T cells identified in the total dataset, 5,574 variable genes were identified and used to construct a force-directed graph and to perform Louvain clustering (resolution = 0.8). Cell type identities for nine T cell sub-clusters were established by examining the expression of known marker genes corresponding to $CD4^+$ T helper and $CD8^+$ T cell subsets. Further, T cell signatures were compared to previously identified signatures in SaVant (Lopez et al., 2017). To further define variation, additional sub-analyses were performed among both $CD8^+$ T cells and NK-Cytotoxic cells to generate separate UMAP dimensionality reduction using a total of 5 principal components calculated across variable genes using Seurat. For $CD8^+$ T cells, sub-clustering was performed using a resolution of 0.3, while a resolution of 0.6 was used for sub-clustering analysis for NK-Cytotoxic T cells.

T cell Receptor Detection and Clonal Expansion

The detection rates for TCR α and β (Constant, V and J genes) were examined among $CD4^+$ T cells from experiments performed on PBMCs using the Seq-Well v1, Seq-Well S^3 protocol and 10x v2. Specifically, detection of constant genes (e.g. *TRAC* and *TRBC2*) and variable genes (e.g. TRAV/TRBV) was determined by non-zero values for either genes for α and β constant genes or any TRAV/TRBV gene, respectively. Detection rates in PBMCs were calculated across multiple sequencing depths: <5,000, 5,000-25,000, 25-000-100,000, and > 100,000 aligned reads per cell.

The rate of TCR detection was identified across 2,908 T cells obtained from human skin biopsies. Conservation of V gene sequence was used as a proxy for clonal expansion among skin T cells. V gene usage for each T cell was established by identified the V gene (TRAV or TRBV) with the highest expression level. The distribution of TRAV and TRBV genes within each sample to identify potential clonal expansions. The gini coefficient and the Shannon divergence were calculated for TRAV and TRBV sequences within each sample to identify over-represented TRAV/TRBV sequences.

Identification of Myeloid Heterogeneity

Sub-analysis was performed among myeloid populations (i.e. dendritic cells, macrophages, mast cells, and Langerhans cells) identified in global analysis of 38,274 total cells. Using a combined dataset of 5,010 myeloid cells, variable gene identification and dimensionality reduction was performed in Scanpy. A force-directed graph was constructed across 6,599 variable genes and Louvain clustering (resolution = 0.80) was performed, and we obtained 10 sub-clusters of myeloid cells.

To understand differences in Langerhans cells in normal skin, differential expression analysis was performed within each cluster of Langerhans cells. Differential expression was performed between Langerhans cells from Myeloid cluster 8 between normal and leprosy skin biopsies. Gene-set enrichment analysis was performed among genes upregulated in Langerhan's cells from leprosy samples in comparison to signatures contained in the MSigDb database.

UMAP dimensionality reduction and Louvain clustering (resolution = 0.45) were performed among 951 dendritic cells. Cluster-defining genes were identified within each of 5 sub-groupings of dendritic cells by performing a Wilcox test in Seurat. Comparisons to published signatures of dendritic cell phenotypes were performed to understand how dendritic cells related to previous findings (Dutertre et al., 2019; Villani et al., 2017). Specifically, expression scores were generated using the top 10 genes using the AddModuleScore function in Seurat. Significance of cluster enrichment was determined by performing 1,000 permutations in which cell and signature score identifiers were randomly re-assigned.

Identification of Endothelial Heterogeneity

Sub-analysis was performed across 8,571 endothelial cells identified in the global analysis to generate a force-directed graph and Louvain clustering (resolution = 0.6) using 5,082 variable genes. Genes enriched in each of 9 endothelial sub-cluster were identified using a Wilcox test in Seurat. For each addressin gene examined, the distribution of cells with non-zero expression was examined in each of the endothelial cells.

Identification of Fibroblast Heterogeneity

Sub-analysis was performed across 7,237 fibroblasts identified in global analysis. UMAP dimensionality reduction using 4,825 variable genes and Louvain clustering (resolution = 0.6) were performed. Enrichment analysis was performed using a Wilcox test to identify cluster-defining genes for each of 10 fibroblasts sub-clusters. For each fibroblast sub-cluster, the fractional composition of cells from each sample and condition was examined.

Enrichment of Immune and Stromal Populations by Condition

To understand enrichment of specific cell types in a given condition, the proportional composition of a given cell subset was calculated with each skin biopsy. For example, the proportion of T cell subsets obtained from each of 19 skin biopsies was determined as the number of cells of a given sub-type divided by the total number of T cells obtained from that biopsy. To assess significance of enrichment within a given inflammatory skin condition, comparisons were performed in two ways: (1) a Mann-Whitney U test was used to examine differences in the proportion of individual cell subsets between biopsies of a given condition and all other samples; and (2) a Mann-Whitney U test was performed between proportions from biopsies of a given condition and those obtained from normal skin. Specifically, we calculated enrichments for those conditions for which at least 2 biopsies were obtained, i.e. acne (n-4), leprosy (n=4), granuloma annulare (n=2), and psoriasis (n=5). No comparisons of enrichment were performed for the single alopecia areata biopsy. We performed tests of enrichment within each of the following subsets: T cells, myeloid cells, dendritic cells, fibroblasts and endothelial cells.

Pseudo-temporal Reconstruction of Epidermal Keratinocytes

Diffusion analysis was performed across all keratinocytes and hair follicle cells using the Diffmap function in Scanpy (Wolf et al., 2018), which implements a method for diffusion pseudotime reconstruction (Haghverdi et al., 2015). Pseudo-temporal analysis was performed within normal keratinocyte separately, using the basal keratinocyte population as the origin of the pseudo-temporal ordering. Differential expression analysis between normal and psoriatic keratinocytes was performed in Seurat using a Wilcox rank-sum test across all keratinocytes and among basal, differentiating, and terminal keratinocytes.

Differentiation trajectories were constructed for keratinocytes from normal and psoriatic skin biopsies using Scorpius as implemented in dyno (Saelens et al., 2019). Correlation of gene expression patterns with pseudo-temporal order were examined separately for keratinocyte populations from each sample. Here, linear regression was performed between pseudo-time values and gene expression values for normal and psoriatic keratinocytes. Differential pseudo-time correlation analysis was performed between normal and psoriatic keratinocytes for 2 normal skin samples and 5 psoriasis samples. The difference in average pseudo-time correlation between psoriatic and normal keratinocytes was calculated to identify genes that are uniquely involved in the development of psoriatic keratinocytes. Reference immuohistochemical staining for KRT14 and FLG was obtained from the Human Protein Atlas (KRT14: https://www.proteinatlas.org/ENSG00000186847-KRT14/tissue/skin+1#img and https://www.proteinatlas.org/ENSG00000143631-FLG/tissue/skin+2#img).

Keratinocyte Cytokine-Response Profiles

Among both normal and psoriatic keratinocytes, cytokine response scores were generated using a series of reference datasets that were previously generated from *in vitro* experiments in which cultured keratinocytes were stimulated with cytokines, individually or in combination (IL-17A, IL17-A + TNF-α, TNF-α, IFN-α, IL-4, IL-13, and IFN-γ). Expression signature were generated relative an unstimulated control population of keratinocytes. For each cytokine condition, the top 100 differentially expressed genes were used to generate a cytokine response score across both psoriatic and normal keratinocytes in Seurat. The distribution of cytokine response scores were examined across basal, differentiating, and terminal keratinocytes between normal and psoriatic keratinocytes.

ADDITIONAL RESOURCES

An extensive protocol is included as part of the supplementary materials along with an itemized cost-model for Seq-Well S^3. These are also available on Shalek lab website: http://shaleklab.com/resource/seq-well/.

Immunity, Volume *53*

Supplemental Information

Second-Strand Synthesis-Based Massively Parallel scRNA-Seq Reveals Cellular States and Molecular Features of Human Inflammatory Skin Pathologies

Travis K. Hughes, Marc H. Wadsworth II, Todd M. Gierahn, Tran Do, David Weiss, Priscila R. Andrade, Feiyang Ma, Bruno J. de Andrade Silva, Shuai Shao, Lam C. Tsoi, Jose Ordovas-Montanes, Johann E. Gudjonsson, Robert L. Modlin, J. Christopher Love, and Alex K. Shalek

Figure S1.

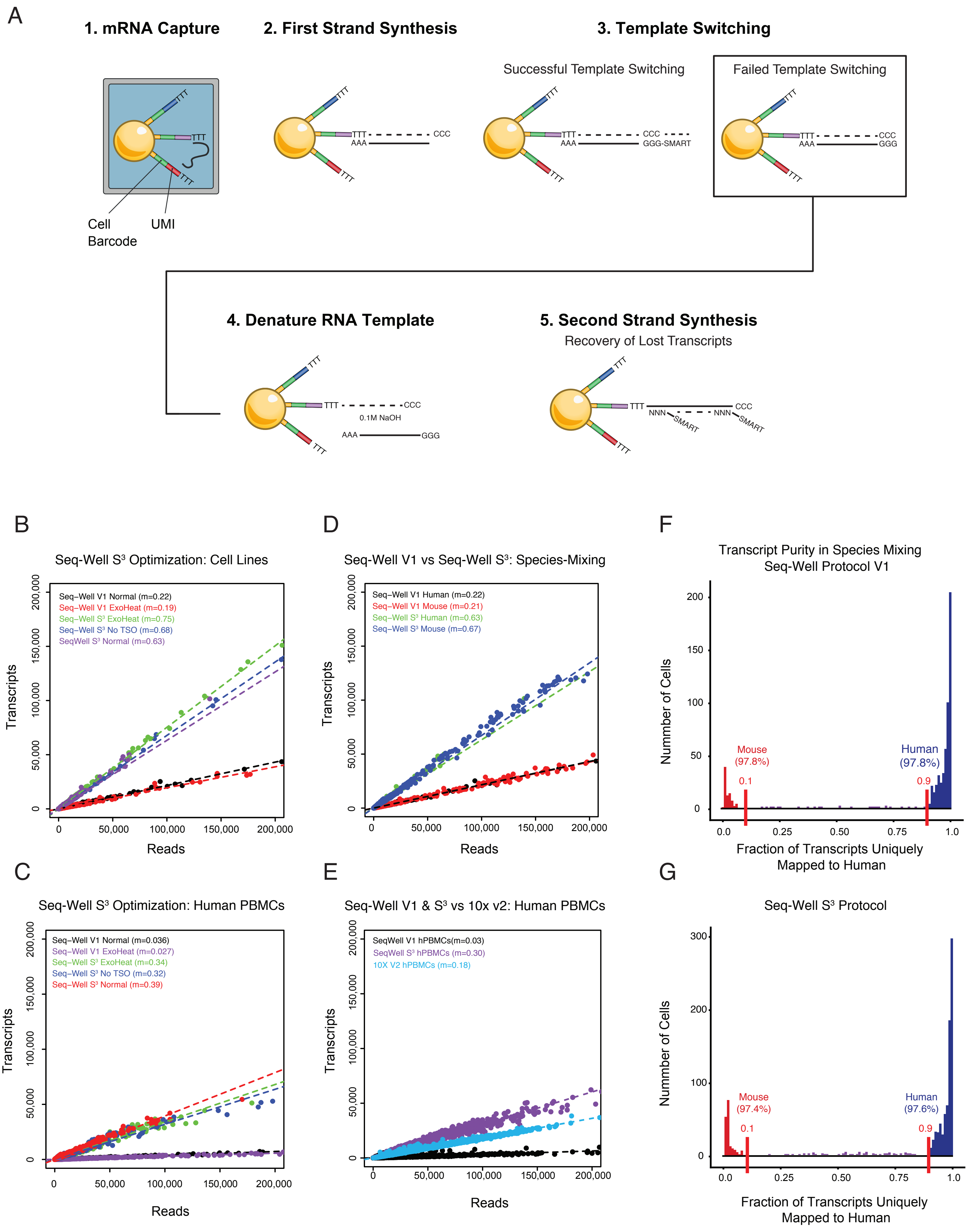

A
1. mRNA Capture
2. First Strand Synthesis
3. Template Switching
Successful Template Switching
Failed Template Switching
TTT
AAA
CCC
GGG-SMART
GGG
Cell Barcode
UMI
4. Denature RNA Template
0.1M NaOH
5. Second Strand Synthesis
Recovery of Lost Transcripts
NNN
SMART
B
Seq-Well S³ Optimization: Cell Lines
Seq–Well V1 Normal (m=0.22)
Seq–Well V1 ExoHeat (m=0.19)
Seq–Well S³ ExoHeat (m=0.75)
Seq–Well S³ No TSO (m=0.68)
SeqWell S³ Normal (m=0.63)
Transcripts
Reads
D
Seq-Well V1 vs Seq-Well S³: Species-Mixing
Seq–Well V1 Human (m=0.22)
Seq–Well V1 Mouse (m=0.21)
Seq–Well S³ Human (m=0.63)
Seq–Well S³ Mouse (m=0.67)
F
Transcript Purity in Species Mixing
Seq-Well Protocol V1
Nummber of Cells
Mouse (97.8%)
Human (97.8%)
0.1
0.9
Fraction of Transcripts Uniquely Mapped to Human
C
Seq-Well S³ Optimization: Human PBMCs
Seq–Well V1 Normal (m=0.036)
Seq–Well V1 ExoHeat (m=0.027)
Seq–Well S³ ExoHeat (m=0.34)
Seq–Well S³ No TSO (m=0.32)
Seq–Well S³ Normal (m=0.39)
E
Seq-Well V1 & S³ vs 10x v2: Human PBMCs
SeqWell V1 hPBMCs(m=0.03)
SeqWell S³ hPBMCs (m=0.30)
10X V2 hPBMCs (m=0.18)
G
Seq-Well S³ Protocol
Mouse (97.4%)
Human (97.6%)

Figure S1. Second-Strand Synthesis Overview, related to Figure 1

A. Illustration of the second strand synthesis procedure: (1) mRNA is captured via poly-T priming of poly-adenylated mRNA; (2) First strand synthesis is performed to generate single-stranded cDNA template on bead-bound sequences; (3) Successful template switching: The use of enzymes with terminal transferase activity generates a 3' overhang of 3 cytosines. Template switching utilizes this overhang to append the SMART sequence to both ends of the cDNA molecule during first strand synthesis. Failed Template Switching: If template switching fails, this results in loss of previously primed and reverse transcribed mRNA molecules; (4) mRNA template is chemically denatured using 0.1M NaOH; (5) Second strand synthesis is performed using a random-octamer with the SMART sequence in the 5' orientation; and, (6) Following second strand synthesis, PCR amplification, library preparation and sequencing are performed to generate data.

B. Scatterplots show the relationship between transcript detection (y-axis) and number of aligned reads per cell (x-axis) for a series of optimization experiments using HEK293 and NIH-3T3 cell lines.

C. Scatterplots show the relationship between transcript detection (y-axis) and the number of aligned reads per cell (x-axis) for a series of optimization experiments using PBMCs.

D. Scatterplots that illustrate the relationship between number of transcripts detected (y-axis) and number of aligned reads per cell (x-axis) between Seq-Well V1 and Seq-Well S^3 in species mixing experiments using HEK293 and NIH-3T3 cells.

E. Scatterplots that illustrate the relationship between number of transcripts detected (y-axis) and number of aligned reads per cell (x-axis) between Seq-Well V1 and Seq-Well S^3 in a series of optimization experiment using human PBMCs.

F. Histograms that show the fraction of transcripts uniquely mapped to the human genome for each cell for Seq-Well V1. Colors indicate species classification for cells with at least 90% purity of human (blue) or mouse (red) mapping.

G. Histograms that show the fraction of transcripts uniquely mapped to the human genome for each cell for Seq-Well S^3. Colors indicate species classification for cells with at least 90% purity of human (blue) or mouse (red) mapping.

Figure S2.

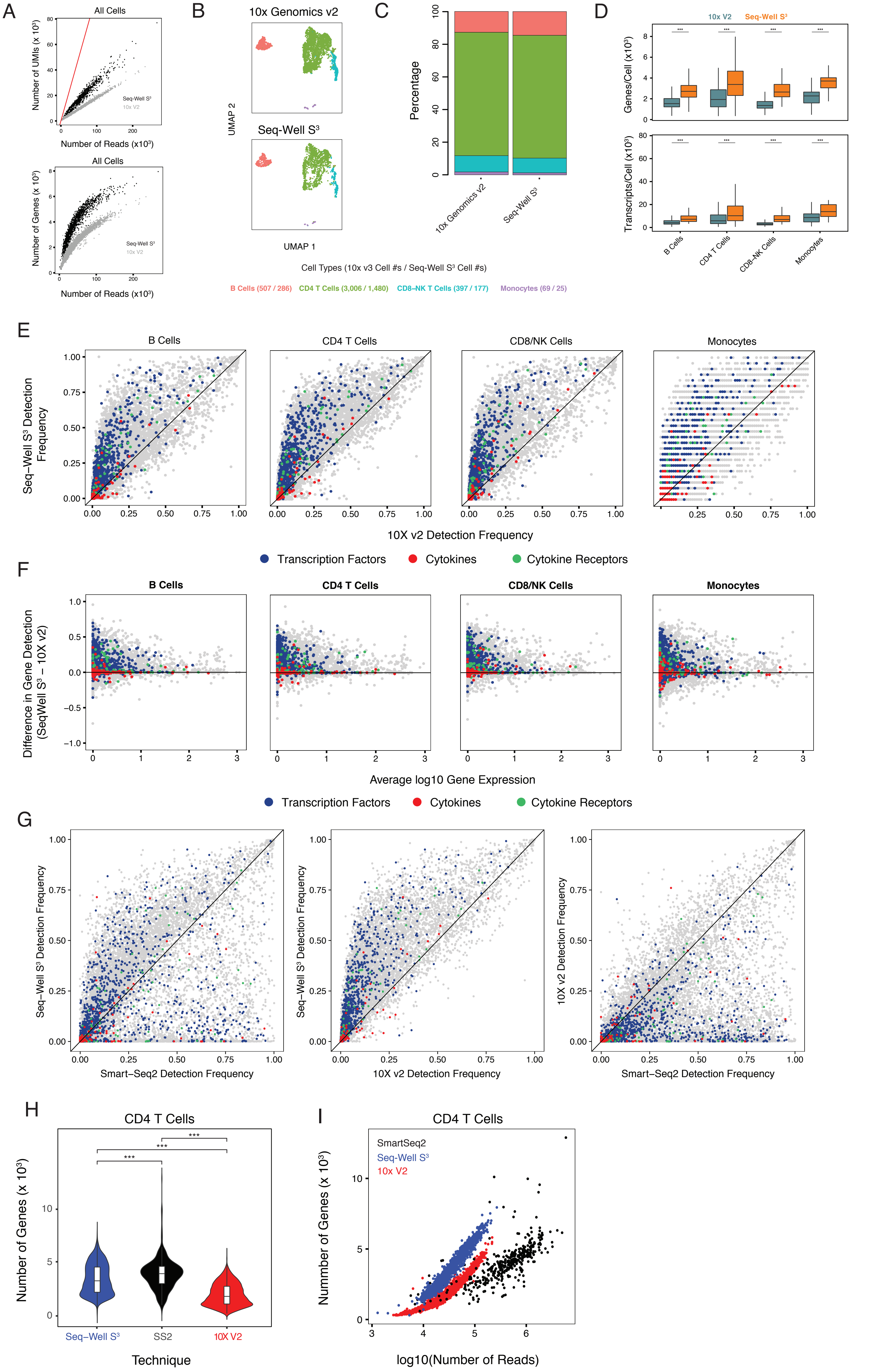
A
All Cells
Number of UMIs (x 10³)
Number of Reads (x10³)
Seq-Well S³
10x V2
All Cells
Number of Genes (x 10³)
Number of Reads (x10³)
B
10x Genomics v2
Seq-Well S³
UMAP 2
UMAP 1
C
Percentage
10x Genomics v2
Seq-Well S³
D
10x V2
Seq-Well S³
Genes/Cell (x10³)
Transcripts/Cell (x10³)
B Cells
CD4 T Cells
CD8-NK Cells
Monocytes
Cell Types (10x v3 Cell #s / Seq-Well S³ Cell #s)
B Cells (507 / 286)
CD4 T Cells (3,006 / 1,480)
CD8–NK T Cells (397 / 177)
Monocytes (69 / 25)
E
B Cells
CD4 T Cells
CD8/NK Cells
Monocytes
Seq–Well S³ Detection Frequency
10X v2 Detection Frequency
Transcription Factors
Cytokines
Cytokine Receptors
F
B Cells
CD4 T Cells
CD8/NK Cells
Monocytes
Difference in Gene Detection (SeqWell S³ – 10X v2)
Average log10 Gene Expression
Transcription Factors
Cytokines
Cytokine Receptors
G
Seq–Well S³ Detection Frequency
Smart–Seq2 Detection Frequency
Seq–Well S³ Detection Frequency
10X v2 Detection Frequency
10X v2 Detection Frequency
Smart–Seq2 Detection Frequency
H
CD4 T Cells
Number of Genes (x 10³)
Seq–Well S³
SS2
10X V2
Technique
I
CD4 T Cells
SmartSeq2
Seq-Well S³
10x V2
Nummber of Genes (x 10³)
log10(Number of Reads)

Figure S2. PBMC Methods Comparisons, related to Figure 1

A. Top: Scatterplot showing differences in per-cell transcript capture (y-axis) as a function of aligned reads per cell (x-axis) between 10x Genomics v2 (10x v3, grey) and Seq-Well S^3 (black). Red line indicates uniform line where transcripts per cell and aligned reads would be equivalent. Bottom: Scatterplot shows the differences in per-cell gene detection (y-axis) as a function of aligned reads per cell (x-axis) between 10x v2 (grey) and Seq-Well S^3 (black).

B. UMAP plots showing detected cell-types among PBMCs using 10X v2 (left) and Seq-Well S^3 (right).

C. Stacked barplots show the proportion of cell types recovered using Seq-Well S^3 (left) and 10X v2 (right).

D. Top: Boxplots (median +- quartiles) showing the distribution of per cell gene detection from 10X v2 (left) and Seq-Well S^3 (right). Bottom: Boxplots (median +/- quartiles) showing the distribution of per cell- transcript capture from 10X v2 (left) and Seq-Well S^3 (right).

E. Scatterplots showing a comparison of gene detection frequencies between Seq-Well S^3 (y-axis) and 10x v2 (x-axis) for each cell type.

F. Scatterplots showing the difference in gene detection between Seq-Well S^3 and 10X v2 (y-axis) as a function of average normalized expression (log(scaled UMI + 1)) (x-axis).

G. Scatterplots showing a comparison of gene detection frequencies among sorted $CD4^+$ T cells between **(Left)** Seq-well S^3 (y-axis) and 10x v2 (x-axis), (**Middle)** Seq-Well S^3 (y-axis) and Smart-Seq2 (x-axis), and **(Right)** 10x v2 (y-axis) and Smart-Seq2 (x-axis).

H. Violin plots (boxplots median +- quartiles) showing the distribution of per-cell transcript capture for Seq-well S^3 (blue; n = 1,485), 10x v2 (red; n = 2995), and Smart-Seq2 (black, n = 382). *** P-values < 1.0 $x10^{-10}$.

I. Scatterplot showing the relationship between aligned reads and genes detected per cell between Seq-Well S^3 (blue), 10x v2 (red) and Smart-Seq2 (black) in sorted PBMC $CD4^+$ T cells.

Figure S3.

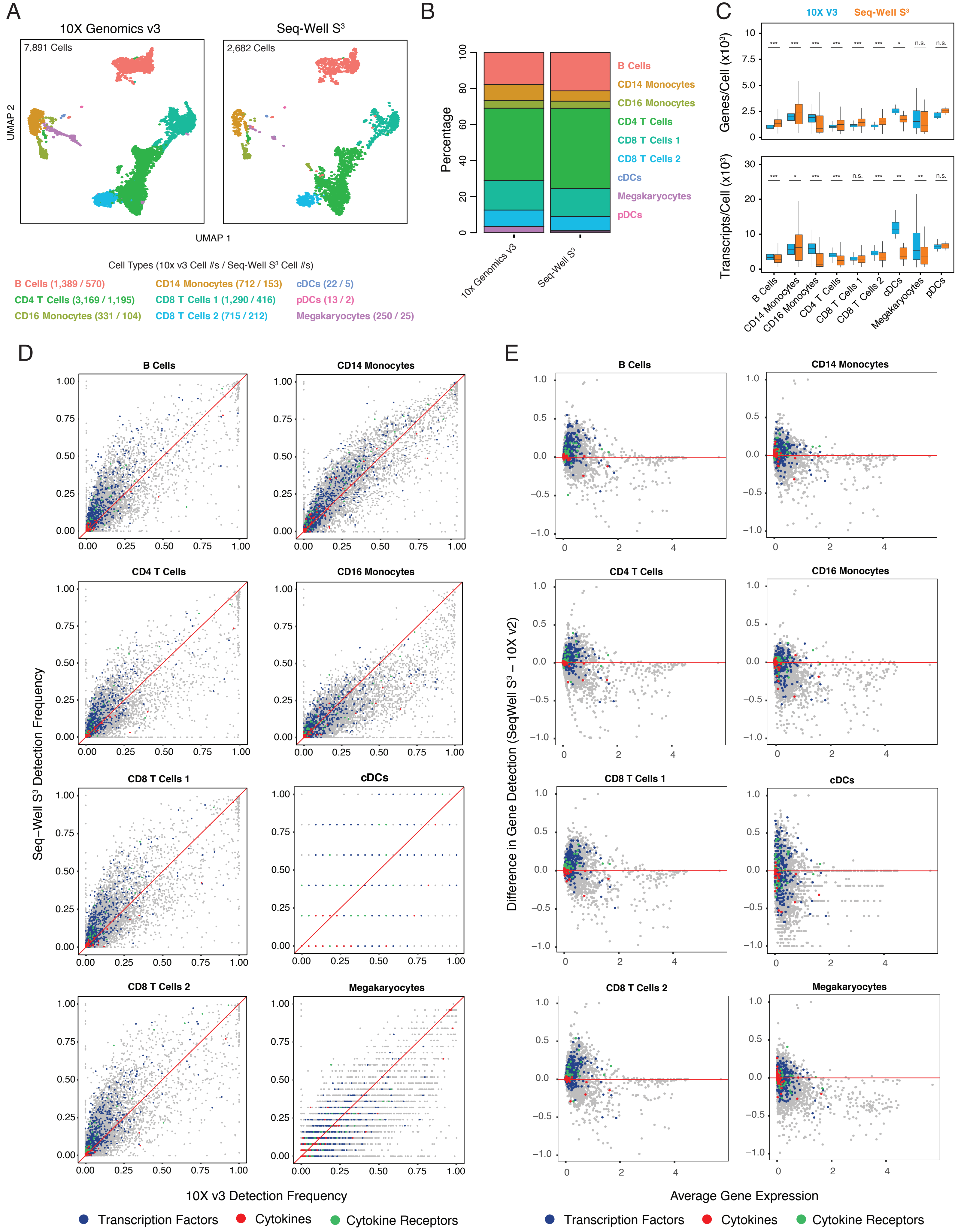
A
10X Genomics v3
Seq-Well S³
7,891 Cells
2,682 Cells
UMAP 2
UMAP 1
Cell Types (10x v3 Cell #s / Seq-Well S³ Cell #s)
B Cells (1,389 / 570)
CD4 T Cells (3,169 / 1,195)
CD16 Monocytes (331 / 104)
CD14 Monocytes (712 / 153)
CD8 T Cells 1 (1,290 / 416)
CD8 T Cells 2 (715 / 212)
cDCs (22 / 5)
pDCs (13 / 2)
Megakaryocytes (250 / 25)
B
Percentage
10x Genomics v3
Seq-Well S³
B Cells
CD14 Monocytes
CD16 Monocytes
CD4 T Cells
CD8 T Cells 1
CD8 T Cells 2
cDCs
Megakaryocytes
pDCs
C
10X V3
Seq-Well S³
Genes/Cell (x10³)
Transcripts/Cell (x10³)
n.s.
B Cells
CD14 Monocytes
CD16 Monocytes
CD4 T Cells
CD8 T Cells 1
CD8 T Cells 2
cDCs
Megakaryocytes
pDCs
D
B Cells
CD14 Monocytes
CD4 T Cells
CD16 Monocytes
CD8 T Cells 1
cDCs
CD8 T Cells 2
Megakaryocytes
Seq-Well S³ Detection Frequency
10X v3 Detection Frequency
Transcription Factors
Cytokines
Cytokine Receptors
E
B Cells
CD14 Monocytes
CD4 T Cells
CD16 Monocytes
CD8 T Cells 1
cDCs
CD8 T Cells 2
Megakaryocytes
Difference in Gene Detection (SeqWell S³ – 10X v2)
Average Gene Expression
Transcription Factors
Cytokines
Cytokine Receptors

Figure S3. Seq-Well S^3 vs 10x v3 3' PBMC Methods Comparisons, related to Figure 1

A. UMAP plots for 7,891 PBMCs from 10x Genomics v3 (left) and 2,682 PBMCs from Seq-Well S^3 (right).

B. Stacked barplots showing the distribution of recovered cell types between 10x Genomics v3 and Seq-Well S^3.

C. Top: Boxplots (median +- quartiles) showing the distribution of per cell gene detection from 10X v3 (left) and Seq-Well S^3 (right). Bottom: Boxplots (median +/- quartiles) showing the distribution of per cell-gene detection from 10X v3 (left) and Seq-Well S^3 (right).

D. Scatterplots showing a comparison of gene detection frequencies between Seq-Well S^3 (y-axis) and 10x v3 (x-axis) for each cell type.

E. Scatterplots showing the difference in gene detection between Seq-Well S^3 and 10X v3 (y-axis) as a function of average expression (counts) (x-axis).

Figure S4.

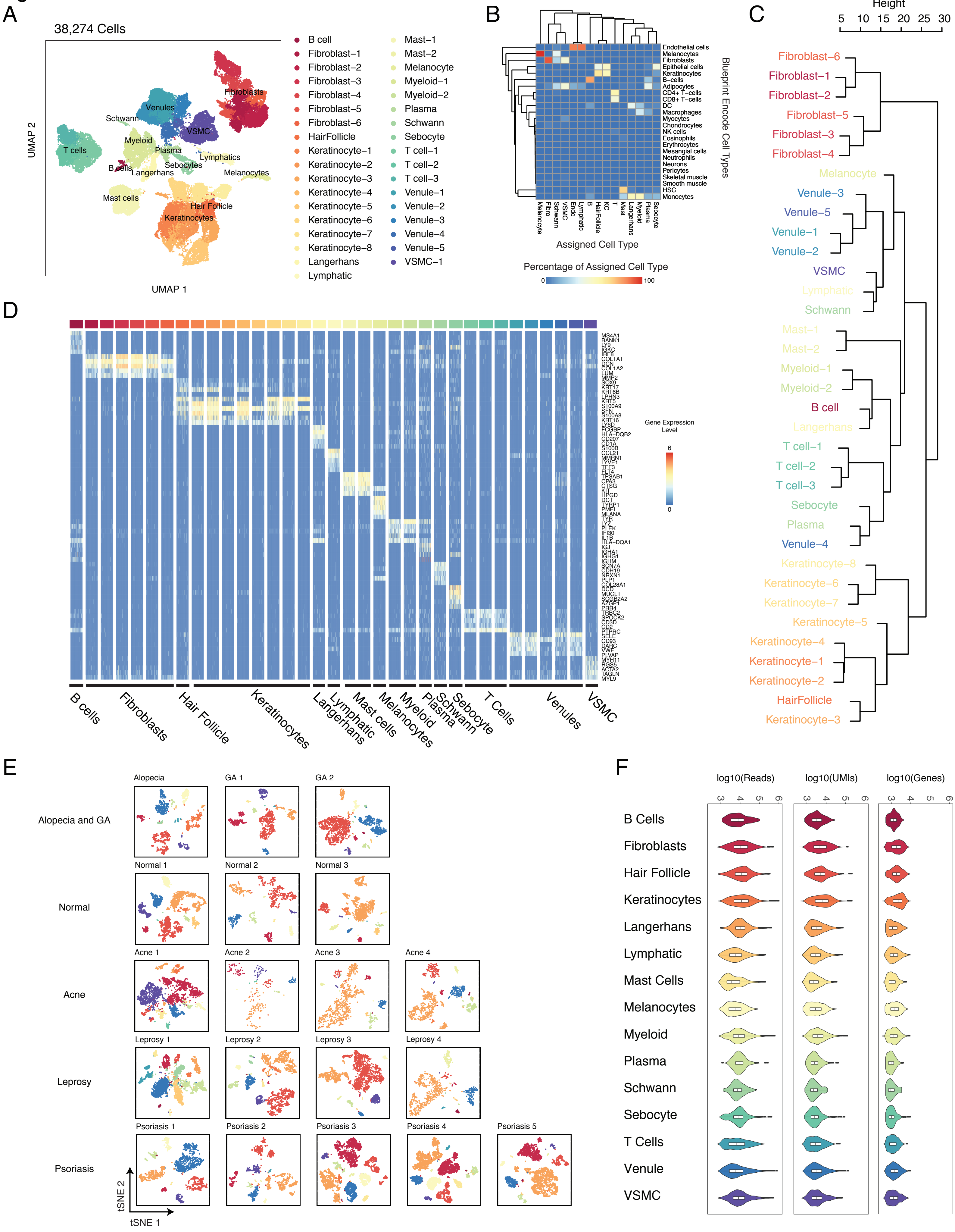

A
38,274 Cells
UMAP 2
UMAP 1
Fibroblasts
Venules
Schwann
VSMC
Myeloid
T cells
Plasma
Lymphatics
B cells
Sebocytes
Langerhans
Melanocytes
Mast cells
Hair Follicle
Keratinocytes
B cell
Fibroblast−1
Fibroblast−2
Fibroblast−3
Fibroblast−4
Fibroblast−5
Fibroblast−6
HairFollicle
Keratinocyte−1
Keratinocyte−2
Keratinocyte−3
Keratinocyte−4
Keratinocyte−5
Keratinocyte−6
Keratinocyte−7
Keratinocyte−8
Langerhans
Lymphatic
Mast−1
Mast−2
Melanocyte
Myeloid−1
Myeloid−2
Plasma
Schwann
Sebocyte
T cell−1
T cell−2
T cell−3
Venule−1
Venule−2
Venule−3
Venule−4
Venule−5
VSMC−1
B
Endothelial cells
Melanocytes
Fibroblasts
Epithelial cells
Keratinocytes
B−cells
Adipocytes
CD4+ T−cells
CD8+ T−cells
DC
Macrophages
Myocytes
Chondrocytes
NK cells
Eosinophils
Erythrocytes
Mesangial cells
Neutrophils
Neurons
Pericytes
Skeletal muscle
Smooth muscle
HSC
Monocytes
Blueprint Encode Cell Types
Melanocyte
Fibro
Schwann
VSMC
Endo
Lymphatic
B
HairFollicle
KC
T
Mast
Langerhans
Myeloid
Plasma
Sebocyte
Assigned Cell Type
Percentage of Assigned Cell Type
0
100
C
Height
5 10 15 20 25 30
Fibroblast−6
Fibroblast−1
Fibroblast−2
Fibroblast−5
Fibroblast−3
Fibroblast−4
Melanocyte
Venule−3
Venule−5
Venule−1
Venule−2
VSMC
Lymphatic
Schwann
Mast−1
Mast−2
Myeloid−1
Myeloid−2
B cell
Langerhans
T cell−1
T cell−2
T cell−3
Sebocyte
Plasma
Venule−4
Keratinocyte−8
Keratinocyte−6
Keratinocyte−7
Keratinocyte−5
Keratinocyte−4
Keratinocyte−1
Keratinocyte−2
HairFollicle
Keratinocyte−3
D
Gene Expression Level
6
0
B cells
Fibroblasts
Hair Follicle
Keratinocytes
Langerhans
Lymphatic
Mast cells
Melanocytes
Myeloid
Plasma
Schwann
Sebocyte
T Cells
Venules
VSMC
E
Alopecia
GA 1
GA 2
Alopecia and GA
Normal 1
Normal 2
Normal 3
Normal
Acne 1
Acne 2
Acne 3
Acne 4
Acne
Leprosy 1
Leprosy 2
Leprosy 3
Leprosy 4
Leprosy
Psoriasis 1
Psoriasis 2
Psoriasis 3
Psoriasis 4
Psoriasis 5
Psoriasis
tSNE 2
tSNE 1
F
log10(Reads)
log10(UMIs)
log10(Genes)
3 4 5 6
B Cells
Fibroblasts
Hair Follicle
Keratinocytes
Langerhans
Lymphatic
Mast Cells
Melanocytes
Myeloid
Plasma
Schwann
Sebocyte
T Cells
Venule
VSMC

Figure S4. Overview of Samples, related to Figure 2

A. UMAP plot for 38,274 cells colored by 35 cell type clusters.

B. Comparison of cell-type classification to results obtained using SingleR. Color corresponds to the percentage of manually classified cells types (columns) assigned to cell-type references contained in the Blueprint Encode dataset (rows).

C. Dendrogram of hierarchical clustering shows similarity of cell type clusters among top 25 cluster-defining genes.

D. Heatmap showing the relative expression of cell-type defining gene signatures across 38,274 cells (**Table S4**).

E. t-SNE plots for each of the nineteen skin biopsies colored by generic cell type.

F. Violin plots show the distribution of per-cell quality metrics displayed in UMAP embedding of 38,274 cells colored by colored generic cell-type classification.

Figure S5.

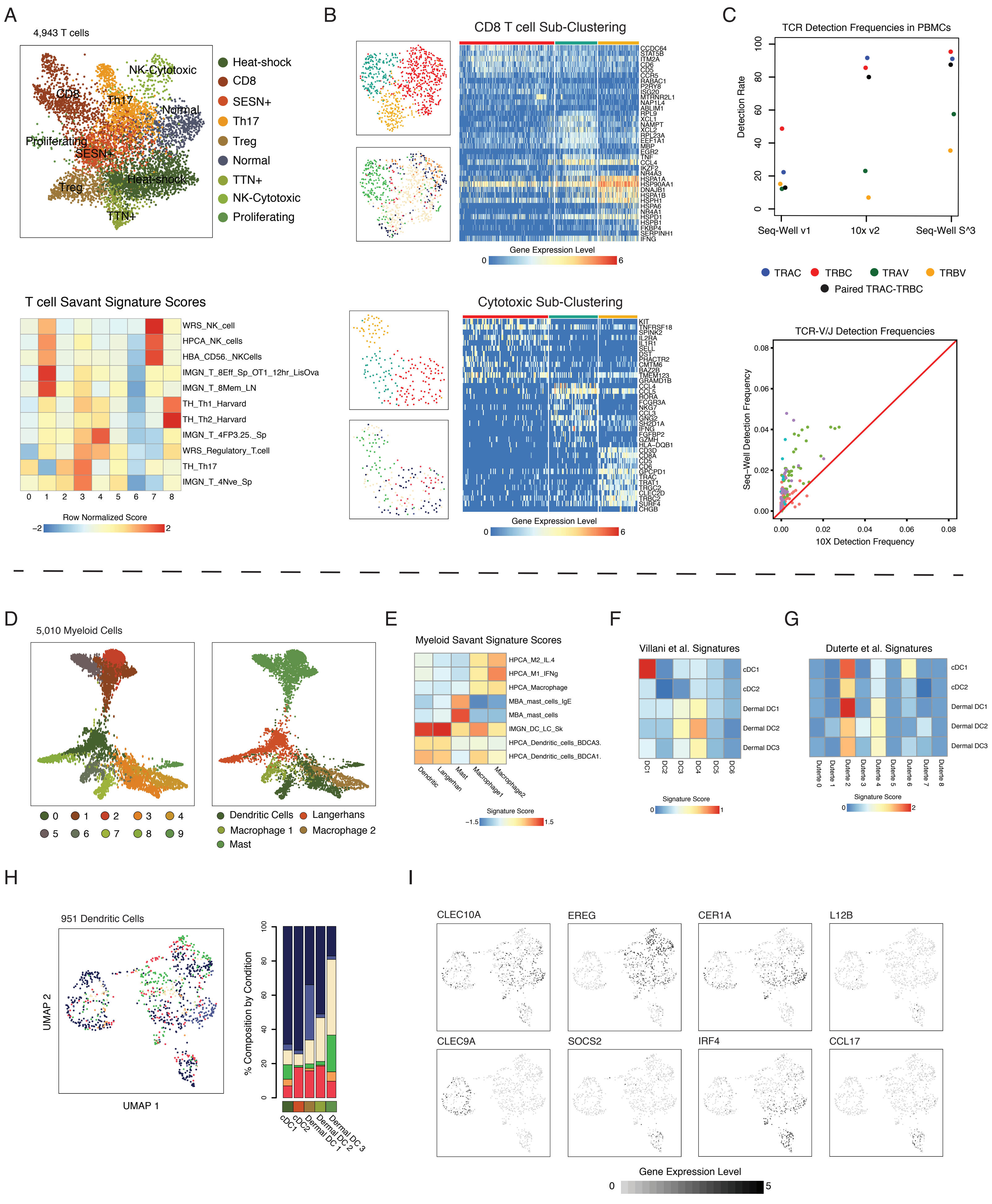
A
4,943 T cells
NK-Cytotoxic
CD8
Th17
Normal
Proliferating
SESN+
Heat-shock
Treg
TTN+
Heat-shock
CD8
SESN+
Th17
Treg
Normal
TTN+
NK-Cytotoxic
Proliferating
B
CD8 T cell Sub-Clustering
CCDC64
STAT5B
ITM2A
CD6
CD5
CCR5
RABAC1
P2RY8
ISG20
MTRNR2L1
NAP1L4
ABLIM1
RPL9
XCL1
NAMPT
XCL2
RPL23A
EEF1A1
MBP
EGR2
TNF
CCL4
IKZF2
NR4A3
HSPA1A
HSP90AA1
DNAJB1
HSPA1B
HSPH1
HSPA6
NR4A1
HSPD1
HSPB1
FKBP4
SERPINH1
IFNG
Gene Expression Level
0
6
C
TCR Detection Frequencies in PBMCs
Detection Rate
0
20
40
60
80
100
Seq-Well v1
10x v2
Seq-Well S^3
TRAC
TRBC
TRAV
TRBV
Paired TRAC-TRBC
T cell Savant Signature Scores
WRS_NK_cell
HPCA_NK_cells
HBA_CD56._NKCells
IMGN_T_8Eff_Sp_OT1_12hr_LisOva
IMGN_T_8Mem_LN
TH_Th1_Harvard
TH_Th2_Harvard
IMGN_T_4FP3.25._Sp
WRS_Regulatory_T.cell
TH_Th17
IMGN_T_4Nve_Sp
0 1 2 3 4 5 6 7 8
Row Normalized Score
−2
2
Cytotoxic Sub-Clustering
KIT
TNFRSF18
SPINK2
IL2RA
IL1R1
SELL
DST
PHACTR2
CMTM6
BAZ2B
TMEM123
GRAMD1B
CCL4
CCL5
RORA
FCGR3A
NKG7
CCL3
GNG2
SH2D1A
IFNG
FGFBP2
GZMH
HLA−DQB1
CD3D
CD8A
CD5
CD6
GPCPD1
TRAC
TRAT1
TRGC2
CLEC2D
TRBC2
SURF4
CHGB
Gene Expression Level
0
6
TCR-V/J Detection Frequencies
Seq−Well Detection Frequency
10X Detection Frequency
0.00
0.02
0.04
0.06
0.08
D
5,010 Myeloid Cells
0 1 2 3 4
5 6 7 8 9
Dendritic Cells
Langerhans
Macrophage 1
Macrophage 2
Mast
E
Myeloid Savant Signature Scores
HPCA_M2_IL.4
HPCA_M1_IFNg
HPCA_Macrophage
MBA_mast_cells_IgE
MBA_mast_cells
IMGN_DC_LC_Sk
HPCA_Dendritic_cells_BDCA3.
HPCA_Dendritic_cells_BDCA1.
Dendritic
Langerhan
Mast
Macrophage1
Macrophage2
Signature Score
−1.5
1.5
F
Villani et al. Signatures
cDC1
cDC2
Dermal DC1
Dermal DC2
Dermal DC3
DC1
DC2
DC3
DC4
DC5
DC6
Signature Score
0
1
G
Duterte et al. Signatures
cDC1
cDC2
Dermal DC1
Dermal DC2
Dermal DC3
Duterte 0
Duterte 1
Duterte 2
Duterte 3
Duterte 4
Duterte 5
Duterte 6
Duterte 7
Duterte 8
Signature Score
0
2
H
951 Dendritic Cells
UMAP 2
UMAP 1
% Composition by Condition
0
20
40
60
80
100
cDC1
cDC2
Dermal DC 1
Dermal DC 2
Dermal DC 3
I
CLEC10A
EREG
CER1A
L12B
CLEC9A
SOCS2
IRF4
CCL17
Gene Expression Level
0
5

Figure S5. Immune Cell Heterogeneity, related to Figures 3 and 4

A. (Top) Force-directed graph of 4,943 T cells colored by T cell sub-cluster. (Bottom) Heatmap of gene-set enrichment scores based on comparison of T cell phenotypic sub-clusters to a curated list of reference signatures in the Savant database.

B. Sub-grouping results for CD8 T cells (top) (**Table S7**) and cytotoxic cells (bottom) (**Table S8**). For each analysis, t-SNE plots colored by inflammatory skin condition (top-left) and sub-cluster (bottom-left) are shown. For each clusters, heatmaps show gene expression patterns across T and NK cells sub-types (right).

C. (Top) Detection rates for TCR genes for PBMCs in Seq-Well v1, 10x v2. and Seq-Well S^3. (Bottom) Detection frequency of TCR V-J (e.g. TRAV/J and TRBV/J) genes in $CD4^+$ T cells from peripheral blood between Seq-Well S^3 (y-axis) and 10x v2 (x-axis). Colors correspond to TRAJ (red), TRAV (green), TRBJ (blue), and TRBV (purple) genes.

D. (Left) Force-directed graph of 5,010 myeloid cells colored by myeloid sub-clusters (Louvain resolution = 0.6). (Right) Force-directed graph of 5,010 myeloid cells colored by myeloid phenotypes.

E. Heatmap of gene-set enrichment scores based on comparison of myeloid phenotypic sub-clusters to a curated list of reference signatures in the Savant database.

F. Heatmap showing average signature score across 5 dermal DC populations based on dendritic cell signatures from *Villani et al. Science 2017*.

G. Heatmap showing average signature score across 5 dermal DC populations based on signatures from et al. Immunity 2019.

H. (Left) UMAP plot for 951 dendritic cells from human skin colored by inflammatory skin condition. (Right) Stacked barplot showing composition of dendritic cells within each of nine skin biopsies by DC sub-cluster.

I. UMAP plots colored by normalized expression levels for DC sub-grouping-defining genes.

Figure S6.

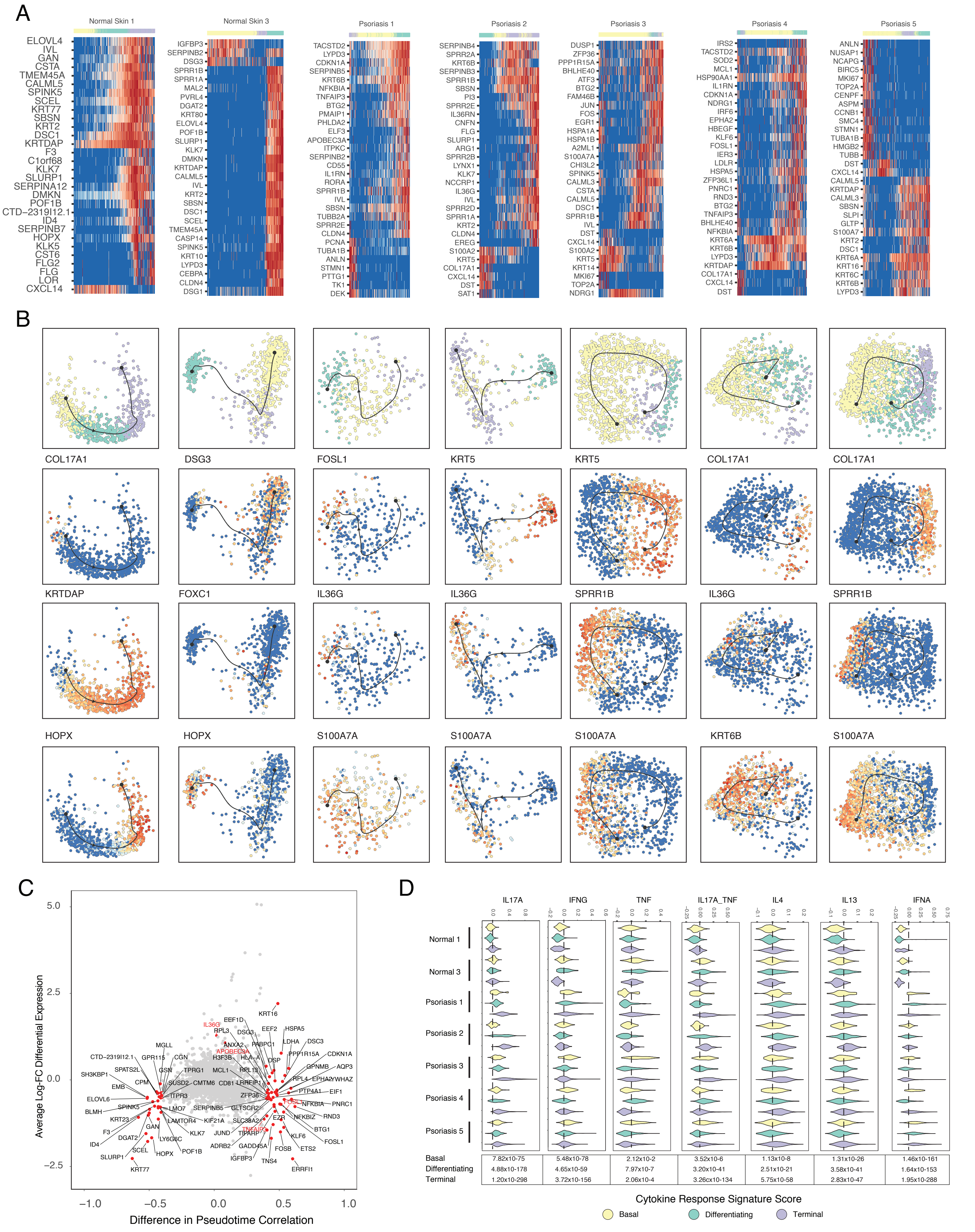
A
Normal Skin 1
Normal Skin 3
Psoriasis 1
Psoriasis 2
Psoriasis 3
Psoriasis 4
Psoriasis 5
B
COL17A1
DSG3
FOSL1
KRT5
KRT5
COL17A1
COL17A1
KRTDAP
FOXC1
IL36G
IL36G
SPRR1B
IL36G
SPRR1B
HOPX
HOPX
S100A7A
S100A7A
S100A7A
KRT6B
S100A7A
C
Average Log-FC Differential Expression
Difference in Pseudotime Correlation
D
IL17A
IFNG
TNF
IL17A_TNF
IL4
IL13
IFNA
Normal 1
Normal 3
Psoriasis 1
Psoriasis 2
Psoriasis 3
Psoriasis 4
Psoriasis 5
Basal
Differentiating
Terminal
7.82x10-75
4.88x10-178
1.20x10-298
5.48x10-78
4.65x10-59
3.72x10-156
2.12x10-2
7.97x10-7
2.06x10-4
3.52x10-6
3.20x10-41
3.26cx10-134
1.13x10-8
2.51x10-21
5.75x10-58
1.31x10-26
3.58x10-41
2.83x10-47
1.46x10-161
1.64x10-153
1.95x10-288
Cytokine Response Signature Score
Basal
Differentiating
Terminal

Figure S6. Keratinocyte Differentiation Trajectories, related to Figure 6

A. Heatmaps showing enrichment of genes along pseudo-temporal trajectories for keratinocytes from 2 normal skin biopsies and 5 psoriatic keratinocyte biopsies.

B. Plots showing the expression of trajectory-defining genes for 2 normal and 5 psoriatic biopsies.

C. Scatterplot showing the relationship between differential expression between psoriatic and normal keratinocyte (y-axis) and differential pseudotime correlation (x-axis). Genes highlighted in red have differential correlation values greater than 0.4 or less than -0.4. Gene names highlighted in red are genes examined through *in situ* staining shown in **Figure 5H**.

D. Violin plots showing localization of cytokine response signatures in basal, differentiating and terminal keratinocytes across 2 normal and 5 psoriatic biopsies. P-values from T-tests showing differences in cytokine signature scores for basal, differentiating and terminal keratinocytes between normal and psoriatic keratinocytes.

Seq-Well S^3 Master Protocol

As outlined in:

Highly Efficient, Massively-Parallel Single-Cell RNA-Seq Reveals Cellular States and Molecular Features of Human Skin Pathology

Travis K Hughes[1,2,3,4,5,12], Marc H Wadsworth II[1,3,4,5,12], Todd M Gierahn[5,6,12], Tran Do[7], David Weiss[7], Priscila R. Andrade[7], Feiyang Ma[7], Bruno J. de Andrade Silva[7], Shuai Shao[8], Lam C Tsoi[8], Jose Ordovas-Montanes [3,9,10,11], Johann E Gudjonsson[8], Robert L Modlin[9], J Christopher Love[3,4,5,6,13], and Alex K Shalek[1,2,3,4,5,13, 14]

[1] Institute for Medical Engineering & Science (IMES) and Department of Chemistry, MIT, Cambridge, Massachusetts, USA

[2] Department of Immunology, Harvard Medical School, Boston, MA 02115, USA

[3] Broad Institute of MIT and Harvard, Cambridge, Massachusetts, USA

[4] Ragon Institute of MGH, MIT and Harvard, Cambridge, Massachusetts, USA

[5] Koch Institute for Integrative Cancer Research, MIT, Cambridge, Massachusetts, USA

[6] Department of Chemical Engineering, MIT, Cambridge, Massachusetts, USA

[7] Division of Dermatology and Department of Microbiology, Immunology and Molecular Biology, David Geffen School of Medicine at UCLA, Los Angeles, California, USA

[8] Department of Dermatology, University of Michigan, Ann Arbor, Michigan, USA

[9] Division of Gastroenterology, Boston Children's Hospital, Boston, MA, USA.

[10] Department of Pediatrics, Harvard Medical School, Boston, MA, USA.

[11] Harvard Stem Cell Institute, Cambridge, MA, USA.

[12] These authors contributed equally to this work

[13] These senior authors contributed equally to this work

[14] Lead Contacts: Alex K. Shalek (AKS), shalek@mit.edu; J. Christopher Love, clove@mit.edu

Table of Contents

In-Depth Protocol

3 Membrane Preparation

7 Bead Loading

11 Cell Loading

12 Membrane Sealing

16 Cell Lysis and Hybridization

17 Bead Removal Method 1

18 Reverse Transcription & Exonuclease Digestion

19 Second Strand Synthesis & PCR

22 Library Preparation

24 Sequencing

Appendices

25 A: Array Synthesis

29 B: Synthesis Protocol Checklist

31 C: Mounting the Master

32 D: Buffer Guide

35 E: Bead Removal Method 2

36 F: Imaging in Array

37 G: Shopping List

Shalek & Love Labs, MIT

Membrane Preparation

1. Carefully place a pre-cut (22 x 66 mm) polycarbonate membrane onto a glass slide using a gloved finger and tweezers to separate the membrane and paper.
   Note 1: Make certain the shiny side of the polycarbonate membrane is facing up to be in contact with the oxygen plasma and eventually the surface of the array.
   Note 2: Discard any membranes that have creases or other large-scale imperfections.

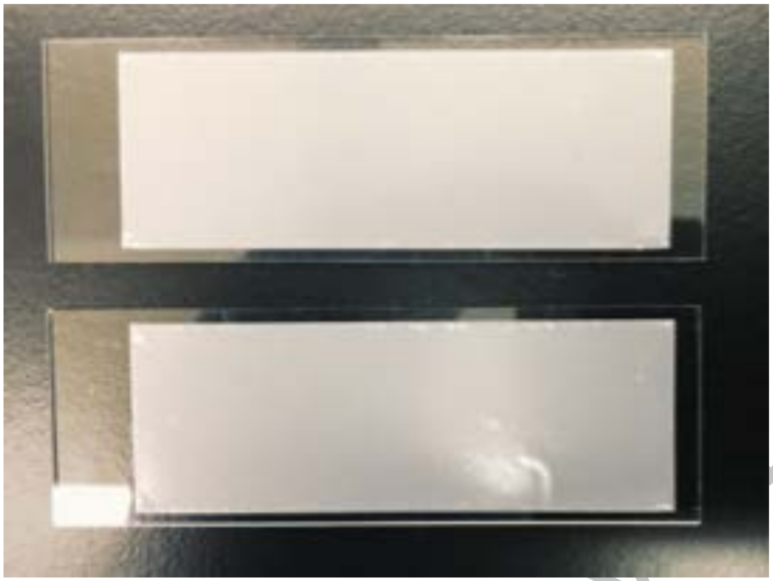

2. Place membranes onto a shelf in the plasma cleaner.
   Note 1: Shelves are not provided, but any piece of glass will do.
   Note 2 (optional): If you have two shelves, place membranes on the bottom shelf to reduce risk of them flying after vacuum is removed.

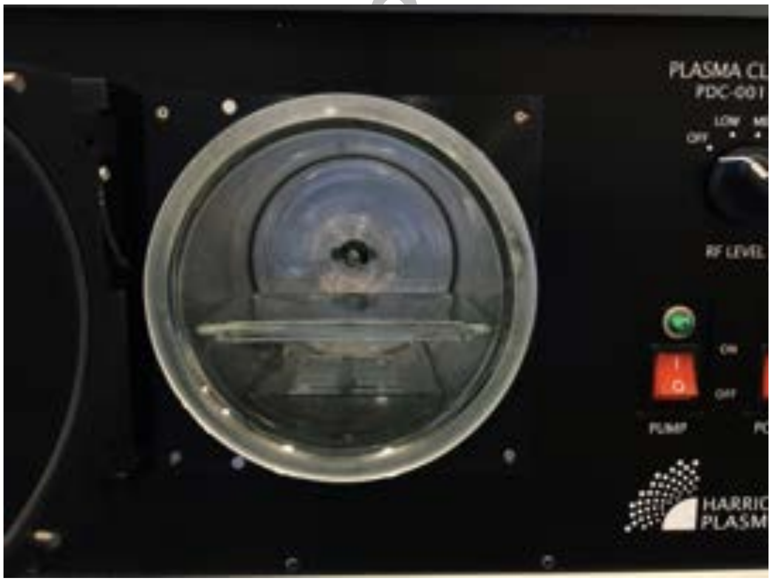

3. Close the plasma cleaner door, then turn on the main power and pump switch. To

[illegible] Labs, MIT

form a vacuum, ensure that the 3-way valve lever is at the 9:00 position as shown below and that the door is completely shut.

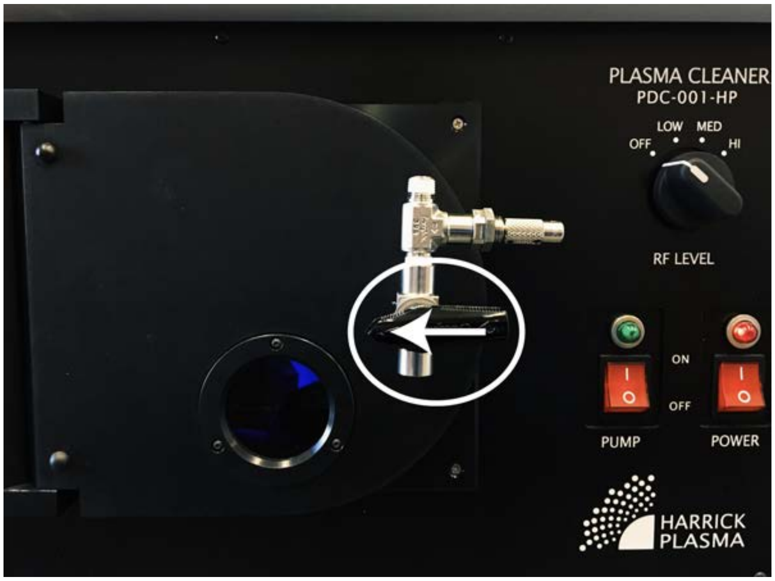

4. Allow vacuum to form for 2-3 minutes. Once the vacuum has formed, simultaneously turn the valve to 12:00 while turning the power to the Hi setting (shown below).
Note: The plasma should be a bright pink. If not, adjust the air valve to increase or decrease the amount of oxygen entering the chamber.

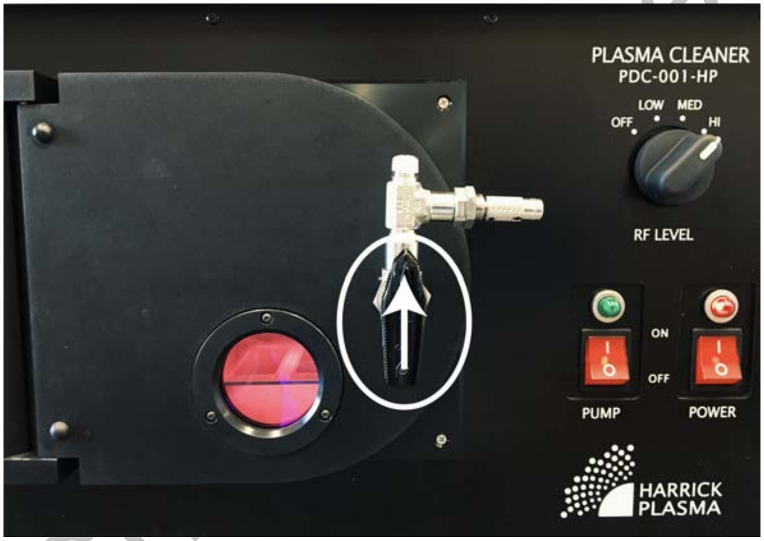

Labs, MIT

5. Treat membranes with plasma for 5-7 minutes.
Note: We treat membranes for 7 minutes, but treatment times can vary.

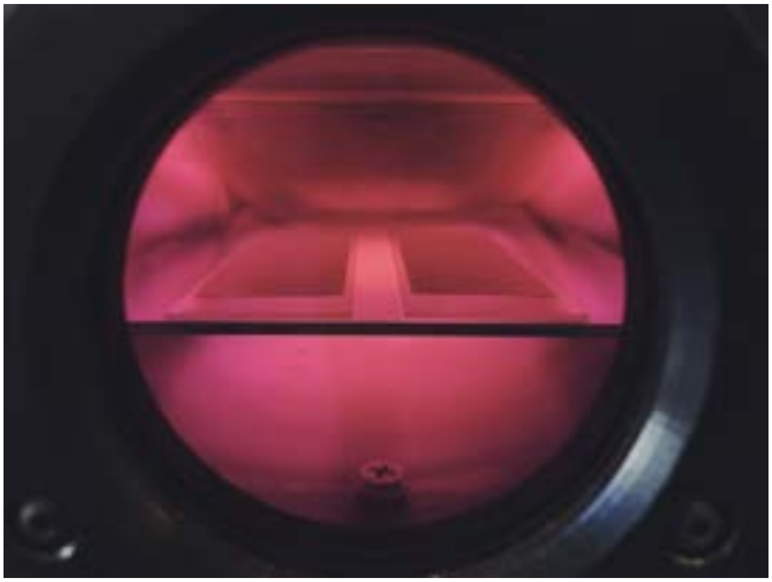

6. **Critical** – After treatment, in the following order: **(1)** turn the RF level valve from HIGH to OFF, **(2)** turn the air valve from the 12:00 position to the 9:00 position, and **(3)** then turn off the power followed by turning off the vacuum. Then *slowly open* the valve until air can be heard entering the chamber (approximate valve position shown below). Leave until door opens (~5 min).

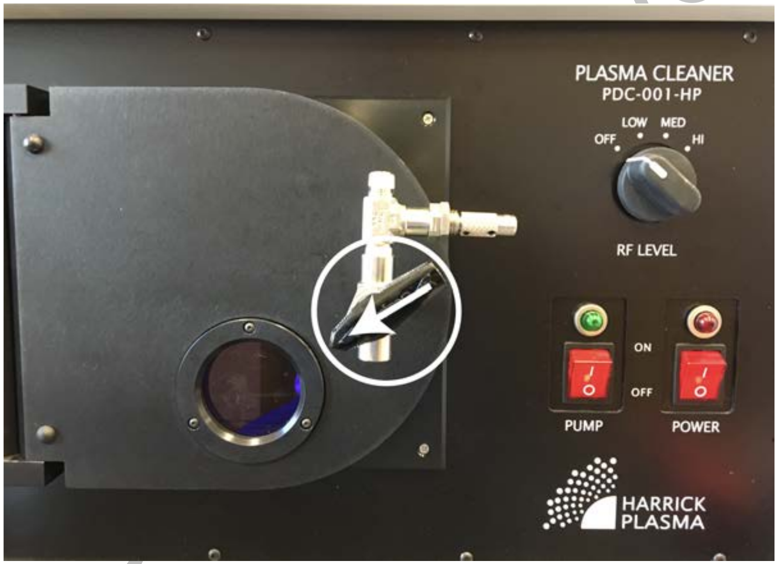

7. Remove slides (with membranes) from the plasma clean and transfer to a 4-well dish.
Note 1: If membranes have slightly folded over, slowly flip the membrane back using needle nose tweezers.
Note 2: If membranes have blown off the slide entirely, repeat above procedure to ensure you know which side was exposed to plasma.

e Labs, MIT

8. Using a P1000 pipette, gently hydrate one end of the membrane with a single drop of 1xPBS so that it adheres to the slide before dispensing the entire volume. Once the membrane is hydrated, continuing add 1xPBS until you reach 5 mL (use either a serological pipette or P1000 pipette to complete hydration).

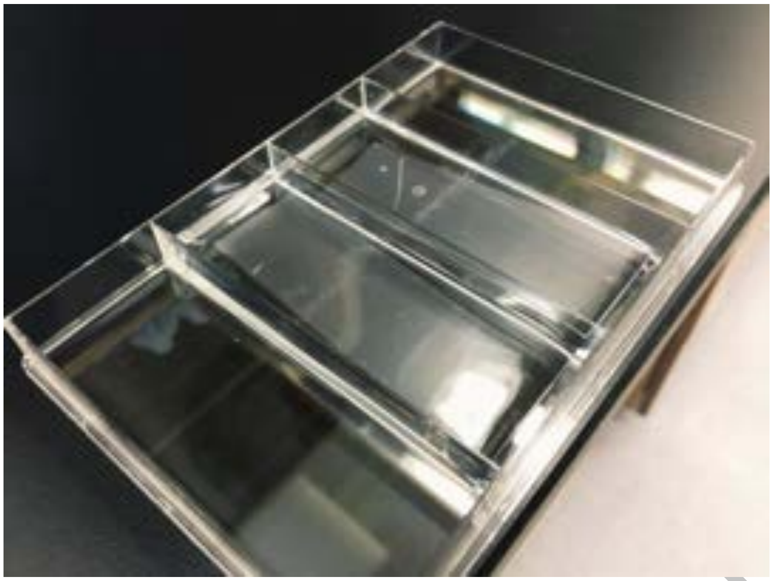

9. Remove any air bubbles underneath the membrane using wafer forceps or a pipette tip.

10. Membranes are now functionalized and ready for use.
Note 1: Membranes solvated with 1xPBS should be used within **48 hours.**
Note 2: If transporting solvated membranes (e.g. between buildings), remove all but ~1 mL of 1xPBS to prevent membranes from flipping within the dish.
Note 3: Alternatively, membranes initially solvated in 1xPBS can be dried and stored for 4 weeks at room temperature. To dry them out, carefully remove membranes, keeping them on their glass slides, from the 1xPBS solution, transfer the membranes to the benchtop, cover them with a tip box, and let them dry for 15-20 minutes. As the membranes dry they'll become opaque which is normal.
Note 4: Before use the membranes should be rehydrated with 5 mL of 1xPBS. Drying out membranes is helpful when traveling or when running seq-well in a laboratory without access to a plasma cleaner.

EXPERIMENTAL NOTES

Shalek & Love Labs, MIT

Bead Loading

1. Aspirate storage solution and solvate each array with 5 mL of bead loading buffer (BLB; **See Appendix D: Buffers Guide**).

2. Place array(s) under vacuum with rotation (50 RPM) for 10 minutes to remove air bubbles in wells. **Note:** Rotation is optional

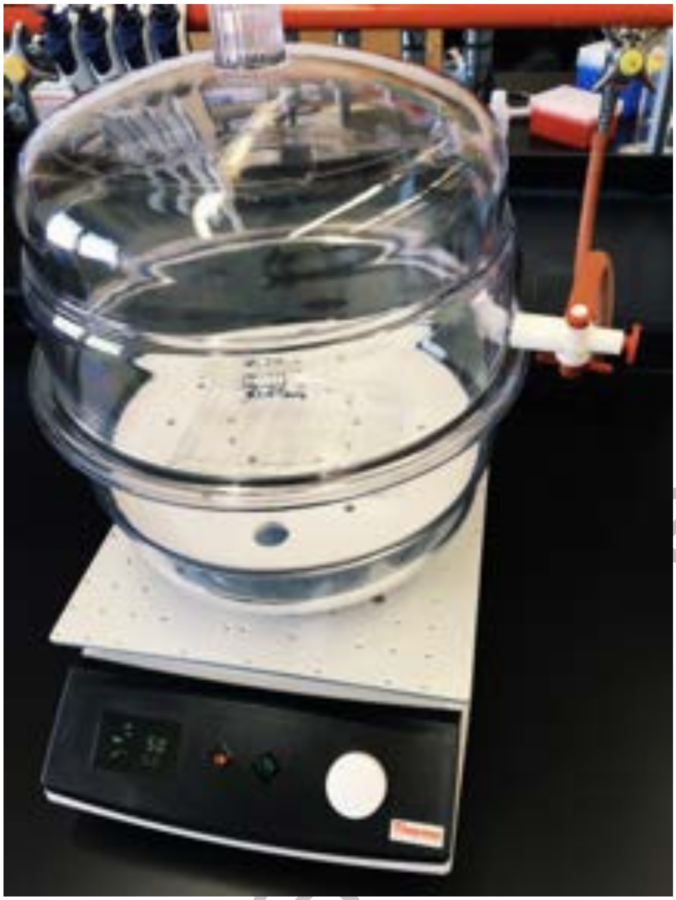

3. Aliquot ~110,000 beads from stock into a 1.5 mL tube and spin on a tabletop centrifuge for 15 seconds to form a pellet.

4. Aspirate storage buffer and wash beads twice in 500 uL of BLB.

5. Pellet beads, aspirate BLB, and resuspend beads in 200 uL of BLB.
Note: For each array, it's recommended to load ~110,000 beads.

6. Before loading beads, thoroughly aspirate BLB from the dish containing the array(s), being careful not to aspirate or dry the PDMS surface of the array(s).

Shale[illegible]e Labs, MIT

7. Using a P200 pipette, apply 200 uL containing 110,000 beads, in a drop-wise fashion, to the surface of each array (see image below and Bead Loading Diagram on page 10).

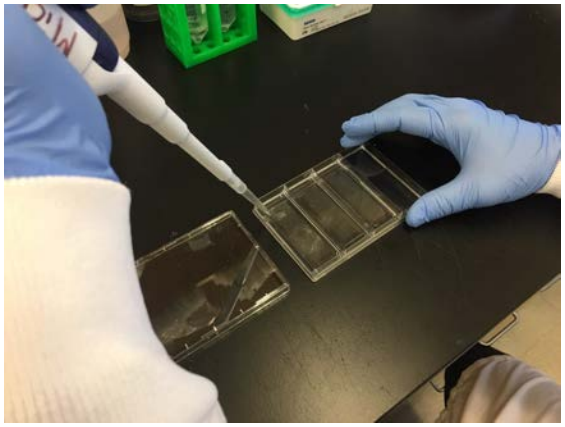

8. Allow the arrays to sit for 5 minutes, rocking them intermittently in the x & y direction. **Pro-Tip:** This step can be extended to 10 minutes to allow the beads more time to settle. However, make sure to monitor the surface of the array so that it doesn't dry out.

9. Thoroughly wash array(s) to remove excess beads from the surface. For each wash:
   1. Position each array so that it sits in the center of the 4-well dish.
   2. Dispense 500 uL of BLB in the upper right corner of each array and 500 uL in the bottom right corner of each array. Be careful not to directly pipette onto the microwells, as it can dislodge beads.
   3. Using wafer forceps or a pipette tip, push each array against the left side of the 4-well dish to create a capillary flow; this will help remove beads from the surface.
   4. Aspirate the liquid, reposition each array, and repeat on the other side.

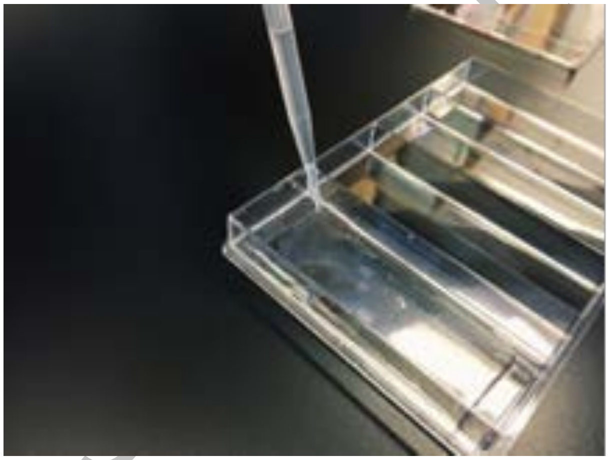

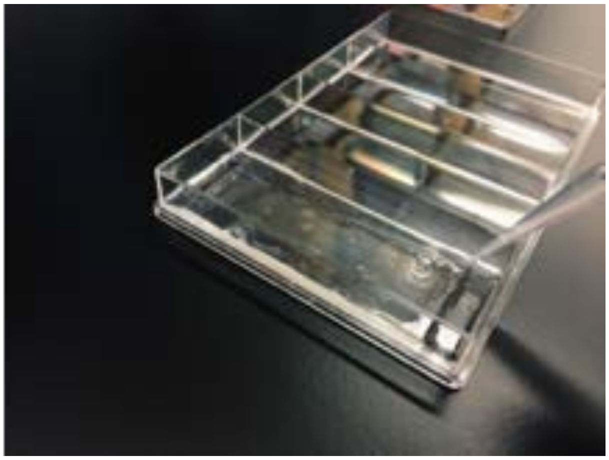

10. Repeat step 9 as necessary. Periodically examine the array(s) under microscope to confirm that no loose beads are present on the surface, as this will interfere with membrane attachment. Usually it takes 4 washes/side to thoroughly remove excess beads (this depends on your original loading density).

ove Labs, MIT

11. Once excess beads have been removed from the surface, solvate each array with 5 mL of BLB and proceed to cell loading.

Notes:

1. If continuing to cell loading immediately (i.e., within 6 hours), loaded arrays should be stored in 5 mL of BLB.
2. If you are not going to use the arrays on the day they're loaded, remove the BLB buffer, rinse the arrays once with 5 mL of 1xPBS, and then solvate the arrays with 5 mL of quenching buffer. Arrays can be stored in quenching buffer for 10 days (**See Appendix D: Buffers Guide**).

EXPERIMENTAL NOTES

Shalek & Love Labs, MIT

Bead Loading Diagrams

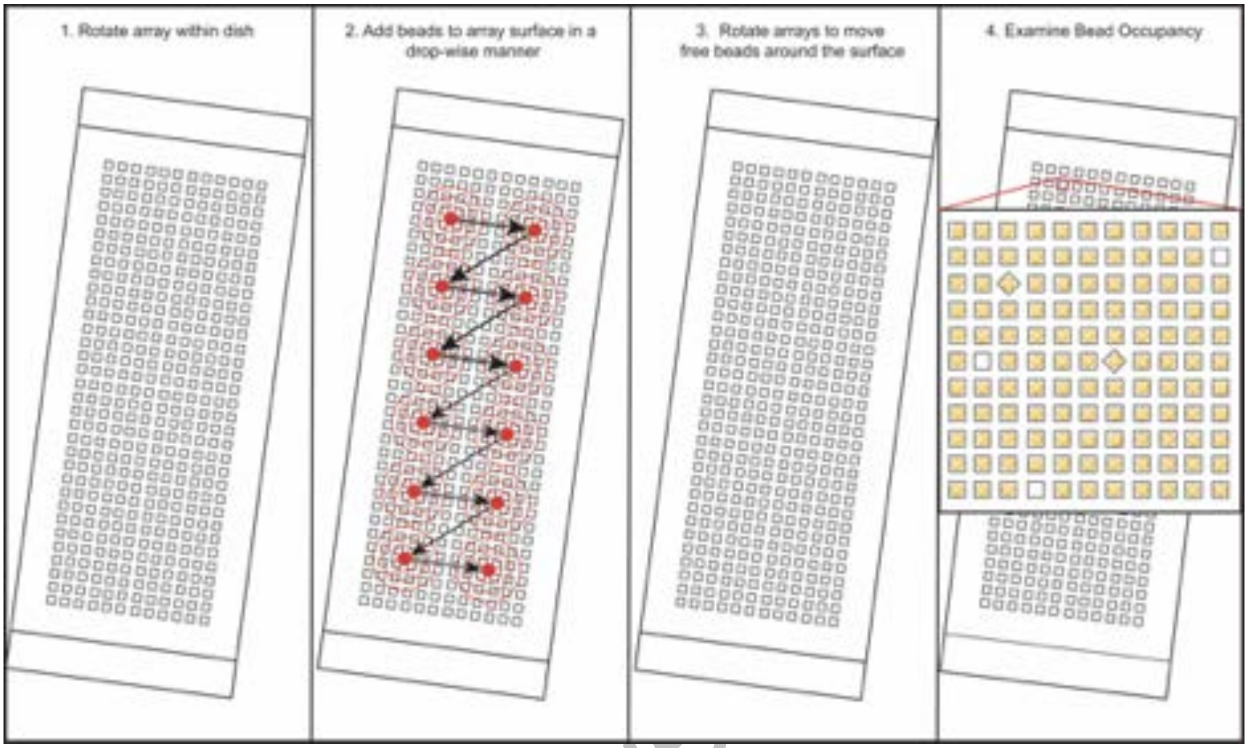

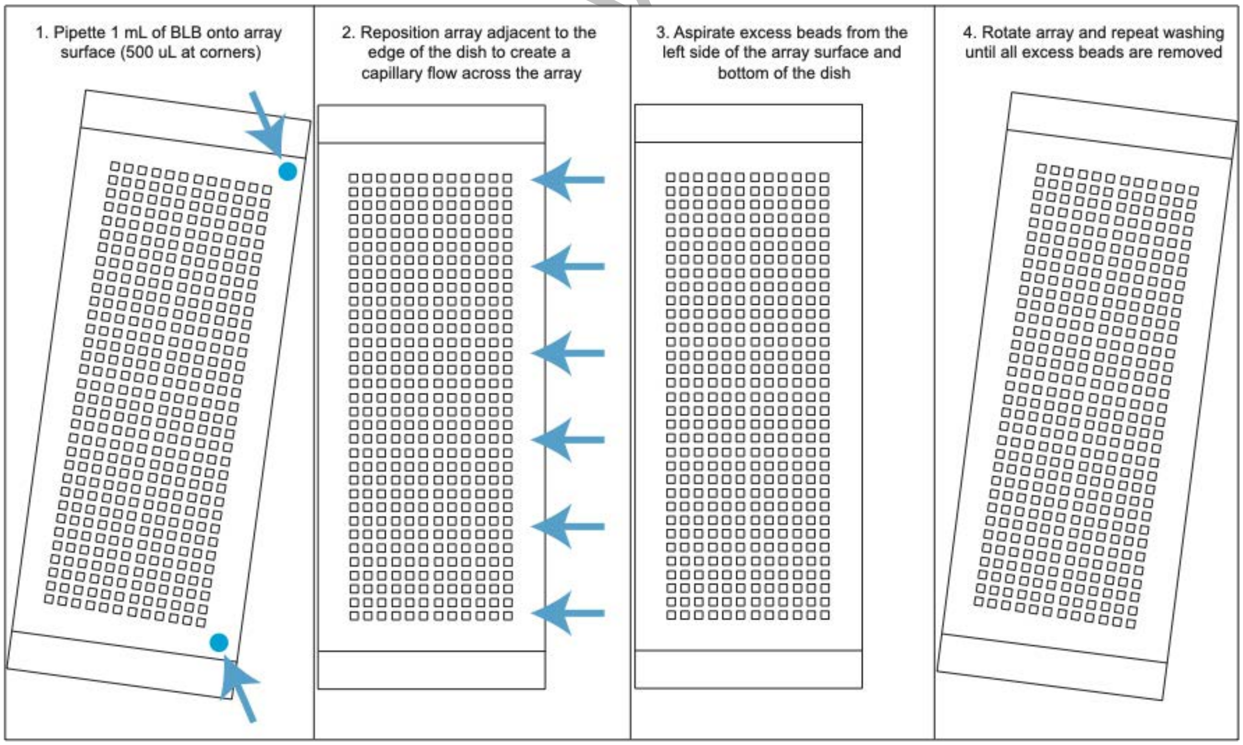

Cell Loading (Without Imaging)

If you want to image cells in the array, please refer to Appendix F

1. At this point, your array should be loaded with beads and sitting in 5 mL of BLB.

2. Obtain the cell or tissue sample and prepare a single cell suspension using an optimized protocol for tissue dissociation.

3. While preparing your single-cell suspension, aspirate the BLB from each array (or quenching buffer) and rinse the array twice in 5 mL of 1xPBS to bring the solution in the four-well dish to physiological pH.

4. After the second wash, aspirate the 1xPBS and soak the loaded array in 5 mL of RPMI + 10% (RP-10) FBS for 5 minutes.
   Note 1: This step is performed to mitigate non-specific adhesion of cells to primary amines on the top surface of the array.
   Note 2: Any supplemented media can be used in place of RP-10.

5. After obtaining a single-cell suspension, count cells using a hemocytometer and make a new solution of 10,000-15,000 cells in 200 uL of RP-10.
   Note 1: You can use your preferred media for prepping the cell loading solution.
   Note 2: Be sure to not use automated cell counters, particularly following tissue dissociation. This can provide an inaccurate cell count, compromising the experiment.

6. Thoroughly aspirate the RP-10/supplemented media (to ensure the array will not move during cell loading).
7. Center your array in the well and then apply the cell loading solution onto the surface in a dropwise fashion (similar to how beads were applied in the previous section).

8. Allow cells to settle for 10 minutes, intermittently rock the array in the x & y direction.

9. Wash array 4x with 5 mL of 1xPBS to remove the serum. For each wash, gently rock the array in the x & y direction, and then aspirate the 1xPBS. Once you have aspirated the 1xPBS out of the dish, gently tilt the 4-well dish toward you and aspirate directly off the bottom border of the array; this will help to completely remove the excess serum on the surface of the array
   Note: These washes are critical to remove excess serum which can interfere with successful membrane attachment.

10. Aspirate the final 1xPBS wash and replace with 5 mL of RPMI media ***without*** FBS.
    Note: You can use any media here as long as it **does not** contain serum.

EXPERIMENTAL NOTES

Shalek & Love Labs, MIT

Membrane Sealing

1. Gather the following materials before sealing the array(s):

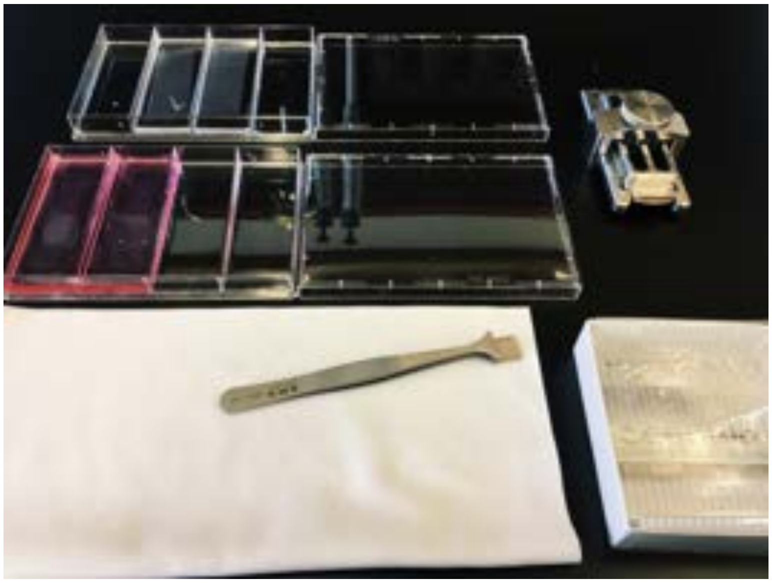

- Array loaded with beads and cells (See Bead/Cell Loading)
- Pre-treated membrane (See Membrane Preparation)
- Wafer forceps (or P1000 pipette tip)
- Paper towels
- Agilent clamp
- Clean microscope slides

2. Use the wafer forceps to transfer the array from media to the lid of a 4-well dish, being careful to ensure that the array is not tilted.

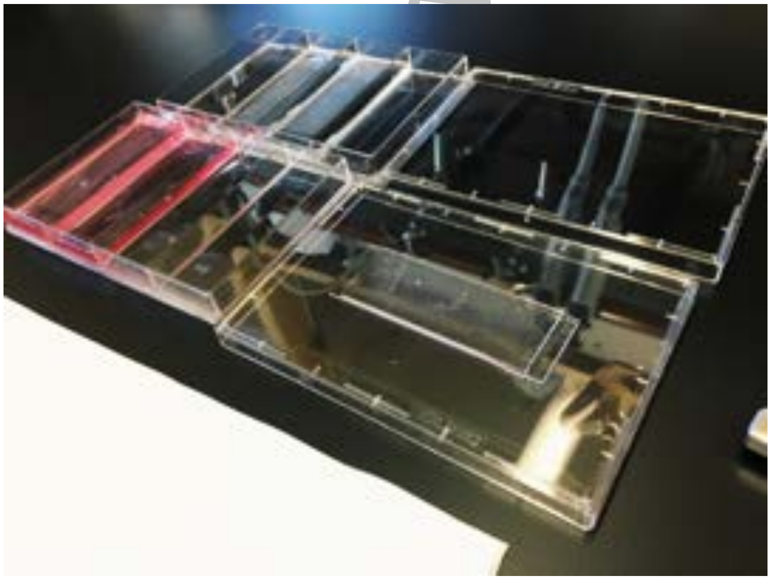

Love Labs, MIT

3. Once the array is positioned on the lid of a 4-well dish, carefully aspirate excess liquid from around the edge of the array and the exposed surface of the glass slide. (Note: Be careful not to aspirate directly from the PDMS surface).

4. Using wafer forceps or a pipette tip, remove a pre-treated membrane from the 4-well dish.

5. Gently dab away moisture from the glass slide on the paper towel until the membrane does not spontaneously change position on the glass slide.

6. Carefully position the membrane on the center of the microscope slide, leaving a small membrane overhang (2-3 mm) beyond the edge of slide.

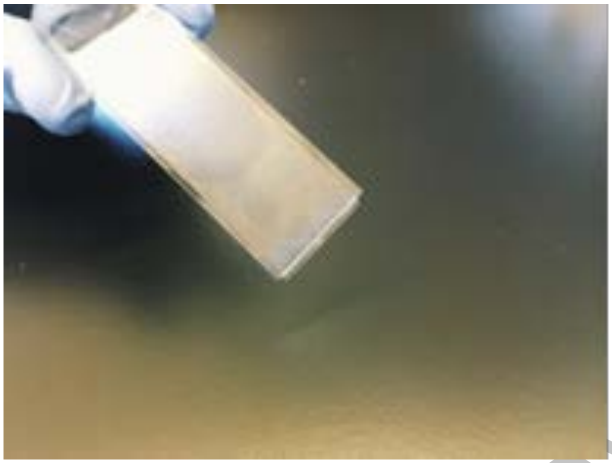

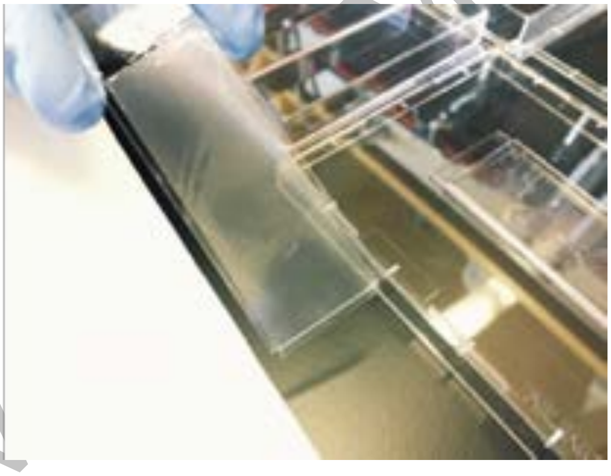

7. Holding the membrane in your left hand, invert the microscope slide so that the treated surface of the membrane is facing down.

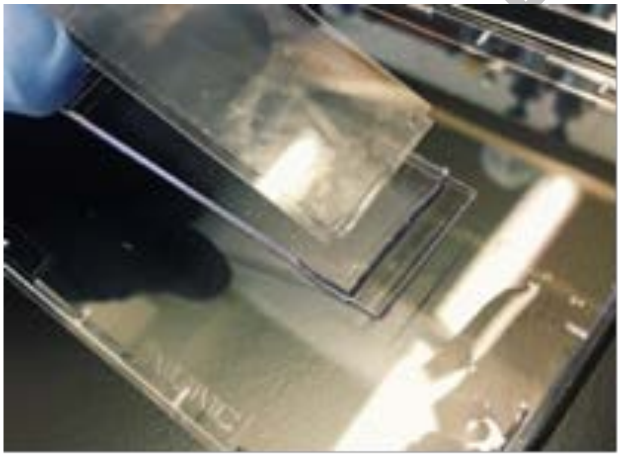

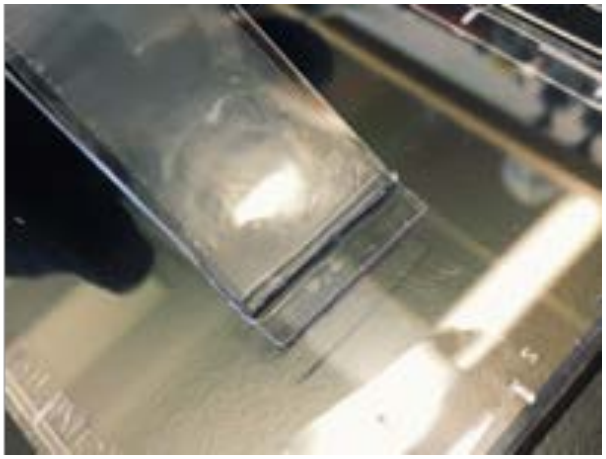

EXPERIMETNAL NOTES

8. Place the overhang of the membrane in contact with the PDMS surface of the array just beyond the boundary of the microwells.

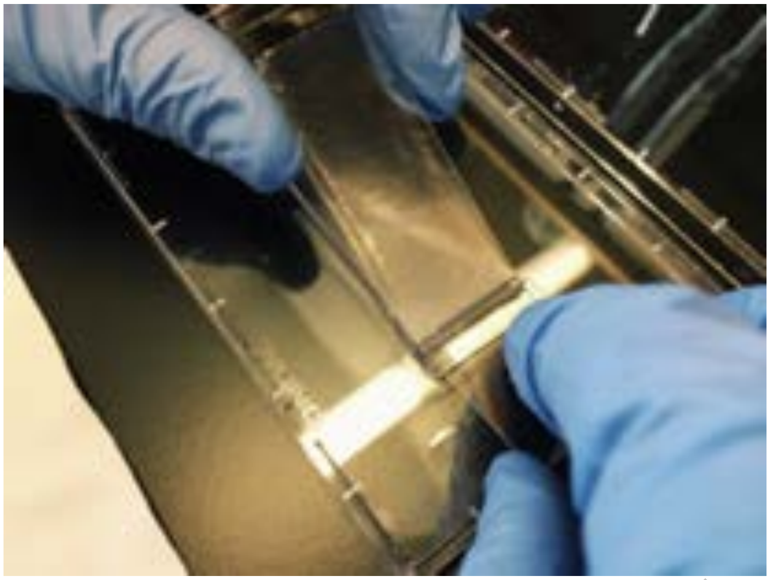

9. Using a clean slide held in your right hand, firmly hold down the overhang of the membrane against the PDMS surface of the array.

10. **Critical Step:** While maintaining pressure with your right hand to hold the membrane in place, gently apply the membrane.
Note 1: For optimal results, use only the weight of the slide to apply the membrane with the left hand.
Note 2: Attempts to manually seal the microwell device using excess pressure result in a 'squeegee' effect, effectively removing moisture from the membrane while fixing membrane creases in place.
Note 3: As you apply the membrane you should see a fluid interface form and expand as direct, uniform contact between the slide and the array will naturally remove some of the media as the membrane is applied.
Note 4: You can use either your left or right hand for membrane-sealing (most people use their dominant hand to apply the membrane). Please practice this step before the actual experiment to figure out which hand you're most comfortable with.

11. After applying the membrane, carefully pry the array and membrane from the surface of the lid and transfer to an Agilent clamp.

12. After transferring the sealed array to the clamp, place a glass slide on top of the sealed array.

Shalek & Love Labs, MIT

13. Close the clamp and tighten to the point of resistance, then place it in a 37C incubator for 30-40 minutes.
Note: This time is flexible and depends on the incubator. If you want to decrease this incubation time, please optimize on cell lines before proceeding with precious samples.

14. Repeat membrane-sealing protocol procedure if running multiple arrays.

EXPERIMETNAL NOTES

Shalek & Love Labs, MIT

Cell Lysis & Hybridization

1. Remove the clamp from the incubator, and then remove the array from the Agilent clamp. (Note: At this point, the glass slide will be attached to the array and membrane).

2. Submerge the array, with top slide still attached, in 5 mL of complete lysis buffer (**See Appendix D: Buffers Guide**).

3. Gently rock the array in lysis buffer until the top glass slide spontaneously detaches.
Note 1: Do not pry the top slide off as this can reverse membrane sealing. The time necessary for detachment of the top slide varies (10 seconds – 10 minutes).
Note 2: If the top slide does not release after 10 minutes, gently pry the top slide off using wafer forceps or a pipette tip. Just be careful.

4. Once the top slide has detached, place the arrays on a horizontal rotator for 20 minutes at 50-60 rpm.

5. After 20 minutes, remove the lysis buffer and wash each array with 5 mL of hybridization Buffer (**See Appendix C: Buffers Guide**).
Note 1: Use a separate waste container for lysis buffer because guanidine thiocyanate can react with bleach in TC traps to create cyanide gas.
Note 2: The hybridization buffer used to wash the array post-lysis may contain trace amounts of guanidine thiocyanate and should, therefore, be disposed of in the lysis buffer waste container.

6. Aspirate hybridization buffer and add another 5 mL of hybridization buffer to each array and rotate for 40 minutes at 50-60 rpm.

7. While the arrays are rocking in hybridization buffer, prepare RT master mix. (See **Reverse Transcription & Exonuclease Digestion**)

EXPERIMENTAL NOTES

Shalek & Love Labs, MIT

Bead Removal Method 1

1. After the arrays have rocked in hybridization buffer for 40 minutes, carefully peel back each membrane using fine-tipped tweezers.

2. Place array into a 50 mL conical containing 30-40 mL of Wash 1 solution.

3. Holding the array above the 50mL conical (shown below), repeatedly dispense approximately 1 mL of Wash 1 solution from the conical across the surface of the array to dislodge beads (**See Appendix D: Buffers Guide**).
   Note: Vigorously dispense Wash 1 buffer to remove beads.

4. Repeat these 10 times, periodically checking to see if beads are dislodging.

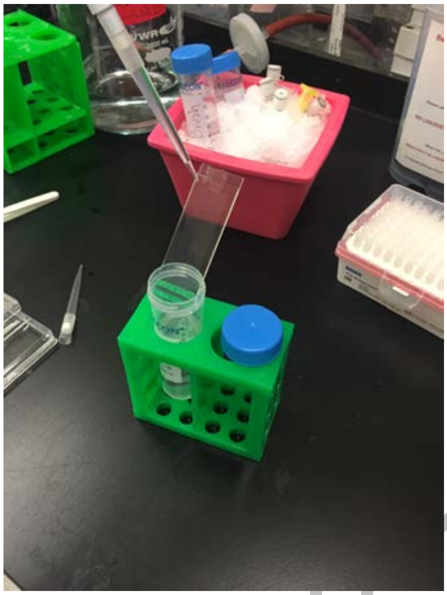

5. After repeatedly rinsing the array from top to bottom, use a clean glass slide to ***gently*** scrape the array to remove any beads that remain in the array.
   Note: At this point it is possible to visually inspect the array to assess bead removal.

6. Once you are satisfied with bead removal, place the empty array back in the 4-well disk, cap the 50 mL conical, and pellet beads for 5 minutes at 1000xg.
   Note 1: You can visually inspect the success of your bead removal by looking at the arrays under a light microscope.
   Note 2: Where possible, use a swinging bucket centrifuge to collect beads. The use of a fixed-rotor centrifuge can lead to the formation of a bead pellet on the elbow rather than the bottom of the conical tube, which can lead to inefficient recovery.

7. After centrifugation, aspirate all but ~1 mL of excess Wash Buffer, collect the beads using a P1000 pipette, and transfer beads suspended in wash buffer to a separate 1.5 mL eppendorf tube for each array.

Shalek & Love Labs, MIT

Reverse Transcription & Exonuclease Digestion

Reverse Transcription (RT)

1. Prepare the following RT mastermix during the hybridization step:

40 uL	H_2O
40 uL	Maxima 5X RT Buffer
80 uL	30% PEG8K
20 uL	10 mM dNTPs (Clontech)
5 uL	RNase Inhibitor (Lucigen)
5 uL	100 uM Template Switch Oligo
10 uL	Maxima H-RT

Note: Add the Maxima H-RT enzyme to the mastermix immediately before adding to beads.

2. Centrifuge eppendorf tubes containing collected beads for 1 minute at 1000xg.

3. Remove supernatant and resuspend in 250 uL of 1X Maxima RT Buffer and centrifuge beads for 1 minute at 1000xg.

4. Aspirate 1X Maxima RT Buffer and resuspend beads in 200 uL of the RT mastermix.

5. Incubate at room temperature for 30 minutes with end-over-end rotation. After 30 minutes, incubate at 52C for 90 minutes with end-over-end rotation.
   Note: The reverse transcription reaction can proceed overnight, if necessary.

7. Following the RT reaction, wash beads once with 500 uL of TE-SDS, and twice with 500 uL of TE-Tween (TE-TW). **Following Reverse Transcription, beads can be stored at 4C in TE-TW.**

Exonuclease I Treatment

1. Prepare the following Exonuclease I Mix:

20 uL	10x ExoI Buffer
170 uL	H_2O
10 uL	ExoI

2. Centrifuge beads for 1 minute at 1000xg and aspirate the TE-TW solution.

3. Resuspend in 500 uL of 10 mM Tris-HCl pH 8.0.

4. Centrifuge beads again, remove supernatant and resuspend beads in 200 uL of exonuclease I mix.

5. Incubate at 37C for 50 minutes with end-over-end rotation.

6. Wash the beads once with 500 uL of TE-SDS, twice with 500 uL TE-TW.
   Beads can be stored at 4C in TE-TW.

Shalek & Love Labs, MIT

Second Strand Synthesis & PCR

Second Strand Synthesis (*Beginning after 2nd wash of TE-TW after Exo treatment)*

1. Prepare the following 2nd strand synthesis mix:

40 uL	Maxima 5X RT Buffer
80 uL	30% PEG8000
20 uL	10 mM dNTPs (Clontech)
2 uL	1 mM dN-SMRT oligo
5 uL	Klenow Enzyme
53 uL	H_2O

Note: Add the Klenow enzyme immediately before adding to beads.

2. After aspiration of 2nd TE-TW wash, resuspend beads in 500 uL 0.1 M NaOH.
Note: Make the 0.1 M NaOH solution fresh each time you perform second strand synthesis.

3. Rotate tube for 5 min at room temp, then spin (800xg for 1 minute) and aspirate supernatant.

4. Wash once with 500 uL of TE-TW, and once with 500 uL 1xTE

5. Resuspend beads in 200 uL 2nd strand synthesis reaction and rotate end-over-end at 37C for 1 hr.

6. Wash beads twice with 500 uL TE-Tween and once with 500 uL TE

7. Proceed directly with the PCR protocol.

PCR (Whole Transcriptome Amplification (WTA))

1. Prepare the following PCR mastermix:

25 uL	2X KAPA HiFi Hotstart Readymix
14.6 uL	H_2O
0.4 uL	100 uM SMART PCR Primer
40 uL	per reaction

2. Wash beads once with 500 uL of water, pellet beads, remove supernatant and resuspend in 500 uL of water.
Note 1: If you do not want to count the beads then after the 500 uL water wash in step 2, resuspend the beads in 240 uL of water and proceed to step 6.
Note 2: If you choose this path, prepare mastermix for 24 PCR reactions for each array being processed.

Shalek & Love Labs, MIT

3. Mix well (do not vortex) to evenly resuspend beads and transfer 20 uL of beads to a separate 1.5 mL tube to count the beads.
   Note: Don't vortex beads as this can result in bead fragmentation.

4. Pellet the small aliquot of beads, aspirate the supernatant, and resuspend in 20 uL of bead counting solution (10% PEG, 2.5 M NaCl).
   Note: The bead counting solution aids in even dispersion of beads across a hemocytometer.

5. Count the beads using a hemocytometer.

6. Add 40 uL of PCR mastermix per reaction to 96-well plate.

7. Add 1,500 – 2,000 beads per reaction in 10 uL of water for a total volume of 50 uL per PCR reaction, making certain to PCR the entire array.

8. Use the following cycling conditions to perform whole-transcriptome amplification:

**Start:**	
95C	3 minutes
**4 Cycles:**	
98C	20 seconds
65C	45 seconds
72C	3 minutes
**9-12 Cycles:**	
98C	20 seconds
67C	20 seconds
72C	3 minutes
**Final Extension:**	
72C	5 minutes
4 C	Infinite hold

   Note: The total number of PCR cycles necessary for amplification depends on the cell type used.
   - 13 cycles are optimal for cell lines or larger cells (e.g. macrophages)
   - 16 cycles are optimal for primary cells

EXPERIMENTAL NOTES

Shalek & Love Labs, MIT

Purification of PCR products and analysis on the BioAnalyzer or Agilent TapeStation

1. Pool PCR products from between 6 and 8 PCR reactions in a 1.5 mL microcentrifuge tube so that you have 10-12,000 beads/1.5 mL microcentrifuge tube.

2. Purify PCR products using Ampure SPRI beads and the following protocol:
   Note: Please refer to the Ampure SPRI bead official protocol for more details.
   A. Spri at 0.6x volumetric ratio.
   B. Allow the tubes to sit on the tube-rack off the magnet for 5 minutes, and then place the rack on the magnet for 5 minutes.
   C. Perform 3 washes with 80% ethanol.
      Note: At each wash step rotate each tube 180 degrees 6 times to allow beads to pass through the ethanol solution to the opposite side of the tube.
   D. After the third wash, remove the 80% ethanol wash solution. Further, use a P200 with fresh tips to remove any residual ethanol and allow beads to dry for 10-15 minutes. (**Note**: Beads will have a cracked appearance once dry). Remove the rack from the magnet, elute dried beads in 100 uL, place the rack on the magnet and then transfer the 100 uL supernatant which contains eluted DNA to a new 1.5 mL microcentrifuge tube or 96-well plate.
   E. Spri the 100 uL at 1.0x volumetric ratio and repeat steps b and c
   F. After the third wash, allow the beads to dry for 15 minutes, remove the rack from the magnetic, elute the beads in 15 uL, place the rack back on the magnet and then transfer the 15 uL to a new 1.5 mL microcentrifuge tube or 96-well plate.

3. Run a BioAnalyzer High Sensitivity Chip or Agilent D5000 High Sensitivity Screentape according to the manufacturer's instructions.
   Use 1 uL of the purified cDNA sample as input.
   - Your WTA library should be fairly smooth, with an average bp size of 0.7-2 kbps.

4. Proceed to library preparation or store the WTA product at 4C (short-term) or -20C (long-term).

EXPERIMENTAL NOTES

Shalek & Love Labs, MIT

Library Preparation

Tagmentation of cDNA with Nextera XT

1. Ensure your thermocyclers are setup for Tagmentation (step 5) & PCR (step 9).

2. For each sample, combine 1000 pg of purified cDNA with water in a total volume of 5 uL. It's ideal to dilute your PCR product in a separate tube/plate so that you can add 5 uL of that for tagmentation.
   Example: For 1000 pg reactions, dilute PCR product, in a new plate, to 200 pg/uL, then you can add 5 uL of this to a reaction tube for a 1000 pg reaction.
   Note 1: We typically perform Nextera reactions in duplicate for WTA product from each pool of 6-8 PCR reactions. For example, if you recover 3 pools/array, you would run a total of 6 nextera reactions.
   Note 2: These volumes can be reduced by half to reduce reagent costs, if desired.

3. To each tube, add 11 uL of Nextera TD buffer, then 4 uL of ATM buffer (the total volume of the reaction is now 20 uL).

4. Mix by pipetting ~5 times. Centrifuge plate at 1000x g for 10-15 seconds.

5. Incubate at 55C for 5 minutes.

6. Add 5 uL of Neutralization Buffer. Mix by pipetting ~5 times. **Note:** Bubbles are normal.

7. Incubate at room temperature for 5 minutes.

8. Add to each PCR tube:

15 uL	Nextera PCR mix
8 uL	H_2O
1 uL	10 uM New-P5-SMART PCR hybrid oligo
1 uL	10uM Nextera N7XX oligo

9. After sealing and centrifuging (1 minute at 1000xg) the PCR plate, run the following PCR program:

**Start:**	
72C	3 minutes
95C	30 seconds
**12 cycles:**	
95C	10 seconds
55C	30 seconds
72C	30 seconds
**Final Extension:**	
72C	5 minutes
4C	Infinite hold

Shalek & Love Labs, MIT

Purification of PCR products and analysis on the BioAnalyzer or Agilent TapeStation

1. Pool PCR products from between 6 and 8 PCR reactions in a 1.5 mL microcentrifuge tube so that you have 10-12,000 beads/1.5 mL microcentrifuge tube.

2. Purify PCR products using Ampure SPRI beads and the following protocol:
   Note: Please refer to the Ampure SPRI bead official protocol for more details.
   A. Spri at 0.6x volumetric ratio.
   B. Allow the tubes to sit on the tube-rack off the magnet for 5 minutes, and then place the rack on the magnet for 5 minutes.
   C. Perform 3 washes with 80% ethanol.
      Note: At each wash step rotate each tube 180 degrees 6 times to allow beads to pass through the ethanol solution to the opposite side of the tube.
   D. After the third wash, remove the 80% ethanol wash solution. Further, use a P200 with fresh tips to remove any residual ethanol and allow beads to dry for 10-15 minutes. (**Note**: Beads will have a cracked appearance once dry). Remove the rack from the magnet, elute dried beads in 100 uL, place the rack on the magnet and then transfer the 100 uL supernatant which contains eluted DNA to a new 1.5 mL microcentrifuge tube or 96-well plate.
   E. Spri the 100 uL at 1.0x volumetric ratio and repeat steps b and c
   F. After the third wash, allow the beads to dry for 15 minutes, remove the rack from the magnetic, elute the beads in 15 uL, place the rack back on the magnet and then transfer the 15 uL to a new 1.5 mL microcentrifuge tube or 96-well plate.

3. Run a BioAnalyzer High Sensitivity Chip or Agilent D1000 High Sensitivity Screentape according to the manufacturer's instructions.
   - Use 1 uL of the purified cDNA sample as input.
   - Your tagmented library should be fairly smooth, with an average bp size of 400-800 bp.
   - Smaller-sized libraries might have more polyA reads
   - Larger libraries may have lower sequence cluster density and cluster quality.

Note: We have successfully sequenced libraries from 400-800bp.

5. Proceed to sequencing.

Shalek & Love Labs, MIT

Sequencing

Once your sequencing library has passed the proper quality controls, you're ready to proceed to sequencing. For a detailed loading protocol, please consult the Illumina website for a step-by-step manual. (https://support.illumina.com/downloads.html)

NextSeq500 – Shalek Lab protocol

1. Make a 5 uL library pool at 4 nM as input for denaturation.
2. To this 5 uL library, add 5 uL of 0.2 N NaOH (make this solution fresh).
3. Flick to mix, then spin down and let tube sit for 5 minutes at room temperature.
4. After 5 minutes, add 5 uL of 0.2 M Tris-HCl pH 7.5.
5. Add 985 uL of HT1 Buffer to make a 1 mL, 20 pM library (solution 1).
6. In a new tube (solution 2), add 165 uL of solution 1 and dilute to 1.5 mL with HT1 buffer to make a 2.2 pM solution – this is the recommended loading concentration.
   Note: Optimal loading concentration is 1.8-2.5 pM
7. Follow Illumina's guide for loading a NextSeq500 Kit

Sequencing specifications for the MiSeq or NextSeq:

Read 1: 20 bp *

Read 2: 50 bp

Read 1 Index: 8 bp ← *only necessary if you are multiplexing samples*

Custom Read 1 primer

Sequencing specifications for the Nova-Seq:

Read 1: 20 bp *

Read 2: 50-80 bp

Read 1 Index: 8 bp

Read 2 Index: 8 bp (optional, but recommended)

Custom Read 1 primer

Note 1: If you're loading on a Nova-Seq you'll want to use dual-indexing to mitigate index switching.

Note 2: Read 1 can sometimes be 21 base pairs; this depends on the company and bead lot you are ordering from. Please consult with your bead provider to determine which read length to use.

NextSeq 500:
(http://support.illumina.com/content/dam/illumina-support/documents/documentation/system_documentation/nextseq/nextseq-custom-primers-guide-15057456-01.pdf)
(Follow Illumina's guide for custom primers)
MiSeq:
(http://support.illumina.com/content/dam/illumina-support/documents/documentation/system_documentation/miseq/miseq-system-custom-primers-guide-15041638-01.pdf)

Shalek & Love Labs, MIT

Appendix A: Array Synthesis

Day 0: Pouring PDMS Arrays

Note: If you need to mount your master, please refer to **appendix F**

1. Combine Sylgard crosslinker with Sylgard base at a 1:10 ratio and mix vigorously for 5 minutes to create a PDMS master mix.

2. Once mixing is complete, put your PDMS master mix under vacuum for 20 minutes to remove any air bubbles.

3. Use a 10 mL syringe to inject 6-10 mL of PDMS master mix into molds with mounted PDMS masters.

4. Incubate at 70C for 2.5 hours.

Day 1: Array Functionalization Part 1

Note: For this section, make all solutions fresh!

1. Remove excess PDMS from edges of the glass slide.

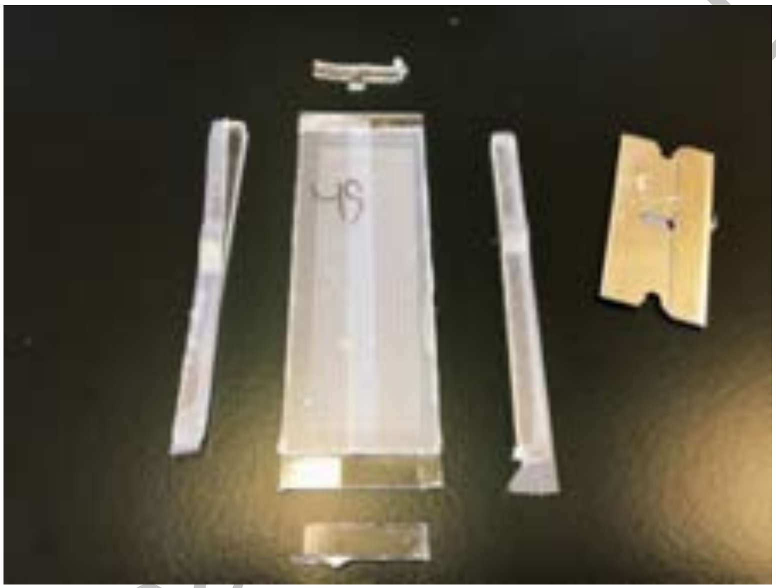

2. Use scotch tape to remove excess PDMS from the surface of the array and the glass slide.

Sh[illegible] Labs, MIT

3. Place clean arrays into a metal slide basket

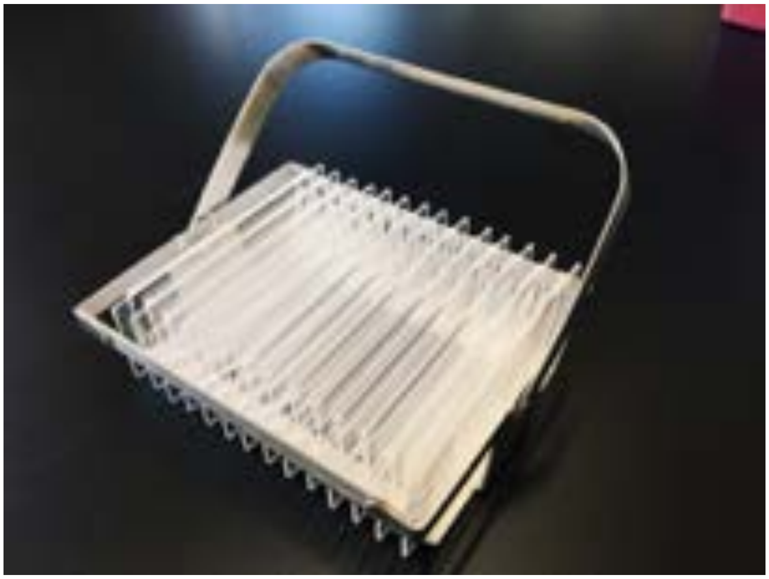

4. Rinse arrays in 100% ethanol for 5 minutes, then let dry at room temperature (RT) for 15 minutes.

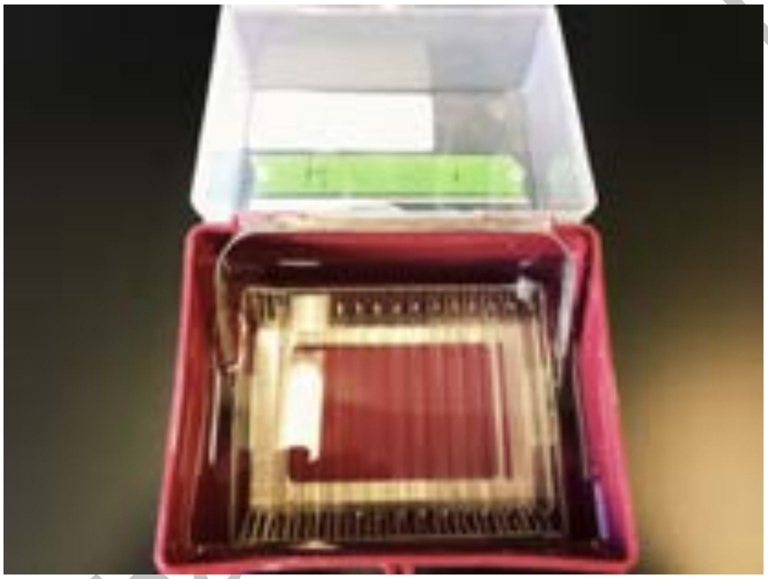

5. Plasma treat arrays on high for 5-7 minutes.
Note 1: Adjust the air valve so that the plasma is pink.

6. Following plasma treatment, immediately submerge arrays in 350 mL of 0.05% APTES in 95% ethanol for 15 minutes.

7. Spin dry arrays **(500 RPM** for 1 minute).
Note: Our rotor model is TX-10000 75003017 (Thermo) with a rotor radius of 209 mm. 500 RPM on this instrument is ~ 60xg.

8. Incubate at room temperature for 10 minutes.

9. Submerge in 300 mL of acetone and rock until all bubbles are out of the wells; this typically takes approximately 5 minutes.

10. Place in 350 mL of 0.2% PDITC/10% pyridine/90% DMF solution in a glass chamber (or polypropylene tip box) for 2 hours at room temperature.
Note: While this is rocking, prepare your chitosan solution (See **Appendix D**)

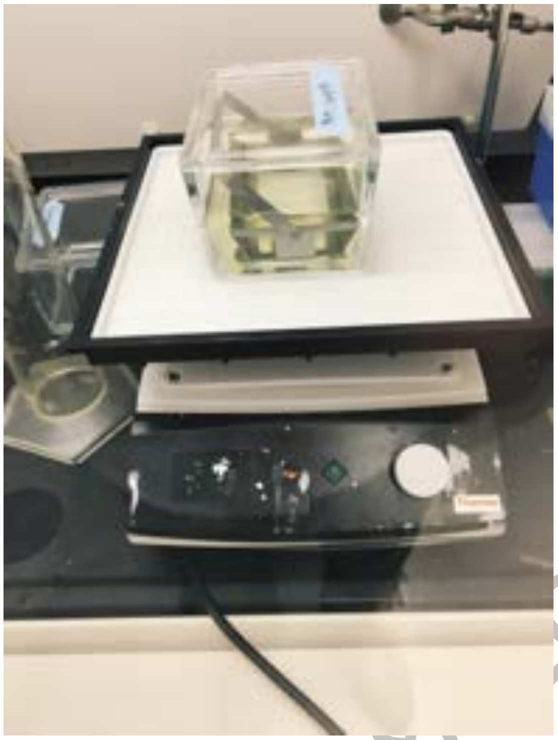

11. After the PDITC soak, wash arrays briefly in two boxes of 300 mL DMF.
Note: For each brief wash, simply dunk the arrays in the solution 5-10 times and then transfer to the new solution.

12. Dunk and wash the arrays in 300 mL of acetone.

13. Move to a fresh 350 mL of acetone and rock for 20 minutes.

14. Spin dry arrays (**500 RPM** for 1 minute).

15. Place arrays at 70C for 2 hours.

16. Remove from oven and let sit at room temperature for 20 minutes.

17. Submerge arrays in 350 mL of 0.2% chitosan solution (pH 6.0-6.1; See Appendix D) and incubate at 37C for 1.5 hours.

Shalek & Love Labs, MIT

18. Wash arrays 4x in separate 300 mL distilled water baths.

19. Submerge in 350 mL of 20 ug/mL aspartic acid, 2 M NaCl, and 100 mM sodium carbonate solution (pH 10.0)

20. Place in vacuum chamber and apply house vacuum.
    Note: You should see bubbles form indicating the solvation of wells.

21. Place vacuum chamber (still connected to house vacuum) on a rocker and rock (50-70 RPM) overnight at room temperature.

Day 2: Array Functionalization – Part 2

1. The following morning, remove arrays from vacuum and rotate at 50-60 RPM for 3 hours at room temperature.

2. Place arrays at 4C and soak 24 hours before use.
   Note: Arrays can be stored in the aspartic acid solution for 3 months at 4C.

EXPERIMENTAL NOTES

Shalek & Love Labs, MIT

Appendix B: Synthesis Protocol Checklist

Date:

Synthesizer:

Number of Arrays:

Start time:

End time:

Before you start:

1. Pull the PDITC from the fridge (this takes ~1hr to come to room temperature)
2. Make certain you have enough boxes for the various incubations
3. Clean a 1L bottle, add stir bar, and dissolve 1 gram of chitosan in 500mL of DI water

Step 1: Plasma treatment of the arrays

1. Soak the arrays in 300mL of 95% ethanol for 5 minutes (50 rpm)
2. Dry the arrays for 5 minutes @ 500 rpm (60xg)
3. Plasma treat **two** trays at a time
   - A. Form seal for **3 minutes**
   - B. Plasma treat for **5-7 minutes**.
   - C. What color was the plasma? (circle one):

     No Color Light purple Light pink
   - D. While the arrays are being treated, prep the APTES solution

     APTES Solution: 180uL of APTES stock in 350mL of 95% ethanol
4. Proceed with protocol

Step 2: PDITC Soak

***Autoclave the chitosan solution after starting the PDITC incubation

1. Volume of PDITC solution you're prepping:________________liters (Standard: 350 mL)
2. Mass of PDITC added:________________________________grams (Standard: 0.72 grams)
3. Volume of pyridine added:________________________________liters (Standard: 35 mL)
4. Length of incubation:___________________________________hours (Standard: 2 hours)
5. Number of DMF washes:___________________________________(Standard: 2 washes)
6. Number of acetone washes:___________________(Standard: 1 wash, and then transfer to new acetone box for a **20-minute** soak)
7. Proceed with protocol

Shalek & Love Labs, MIT

Step 3: Oven incubation and chitosan preparation

1. After **20-minute soak**, remove arrays from acetone and spin down (1 min. @ 500 rpm)
2. Length of 70C incubation:__________________________________hrs (Standard: 2 hrs)
3. Chitosan protocol checklist: Did you… (Y / N responses)
   A. Autoclave chitosan (40-minute sterilization, 20-minute drying):________________
   B. Let solution come to room temperature (can also do this @ 4C):_______________
   C. Calibrate the pH Meter with appropriate buffers:____________________________
   D. Add 4 mL of glacial acetic acid (solution should be on stir plate):______________
   E. Let solution stand for **5 minutes**:____
   F. Add 50 mL of 5 M NaCl:____
   G. Titrate with 5 M NaOH:____
   H. Achieve pH of 6.0 – 6.1:____
   I. Remember, it is **critical** to make certain the you achieve a pH of 6.0 – 6.1 and that it holds. Parts A-H should be completed **before** the completion of the 2 hr 70C incubation.
4. Length of room temperature incubation:________________minutes (Standard: 20 minutes)
   A. Second check of chitosan pH:
5. Length of chitosan incubation:_________________________________(Standard: 1.5 hrs)
   A. Temperature = 37C, Rotation = 70 rpm

Step 4: In-well functionalization

1. Number of DI water rinses:_______________________________________(Standard: 4)
2. pH of aspartic acid solution:___________________________________(Standard: pH 10)
3. Length of overnight incubation:______________________________(Standard: 12-16 hrs)
4. Day 2: length of room temperature incubation @ 50 rpm:___________(Standard: 4 hrs)

Array Lot: <Your initials>_<Synthesis Date>_<Box Number>

Shalek & Love Labs, MIT

Appendix C: Master Mounting Protocol

1. Mix and degas PDMS in normal 1:10 ratio

2. While PDMS degases, use sandpaper to gently score back of silicon master and base plate to improve adhesion. Careful – silicon masters are brittle.

3. Wash back of master and base plate with 95% ethanol until no more dust is removed when wiping surface clean with paper towel.

4. Use gloved finger to spread vacuum grease on bottom of BasePlate2 around square holes where the nanowell arrays will be cast. You want a relatively thick layer, even on skinny parts between array holes, to make sure there is a seal between master and plate.

5. Carefully lower BasePlate2 onto the array side of the master making sure to not touch the array area with any of the greased surface. 4 array masters should fit into the 4 square holes. Gently slide plate against master to center the arrays.

6. Place Base Plate 1 on paper towels to catch PDMS running off plate.

7. Pour ~30 mL of mixed PDMS in center of Base Plate 1.

8. Place master/BasePlate2 sandwich on top of the PDMS.

9. Gently apply pressure in the center of the master while making circular motions to push PDMS out from between layers. You want to see PDMS coming out of all sides to ensure a complete coat.

10. Screw 6/32 screws into respective holes on base plate very gently. Too much pressure too fast may crack master. Do not fully tighten. Do your best to make screws even – look at width of crack between base plates on all sides and make equal.

11. Place both top plates on top.

12. Screw 10/24 screws into their holes just enough such that they catch. Again, do not fully tighten.

13. Place in 90C oven for 3 hours.

14. May need to do one dummy round of arrays to remove any PDMS or grease that got onto the nanowell features.

Shalek & Love Labs, MIT

Appendix D: Buffers Guide

CellCover10
Reagents
- CellCover (Anacyte Art. No. 800-125)
- FBS (Thermo Fisher Scientific Cat. No. 10437028)
- Sodium Carbonate (Sigma Cat. No. 223530-500G)

Working Concentrations
- 10% FBS
- 100 mM Sodium Carbonate

Bead Loading Buffer
Reagents
- Sodium Carbonate (Sigma Cat No. 223530-500G)
- BSA (Sigma Cat No. A9418-100G)
- Water (Thermo Fisher Scientific Cat No. 10977023)

Quick Preparation Guide (50 mL)
1. 2.5 mL 2 M Sodium Carbonate
2. 42.5 mL H_2O
3. Add 5 mL BSA (100 mg/mL)
4. Titrate with glacial acetic acid to achieve a pH of 10.0

Working Concentrations
- 100 mM Sodium Carbonate
- 10% BSA

Complete Lysis Buffer
Reagents
- Pre-lysis buffer
- 10% Sarkosyl (Sigma Cat No. L7414)
- 100% 2-Mercaptoethanol (Sigma Cat No. M3148-25ML)

Quick Preparation Guide (50 mL)
1. 47.25 mL Pre-Lysis Buffer
2. 250 uL 10% Sarkosyl
3. 500 uL BME

Working Concentrations
- 5 M Guanidine Thiocyanate
- 1 mM EDTA
- 0.50% Sarkosyl
- 1.0% BME

Shalek & Love Labs, MIT

Hybridization Buffer

Reagents

- 5 M NaCl (Thermo Fisher Scientific Cat No. 24740011)
- 1x PBS (Thermo Fisher Scientific Cat No. 10010023)
- 8% (v/v) PEG8000 (Sigma Cat No. 83271-500ML-F)

Quick Preparation Guide (50 mL)

1. 20 mL 5 M NaCl
2. 26 mL of PBS
3. 4 mL PEG8000

Working Concentrations

- 2 M NaCl

Wash Buffer

Reagents

- 5 M NaCl (Thermo Fisher Scientific Cat No. 24740011)
- 1 M $MgCl_2$ (Sigma Cat No.63069-100ML)
- 1 M Tris-HCl pH 8.0 (Thermo Fisher Scientific Cat No. 15568025)
- Water (Thermo Fisher Scientific Cat No. 10977023)
- 8% (v/v) PEG8000 (Sigma Cat No. 83271-500ML-F)

Quick Preparation Guide (50 mL)

1. 20 mL 5 M NaCl
2. 150 uL 1 M $MgCl_2$
3. 1 mL 1 M Tris-HCl pH 8.0
4. 24.85 mL H_2O
5. 4 mL PEG8000

Working Concentrations

- 2 M NaCl
- 3 mM $MgCl_2$
- 20 mM Tris-HCl pH 8.0

Array Quenching Buffers

Reagents

- Sodium Carbonate (Sigma Cat No. 223530-500G)
- 1 M Tris-HCl pH 8.0 (Thermo Fisher Scientific Cat No. 15568025)
- Water (Thermo Fisher Scientific Cat No. 10977023)

Quick Preparation Guide (50 mL)

1. 2.5 mL 2 M Sodium Carbonate
2. 500 uL 1 M Tris-HCl pH 8.0
3. 47 mL H_2O

Working Concentrations

- 100 mM Sodium Carbonate
- 10 mM Tris-HCl pH 8.0

Shalek & Love Labs, MIT

0.2% Chitosan Solution

Reagents

- Chitosan (Sigma Cat No. C3646-100G)
- Water (Thermo Fisher Scientific Cat No. 10977023)

Quick Preparation Guide

1. Add 1 gram of chitosan to 500 mL of DI water
2. Autoclave solution (40 minutes sterilization, 20 minutes dry)
3. Allow chitosan solution to come to room temperature, and then add 2-3 mL of glacial acetic acid.

Note: The chitosan will not start dissolving until the pH is acidic, and even then it will not fully dissolve. This is ok.

4. Add 50 mL 5 M NaCl, then titrate the chitosan solution with NaOH to bring the pH to 6.2.

TE - Tween Storage Solution

- 10 mM Tris pH 8.0 + 1 mM EDTA
- 0.01% Tween-20

Quick Preparation Guide (50 mL)

1. 49.95 mL H_2O
2. 5 uL Tween-20

TE - SDS Solution

- 10 mM Tris pH 8.0 + 1 mM EDTA
- 0.5% SDS

Quick Preparation Guide (50 mL)

1. 49.75 mL H_2O
2. 250 uL SDS

Shalek & Love Labs, MIT

Appendix E: Bead Removal Method 2 ("Spin-Out")

1. Remove membrane and place array into an empty 50 mL conical tube.

2. Ensure that the array is angled within the tube as shown below.
Note: The array might move around at this point, which isn't something to worry about.

3. Add 48-50 mL of Wash 1 solution (See Buffers Guide)

4. Place the insert so the array is secured angled as shown in the image below.

5. Secure the lid and seal with parafilm, if necessary.

6. Put the sealed conical in a centrifuge, making certain the PDMS surface of the array is facing away from the rotor arm (See Diagram Below).

7. Centrifuge at 2000 x g for 5 minutes to remove the beads.

8. At this point you should see a small, but visible, pellet of beads at the bottom of the tube.

9. Aspirate 5 - 10 mL of Wash 1 solution to enable easier removal of the array.

10. Remove the array and carefully position it over the top of the 50 mL tube.

11. Repeatedly wash any remaining beads from the surface of the array over the surface of the 50 mL falcon tube using 1 mL of Wash 1 remaining in the tube.

12. Spin again at 2000 x g for 5 minutes to pellet beads.

13. Aspirate all wash 1 solution except for ~ 1mL.
Note: Be careful to not disturb the pellet of beads.

14. Transfer beads to a 1.5 mL centrifuge tube and proceed to reverse transcription.

Shalek & Love Labs, MIT

Appendix F: Imaging in Array

1. When pre-imaging cells, cells should be loaded first as beads will obstruct view of many cells and beads autofluorescence can interfere with the signal.

2. Obtain a cell or tissue sample and prepare a single cell suspension using your preferred protocol.

3. Count cells using a hemocytometer and resuspend 10,000 cells in 200 uL of cold CellCover (Anacyte).

4. Incubate cells in at 4C for 1 hour.

5. After the cells have been fixed, perform antibody staining at 4C.
Note: Some epitopes may no longer be available as a result of the fixation process.

6. Wash cells twice with 1x PBS, resuspend in 200 uL of CellCover10 buffer (pH 10 + 10% FBS; **See Appendix D: Buffers Guide**), and place on ice.
Note: CellCover != CellCover10.

7. Obtain empty functionalized array(s), aspirate storage solution and soak each array in 5 mL of CellCover10 buffer (**See Appendix D: Buffers Guide**).

8. Aspirate media and load your fixed cells onto each array in a dropwise format.

9. Gently rock the array(s) in the x & y direction for 5 minutes.

10. Wash each array twice with 5 mL of CellCover10 (pH 10 + 10% FBS), then solvate each them in 5 mL of CellCover (No FBS).

11. Place a lift slip on each array, then image with a microscope.

12. After imaging, wash each array in 5 mL of CellCover10 media.

13. Immediately load beads using the bead loading protocol provided above.
Note: In the protocol provided above, beads are washed and loaded in BLB. When loading cells first, you will replace BLB with CellCover10 for all steps. After beads are loaded and sufficiently washed, you will wash the array 4x with CellCover10 without FBS and solvate arrays in CellCover.

14. Proceed with membrane sealing.

Shalek & Love Labs, MIT

Appendix G: Shopping List

Device Manufacturing

Equipment

- Dow Corning Sylgard 184 Silicone Encapsulant Clear 0.5 kg kit (Part No. 184 SIL ELAST KIT 0.5 PG)
- Protolabs Custom Array Molding Plates (Please refer to www.shaleklab.com/seq-well
  - Make out of aluminum and make sure to tap holes only on base plateBasePlate1 v3.1 (Bottom plate you mount the wafer to)
- BasePlate2 v3.1 (Divider for arrays)
- TopPlate1 v3.1 (Plate that holds the glass slides)
- TopPlate2 v3.1 (Top plate)
- 45 micron Silicon Master Wafer Size (Please refer to www.shaleklab.com/seq-well)
- Master, pre-silanized – (FlowJem, Inc. Toronto, Canada)
- Corning 72x25 Microscope Slides (Corning Life Sciences Cat. No. 2947)
- 6/32 ¼" Hex Screws
- 5/8" Hex 10/24 Screws
- Hex Screwdriver
- Vacuum grease
- 80 grit sandpaper
- 95% ethanol in spray bottle

Array Functionalization

Equipment

- Plasma Oven (Harrick Plasma PDC-001-HP)
- 2x 30-slide rack slotted (VWR Cat No. 25461-014)
- 16x20 cm staining dish (VWR Cat No. 25461-018)
- Vacuum Desiccator (VWR Cat No. 24988-164)
- Sterile 4-well dishes (Thermo Fisher Scientific Cat No. 267061)

Reagents

- 200 proof ethanol (VWR Cat No. 89125-188)
- (3-Aminopropyl)triethoxysilane (Sigma Cat No. A3648)
- Acetone (Avantor Product No. 2440-10)
- p-Phenylene Diisothiocyanate (PDITC) (Sigma Cat No. 258555-5G)
- Pyridine (Sigma 270970-1L)
- Dimethylformamide (DMF) (Sigma Cat No. 227056-1L)
- Chitosan (Sigma Cat No. C3646-100G)
- Poly(L-glutamic) acid sodium solution (Sigma Cat No. P4761-100MG)
- 5M NaCl (Sigma Cat No. S6546-1L)
- Sodium Carbonate (Sigma Cat No. S2127-500G)

Shalek & Love Labs, MIT

Buffer Reagents

Bead Loading Buffer

- Sodium Carbonate (Sigma Cat No. 223530-500G)
- BSA (Sigma Cat No. A9418-100G)
- Water (Thermo Fisher Scientific Cat No. 10977023)

Complete Lysis

- Guanidine Thiocyanate, (Sigma Cat No. AM9422)
- 0.5 M EDTA (Thermo Fisher Scientific Cat No. 15575020)
- Water (Thermo Fisher Scientific Cat No. 10977023)
- 10% Sarkosyl (Sigma Cat No. L7414)
- 100% 2-Mercaptoethanol (Sigma Cat No. M3148-25ML)

Hybridization Buffer

- 5 M NaCl (Thermo Fisher Scientific Cat No. 24740011)
- 1x PBS (Thermo Fisher Scientific Cat No. 10010023)
- PEG-8K (50%) (Fisher Scientific Cat No. BP337-100ML)

Wash Buffer

- 5 M NaCl (Thermo Fisher Scientific Cat No. 24740011)
- 1 M $MgCl_2$ (Sigma Cat No.63069-100ML)
- 1 M Tris-HCl pH 8.0 (Thermo Fisher Scientific Cat No. 15568025)
- Water (Thermo Fisher Scientific Cat No. 10977023)

Array Quenching Buffer

- Sodium Carbonate (Sigma Cat No. 223530-500G)
- 1 M Tris-HCl pH 8.0 (Thermo Fisher Scientific Cat No. 15568025)
- Water (Thermo Fisher Scientific Cat No. 10977023)

RT Reagents

- UltraPure Distilled Water (Thermo Fisher Scientific Cat No. 10977023)
- Maxima 5x RT Buffer/Maxima H-RT (Thermo Fisher Scientific Cat No. EPO0753)
- 20% Ficoll PM-400 (Sigma Cat No. F5415-50mL)
- 10 mM dNTPs (New England BioLabs Cat No. N0447L)
- RNAse Inhibitor (Thermo Fisher Scientific Cat No. AM2696)
- Template Switching Oligo (Order from IDT)

Exonuclease Reagents

- Exonuclease I (*E. coli*) (New England Biolabs Cat No. M0293S)

Second Strand Synthesis Reagents

- Maxima 5x RT Buffer/Maxima H-RT (Thermo Fisher Scientific Cat No. EPO0753)
- 10 mM dNTPs (New England BioLabs Cat No. N0447L)
- dN-SMART Oligo (Order from IDT)
- UltraPure Distilled Water (Thermo Fisher Scientific Cat No. 10977023)
- Klenow Exo- (New England BioLabs Cat No. M0212S)
- 30% PEG8000 (Sigma-Aldrich 89510-1KG-F)

Shalek & Love Labs, MIT

PCR Reagents

- IS PCR Primer (Order from IDT)
- KAPA HiFi Hotstart Readymix PCR Kit (Kapa Biosystems Cat No. KK-2602)

Nextera Reagents

- Nextera XT DNA Library Preparation Kit (96 samples) (Illumina FC-131-1096)
- New-P5-SMART PCR Hybrid Oligo (Order from IDT)
- Nextera N70X Oligo (Order from Illumina)

Operating Equipment

- Polycarbonate (PCTE) 0.01 micron 62x22 mm precut membranes, 100 count (Sterlitech Custom Order)
- mRNA Capture Beads (Chemgenes Cat No. MACOSKO-2011-10)
- Lifter Slips, 25x60mm (Electron Microscopy Science Cat No. 72186-60)
- Agilent Clamps (Agilent Technologies Cat No. G2534A)

Sequences

Barcoded Bead SeqB:
5'–Bead–Linker--TTTTTTTAAGCAGTGGTATCAACGCAGAGTAC-
JJJJJJJJJJJJNNNNNNNNTTTTTTTTTTTTTTTTTTTTTTTTTTTTTT--3'

Template Switching Oligo (TSO):
AAGCAGTGGTATCAACGCAGAGTGAATrGrGrG

dN-Smart Randomer (dN-SMRT):
AAGCAGTGGTATCAACGCAGAGTGANNNGGNNNB

Smart PCR Primer (TSO_PCR):
AAGCAGTGGTATCAACGCAGAGT

New-P5-SMART PCR Hybrid Oligo (P5-TSO_Hybrid):
AATGATACGGCGACCACCGAGATCTACACGCCTGTC-
CGCGGAAGCAGTGGTATCAACGCAGAGT*A*C

Custom Read 1 Primer (Read_1_Custom_SeqB):
GCCTGTCCGCGGAAGCAGTGGTATCAACGCAGAGTAC

Shalek & Love Labs, MIT

Seq-Well Cost Object Breakdown (Non-discounted prices were used in this cost model)

**Array Synthesis**	**Cost (USD)**	**Per Array (USD)**	**Part Number**
PDMS	109.23	1.37	184 SIL
ProtoLabs Mounts	1100.84	11.01	Protolabs Quote
Silicon Wafer	1070	10.70	FlowJem Quote
Corning Microscope slides	320	0.22	Cat no. 2947
Chitosan	156	0.05	C3646-100G
APTES (100mL)	73.7	0.00	A3648-100ML
Acetone	180.06	1.80	2440-10
PDITC	116	1.29	258555-5G
Pyridine	190	0.45	270970-1L
DMF	321	2.82	319937-4L
L-Aspartic Acid	29	0.03	A9256-100G
5M NaCl	51.2	0.49	S6546-1L
Sodium Carbonate	57.2	0.00	S2127-500G
	**Total (per array)**	30.22	

**Bead Synthesis**	**Cost (USD)**	**Per Array (USD)**	**Part Number**
**Bead Synthesis**	4500	45	Macosko-Chemgenes
	**Total (per array)**	45.00	

**Loading Buffers**	**Cost (USD)**	**Per Array (USD)**	**Part Number**
Sodium Carbonate	54.1	0.01	S2127-500G
BSA	641	0.01	A9418-100G
Water	185	0.37	10977023
Guanidine Thiocyanate	284	0.01	AM9422
0.5M EDTA	81.25	0.01	15575020
10% sarkosyl	117	0.01	L7414-10ML
100% BME	80.6	0.01	M3148-250ML
5M NaCL	51.2	0.01	S6546-1L
1xPBS	196	0.98	10010049
1M Mg2Cl	119	0.01	63069-100ML
1M Tris-HCl pH 8.0	69.8	0.1	15568025
	**Total (per array)**	46.53	

**Downstream Processing**	**Cost (USD)**	**Per Array (USD)**	**Part Number**
**RT**			
Maxima RT Buffer (5X)	680	17.00	EPO0753
PEG8K	105	0.01	F5415-50ML

10 mM dNTP	250	1.25	N0447L
RNAse Inhibitor	448	4.48	AM2696
TSO	IDT Quote	0.01	IDT quote
**Second Strand**			
Klenow	244	6.10	M0212M
10 mM dNTP	250	1.25	N0447L
**PCR**			
Exonuclease digestion	281	0.75	M0293L
ISPCR	IDT Quote	0.01	IDT quote
KAPA HIFI	640.5	61.00	KK-2602
**Nextera**		0.00	
Nextera XT library prep kit	3270	96.18	FC-131-1096
P5 hybrid olgio	IDT Quote	0.01	IDT quote
Nextera N7XX primer	IDT Quote	0.01	IDT quote
**Sequencing QC**			
Tape Station - D5000 screentapes	527	25.00	5067-5592
Tape Station - D5000 Reagents	179	8.50	5067-5593
	**Total (per array)**	221.56	
**Sequencing**			
Nextseq 500 kit	1570		Illumina quote
QC librarys (WTA and NTA)			
*Assume we load 2 arrays/run			

**Seq-Well S^3 Protocol Steps**	**Costs per array**	**10x V3 3' Protocol Steps (BMC)**	**Costs per array**
Array Synthesis	30.22	Setup Costs	1235
Bead Synthesis	45.00	Price per sample	2100
Loading Buffers	46.53	Sequencing	3010
Downstream Processing	221.56		
Sequencing	785.00		
**Seq-Well Pipeline Costs**	**Costs per array**	**10x V3 3' Pipeline Costs**	**Costs per array**
Pre-Seq Costs (w/ synthesis)	343.31	Pre-Seq Costs (w/ setup)	3335
Pre-Seq Costs (w/o synthesis)	313.09	Pre-Seq Costs (w/o setup)	2100
Total Costs (w/ synthesis)	1128.31	Total Costs (w/ setup)	6345
Total Costs (w/o synthesis)	1098.09	Total Costs (w/o setup)	5110
